# Research based on serine metabolism indicates mesenchymal stem cells alleviate psoriasis by regulating the PSPH-PINK1-Parkin-NLRP3 pathway in HaCaT

**DOI:** 10.1186/s13287-026-04964-z

**Published:** 2026-03-28

**Authors:** Qing Lin, Yunfei Ji, Bin Yang, Rongjia Zhu, Ping Song, Robert chunhua Zhao

**Affiliations:** 1 Department of Dermatology, China Academy of Chinese Medical Sciences Guanganmen Hospital, Beijing, 100053 China; 2 Institute of Basic Medical Sciences Chinese Academy of Medical Sciences, School of Basic Medicine Peking, Union Medical College, Beijing, 100010 China; 3 Beijing Key Laboratory of Artificial Intelligence and Cell-Based Medical Engineering for Interdisciplinary Innovation and Clinical Translation, Beijing, 100010 China; 4 State Key Laboratory of Common Mechanism Research for Major Diseases, Beijing, 100010 China; 5 Department of Pathology, China Academy of Chinese Medical Sciences Xiyuan Hospital, Beijing, 100091 China; 6 Department of Dermatology, China Academy of Chinese Medical Sciences Xiyuan Hospital, Beijing, 100091 China

**Keywords:** Mesenchymal stem cells, Psoriasis, Serine metabolism, PSPH, PINK1-Parkin mitophagy, NLRP3

## Abstract

**Background:**

Psoriasis is a refractory immune-related disease. In recent years, it has been discovered that mesenchymal stem cells (MSCs) can be used as a new therapeutic approach for psoriasis, but their potential therapeutic mechanism remains unclear. This study aims to explore the role of MSCs in the treatment of psoriasis.

**Methods:**

We employed a mouse psoriasis model induced by imiquimod (IMQ) in vivo and a co-culture system of MSCs and HaCaT keratinocytes (KCs) cell line in vitro. These approaches allowed us to investigate the effect of MSCs on the levels of inflammatory factors and the activation of inflammasomes in both contexts. Mouse-targeted amino acid sequencing, transmission electron microscopy for in vitro observation, immunofluorescence for both in vivo and in vitro analyses, and siRNA transfection in vitro were employed in this study.

**Results:**

Our results showed that MSCs significantly improved the skin lesion of mice with psoriasis, and reduced the levels of inflammatory factors and chemokines including IL-1β, IL-6, IL-8, TNF-α, MCP-1, CCL7, CCL20 and CCL27 in the mouse skin lesion areas and M5- induced psoriatic KCs models in vitro. Likewise, MSCs repaired the skin barrier by enhancing claudin-1 expression in vivo. In addition, MSCs increased KRT1 and decreased KRT6 levels in vivo and in vitro. Amino acid metabolism analysis showed that MSCs could improve the serine metabolism level in the mouse skins and upregulated the key enzyme phosphoserine phosphatase (PSPH) in serine metabolism. In vitro experiments demonstrated that knockdown of PSPH could reverse the therapeutic effects of MSCs on psoriasis. Furthermore, studies in vitro and in vivo revealed that MSCs can activate the PINK1-Parkin pathway. It was specifically manifested by elevated levels of PINK1, Parkin, p-Parkin, Beclin-1, and LC3B-II/I, coupled with a reduction in P62 protein. Subsequently, the activation of PINK1-Parkin led to decreased expressions of IL-1β, IL-6, IL-8, TNF-α, CCL7, CCL20, CCL27, and MCP-1. In vitro and in vivo experiments indicated that MSCs can reduce the levels of IL-1β, IL-6, IL-8, TNF-α, CCL7, CCL20, CCL27, and MCP-1 by inhibiting the activation of NLRP3 inflammasomes. Meanwhile, PSPH knockdown in vitro can reverse the activating effects of MSCs on the PINK1-Parkin, as shown by decreased levels of PINK, Parkin, p-Parkin, Beclin-1, and LC3B-II/I, concurrently with an elevation in P62..

**Conclusions:**

The results of this study indicated that MSCs can alleviate IMQ-induced psoriasiform dermatitis in mice by upregulating serine metabolism. The key serine metabolism enzyme PSPH may enhance PINK1/Parkin-mediated mitochondrial autophagy in psoriatic HaCaT and inhibit NLRP3 inflammasome activation in HaCaT cells, thereby alleviating skin inflammatory responses and suppressing skin p

roliferation in psoriatic mice.

**Graphical Abstract:**

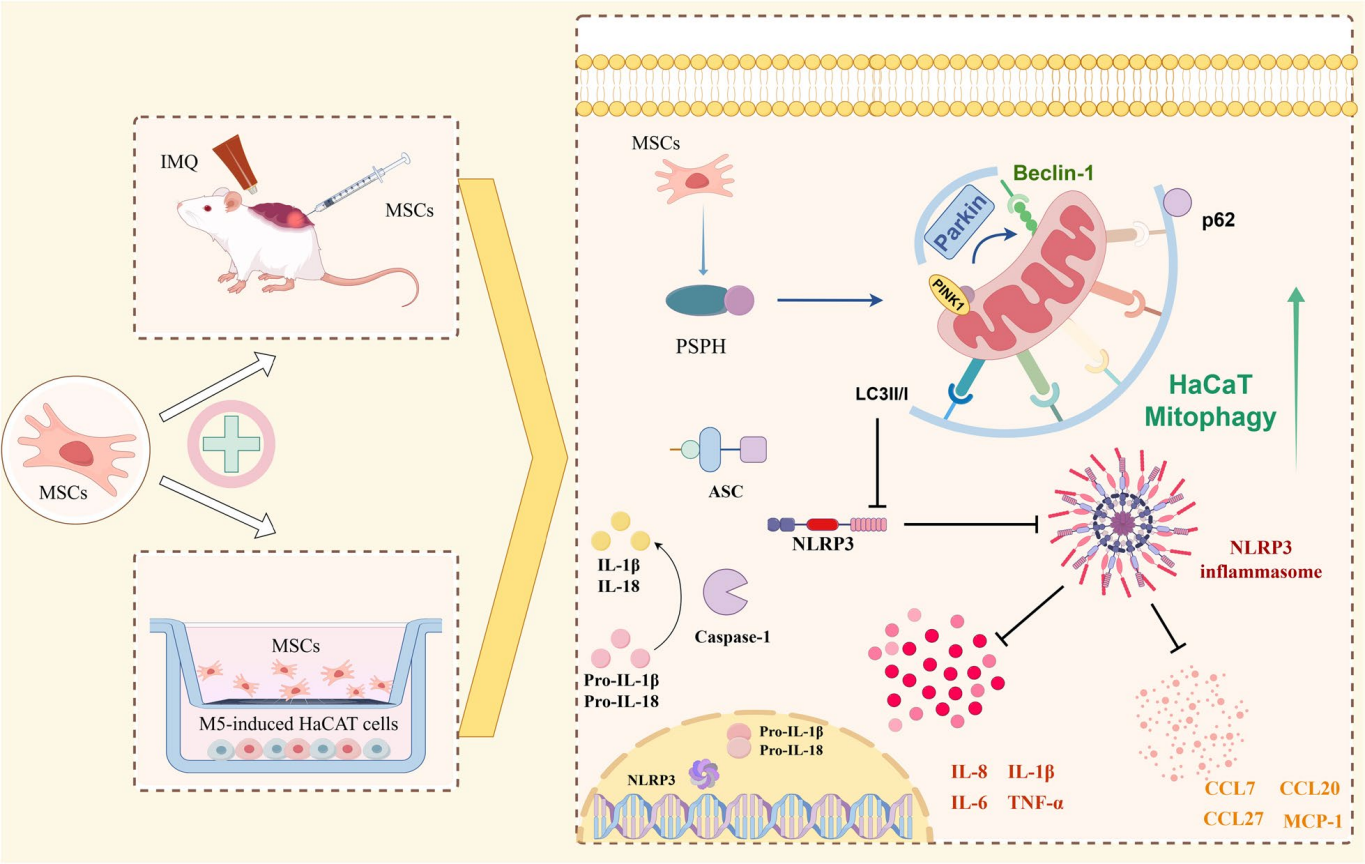

**Supplementary Information:**

The online version contains supplementary material available at 10.1186/s13287-026-04964-z.

## Highlights

1. MSCs can inhibit NLRP3 inflammasomes activation in psoriatic mouse skin, potentially through the regulation of PSPH and subsequent engagement of the PINK1-Parkin mitophagy pathway in psoriatic HaCaT in this study.
2. MSCs can upregulate dysregulated serine metabolism in IMQ-induced mice in our experiments.

**Supplementary Information:**

The online version contains supplementary material available at 10.1186/s13287-026-04964-z.

## Introduction

Psoriasis is a chronic inflammatory skin disease that affects approximately 2–3% of the global population [1]. It is characterized by recurrent autoimmune responses, which result in excessive hyperproliferation and aberrant differentiation of KCs [2, 3]. The prevailing hypothesis suggests that the stimulation of inflammatory factors leads to the hyperproliferation of KCs, which subsequently promotes the secretion of pro-inflammatory cytokines, exacerbating the inflammatory response [4–6]. Consequently, psoriatic KCs play a central role in the inflammatory pathogenic loop of psoriasis, functioning both as triggers of immune responses and as pro-inflammatory effectors [7]. Immunomodulatory drugs have shown some success in treating psoriasis [8]. Biologic therapies directed against TNF-α, IL-17, and IL-23 have been introduced, demonstrating considerable clinical efficacy. Nevertheless, treatment with these agents is associated with a susceptibility to disease recurrence and potential adverse effects, including nasopharyngitis, upper respiratory tract infections, and tuberculosis [9]. These limitations underscore the pressing need to develop safer alternative therapeutic strategies for psoriasis management.

MSCs are multipotent progenitor cells capable of modulating immune responses [10, 11]. Interestingly, their immunomodulatory properties can be enhanced by inflammatory cytokines [12, 13]. Thus, MSCs exhibit significant anti-inflammatory abilities in inflammatory microenvironments and are commonly used to treat psoriasis. Several case reports have documented the therapeutic effects of MSC treatment for psoriasis [14, 15]. For example, adipose-derived MSCs were utilized for one patient with psoriasis vulgaris and another with psoriatic arthritis. The patients' Psoriasis Area Severity Index (PASI) scores of the patients decreased (from 21.6 to 9.0 and 24.0 to 8.3, respectively) after multiple MSCs infusions, with no serious adverse events reported [16]. Given their easy accessibility, multipotency, and active paracrine activity, adipose-derived MSCs have emerged as the preferred MSC type for immunotherapy [17]. Therefore, adipose-derived MSCs were adopted in our current study. Nevertheless, the anti-inflammatory mechanisms of MSCs in psoriatic KCs remain poorly understood.

One study has indicated that amino acid and carnitine metabolism is significantly altered in psoriasis, particularly the metabolism of essential amino acids (EAAs), branched-chain amino acids (BCAAs), and long-chain carnitines (LC (C0)), which may play critical roles in the disease's pathogenesis [18]. Another study revealed 11 significant alterations in amino acid metabolism pathways within psoriatic lesion. Branched-chain amino acids, tyrosine and arginine metabolism have a causal relationship with the occurrence of psoriasis and may play a crucial role by promoting the proliferation and differentiation of the KCs or immune-related pathways [19]. The hyperactivation of serine metabolism has been linked to abnormal cellular nucleotide, protein, and lipid synthesis, mitochondrial dysfunction, and epigenetic modifications, driving malignant transformation, unlimited proliferation, metastasis, immunosuppression, and drug resistance in tumor cells [20]. PSPH, an enzyme involved in the serine metabolism pathway, has been implicated in cancer progression and survival [21]. However, whether MSCs exert anti-inflammatory effects by regulating amino acid metabolism in psoriatic KCs has not been studied.

Mitochondria are dynamic organelles with multiple functions [22]. They participate in necrotic cell death and programme apoptotic and are crucial for cell metabolism and survival [23]. Mitophagy serves as a cytoprotective mechanism to remove superfluous or dysfunctional mitochondria and maintain mitochondrial fine-tuning numbers to balance intracellular homeostasis [24]. The PINK1-Parkin pathway is a classical mechanism for the removal of damaged mitochondria [25]. Evidence suggests that activating the PINK1-Parkin pathway enhances mitochondrial autophagy (mitophagy) in HaCaT KCs following photodamage, thus promoting their functional recovery [26–28]. Ligustilide could ameliorate the neuronal injury against ischemia stroke by promoting mitophagy via PINK1/Parkin. Targeting PINK1/Parkin mediated mitophagy with LIG treatment might be a promising therapeutic strategy for ischemia stroke [29]. NLRP3, a core component of the inflammasomes, plays a pivotal role in triggering downstream inflammatory changes involving IL-1β, significantly impacting psoriasis [30]. PINK1-Parkin-mediated mitophagy also protects against inflammation in acute kidney injury disease by suppressing IL1β processing via autophagic degradation of the NLRP3 inflammasome [31]. A study detected the major microbial metabolites of tryptophan on the skin surfaces, suggesting that the quinolinic acid was significantly lower in the lesional skin of patients with psoriasis than in that of healthy subjects and correlated negatively with the severity of psoriasis [32]. Quinolinic acid markedly reduced skin inflammation through an AhR-dependent mechanism, leading to the inhibition of NLRP3 inflammasome activation.

However, whether MSCs can ameliorate mouse psoriatic dermatitis by improving HaCaT PSPH-PINK1-Parkin-NLRP3 pathway was unknown. Here, we aim to uncover the specific mechanisms by which MSCs modulate psoriatic KCs and provided a new perspective on MSCs therapy for psoriasis.

## Materials and methods

### Cell culture and establishment of M5 models

MSCs were isolated from liposuction waste tissue obtained from a 28-year-old male patient at Peking Union Medical College Hospital, with approval from the Institutional Ethics Committee (Approval No. 82300129). The cells were cultured in DMEM/F12 medium (Gibco, Grand Island, NY, USA) supplemented with 20% fetal bovine serum (Gibco, Grand Island, NY, USA) and 1% PS. Commercially available human HaCaT KCs [33] were obtained from the Cell Resource Center, School of Basic Medicine, Chinese Academy of Medical Sciences & Peking Union Medical College (Beijing, China). Cells were maintained in Dulbecco's modified Eagle's medium (DMEM, Gibco, Grand Island, NY, USA) containing 10% Fetal Bovine Serum (Gibco, Grand Island, NY, USA) and 1% PS cultured in an incubator with 5% CO_2_ at 37 °C. The HaCaTs (KCs) cells were placed in the lower layer of the chambers, and MSCs spread plates were co-cultured (Corning, NY, USA) in the upper layer of the chambers for 24 h. The experimental use of MSCs was limited to the 3rd-5th generation. MSCs characterization provided in the supplementary materials. A psoriasis-like KCs model was established by adding M5 cocktail cytokine (IL-17A, IL-22, Oncostatin M, IL-1α, and TNF-α(PeproTech, Rocky Hill, USA), each at a final concentration of 10 ng/ml) into the medium of HaCaTs KCs [34]. When HaCaTs cells reached approximately 50% confluence, the culture medium was replaced with serum-free DMEM and maintained for 24 h. Subsequently, cells were stimulated with the M5 inflammatory cytokine cocktail for an additional 24 h.

Lipogenic and osteogenic differentiation kits provided by Procell (Wuhan, China). The lipid-induced group was stained with oil red O to identify lipid droplet formation after 13 days of directed culture, while osteogenic induction was detected by alizarin red staining for calcium nodules after 6 days of differentiation cycle. Flow cytometry confirmed the immunophenotype of adipose-derived MSCs, which also exhibited robust osteogenic and adipogenic differentiation potential upon directed induction. Detailed materials can be found in supplementary materials.

### RNA isolation and quantitative real-time PCR analysis

Total RNA extraction from cells or mouse skin tissues was performed with Trizol (Invitrogen, Carlsbad, CA, USA), after which 2 μg of RNA served as a template for cDNA synthesis using a reverse transcription kit (TAKARA, Tokyo, Japan). SYBR Green-based quantitative real-time PCR was performed on a CFX96 Touch™ Real-Time PCR Detection System (Bio-Rad, USA). The thermal cycling conditions were as follows: initial denaturation at 95 ℃ for 10 s, followed by 40 cycles of 95 °C for 10 s, 60 °C for 20 s, and 72 °C for 20 s. Relative gene expression, normalized to the internal control GAPDH, was determined using the 2^−ΔΔCt^ method. The primer sequences used in this study are listed in supplementary materials.

### UPLC-ESI–MS/MS analytical methods

The experiment was provided by Shanghai Luming Biotechnology (Shanghai, China), using Liquid Chromatography Mass Spectrometry for the targeted analysis of amino acid metabolism in mouse skins. Metabolite analysis was performed using an ACQUITY UPLC I-Class plus system coupled with a Q-Exactive mass spectrometer in heated electrospray ionisation mode with both positive and negative ion modes. Chromatographic separation employed an ACQUITY UPLC HSS T3 column with gradient elution using a mobile phase of water and acetonitrile containing 0.1% formic acid. Mass spectrometry scanning covered m/z 100–1000 with resolutions of 70,000 (full scan) and 17,500 (MS/MS), employing collision energies of 10, 20, and 40 eV. For quality control, a QC sample was prepared by pooling all samples to monitor system stability. Raw data underwent preprocessing and compound identification using Progenesis QI, validated against databases including HMDB. Model reliability was assessed via PCA, OPLS-DA/PLS-DA combined with sevenfold cross-validation and 200 permutation tests. Differentially expressed metabolites were selected based on VIP > 1.0 and *p* < 0.05.

### Western blotting

Mouse skin tissues were ground in liquid nitrogen, or cells were collected and added to pre-cooled RIPA lysate (Epizyme, Shanghai, China). Protein concentration was determined using a BCA assay (Epizyme, Shanghai, China). Proteins were separated by 4–20% gradient SDS-PAGE (Epizyme, Shanghai, China) and transferred to PVDF membranes (Bio-Rad, Marnes-la-Coquette, France). Membranes were blocked for 10 min at room temperature and then incubated overnight at 4 °C with the following primary antibodies: anti-LC3B (1:1000, Abcam, Cambridge, UK), anti-P62 (1:1000, CST, Danvers, MA, USA), anti-Beclin-1 (1:1000, CST, Danvers, MA, USA), anti-Parkin (1:1000, CST, Danvers, MA, USA), and anti-PINK1 (1:1000, CST, Danvers, MA, USA),anti-p-Parkin (1:500, Affinity, Shanghai, China), anti-ASC(1:1000, CST, Danvers, MA, USA),anti-NLRP3(1:1000, Abcam, Cambridge, UK), anti-KRT1(1:1000, Abcam, Cambridge, UK),anti-KRT6(1:1000,Proteintech,Wuhan, China),Anti-GAPDH (1:5000, Proteintech, Wuhan, China) served as the loading control. After incubation with HRP-conjugated secondary antibodies (1:2000–1:5000, Proteintech, Wuhan, China) for one hour at room temperature, protein bands were visualized using an ECL detection system (Yeasen, Shanghai, China). Band intensities were quantified using ImageJ software (NIH) and normalized to GAPDH expression. Each experiment was performed in triplicate.

### Flow cytometry assay

After digestion and centrifugation, the MSCs cell samples were resuspended to obtain a single-cell suspension. To assess the expression of cell surface markers, several antibodies, including CD73, CD90, CD105, CD29, CD44, CD34, CD45, CD106, CD206, and HLA-DR (BD, San Jose, CA, USA), were selected. Each antibody was used at a concentration of 2 µg and was diluted in 100 µl of PBS for labeling. The resuspended cell samples were then analyzed using a flow cytometer, ensuring that at least 1 × 10^4^ cells were collected per sample. Finally, the expression of each marker was evaluated through the analysis of Flow Jo software (TreeStar), with positive expression (≥ 95%) and negative expression (≤ 2%) being determined to facilitate quantitative analysis of cell phenotypes. Detailed materials can be found in supplementary materials.

### Knockdown of PSPH by siRNA

Human siRNA-PSPH and siRNA-NC (Jiman, Shanghai, China) and Lipofectamine 3000 (Thermo Fisher, MA, USA) were used to transfect HaCaTs (5 × 10^^5^) according to the manufacturer's instructions. After 24 h, cells were subjected to the specified stimulations. qRT-PCR was performed to examine transfection efficiency.

### Transmission electron microscope

For ultrastructural analysis, the collected cells were fixed in 2.5% glutaraldehyde at 4 °C and post-fixed with 1% osmium tetroxide for 2 h at room temperature under light protection. The samples were then dehydrated through a series of ethanol gradients. Infiltration with a mixture of acetone and EPON 812 resin was performed, followed by embedding and polymerization at 37 °C overnight, and then at 60 °C for 48 h. Ultrathin Sects. (70–90 nm) were stained with uranyl acetate and lead citrate, and imaging was conducted using a Hitachi electron microscope (Hitachi, Tokyo, Japan).

### ELISA

Commercial ELISA kits were employed to quantify target protein levels in tissue homogenates and cell culture supernatants according to the manufacturers' instructions. The kits used included those for human IL-6, IL-8, TNF-α, and IL-1β, as well as mouse IL-6, TNF-α, and IL-1β (Servicebio, Wuhan, China), and a mouse CXCL1 kit (Thermo Fisher Scientific, Massachusetts, USA).

### Histopathology and immunohistochemistry

Skin tissue samples were fixed in 4% paraformaldehyde and were embedded in paraffin. Paraffin-embedded sections were deparaffinized and rehydrated, followed by staining with Hematoxylin and Eosin (H&E).To repair mouse epidermal antigens, the samples were incubated overnight at 4 °C with the following primary antibodies: rabbit anti-KI67 (1:200, Abcam, Massachusetts, USA), rabbit anti-claudin-1 (1:1000, Proteintech, Wuhan, China), rabbit anti-KRT1 (1:1000, Proteintech, Wuhan, China), and rabbit anti-KRT6 (1:1000, Proteintech, Wuhan, China). The next day, the samples were washed and incubated with an HRP-labeled secondary antibody (Servicebio, Wuhan, China). Nuclei stained with hematoxylin appeared blue, while positive expression was indicated by brownish-yellow DAB staining. The samples were then analyzed using a light microscope (Olympus, Tokyo, Japan).

### Immunofluorescence

Mouse skin tissue samples were fixed with 4% paraformaldehyde and embedded in paraffin. The first target staining was performed, followed by antibody stripping, then the second target staining and antibody stripping, and finally nuclear staining, with observation using a microscope. Primary antibodies: ASC (1:10,000, Servicebio, Wuhan, China), NLRP3 (1:1,000, Wuhan,China).Secondary antibody: HRP-conjugated goat anti-rabbit IgG (1:500, Servicebio, Wuhan, China).

After permeabilizing and blocking HaCaTs cells, the primary antibody was added and incubated overnight at 4 °C. Rabbit anti-ASC antibody (1:500, Abcam, Cambridge, UK) was used. After washing the cells three times, they were incubated with FITC-conjugated goat anti-rabbit IgG (1:500, Abcam, Cambridge, UK) for 1 h. The cells were stained with DAPI for 10 min and analyzed using a confocal microscope (Leica TCS SP8 STED, China). Primary antibodies: goat anti-rabbit ASC (1:500, ab Cambridge, UK), goat anti-rabbit NLRP3 (1:50, Proteintech,Wuhan, China).

### Animal experiments

All animal experiments were performed in accordance with the ARRIVE guidelines 2.0 (animal research: reporting of in vivo experiments). The study was approved by the Beijing Maidcona Animal Experiment Co., Ltd, Institutional Animal Care and Use Committee (IACUC) (Approval No. MDKN-2024–016). Male C57/BL6 mice (aged 8 weeks, weighing 20 g) were used in this study. The mice were housed under a 12-h light/dark cycle at a constant temperature (22 ± 2 °C) and humidity (50 ± 10%) with free access to food and water. All efforts were made to minimize animal suffering and to reduce the number of animals used. For the IMQ-induced psoriasis model, the experimental design, including sample size estimation, randomization, and blinding during outcome assessment, followed the essential requirements of the ARRIVE 2.0 guidelines. Mice received a daily topical application of imiquimod cream (62.5 mg/kg, Mingxin Sichuan, China) for six consecutive days. The dorsal skin lesions in IMQ-induced psoriasis mice measure 4 cm in length and 3 cm in width. MSCs cells (2 × 10^6^ cells) were administered via subcutaneous injection on days 1 and 4[35], with tissue collection performed on day 6. An objective scoring system based on the clinical Psoriasis Area and Severity Index (PASI) was applied to score the severity of inflammation of the dorsal skin of mice. Thus, erythema, scaling, and thickening were scored on a scale from 0 to 4, as follows: 0, none; 1, slight; 2, moderate; 3, severe; and 4, very severe. The cumulative score served as a measure of the severity of psoriasis (0–12).

Animals were anesthetized using isoflurane at a concentration of 2–2.5%. Isoflurane anesthesia was administered to facilitate hair removal from the mice, followed by the application of IMQ cream. Euthanasia Procedure Details: mice were first transferred to a pre-cleaned, transparent induction chamber. Compressed carbon dioxide (CO₂) gas was introduced at a controlled flow rate (30%-50% of the chamber volume per minute). This flow rate was designed to gradually increase the CO₂ concentration to avoid animal distress. After the animals lost consciousness (as determined by the loss of righting reflex) and ceased breathing, CO₂ exposure was maintained for at least one additional minute to ensure complete respiratory arrest. Subsequently, mice were immediately removed from the induction chamber, and death was confirmed by a secondary physical method of euthanasia—cervical dislocation. During cervical dislocation, the operator firmly secured the mouse's head and neck with one hand while grasping the base of the tail with the other, applying a swift and consistent pulling force to separate the cervical vertebrae from the skull, thereby destroying brainstem function. Death was confirmed by observing the absence of at least three of the following indicators: cessation of spontaneous breathing, fixed and dilated pupils, lack of response to a strong mechanical stimulus (e.g., toe pinch), and cardiac arrest (confirmed via direct observation following thoracotomy). The entire procedure was conducted to minimize animal pain, distress, and anxiety to the greatest extent possible. Following euthanasia, subsequent tissue collection and processing were performed immediately.

### Imaging analysis

Image analysis was conducted by ImageJ software (National Institutes of Health). The surface area of astrocytes was quantified after staining them with phalloidin. The total signal area is obtained using ImageJ, then divided by the number of cells to calculate the average area per cell within each microscopic field of view.

### Statistical analysis

All data are expressed as mean ± standard deviation. Statistical analysis was performed using GraphPad Prism 10.0 software. Comparisons between two groups were conducted using Student's t-test, while comparisons among multiple groups were performed using one-way ANOVA or two-way ANOVA. *p* < 0.05 was considered statistically significant.

## Results

### MSCs subcutaneous injection may alleviate the IMQ-induced psoriatic mice

**Fig. 1:**
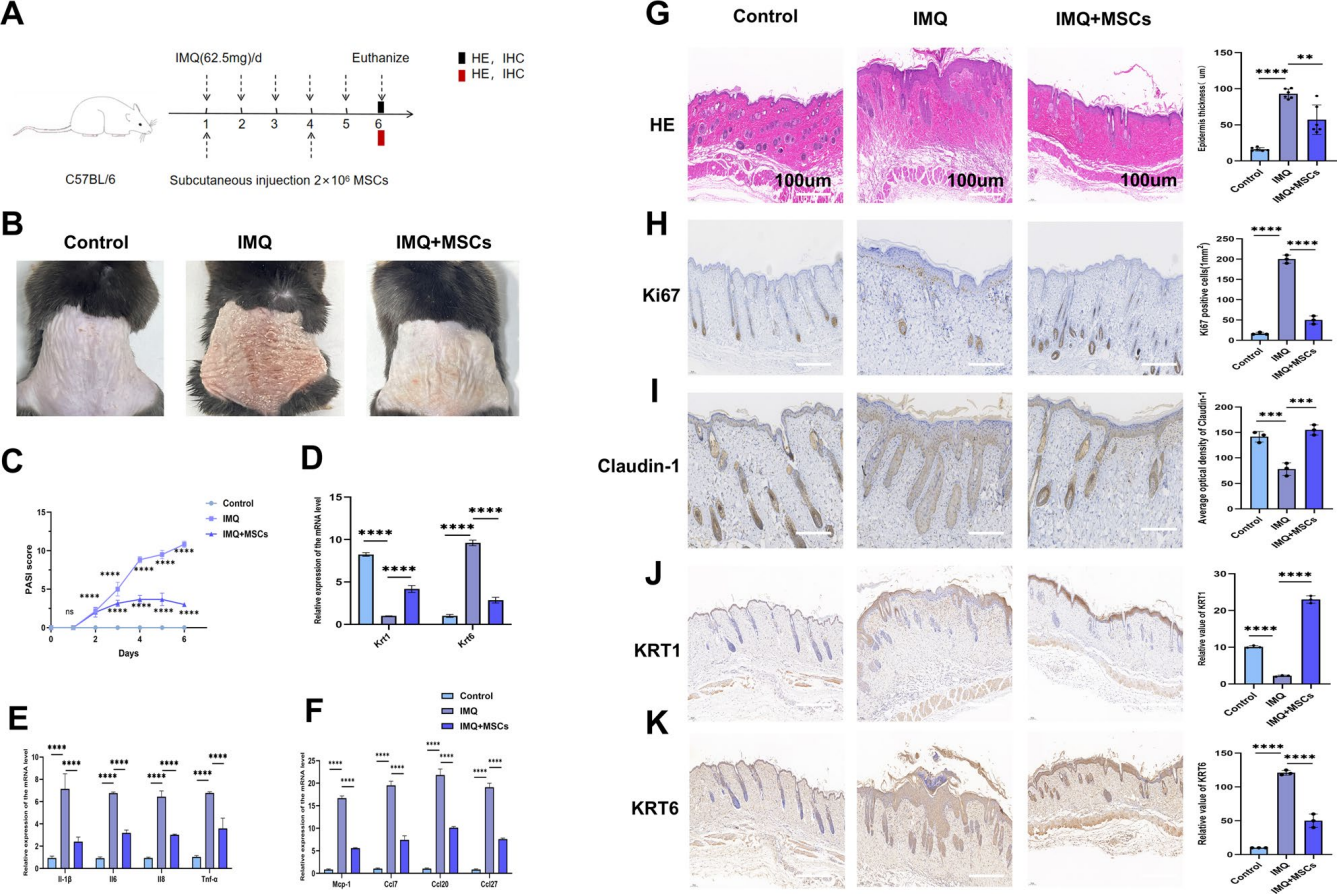
MSCs alleviated IMQ-induced psoriatic-like lesion. **A** Schematic diagram of subcutaneous administration in mice (MSCs 2 × 10^6^ cells, 200 μl PBS cell suspension). **B** Phenotypical presentation of the mouse dorsal back skin represented by gross images, n = 6. **C** Psoriasis Area and Severity Index (PASI) scores in mice, n = 6. **D**–**F** qRT-PCR analysis of KRT1, KRT6, levels of inflammatory factors, and chemokines in different groups, n = 3. **G** HE staining of the lesion in 3 groups, scale bar 100 μm, n = 6. **H**–**K** The Ki67, claudin-1, KRT1, KRT6 immunostaining of skin in the indicated groups, n = 3, scale bar: 100 μm. (**P< 0.01, *** P< 0.001, ****P < 0.0001)

To understand how MSCs subcutaneous injection affected psoriasis, we applied IMQ to the mice every day for six days. MSCs were injected subcutaneously on days 1 and 4 after IMQ treatment (Fig. 1A). The mice's dorsal skin showed symptoms like erythema, thickening, and scaling, along with inflammation. These symptoms worsened until the IMQ treatment ended on day 6. The Psoriasis Area and Severity Index (PASI) scores also increased. Since the IMQ-induced psoriasis model can self-heal, we recorded observations on day 6, when the symptoms peaked. The mice were euthanized for histological and immunohistochemistry analysis on this day. Compared with mice treated with IMQ alone, those receiving MSCs therapy demonstrated significant reductions in erythema, thickening, scaling, and PASI scores. (Fig. 1B, C). The epidermal thickness of psoriasis lesion decreased significantly after MSCs treatment. HE staining revealed a significant reduction in epidermal proliferation after MSCs treatment (Fig. 1G). The levels of key skin proliferation proteins Ki67 and KRT6 also decreased significantly (Fig.H–K). Conversely, KRT1, a protein in normal skin, increased after MSCs treatment (Fig. 1J). These results suggested that MSCs can help balance KCs proliferation and differentiation. Claudin-1, a marker of skin barrier function, showed significant improvement following MSCs therapy (Fig. 1I). From our results, the mRNA levels of KRT1, KRT6 were consistent with the immunohistochemical findings in mice (Fig. 1D). Furthermore, mRNA levels of the inflammatory cytokines IL-1β, IL-6, IL-8, and TNF-α were reduced in mice skins following MSCs administration (Fig. 1E). Concurrently, levels of chemokines including CCL7, CCL20, CCL27, and MCP-1 were also diminished (Fig. 1F). In summary, our data indicated that MSCs can alleviate skin lesion in IMQ-induced mice, with the potential mechanism likely mediated through reducing the release of core psoriatic inflammatory cytokines and chemokines.

### MSCs can attenuate M5-induced hyperproliferation, inflammatory cytokines and chemokines expression in HaCaT KCs

**Fig. 2:**
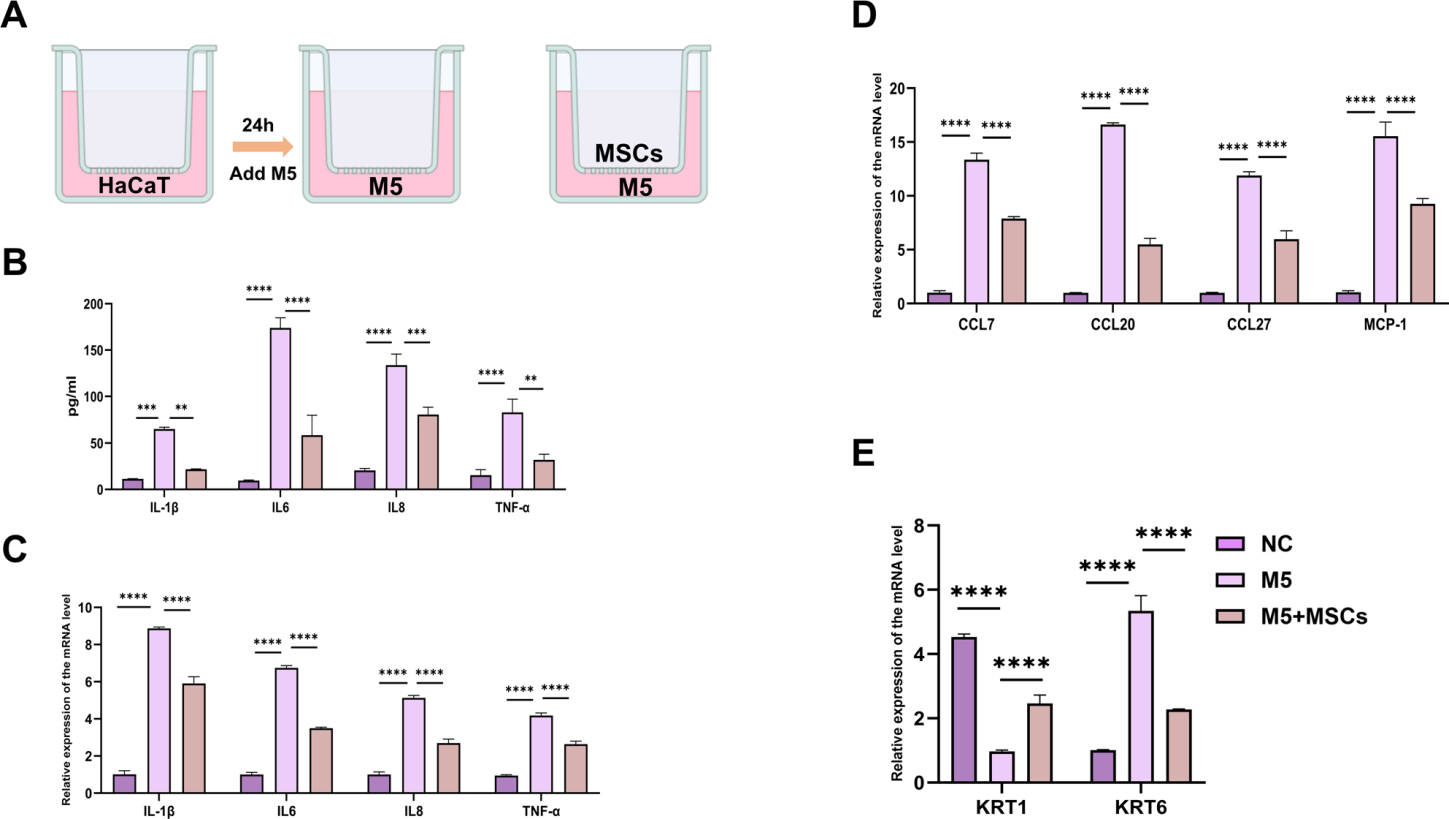
MSCs reduced M5- induced psoriatic model. **A** A schematic diagram showing the co-culture of M5 and MSCs, n = 3. **B** ELISA analysis was performed to evaluate the expression of IL-1β, IL-6, IL-8, and TNF-α in the supernatants from the NC, M5, and M5 + MSCs groups, n = 3. **C**–**E** qRT-PCR was performed to assess the mRNA levels of inflammatory factors, chemokines, KRT1 and KRT6 in the various groups, n = 3. (**P< 0.01, *** P < 0.001, **** P < 0.0001)

Given that KCs are key players in the pathological lesion of psoriasis, we investigated the effects of MSCs on pathological KCs. A mixture of IL-17A, IL-22, oncostatin M, IL-1α, and TNF-α, termed M5 (each at 10 ng/ml), was used to stimulate HaCaT KCs to recapitulate some features of psoriasis in vitro (Fig. 2A). The methodology for constructing the M5 model was detailed in the methods section. The mRNA levels of M5-induced inflammatory cytokines (IL-1β, IL-6, IL-8, TNF-α) and chemokines (MCP-1, CCL7, CCL20, CCL27) were significantly reduced following 24-h co-culture of MSCs (Fig. 2C, D).The downregulation of KRT6 coupled with the synchronous upregulation of KRT1 indicated that MSCs could promote the differentiation and inhibit proliferation in the M5 model (Fig. 2B). ELISA results further confirmed that co-culture with MSCs significantly reduced M5-induced IL-1β, IL-6, IL-8, and TNF-α protein levels (Fig. 2E). To summarize, our results indicated that MSCs were able to attenuate M5-induced excessive inflammatory response in HaCaT KCs, consistenting with the experiments results conducted in vivo.

### PSPH may play a pivotal role in the serine metabolism

**Fig. 3:**
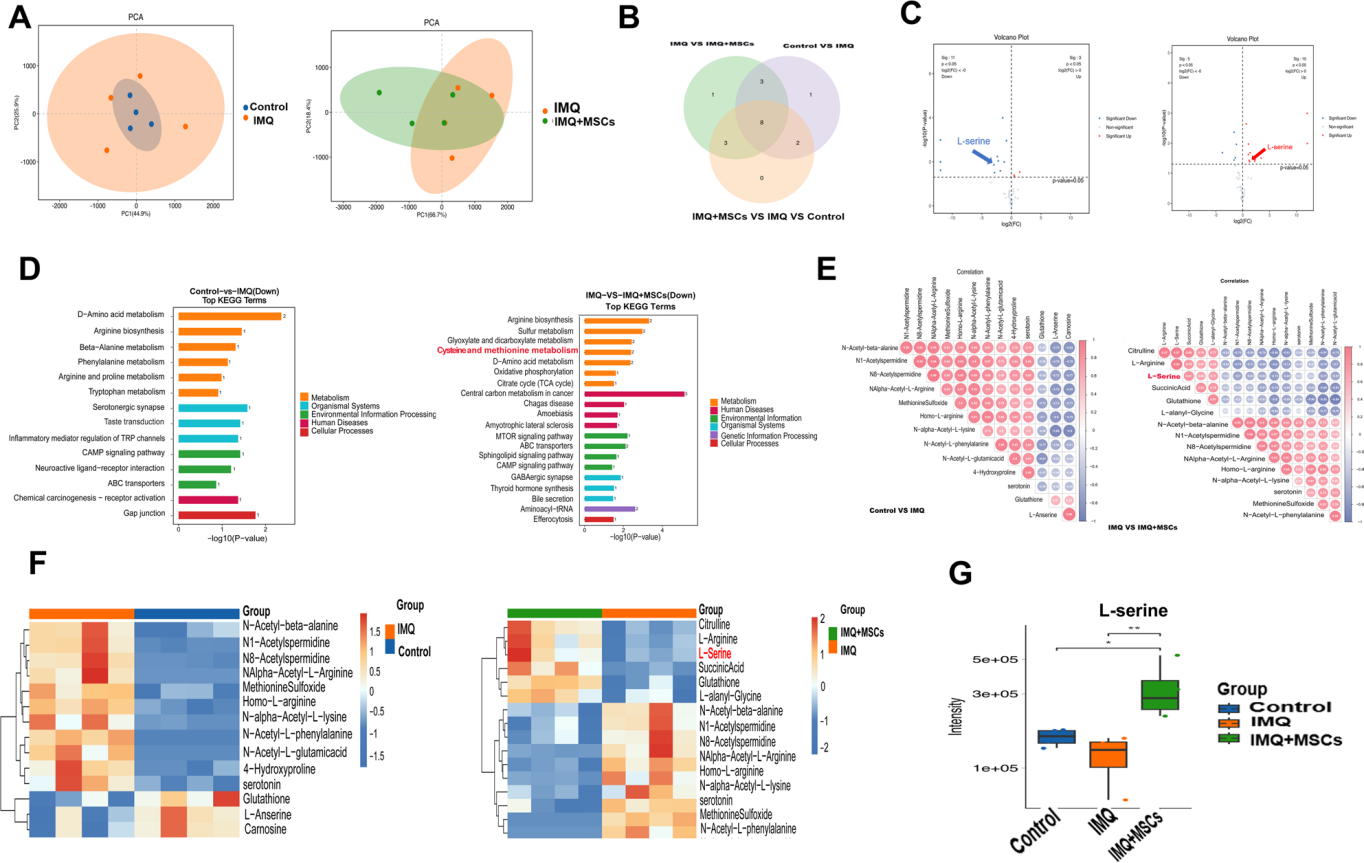
L-serien metabolomics of the Control, IMQ, and IMQ + MSCs groups. (**A**) PCA plots illustrated the distribution of data points from the Control, IMQ, and IMQ + MSCs groups (n = 4). (**B**) Venn diagrams displayed. (**C**) Volcano plots showed significantly different metabolites between the Control and IMQ groups, as well as between the IMQ and MSCs groups (*p* < 0.05). (**D**) KEGG pathways from the Control, IMQ, and IMQ + MSCs groups were shown. (**E**) The correlation maps displayed the levels of correlation between Control, IMQ, and IMQ + MSCs subjects. (**F**) Heatmaps of the Control, IMQ, and IMQ + MSCs groups were shown. (**G**) The abundance of serine in the Control, IMQ, and IMQ + MSCs groups was shown (*P < 0.05, ** P< 0.01)

Research has shown that MSCs can regulate macrophage polarization through the IDO-dependent Kyn – AhR – NRF2 pathway, alleviating ligation-induced periodontitis [32]. This suggested that MSCs can improve inflammation through immune metabolism. Therefore, we hypothesized that there may be changes in amino acid metabolism in psoriasis lesion following MSCs treatment. To explore the link between psoriasis and amino acid metabolism, we performed targeted amino acid metabolomics analysis of the skin from Control, IMQ, and IMQ + MSCs mice. PCA analysis showed significant differences among the three groups (Fig. 3A), indicating that IMQ-induced inflammation and MSCs intervention changed amino acid metabolism in the epidermis. The heatmap (Fig. 3F) showed the metabolic characteristics through hierarchical clustering analysis. Notably, after MSCs treatment, the levels of citrulline, arginine, and serine in the IMQ mice increased significantly (*p* < 0.05). Volcano plot analysis (Fig. 3C) identified differentially expressed metabolites between the control and IMQ groups, as well as between the IMQ and IMQ + MSCs groups. L-serine levels significantly decreased in the IMQ-treated mice (*p* < 0.05). KEGG pathway analysis confirmed that cysteine and methionine metabolism was significantly reduced in IMQ mice (Fig. 3D). Correlation analysis revealed that serine, a key amino acid in cysteine and methionine pathways, also decreased significantly in IMQ mice (Fig. 3E). The heatmap showed that, compared to the normal group, serine levels significantly increased after MSCs treatment in IMQ mice (Fig. 3F). Boxplot results indicated that serine abundance in the epidermis of IMQ mice increased after MSCs treatment (Fig. 3G). These findings suggested that IMQ-induced inflammation disrupted cysteine and methionine metabolism, with a decrease in serine levels further contributing to these pathological changes. MSCs may alleviate disease pathology by modulating cysteine and methionine metabolism, potentially through the upregulation of serine. As PSPH was crucial in serine synthesis, we hypothesized that a significant relationship may exist among MSCs and PSPH.

### Knockdown of PSPH can reverse the therapeutic effect of MSCs on the M5-induced model

**Fig. 4:**
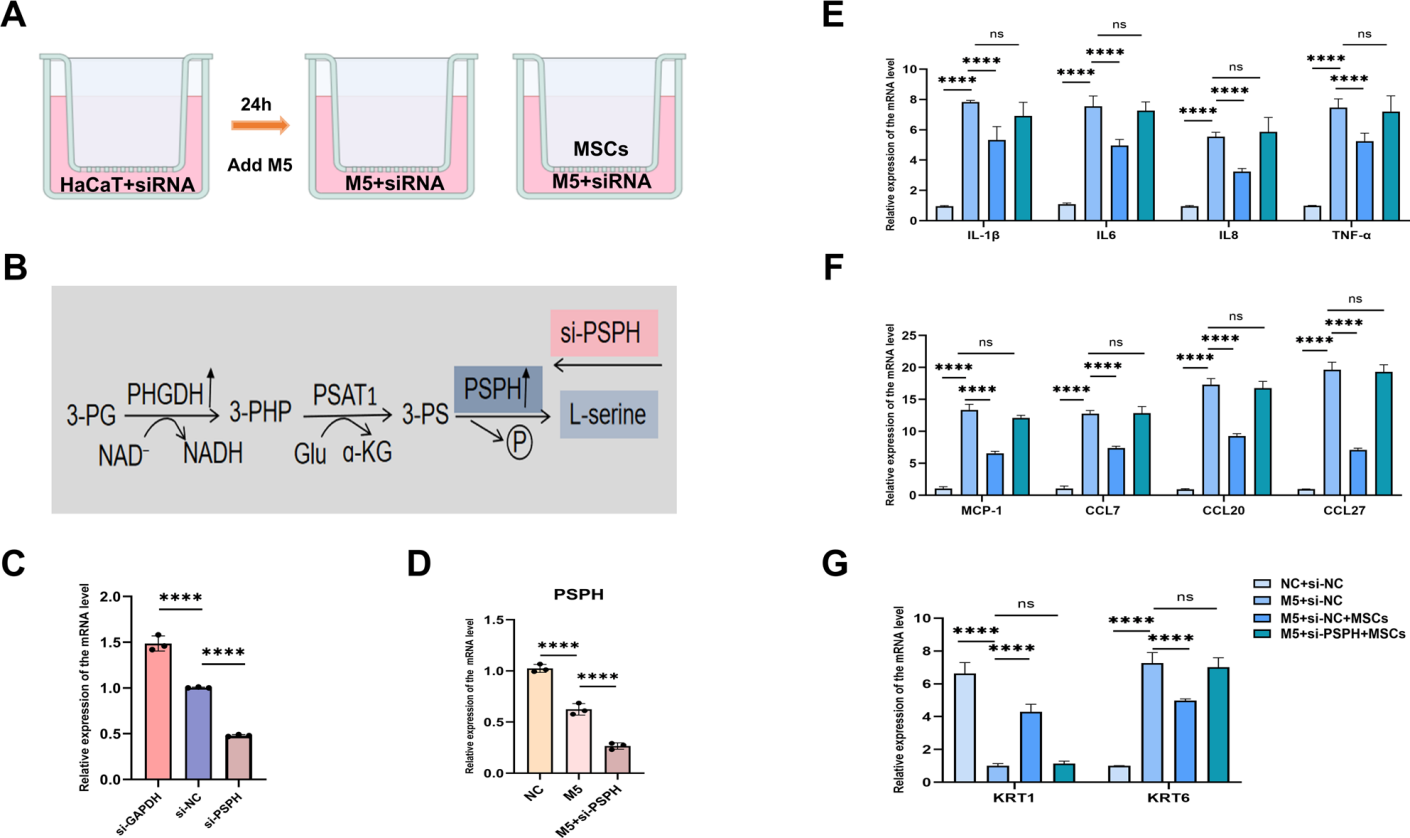
Interfering with PSPH expression using siRNA reversed the MSCs treatment phenotype in the M5- induced model. **A** HaCaT cells were transfected with siRNA (1 nM) or an empty vector and cultured with MSCs for 24 h, n = 3. **B** Schematic representation of serine synthesis. **C** Diagram illustrated the efficiency of siRNA. **D** PSPH was validated in the three groups, n = 3. **E**–**G** qRT-PCR evaluated the mRNA expression levels of inflammatory factors, chemokines,KRT1 and KRT6 in NC + si-NC, M5 + si-NC, M5 + si-NC + MSCs, and M5 + si-PSPH + MSCs, n = 3.siRNA, small interfering RNA. (* P < 0.05, ** P< 0.001, *** P < 0.001, **** P < 0.0001, ns: not significant)

To assess the functional consequences of PSPH loss, we employed siRNA-mediated knockdown in HaCaT cells. Detection of mRNA revealed that knocking down PSPH reversed the psoriatic phenotype induced by MSCs treatment. Notably, There were no differences in IL-1β, IL-6, IL-8, TNF-α, MCP-1, CCL7, CCL20, CCL27, KRT1 and KRT6 following co-culture with MSCs after knockdown of PSPH in M5-induced KCs (Fig. 4E-G). In conclusion, these data suggested that PSPH may be a specific response gene to MSCs treatment in M5 psoriatic model.

### MSCs may activate the PINK1/Parkin pathway in vivo and in vitro

**Fig. 5:**
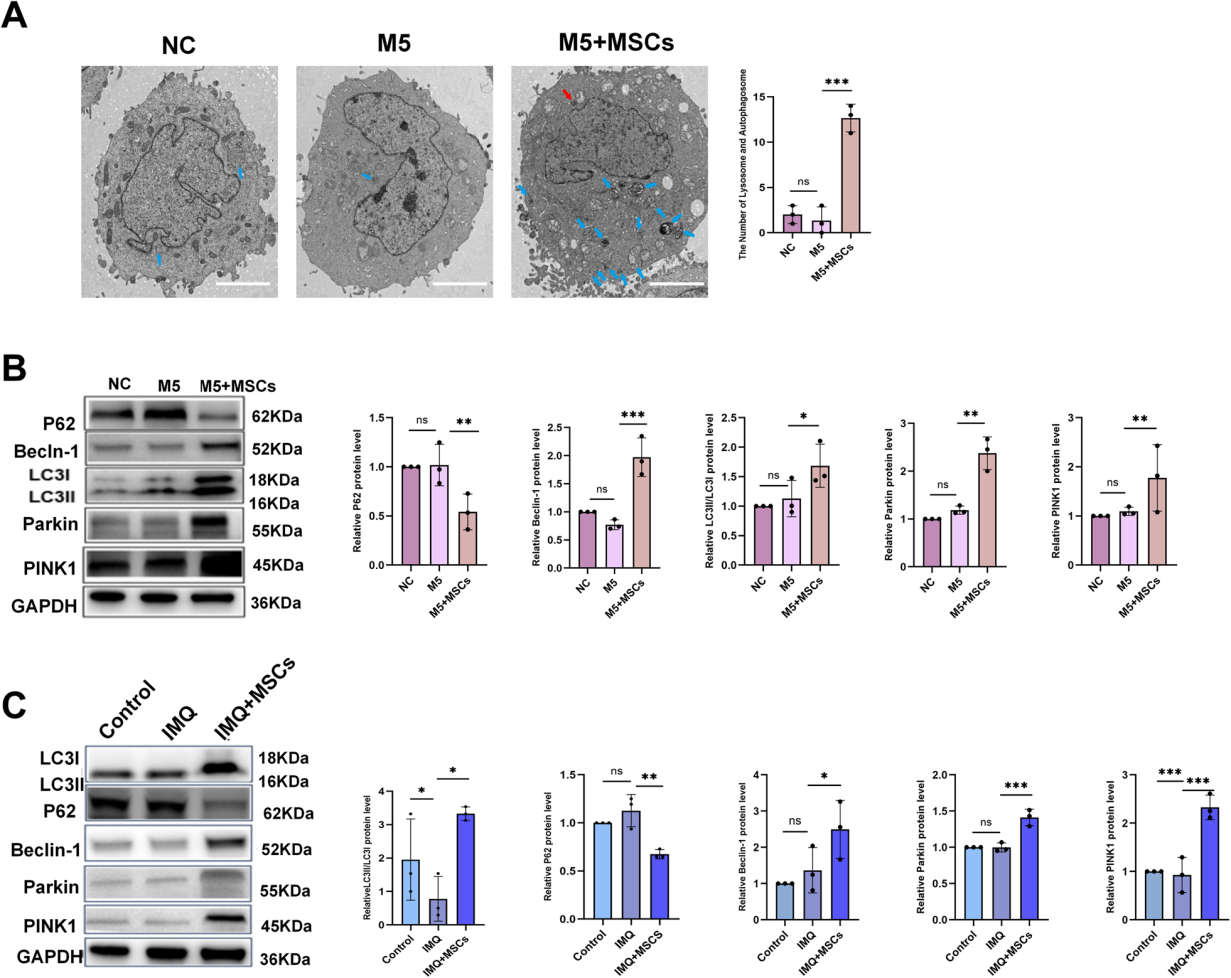
MSCs activated the PINK1-Parkin pathway both in vitro and in vivo. **A** Mitochondrial autophagy (mitophagy) observed under a transmission electron microscopy in three groups in vitro (red arrows indicated mitochondrial autophagy, blue arrows indicated lysosomes; scale bar: 2 μm, magnification: 3000x, high contrast mode, 80 kV), n = 3. **B**, **C** WB results displayed the expression levels of PINK1, Parkin, p-Parkin, Beclin-1, LC3B, P62, and statistical results in vitro and in vivo, n = 3. (*P < 0.05, **P< 0.001, *** P < 0.001, ns: not significant)

It has been reported that MSCs performed a regulatory effect on autophagy in HaCaT KCs [36]. As mitophagy was a central paradigm of autophagic clearance, we postulated that MSCs were capable of inducing mitophagy in KCs. We performed transmission electron microscopy for observing mitochondrial autophagy, as transmission electron microscopy served as the gold standard. Transmission electron microscopy revealed minimal mitochondrial autophagy in both the NC and M5 groups, likely due to the use of a starvation-based model. Since starvation is a physiological trigger known to regulate cellular autophagy, this may account for the limited mitophagy observed. In the presence of MSCs, the M5 group exhibited a notable increase in autophagosomes and lysosomes, suggesting enhanced mitophagy compared to the other two groups (Fig. 5A). Afterward, the expression levels of proteins associated with mitophagy were assessed in both in vitro and in vivo experiments. According to the protein expression levels, in vitro results indicated that upon exposure to MSCs, M5 KCs exhibited increased levels of mitophagy-related proteins (PINK1, Parkin, LC3B-II/I, and Beclin-1); in contrast, the autophagy substrate P62 decreased (Fig. 5B). The administration of MSCs triggered an increase in PINK1, Parkin, p-Parkin, LC3B-II/I, and Beclin-1, alongside a reduction of P62 in the skin of psoriatic mice (Fig. 5C). Collectively, the experimental data linked the efficacy of MSCs in psoriasis to the activation of the PINK1-Parkin mitophagy pathway.

### MSCs can inhibit NLRP3 inflammasome activation in vitro and in vivo

**Fig. 6:**
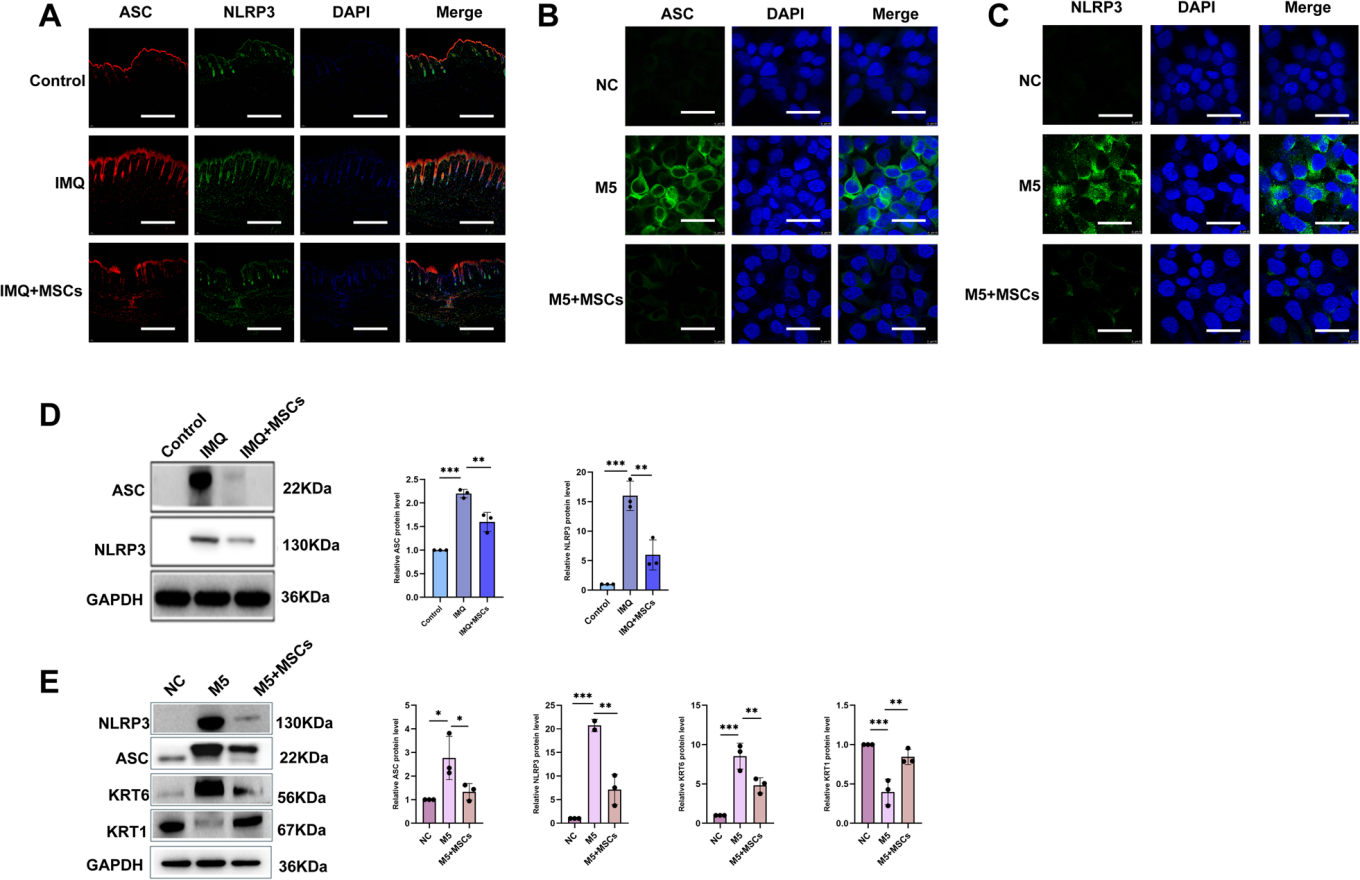
MSCs may negatively regulate NLRP3 inflammasome mediated inflammatory cytokine production in vivo and in vitro. **A** Immunofluorescence double staining results represented in mice, with ASC labeled in red and NLRP3 in green; scale bar: 25 μm. Blue: DAPI DAPI, n = 3. **B** ASC immunofluorescence single staining. Green: ASC; blue: DAPI. Scale bar: 10 μm, n = 3. **C** NLRP3 immunofluorescence single staining. green: NLRP3; blue: DAPI. Scale bar: 10 μm. n = 3. **D**, **E** WB images and statistical results in vivo and in vitro, n = 3. (*P< 0.05, ** P< 0.001, *** P < 0.001)

NLRP3 acts as a “sensor” detecting danger signals, whilst ASC serves as the “connector” receiving its signals and relaying them downstream. Upon activation, NLRP3 recruits ASC, which then oligomerises to form a complex, activating the downstream Caspase-1. Together, they assemble into a functional NLRP3 inflammasome [36]. Driven by the potential of MSCs to modulate innate immunity, we interrogated their effect on the NLRP3 inflammasome. Of particular note, the IMQ-induced model was characterized by NLRP3 inflammasome activation. The inhibitory effect of MSCs on ASC and NLRP3 activation was evident from immunofluorescence co-staining results, showing pronounced suppression (Fig. 6A). In line with the results, WB analysis consistently demonstrated a reduction in ASC and NLRP3 protein levels in the wake of MSCs administration (Fig. 6D). To further elucidate the effects of MSCs on NLRP3 inflammasome activation, we extended our study to an M5 model. Immunofluorescence results demonstrated that MSCs treatment suppressed NLRP3 activation in M5-induced HaCaT cells (Fig. 6B, C), consistent with prior in vitro data.WB analysis further confirmed that co-culture MSCs with M5-stimulated KCs led to a marked reduction in both ASC and NLRP3 protein expression (Fig. 6E). In vitro analyses further revealed an upregulation of KRT1 and a concomitant downregulation of KRT6 in M5 KCs upon MSCs co-culture, thereby corroborating our earlier immunohistochemical results in IMQ-induced murine models. Together, these results showed that the inhibition of NLRP3 inflammasome activation by MSCs may contribute to the alleviation of psoriasis.

### Inhibiting the PINK1-Parkin pathway can restore NLRP3 expression and reverse the therapeutic effects of MSCs

**Fig. 7:**
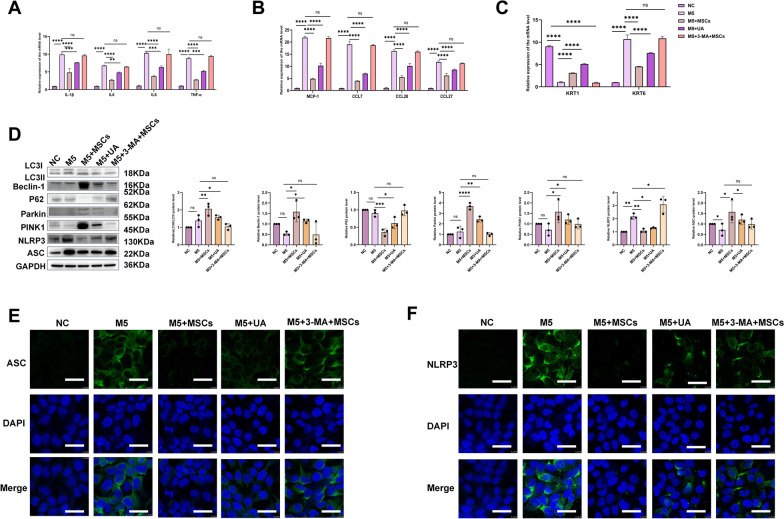
MSCs can enhance the PINK1-Parkin pathway to inhibit the formation of NLRP3 inflammasomes in an M5- induced model. **A**–**C** qRT-PCR analysis of the mRNA levels of inflammatory factors, chemokines, KRT1, and KRT6, n = 3. **D** Representative WB images and statistical results are shown, n = 3. HaCaT cells were treated with the mitochondrial autophagy activator urolithin A (UA) (10 µM) for four hours or HaCaT cells were incubated with 3-methyladenine (3-MA, 5 μg/ ml) for 24 h during MSCs treatment, n = 3. **E** Immunofluorescence results showed the expression of ASC, with inflammation marked in green; blue: DAPI. Scale bar: 10 µm. n = 3. **F** Immunofluorescence results showed the expression of NLRP3, with inflammation marked in green; blue: DAPI. Scale bar: 10 µm,n = 3 (*P< 0.05, ** P < 0.001, *** P < 0.001)

To further investigate the functional link between the PINK1–Parkin pathway and inflammation in psoriatic models in vitro, we employed the autophagy inhibitor 3-methyladenine (3-MA) to suppress mitophagy in the M5 + MSCs group. Conversely, the mitochondrial autophagy activator urolithin A(UA) was introduced to the M5 group. Strikingly, inhibition of mitophagy via 3-MA largely abolished the anti-inflammatory effects conferred by MSCs. Specifically, 3-MA significantly reversed the suppressive effects of MSCs on the expression of pro-inflammatory cytokines (IL-1β, IL-6, IL-8, TNF-α), chemokines (MCP-1, CCL7, CCL20, CCL27), and keratinocyte differentiation markers (KRT1 and KRT6) (Fig. 7A–C). In contrast, UA treatment markedly ameliorated both hyperproliferation and inflammatory responses in M5-induced KCs. Importantly, immunofluorescence results indicated that UA can moderately reduce the fluorescence intensity of ASC and NLRP3 in M5 models. However, the immunofluorescence therapeutic effect of M5 + MSCs + 3MA was markedly inferior to that of M5 + MSCs alone. The therapeutic outcome observed in the UA group exhibited an intermediate efficacy profile relative to the other two treatment conditions (Fig. 7E, F). Additionally, the expression of ASC and NLRP3 showed highly consistent changes, synchronised with that of PINK1-Parkin-associated key proteins. M5 + UA partially reduced ASC and NLRP3, indicating that mitochondrial autophagy inherently possesses the capacity to inhibit NLRP3 activation. However, upon adding 3MA to M5 + MSC, the MSCs lost their ability to downregulate ASC and NLRP3 (Fig. 7D). Through functional validation, our results suggested a central role for MSCs in activating the PINK1–Parkin mitophagy pathway to suppress NLRP3 inflammasome activation.

### The activation of PINK1-Parkin may depend on PSPH in HaCaT

**Fig. 8:**
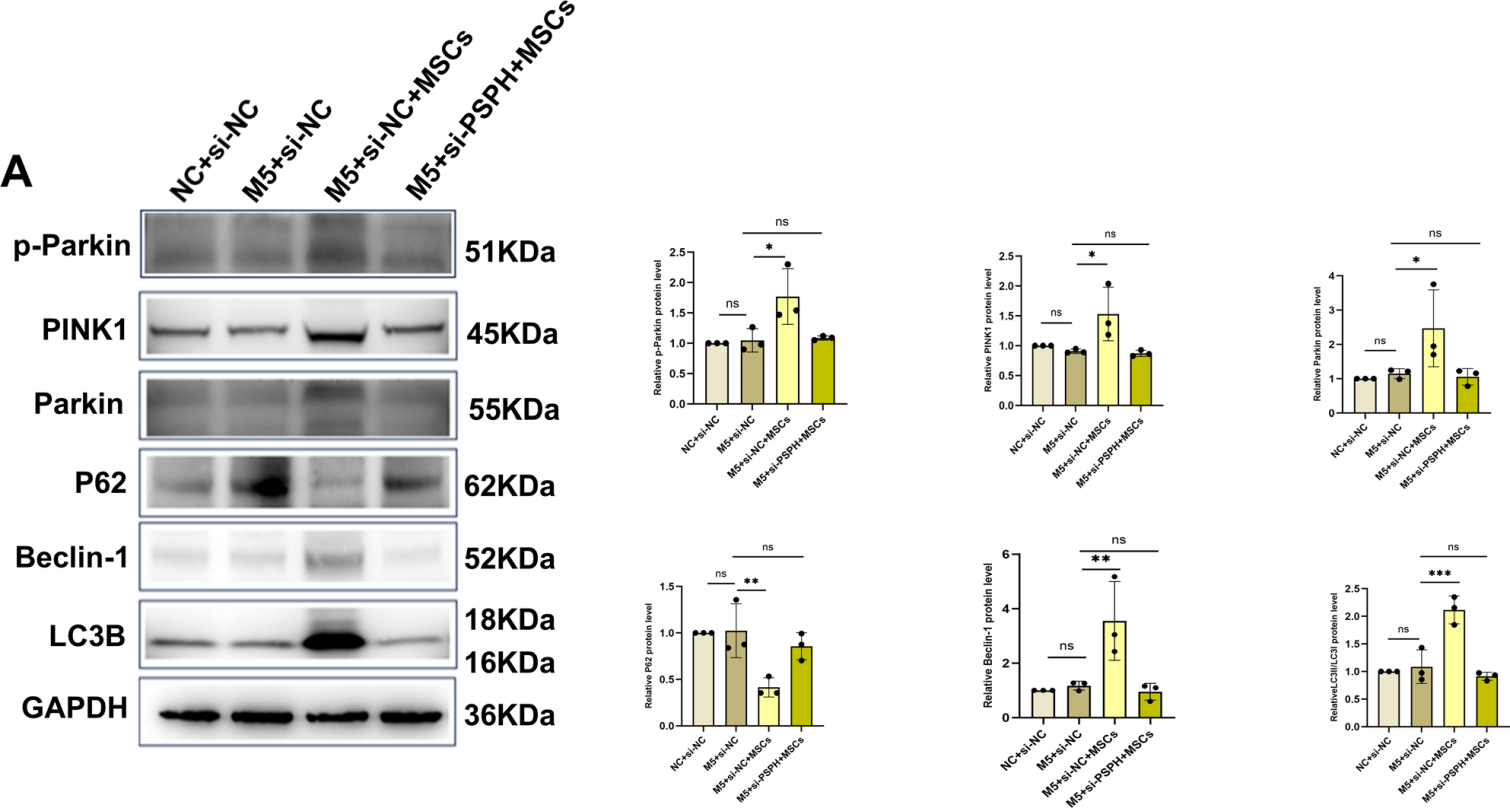
The activation of PINK1-Parkin in HaCaT may depend on PSPH. **A** WB and statistical results, n = 3. (*P< 0.05, ** P < 0.001, *** P < 0.001, ns: not significant)

We have verified that PSPH can affect the therapeutic effects of MSCs, so we speculated that PSPH can regulate the progression of M5 HaCaT cells by activating the PINK1-Parkin signaling pathway. To further investigate the upstream regulatory mechanisms of PINK1-Parkin, a knockdown experiment on the PSPH was conducted. The results showed that the silencing of PSPH can reverse the activation of the PINK1-Parkin pathway, thereby counteracting the anti-inflammatory effects of MSCs treatment. Overall, our study indicated that PSPH can enhance the autophagic capacity of M5 mitochondria to alleviate the pathological processes associated with psoriasis (Fig. 8A).

## Discussion

Previous studies have focused on the direct effects of paracrine secretion of MSCs on disease [37, 38]. In contrast, our study focused on changes in psoriasis after MSCs treatment. KCs, as resident skin cells, play a dual role as participants and victims of psoriasis. Our results suggest that MSCs can decrease inflammatory factors and chemokines in psoriatic skin, inhibiting epidermal hyperplasia and promoting epidermal differentiation. This study verified that PSPH may be a key gene involved in serine metabolism in psoriasis. Silencing PSPH through RNA interference resulted in a significant decrease in PINK1-Parkin activation and an increase in inflammatory cytokine production and cell proliferation (as well as KRT6 expression) in KCs. Inhibition of the PINK1-Parkin pathway reversed the inhibitory effect of MSCs on the NLRP3 phenotype. Altogether, these results reveal a novel mechanism of action for MSCs in psoriasis-induced HaCaT KCs.

Compared with conventional psoriasis treatments, MSCs therapy demonstrates multiple potential advantages. While traditional topical therapies and systemic immunosuppressants can control symptoms, they present limitations in efficacy, high recurrence rates, and long-term safety concerns such as hepatic and renal toxicity [39]. Biological agents, whilst highly targeted, typically address only a single inflammatory pathway (such as TNF-α, IL-17/23). Some patients may experience loss of response or an increased risk of infection [40, 41]. In contrast, MSC therapy exhibits multi-targeted, systemic regulatory properties: not only does it synergistically inhibit multiple inflammatory pathways and enhance the function of regulatory immune cells through paracrine effects, but it may also directly participate in tissue repair [42, 43]. Moreover, its mechanism of action encompasses dual regulation of both immune imbalance and metabolic disorders, a feature rarely observed in conventional therapies [44, 45]. For patients with refractory, recurrent psoriasis or those with concomitant metabolic abnormalities, MSCs therapy can play as a complementary or alternative option [46].

Serine metabolism, as a key regulator of cellular function, is increasingly recognized for its pivotal role in diverse pathophysiological processes. It is known that KCs have been demonstrated to be serine/glycine nutrient-deficient cells, relying on serine hydroxy methyltransferase (SHMT) to convert serine into glycine and one-carbon units to support cellular growth and proliferation. One study showed inhibition of SHMT activity may lead to restricted nucleotide synthesis, thereby concurrently suppressing both excessive KCs proliferation and inflammatory responses. Animal studies further confirmed that local inhibition of SHMT effectively can alleviate psoriasiform skin inflammation. Collectively, these findings indicated that serine represented a pivotal metabolic node regulating abnormal proliferation of epidermal cells and inflammatory expansion in psoriasis [47]. A separate line of investigation has elucidated the critical involvement of serine metabolic dysregulation in driving psoriatic inflammation. Clinical samples demonstrated that phosphoglycerate dehydrogenase (PHGDH), a key enzyme in serine synthesis, exhibited downregulated expression in psoriatic lesions. Inhibition of PHGDH in KCs promoted the secretion of pro-inflammatory factors including IL-6, TNF-α, IL-17, and NF-κB. Mechanistically, PHGDH inhibition reduces DNA methylation levels in the IL-6 gene promoter region by decreasing the methyl donor S-adenosylmethionine (SAM), thereby promoting transcription factor MEF2A binding and upregulating its expression. Concurrently, it enhances activation of the NF-κB pathway. Ultimately, in a mouse psoriasis model induced by IMQ, supplementation with SAM effectively alleviated skin inflammation. These findings suggest that targeting the PHGDH-SAM axis represents a novel therapeutic strategy for psoriasis [48].In our study, serine metabolism was effective after MSCs therapy in IMQ mice, which was consistent with previous study.

Notably, the maintenance of serine homeostasis is equally crucial for cellular survival and function. Correspondingly, research has revealed that human umbilical cord stem cell-derived exosomes (hUSC-Exo) are rich in miR-27b-3p, which targets and suppresses the expression of the serine transporter SLC1A4 in granulosa cells. This reduces serine efflux, maintains intracellular serine concentration, activates the PI3K/AKT/mTOR pathway, and ultimately inhibits cyclophosphamide-induced granulosa cell apoptosis and premature ovarian failure [49]. Moreover, the attenuation of serine metabolism has been demonstrated to be a key factor driving cellular senescence. The regenerative potential of dentine-pulp-derived mesenchymal stem cells (DPSCs) diminishes significantly with advancing age, a phenomenon closely linked to the underlying mechanism of reduced intracellular serine metabolism. In aged DPSCs, downregulation of the key serine synthesis enzymes PSAT1 and PHGDH leads to insufficient production of the methyl donor S-adenosylmethionine (SAM). This, in turn, causes hypomethylation and upregulation of the p16 promoter region, ultimately driving cellular senescence. This discovery elucidates the central role of serine metabolism in maintaining MSC function [50]. Collectively, this evidence may point out a pathogenic axis: disruption of serine metabolism—whether through impaired synthesis, increased efflux, or overall depletion—can ultimately trigger tissue dysfunction by perturbing inflammatory balance, apoptotic signaling, or epigenetic homeostasis. The evidence may underscore the significant role of serine metabolism in psoriasis.

PSPH, as the terminal key enzyme in the serine biosynthesis pathway, is responsible for dephosphorylating phosphatidylserine to yield serine [51]. Therefore, we assumed that a high level of PSPH may elevate serine level. Elevated levels of PSPH may activate the PINK1-Parkin-NLRP3 pathway, lowering the burden of inflammatory factors in psoriasis. To our knowledge, research on PSPH in psoriasis remains largely unexplored, with its specific expression patterns and cell-specific functions.

The PINK1/Parkin pathway is a key mediator of mitophagy [52]. The progression of psoriasis may impaire mitophagy and downregulate the PINK1/Parkin pathway [53, 54]. Our results demonstrated that MSCs can enhance PINK1/Parkin mitophagy in the M5 model. The functional recovery experiments in this study indicated that the PINK1-Parkin pathway may be involved in psoriasis progression. Activation of PINK1-Parkin pathway may reduce NLRP3 inflammasome activation and decrease release of downstream inflammatory mediators both in vivo and in vitro. These observations further corroborated previous research results [55]. Additionally, a study reported that in contrast-induced acute kidney injury, PINK1-Parkin-mediated mitophagy can suppress NLRP3 inflammasome activation [56]. The conclusions were consistent with the findings of our study.

The clinical application prospects of MSCs in treating psoriasis are promising. Regarding treatment timing, MSCs may be more suitable for moderate-to-severe, active-phase patients who respond poorly to conventional therapies, or as an early intervention to regulate immune imbalance and prevent chronic progression [57]. The phase 1/2a clinical trial demonstrated that treatment with clinical-grade umbilical cord-derived mesenchymal stem cells, administered as a single infusion at a dose of 1–3 × 10⁶ cells per kilogram of body weight, is safe and partially effective in patients with psoriasis [58]. Due to the limitation of the small sample size, current evidence suggests that MSC therapy for psoriasis is safe and effective with no apparent significant adverse effects [59]. However, long-term safety warrants close monitoring, encompassing potential hypersensitivity reactions, abnormal tissue proliferation, risk of pulmonary embolism (particularly following intravenous administration), and heightened susceptibility to infection or potential pro-tumour microenvironment formation due to their immunomodulatory function [60, 61]. Therefore, future clinical translation must incorporate individualised assessment and establish a long-term follow-up system to comprehensively weigh its efficacy against risks.

## Conclusion

In summary, adipose-derived MSCs hold great promise as a novel therapeutic tool in the treatment of psoriasis. The results of our study provided evidence that PSPH may act as a weight in MSCs therapy for IMQ-induced mouse psoriasis. This study showed that MSCs can alleviate IMQ-induced psoriasis by activating the PINK1-Parkin mitophagy pathway through upregulating the PSPH in HaCaT KCs. This cascade may culminate in PSPH-PINK1-Parkin mediated activation of mitophagy, concurrent attenuation of NLRP3 inflammasome activation, and restraint of psoriatic KCs hyperproliferation. Notwithstanding these results, the current study acknowledged certain limitations. Chief among these was the need to identify the specific soluble factors released by MSCs that were primarily responsible for PSPH upregulation in HaCaT KCs. Moreover, clinical validation, which would be expected to substantiate the relevance of our results, was not incorporated into this investigation. These gaps will be addressed in our future research. Meanwhile, our preliminary findings suggest that mesenchymal stem cells (MSCs) may be associated with PSPH, a key enzyme in serine metabolism in mouse psoriasis. And our forthcoming research will further elucidate the regulatory role of specific components within the MSCs secretome—such as cytokines or exosomes—on PSPH expression in psoriatic KCs. Unravelling this regulatory axis may reveal the further mechanisms by which MSCs paracrine signaling modulates psoriatic inflammatory responses, thereby providing a rationale for developing targeted therapies about PSPH.

## Supplementary Information

Additional file 1.

Additional file 2.

Additional file 3.

Additional file 4.

Additional file 5.

## Data Availability

All datasets of this article are included within the article.
