# Supplementary material for: Research based on serine metabolism indicates mesenchymal stem cells alleviate psoriasis by regulating the PSPH-PINK1-Parkin-NLRP3 pathway in HaCaT
Source: Stem Cell Res Ther. 2026 Mar 28;17:176. doi: 10.1186/s13287-026-04964-z (PMC13151198; doi:10.1186/s13287-026-04964-z)
Supplement: Supplementary file 1 — Additional file 1. [file 13287_2026_4964_MOESM1_ESM.pdf]

The ARRIVE guidelines 2.0

1.Study design 1 For each experiment, provide brief details of study design including: a. The groups being compared, including control groups. If no control group has been used, the rationale should be stated. b. The experimental unit (e.g. a single animal, litter, or cage of animals).

Response:a.The experiment comprised a blank control group, an IMQ-induced psoriasis mouse disease group, and an IMQ-induced psoriasis mouse model group treated with MSCs. b.Mice in each group were purchased from the same batch and housed individually.

2. Sample size 2 a. Specify the exact number of experimental units allocated to each group, and the total number in each experiment. Also indicate the total number of animals used. b. Explain how the sample size was decided. Provide details of any a priori sample size calculation, if done.

Response:The sample size of six mice per group was determined based on previous similar studies [Shen H, Sha Y, Huang J, et al. The roles of AMPK-mediated autophagy and mitochondrial autophagy in a mouse model of imiquimod-induced psoriasis. Am J Transl Res. 2021;13(11):12626-12637.] and Follow the '3Rs' principle to minimize animal usage while ensuring sufficient statistical power to detect significant differences.

3.Inclusion and exclusion criteria 3 a. Describe any criteria used for including and excluding animals (or experimental units) during the experiment, and data points during the analysis. Specify if these criteria were established a priori. If no criteria were set, state this explicitly. b. For each experimental group, report any animals, experimental units or data points not included in the analysis and explain why. If there were no exclusions, state so. c. For each analysis, report the exact value of n in each experimental group.

Response: a. Animal inclusion criteria were predefined based on species (C57BL/6), age (8 weeks), and body weight (approximately 20 grams). Exclusion criteria were defined prior to the experiment as: (1) death during the experiment; (2) failure to develop psoriasis-like lesions (e.g., cumulative PASI score < 4 after 3 consecutive days of IMQ application); (3) Severe distress exceeding humane endpoints (e.g., >20% body weight loss, paralysis, or inability to access food and water). For data analysis, Grubbs' test was used to assess outliers; values deviating more than 2 standard deviations from the mean were excluded.

b. No animal deaths or predefined exclusion criteria were observed during the study.

All animals successfully developed psoriasiform lesions and were included in the final analysis. No data points were excluded during statistical procedures.

c. The final analysis included the following exact sample sizes (n) per group: Control (n = 6), IMQ (n = 6), IMQ + MSC (n = 6). These n values represent the actual number of animals used for subsequent analyses (including PASI scores and HE staining).

4.Randomisation a. State whether randomisation was used to allocate experimental units to control and treatment groups. If done, provide the method used to generate the randomisation sequence. b. Describe the strategy used to minimise potential confounders such as the order of treatments and measurements, or animal/cage location. If confounders were not controlled, state this explicitly.

Response: a. Animals were randomly assigned to experimental groups using a randomized method. Eighteen male C57BL/6 mice (8 weeks old, approximately 20 g) were randomly assigned to three groups (control, IMQ, and IMQ+MSC; n=6 per group) using a random number table generated by Microsoft Excel. The randomization sequence was generated prior to the start of the experiment.

b. To minimize potential confounding factors, we employed the following strategies: First, all cages were placed on the same rack under identical environmental conditions (temperature, humidity, light cycle) to eliminate location effects. Second, the sequence of treatments (e.g., IMQ application, MSC injection) and measurements (e.g., PASI scoring, sample collection) was randomized across groups using a block design to avoid time-dependent bias.

5 Blinding Describe who was aware of the group allocation at the different stages of the experiment (during the allocation, the conduct of the experiment, the outcome assessment, and the data analysis).

Response:To minimize bias, this study employed blinding wherever possible. Randomization sequences were generated by an independent researcher not involved in subsequent procedures. Group assignments were concealed from researchers performing animal handling (IMQ application and MSC injection) and outcome assessments through the use of coded cages and sample labels. All outcome measurements, including PASI scores, histological assessments, and biochemical tests, were performed by researchers unaware of group assignments. Data analysis was conducted by the corresponding author after unblinding.

6.Outcome measures a. Clearly define all outcome measures assessed (e.g. cell death, molecular markers, or behavioural changes). b. For hypothesis-testing studies, specify the primary outcome measure, i.e. the outcome measure that was used to determine the

sample size

Response:a. This study evaluated the following outcome measures:

Clinical Scoring: Daily assessment of psoriasiform dermatitis severity using a standardized scoring system, including erythema, scaling, and thickening (each item scored 0-4 points). The cumulative PASI (Psoriasis Area and Severity Index) score represents the sum of these three parameters.

Inflammatory Cytokines: Protein levels of pro-inflammatory cytokines interleukin (IL)-6, IL-8, IL-1β, and tumor necrosis factor-α (TNF-α) were quantified in skin tissue homogenates using commercial ELISA kits following manufacturer protocols.

Inflammasome and keratinocyte differentiation-related protein expression: Western blot analysis of skin tissue lysates assessed proteins involved in inflammasome activation (NLRP3, ASC), keratinocyte differentiation and hyperproliferation (keratin 1, KRT1; Keratin 6, KRT6), mitochondrial autophagy (PINK1, Parkin, and its phosphorylated form p-Parkin), and autophagy markers (Beclin-1, P62, and the LC3-II/LC3-I ratio). β-actin or GAPDH served as loading controls.

b. The primary outcome measure in this study was the cumulative PASI score on the final day of the experiment (day 6 after IMQ application). This metric was selected as the most direct and widely accepted indicator of psoriasiform skin inflammation severity in IMQ-induced psoriasis mouse models. The sample size (n=6 per group) was determined based on prior studies using PASI as the primary endpoint [Li J, Yan W, Yuan H, et al. Theacrine enhances autophagy and inhibits inflammation via regulating SIRT3/FOXO3a/Parkin pathway. Int J Rheum Dis. 2024;27(2):e15085.], ensuring consistency with existing literature and adherence to the 3Rs principle to minimize animal use. All other outcome measures, including cytokine levels and protein expression analyses, were considered secondary/exploratory endpoints to investigate potential mechanisms.

7. Statistical methods a. Provide details of the statistical methods used for each analysis, including software used. b. Describe any methods used to assess whether the data met the assumptions of the statistical approach, and what was done if the assumptions were not met.

Response: All statistical analyses were performed using GraphPad Prism10.0 software (GraphPad Software, San Diego, California, USA). Data are expressed as mean ± standard deviation (SD). The specific statistical tests used for each analysis are as follows:

Data Type	Applicable statistical methods
PASI scores<br>CCL7,CCL20,CCL27,MCP-1,	Two-way ANOVA with<br>repeated measures
IL-6, IL-8, IL-1β, TNF-α,	One-way ANOVA followed by<br>Tukey's post hoc test
Western blot Gray value	One-way ANOVA followed by<br>Tukey's post hoc test

Due to the relatively small sample size (n=6 per group), formal normality tests were not performed. However, based on graphical inspection of data distributions (Q-Q plots and histograms) and consistency with previous studies [Ni Q, Zhen L, Zeng Z, et al. Mesenchymal stromal cells restrain the Th17 cell response via L-amino-acid oxidase within lymph nodes. Cell Death Dis. 2024;15(9):640.], the data were considered to be approximately normally distributed. Homogeneity of variance was assessed using the Tukey's test test, and all data met this assumption. For all parametric tests, residuals were examined to ensure their approximate normality.

8. Experimental animals a. Provide species-appropriate details of the animals used, including species, strain and substrain, sex, age or developmental stage, and, if relevant, weight. b. Provide further relevant information on the provenance of animals, health/immune status, genetic modification status, genotype, and any previous procedures.

Response: a has already been addressed in the animal experiment section of the article.b These animals had not undergone any experimental procedures prior to this.

9. Experimental procedures For each experimental group, including controls, describe the procedures in enough detail to allow others to replicate them, including: a. What was done, how it was done and what was used. b. When and how often. c. Where (including detail of any acclimatisation periods). d. Why (provide rationale for procedures)

Response:All experimental procedures were conducted under specific pathogen-free conditions at the Beijing Meidekona Laboratory Animal Center. Mice were acclimated to the environment for 6 days prior to the start of the experiment to minimize stress.

a. Procedure, Implementation Method, and Materials Used

Depilation: One day prior to the first IMQ application (Day -1), mice were anesthetized with isoflurane (2–2.5% inhalation) and shaved using an electric shaver to remove hair from the dorsal skin (approximately 3 cm × 4 cm area). Depilatory cream (Veet, Reckitt Benckiser) was then applied for 30 seconds to remove residual hair. The skin was then gently wiped with PBS to remove residual depilatory cream.

IMQ-Induced Psoriasis Model: For the IMQ and IMQ+MSC groups, 62.5 mg of 5% imiquimod cream (Mingxin Sichuan, China) was applied daily to the shaved dorsal skin using a sterile spatula. Gently massage the cream for approximately 30 seconds to ensure even distribution. For the control group, apply an equal amount (62.5 mg) of petroleum jelly following the same procedure.

MSC Treatment: For the IMQ+MSC group, resuspend human adipose-derived mesenchymal stem cells in sterile phosphate-buffered saline at a concentration of [e.g., 2×10^6 cells/200 μL]. On days 1 and 4 of the IMQ application period, administer subcutaneous injections to mice using a 1 mL insulin syringe. Apply gentle pressure to the injection site with sterile gauze for 30 seconds post-injection to prevent bleeding. For the control and IMQ groups, administer an equal volume (200 μL) of sterile PBS via tail vein injection following the same schedule.

Sample collection: On day 6 (24 hours after the final IMQ application), mice were euthanized by CO_2 inhalation (flow rate: 20% of chamber volume per minute), followed by cervical dislocation to confirm death. Dorsal skin tissue collection: A portion was fixed in 4% paraformaldehyde for histological analysis, while the remainder was rapidly frozen in liquid nitrogen and stored at -80°C for protein extraction (ELISA and Western blot).Minimize excessive discomfort.

MSC administration schedule: MSCs were administered on Days 1 and 4 to evaluate early intervention effects (Day 1) and sustained therapeutic effects (Day 4) during IMQ-induced inflammatory progression. The dose of [2×10^6 cells/injection] was selected based on prior studies demonstrating efficacy in psoriasis models.

b. Treatment Timing and Frequency

Depilation: Performed once, on the day prior to the first IMQ application (Day -1).

IMQ/Vaseline Application: Once daily for 6 consecutive days (Day 0 to Day 6).

MSC/PBS Injection: Administered once each on Day 1 and Day 4.

Clinical Scoring: PASI scoring performed daily at approximately the same time (9:00-11:00 AM) throughout the study period (D0 to D6).

c.Sample Collection: Conducted 24 hours after the final IMQ application on Day 6.

All procedures were conducted within the animal facility at Beijing Meidekona. A 7-day acclimation period preceded experimentation, during which mice were housed under controlled conditions (temperature 22 ± 2°C, humidity 50 ± 10%, 12-hour light/dark cycle) with free access to food and water. IMQ application and PASI scoring were performed in designated operating rooms within the animal facility. Subcutaneous injections were performed in a sterile biosafety cabinet. Euthanasia and tissue collection were conducted in a separate dissection room.

d. Operational Basis (Explanation of Protocol Design Principles)

IMQ Dose and Duration: The selection of a dose of 62.5 mg daily for 6 consecutive days (approximately 5% IMQ cream) is based on an established protocol that reliably induces psoriasiform skin inflammation in C57BL/6 mice, characterized by erythema, scaling, and thickening. This duration allows for the formation of stable psoriatic lesions while minimizing excessive discomfort.

MSC Administration Schedule: MSCs were administered on Days 1 and 4 to evaluate the effects of early intervention (Day 1) and sustained treatment (Day 4) during the progression of IMQ-induced inflammation. The dose of [2 × 10^6 cells/injection] was selected based on prior studies demonstrating its efficacy in psoriasis models.

Subcutaneous Injection Route: Subcutaneous injection was chosen to ensure MSC distribution at the mouse skin lesion site for immunomodulatory effects.

Euthanasia Timing: Euthanasia performed 24 hours after the final IMQ application to capture the peak inflammatory response while avoiding acute stress from the last treatment.

Tissue Processing: Skin samples divided into paraformaldehyde-fixed (to preserve tissue structure for histology) and -80°C frozen (to maintain protein integrity for molecular analysis).

10 Results For each experiment conducted, including independent replications, report:

a. Summary/descriptive statistics for each experimental group, with a measure of variability

where applicable (e.g. mean and SD, or median and range).

b. If applicable, the effect size with a confidence interval.

Response: The mean ± standard deviation of the primary statistical indicators for mice in the text is provided below.

Group	TNF-α	IL-1β	IL6	IL8
Control	1.04 ± 0.12	0.94 ± 0.16	0.93 ± 0.11	0.95 ± 0.07
IMQ	6.78 ± 0.09	7.14 ± 1.35	6.75 ± 0.10	6.44 ± 0.52
IMQ+MSCs	3.59 ± 0.94	2.41 ± 0.41	3.20 ± 0.25	3.02 ± 0.07

Group	CCL7	CCL27	CCL20	MCP-1
Control	1.055 ± 0.151	0.862 ± 0.124	1.083 ± 0.107	0.848 ± 0.143
IMQ	19.530 ± 0.914	19.099 ± 0.908	21.823 ± 1.342	16.675 ± 0.508
IMQ+MSCs	7.437 ± 0.882	7.598 ± 0.219	10.165 ± 0.234	5.607 ± 0.092

Group	Ki67	KRT1	KRT6	Claudin-1
Control	16.67 ± 2.22	10.13 ± 0.24	10.00 ± 0.00	141.67 ± 7.78
IMQ	200 ± 6.67	2.23 ± 0.16	121.00 ± 2.67	78.33 ± 8.89
IMQ+MSCs	50 ± 6.67	23.00 ± 0.67	50.00 ± 6.67	155.00 ± 6.67
