# Supplementary material for: Research based on serine metabolism indicates mesenchymal stem cells alleviate psoriasis by regulating the PSPH-PINK1-Parkin-NLRP3 pathway in HaCaT
Source: Stem Cell Res Ther. 2026 Mar 28;17:176. doi: 10.1186/s13287-026-04964-z (PMC13151198; doi:10.1186/s13287-026-04964-z)

3 组人的 p-Parkin

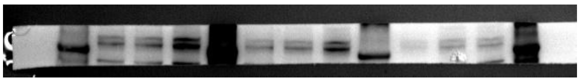

51KDa

人 GAPDH

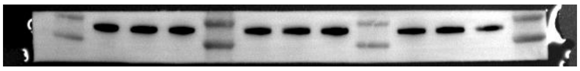

36KDa

人 p-parkin+GAPDH 拼接全膜

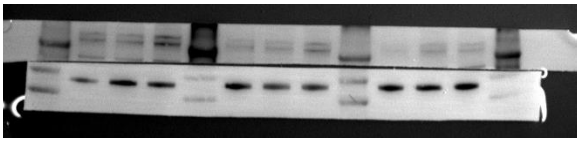

3 组小鼠的 p-Parkin

小鼠 GAPDH

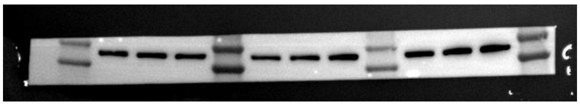

36KDa

小鼠 p-parkin

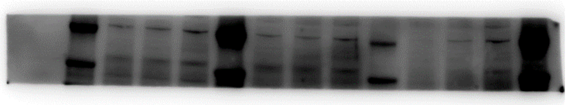

51KDa

小鼠 p-parkin+GAPDH 拼接全膜

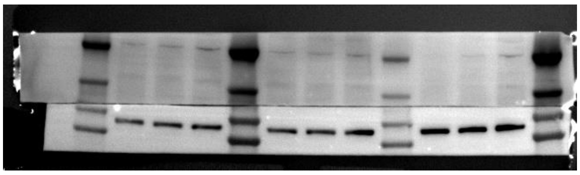

人 5 组的 p-Parkinx1

GAPDH

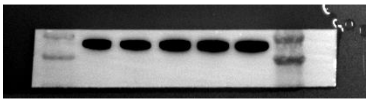

36KDa

p-Parkinx1

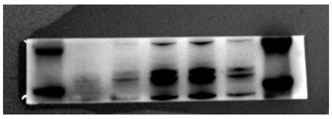

51KDa

p-Parkinx+GAPDHx1 拼接

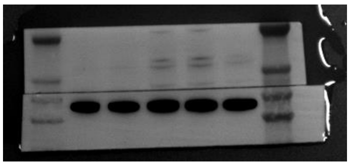

GAPDH X2

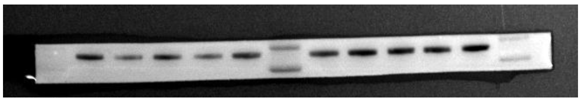

36KDa

p-Parkinx2

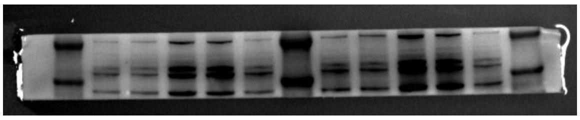

51KDa

p-Parkinx2+GAPDH 拼接

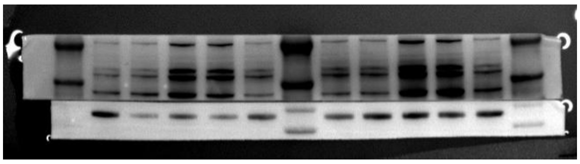

4 组人的 p-parkin

GAPDH

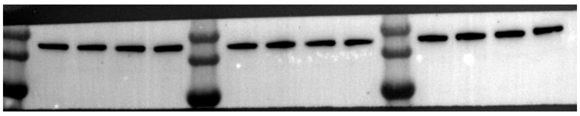

36KDa

p-parkin

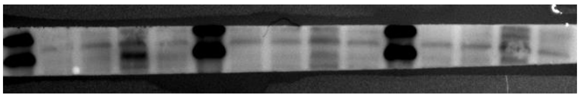

51KDa

p-parkin4 组拼接

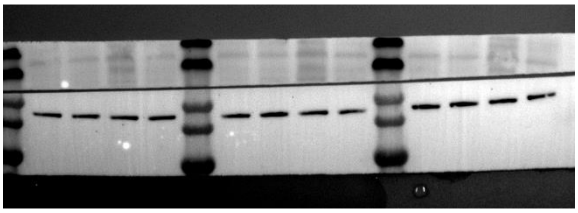

Supplement: Supplementary file 2 — Additional file 2. [file 13287_2026_4964_MOESM2_ESM.zip › Figures2025.12+supplement p-parkin WB/supplementWBp-parkin2026.1.1.pdf]
