# Supplementary figures and images for: Research based on serine metabolism indicates mesenchymal stem cells alleviate psoriasis by regulating the PSPH-PINK1-Parkin-NLRP3 pathway in HaCaT
Source: Stem Cell Res Ther. 2026 Mar 28;17:176. doi: 10.1186/s13287-026-04964-z (PMC13151198; doi:10.1186/s13287-026-04964-z)

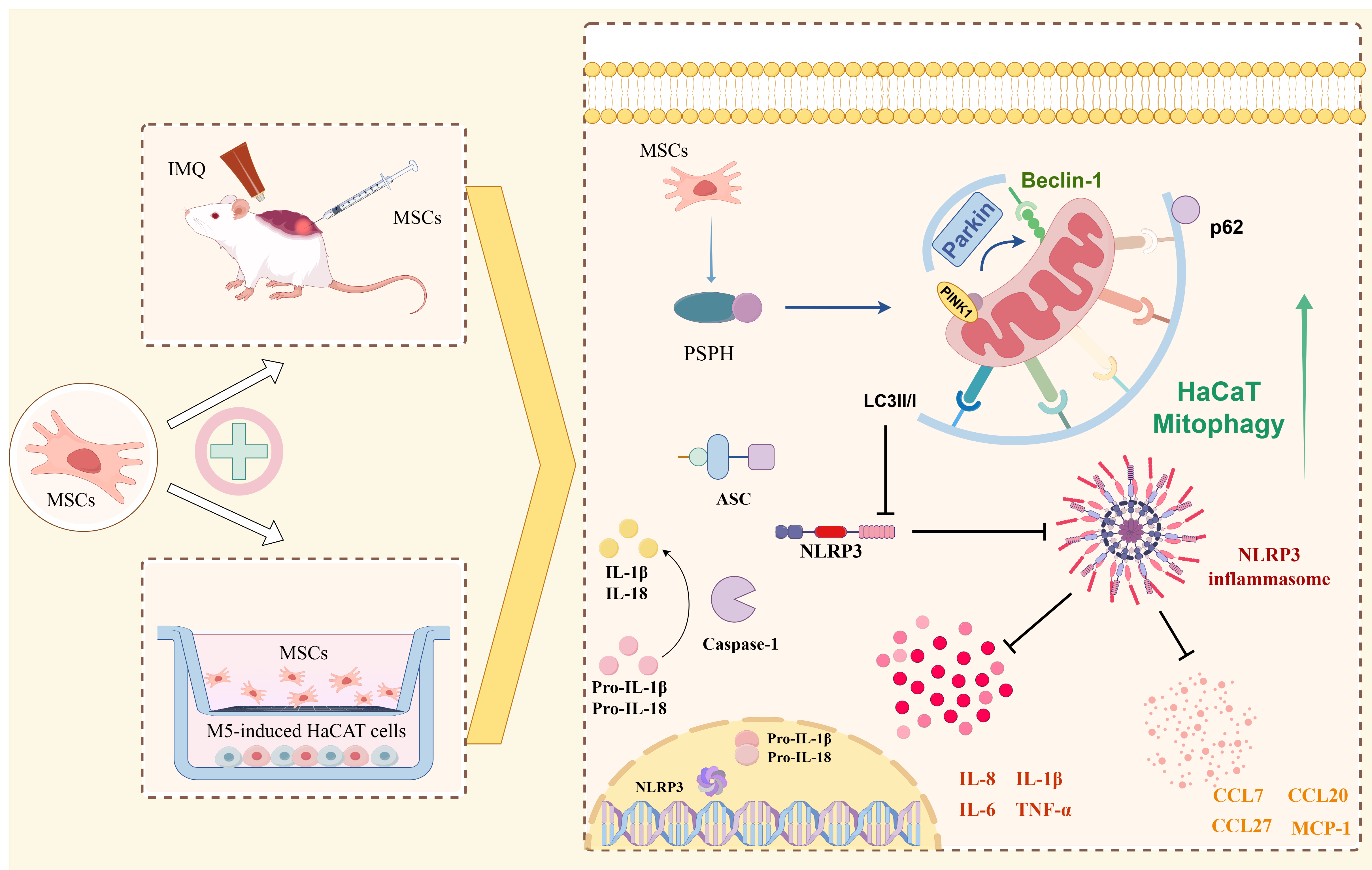

Supplement: Supplementary file 2 — Additional file 2. [file 13287_2026_4964_MOESM2_ESM.zip › Figures2025.12+supplement p-parkin WB/MSCmechanism0908.png]

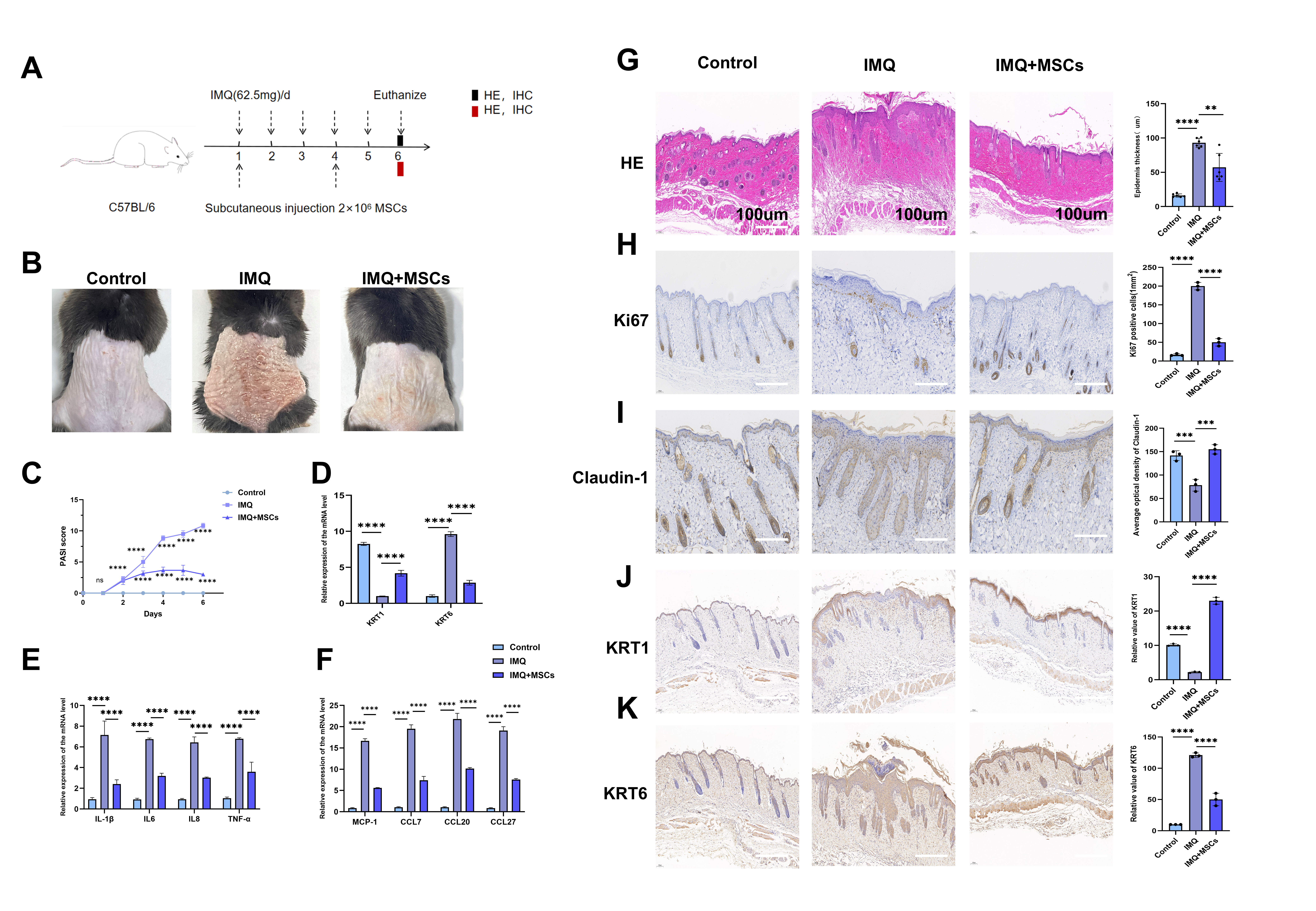

Supplement: Supplementary file 2 — Additional file 2. [file 13287_2026_4964_MOESM2_ESM.zip › Figures2025.12+supplement p-parkin WB/fig1.tif]

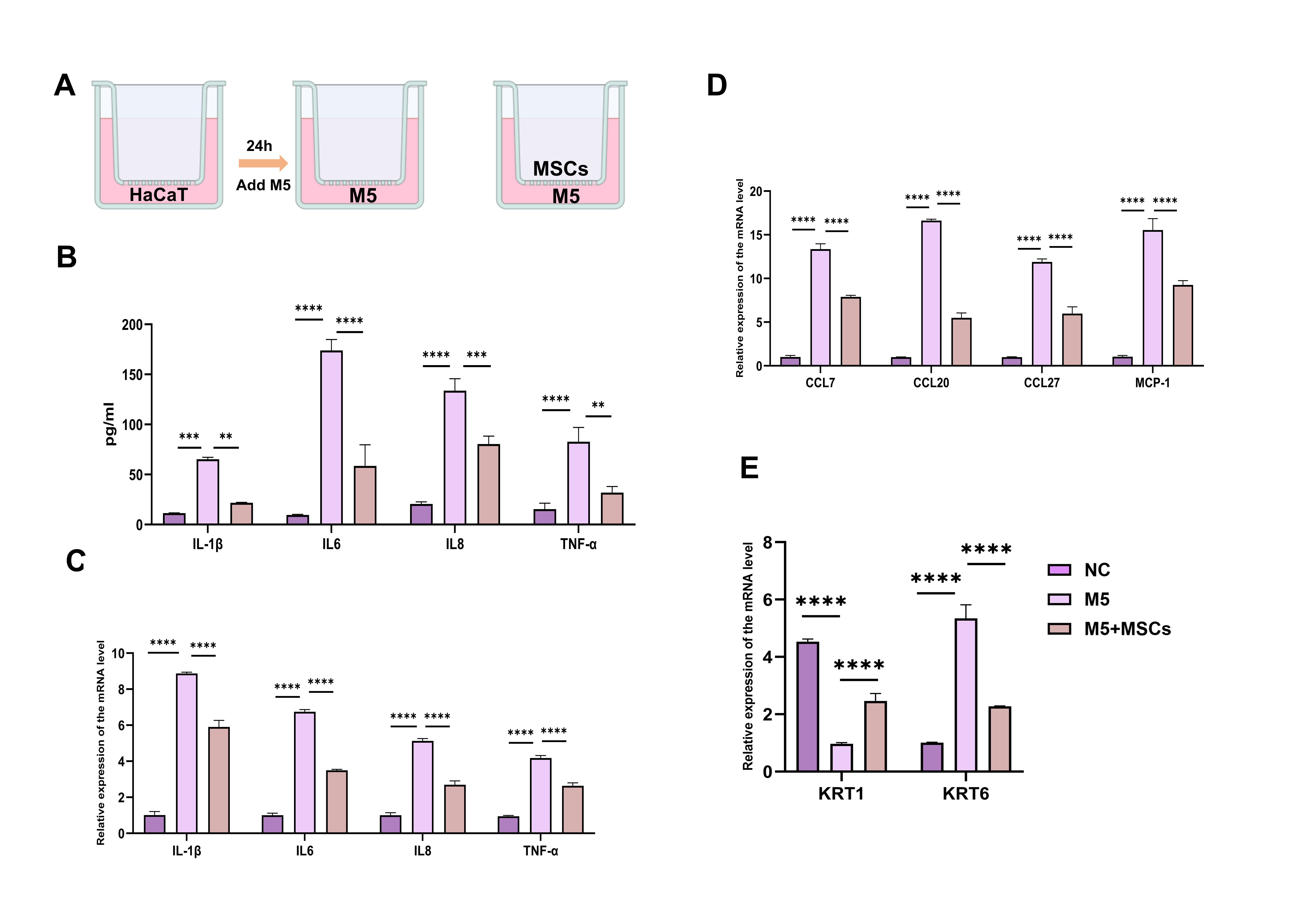

Supplement: Supplementary file 2 — Additional file 2. [file 13287_2026_4964_MOESM2_ESM.zip › Figures2025.12+supplement p-parkin WB/fig2.tif]

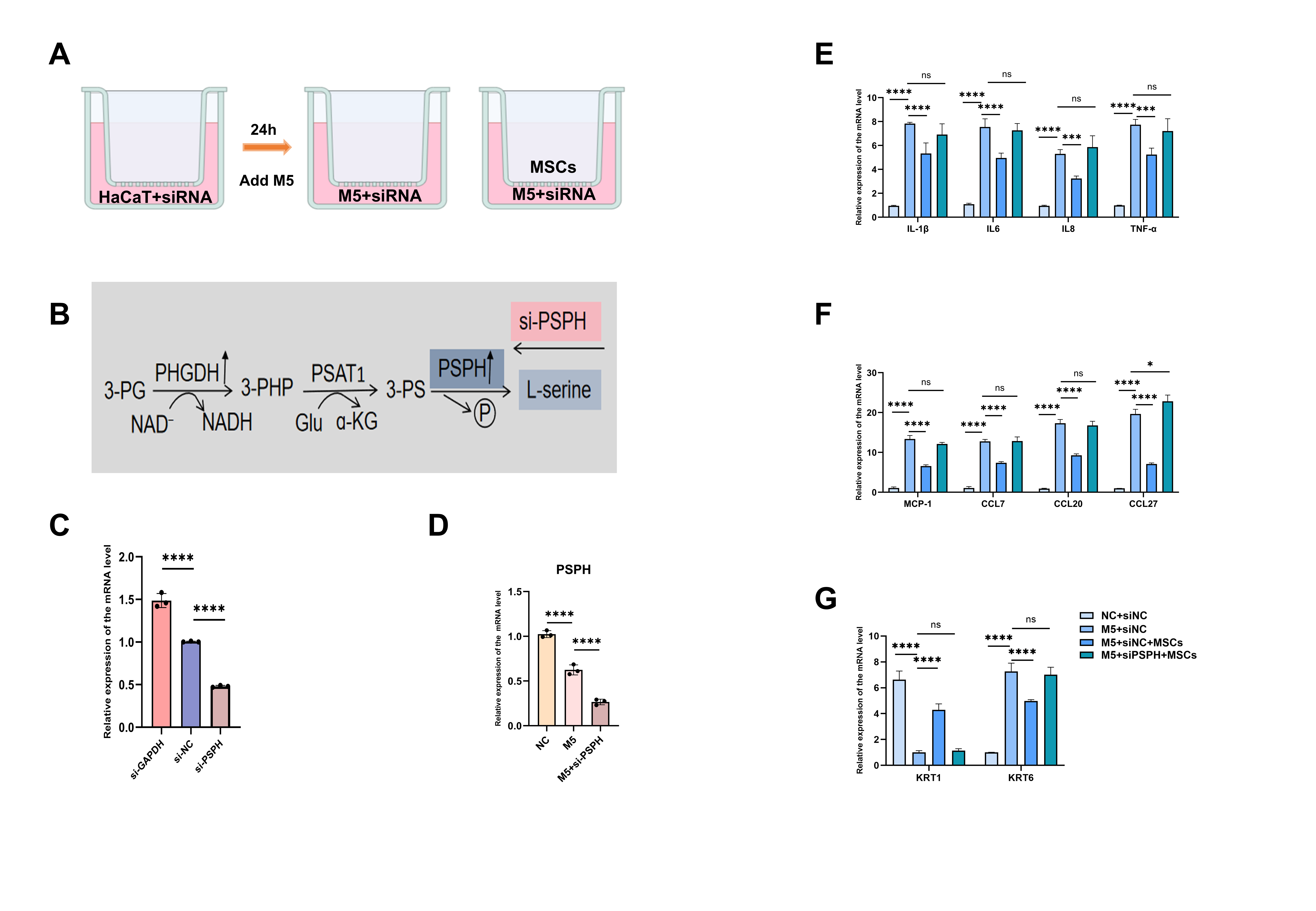

Supplement: Supplementary file 2 — Additional file 2. [file 13287_2026_4964_MOESM2_ESM.zip › Figures2025.12+supplement p-parkin WB/fig4.tif]

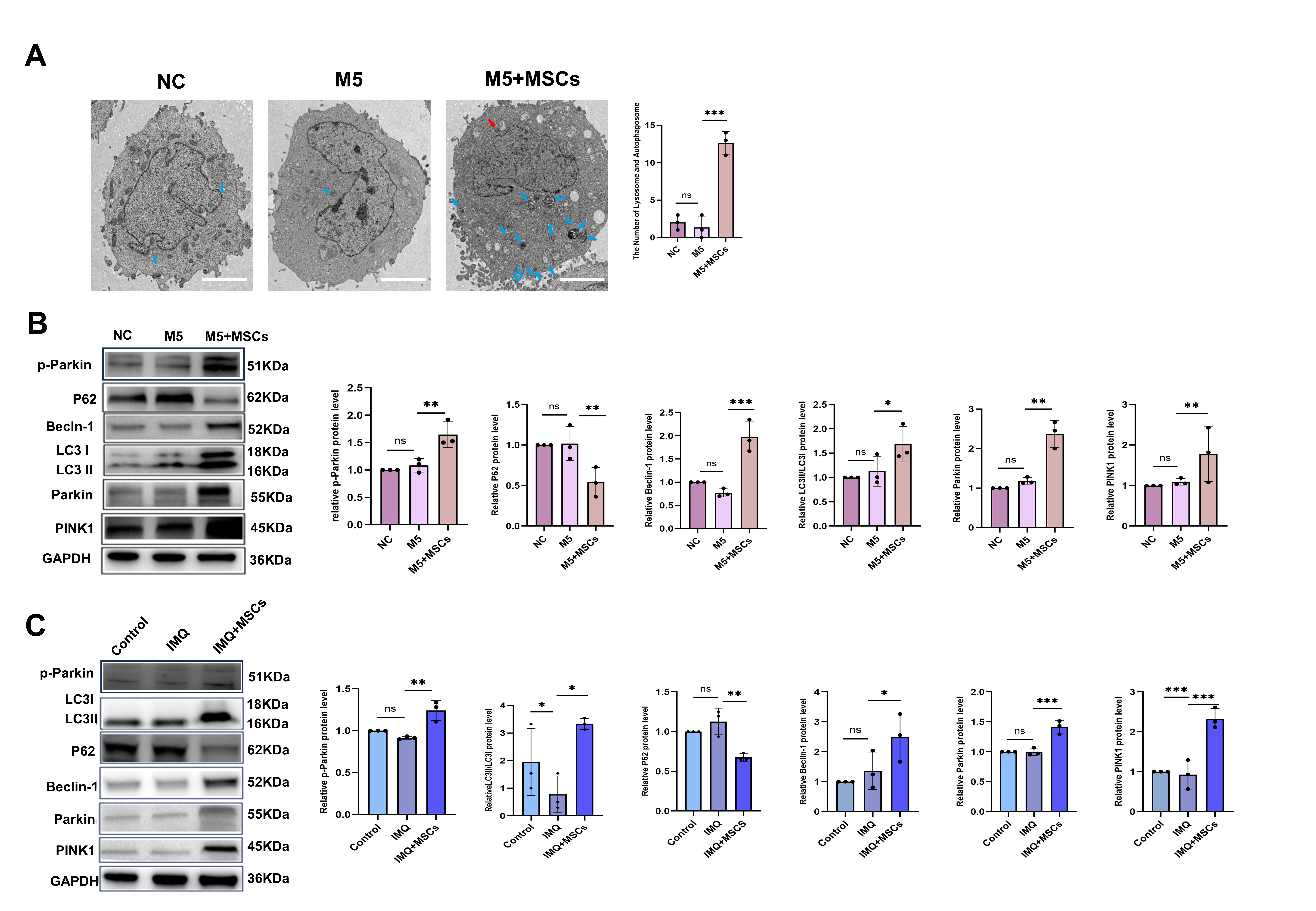

Supplement: Supplementary file 2 — Additional file 2. [file 13287_2026_4964_MOESM2_ESM.zip › Figures2025.12+supplement p-parkin WB/fig5.tif]

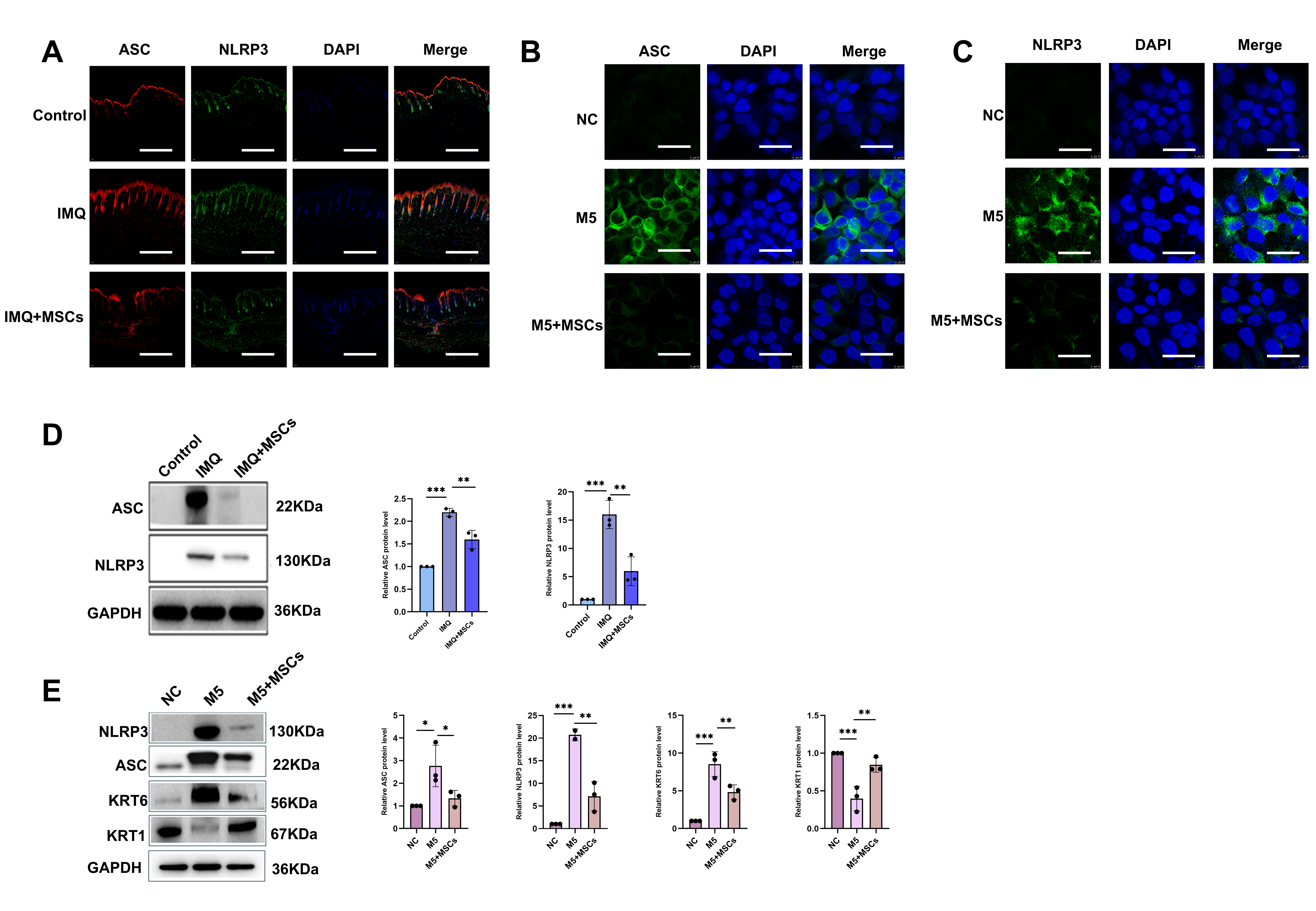

Supplement: Supplementary file 2 — Additional file 2. [file 13287_2026_4964_MOESM2_ESM.zip › Figures2025.12+supplement p-parkin WB/fig6.tif]

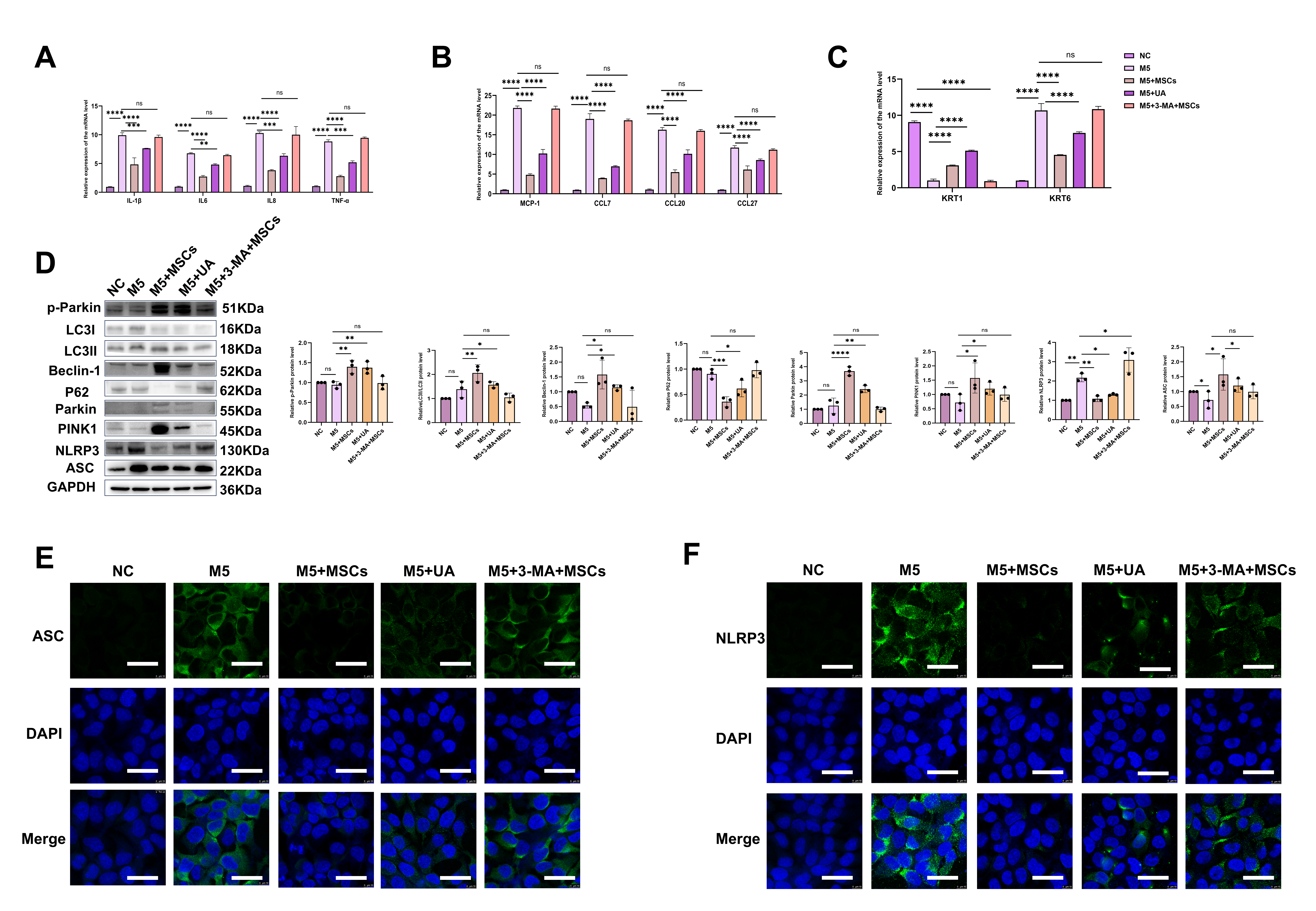

Supplement: Supplementary file 2 — Additional file 2. [file 13287_2026_4964_MOESM2_ESM.zip › Figures2025.12+supplement p-parkin WB/fig7.tif]

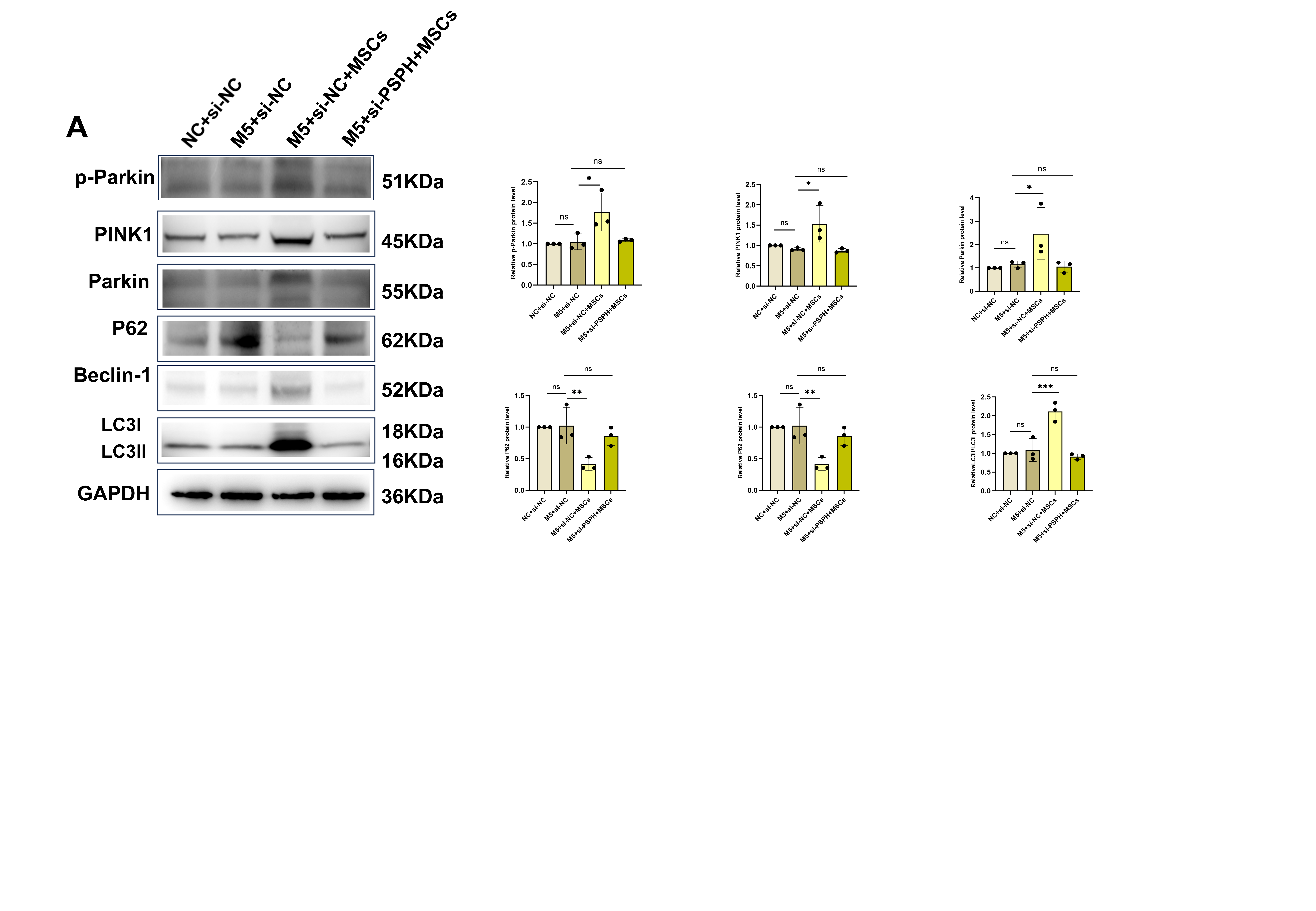

Supplement: Supplementary file 2 — Additional file 2. [file 13287_2026_4964_MOESM2_ESM.zip › Figures2025.12+supplement p-parkin WB/fig8.tif]

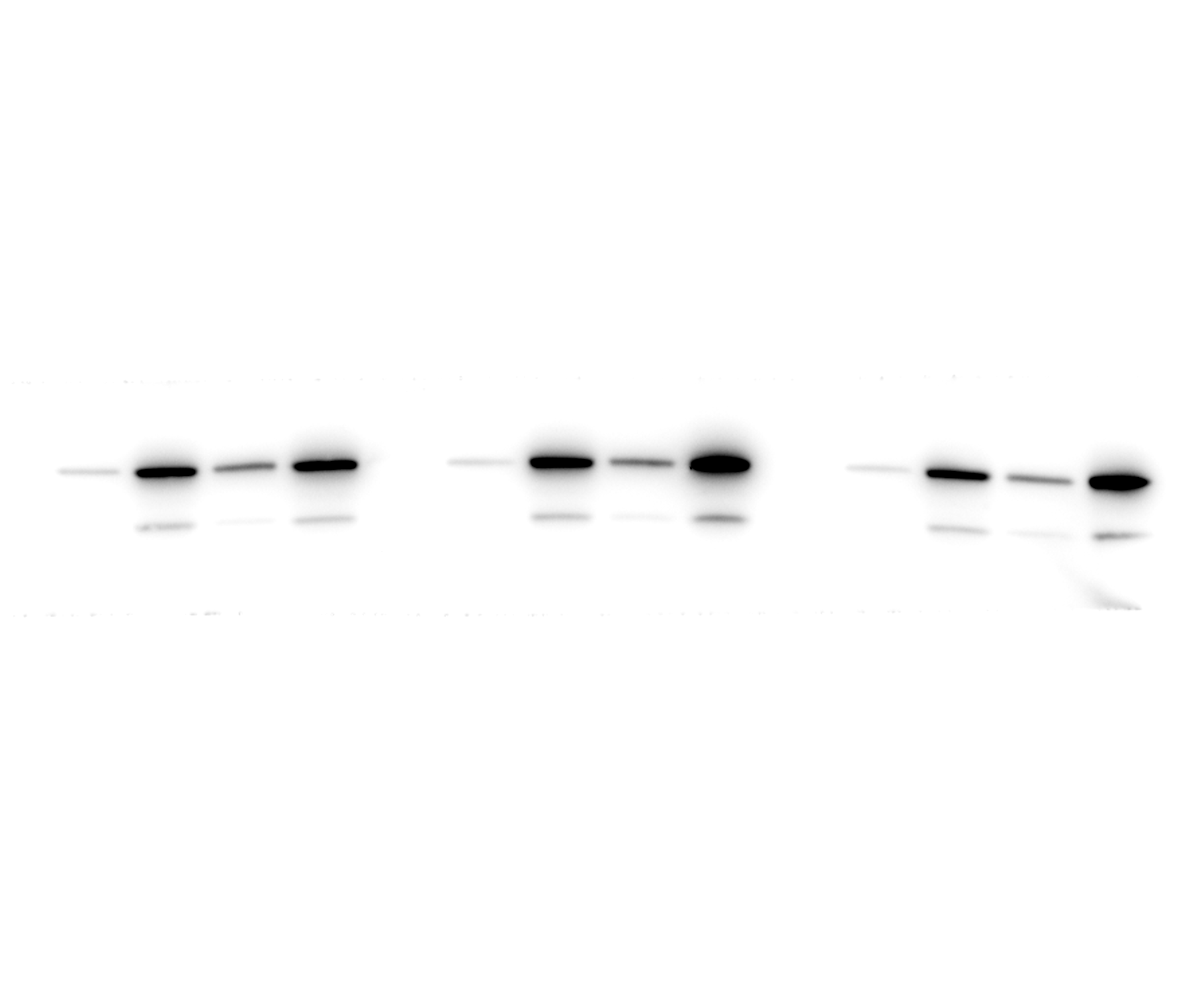

Supplement: Supplementary file 3 — Additional file 3. [file 13287_2026_4964_MOESM3_ESM.zip › Raw WB data 0809/ASC+NLRP3+PARKIN SIRNA0428/ASC-600MS.Tif]

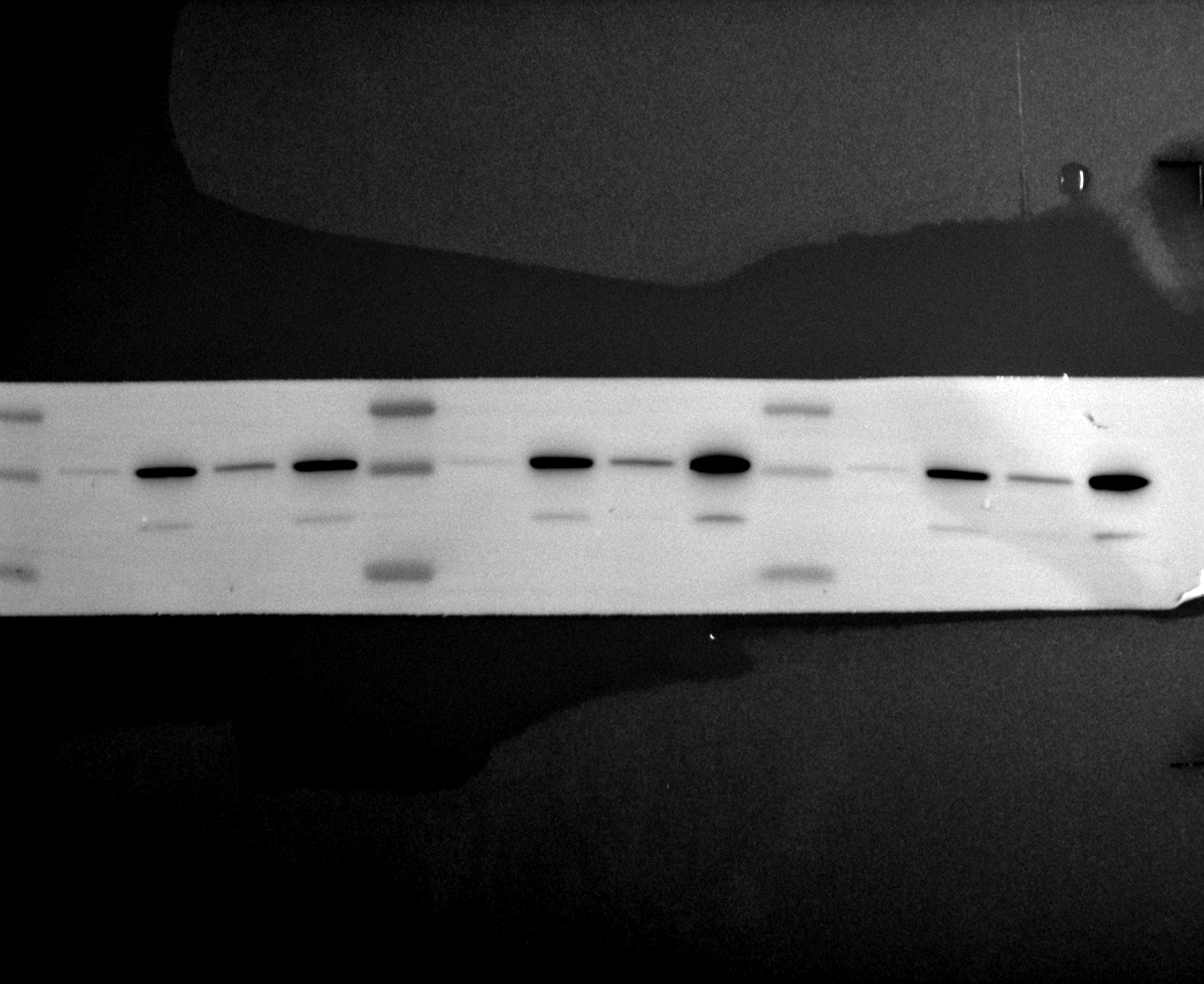

Supplement: Supplementary file 3 — Additional file 3. [file 13287_2026_4964_MOESM3_ESM.zip › Raw WB data 0809/ASC+NLRP3+PARKIN SIRNA0428/ASC全膜.Tif]

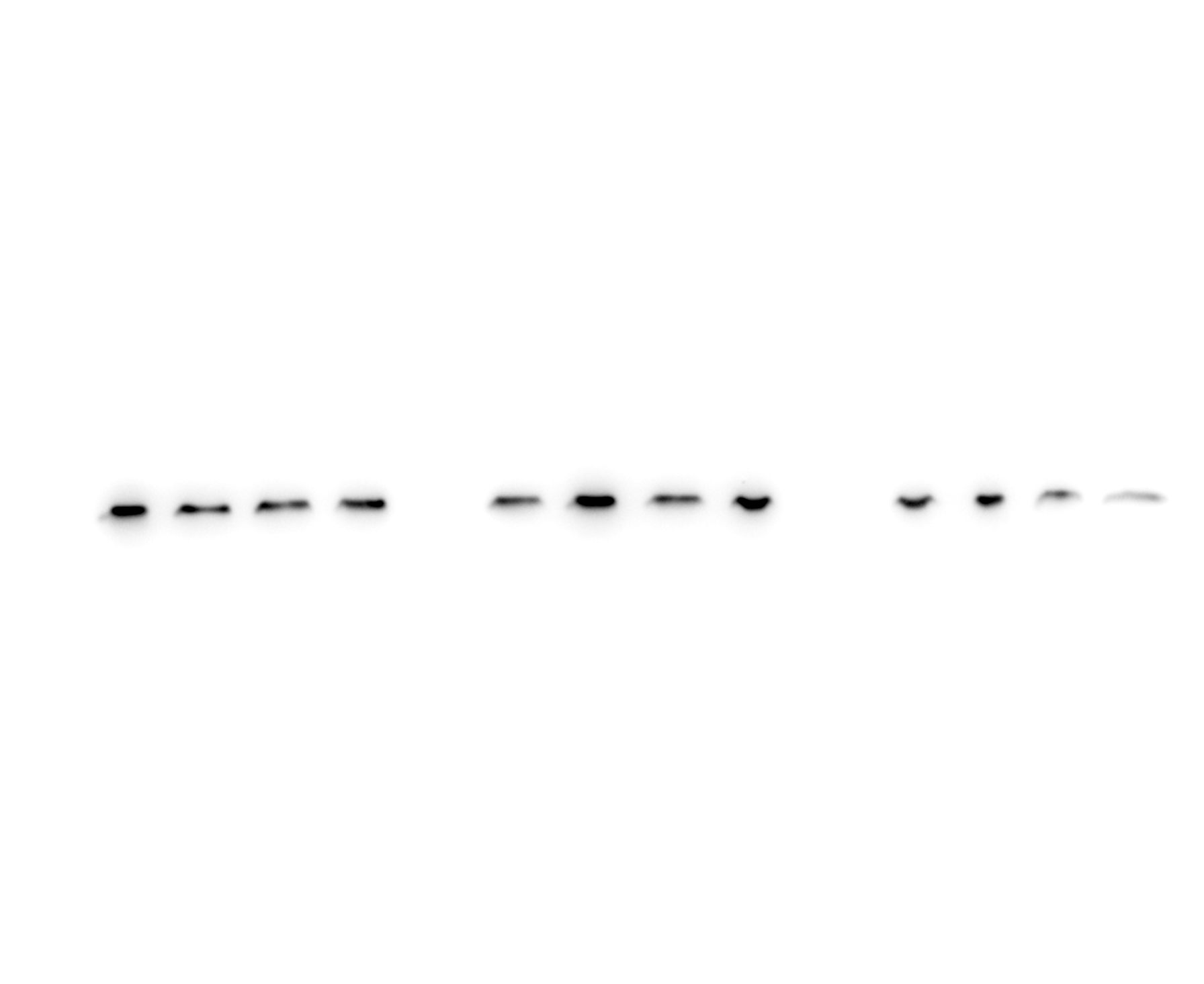

Supplement: Supplementary file 3 — Additional file 3. [file 13287_2026_4964_MOESM3_ESM.zip › Raw WB data 0809/ASC+NLRP3+PARKIN SIRNA0428/GAPDH-800MS.Tif]

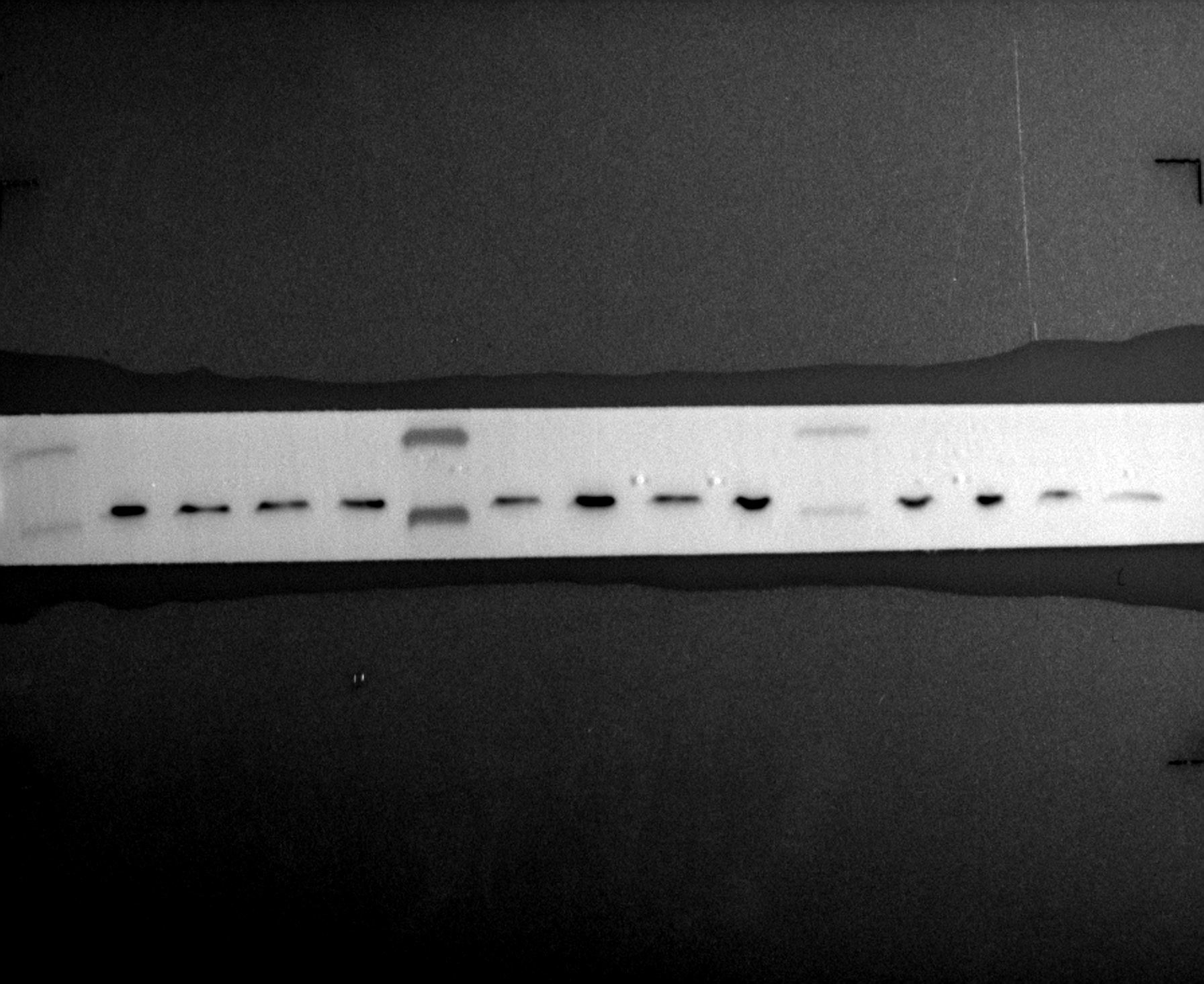

Supplement: Supplementary file 3 — Additional file 3. [file 13287_2026_4964_MOESM3_ESM.zip › Raw WB data 0809/ASC+NLRP3+PARKIN SIRNA0428/GAPDH全膜.Tif]

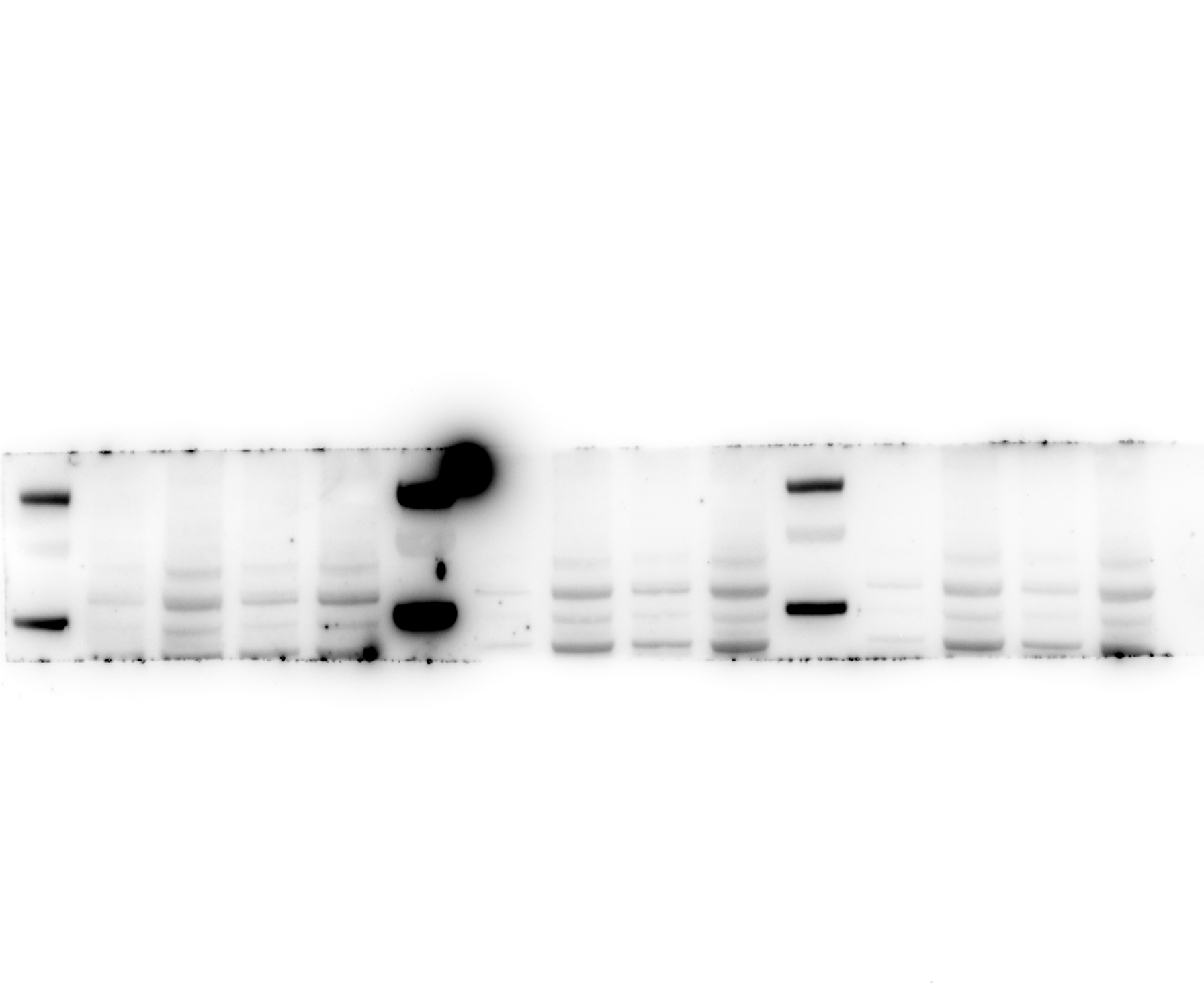

Supplement: Supplementary file 3 — Additional file 3. [file 13287_2026_4964_MOESM3_ESM.zip › Raw WB data 0809/ASC+NLRP3+PARKIN SIRNA0428/NLRP3-30S.Tif]

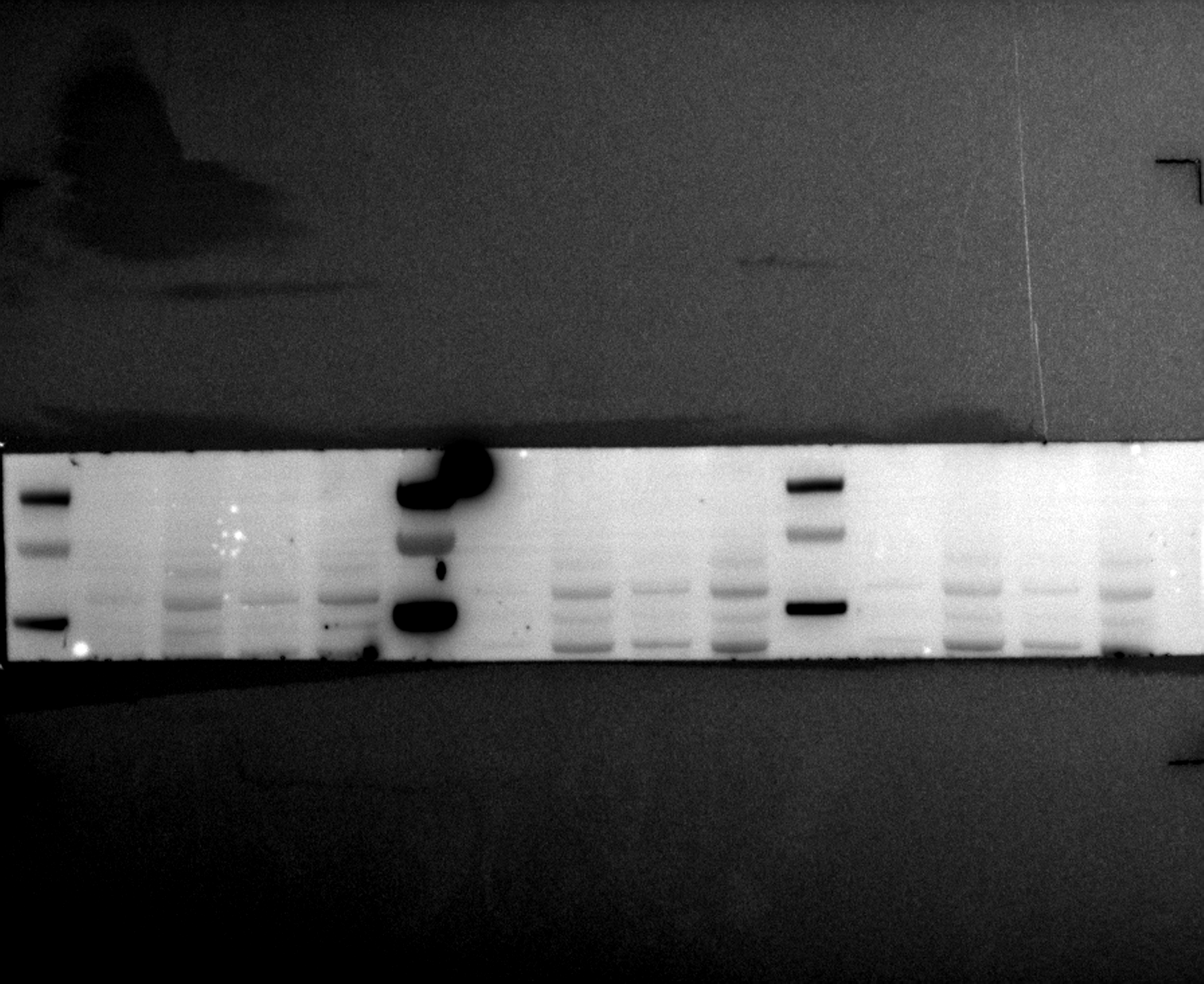

Supplement: Supplementary file 3 — Additional file 3. [file 13287_2026_4964_MOESM3_ESM.zip › Raw WB data 0809/ASC+NLRP3+PARKIN SIRNA0428/NLRP3全膜.Tif]

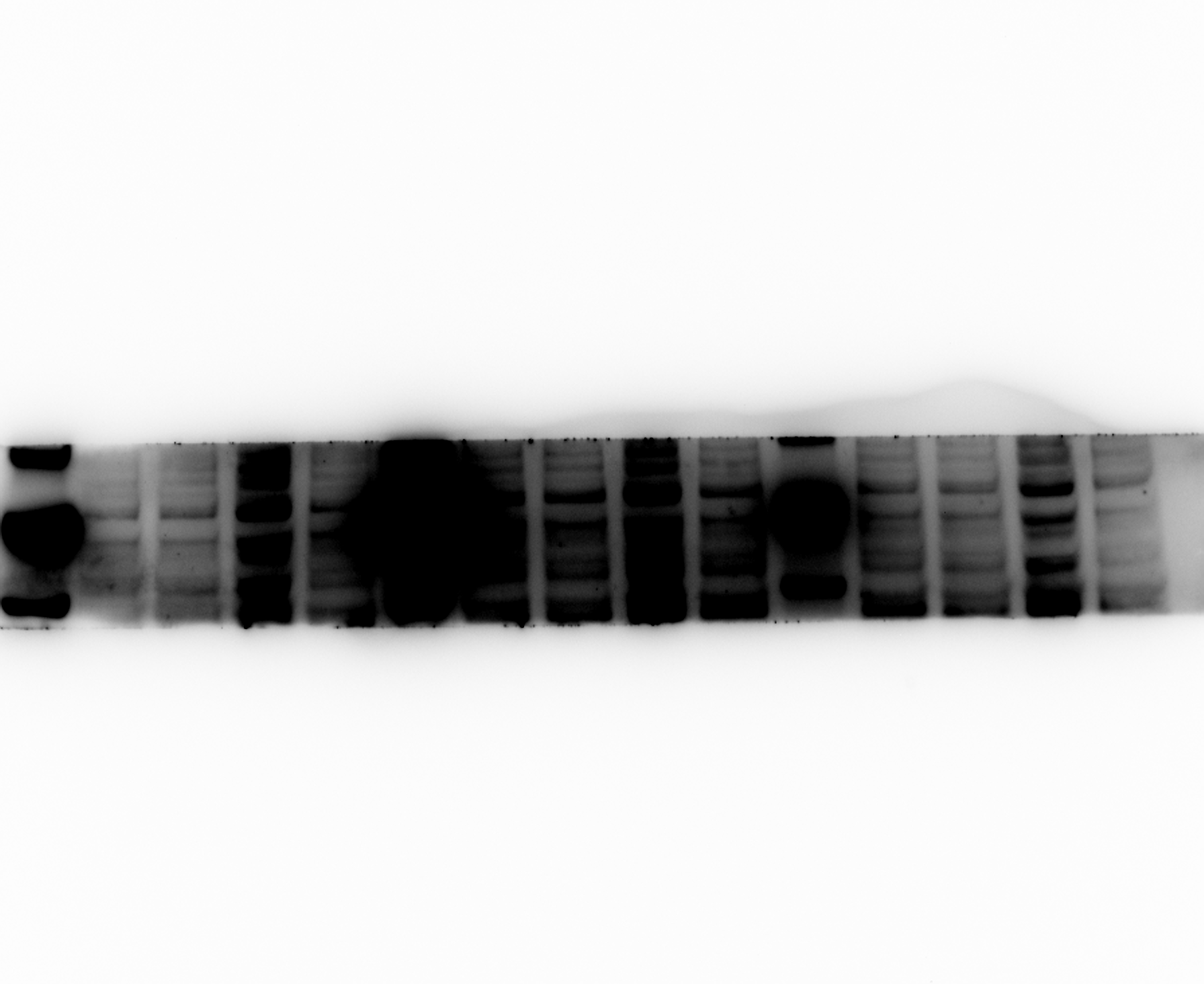

Supplement: Supplementary file 3 — Additional file 3. [file 13287_2026_4964_MOESM3_ESM.zip › Raw WB data 0809/ASC+NLRP3+PARKIN SIRNA0428/parkin-10s.Tif]

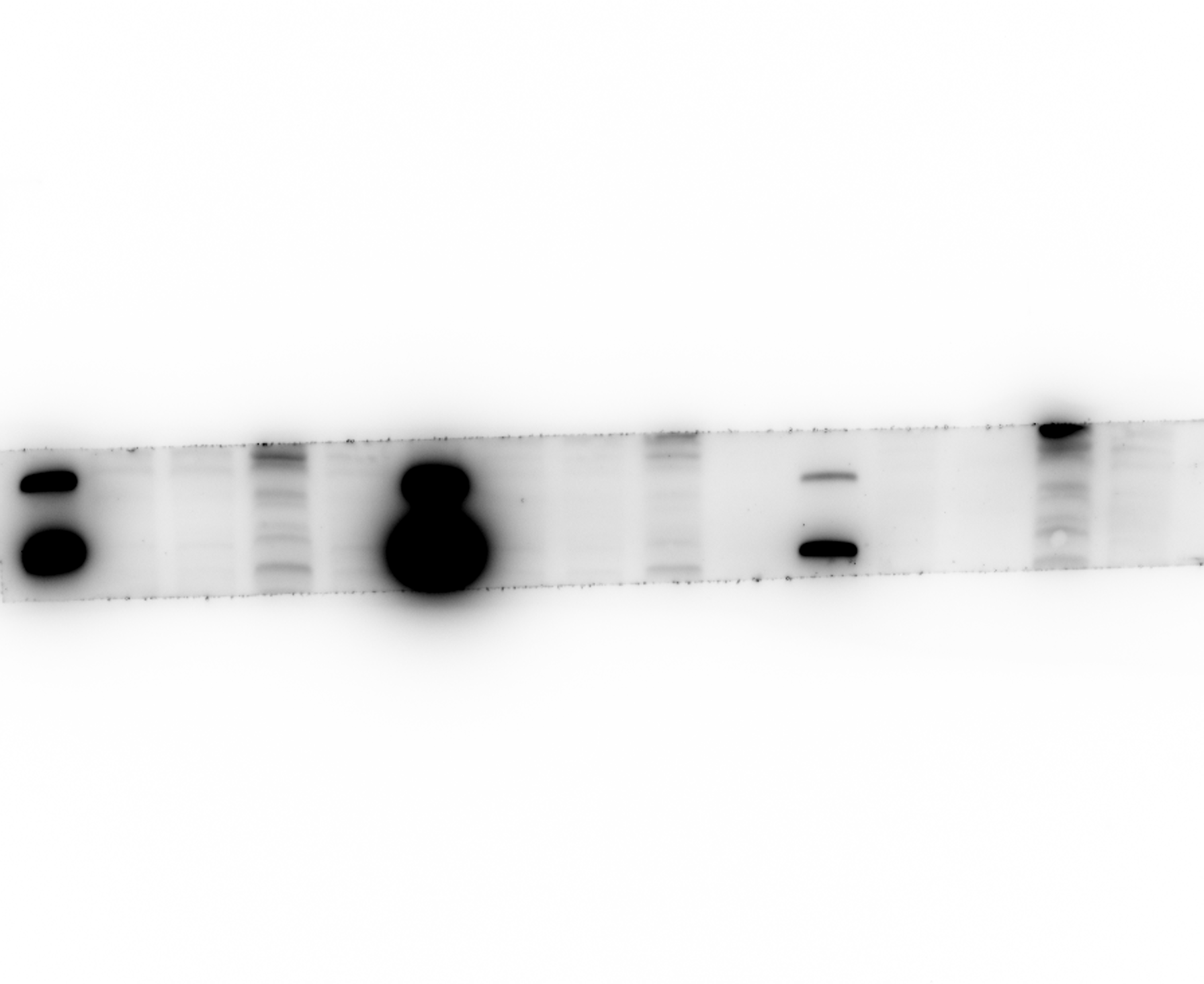

Supplement: Supplementary file 3 — Additional file 3. [file 13287_2026_4964_MOESM3_ESM.zip › Raw WB data 0809/ASC+NLRP3+PARKIN SIRNA0428/parkin-5s.Tif]

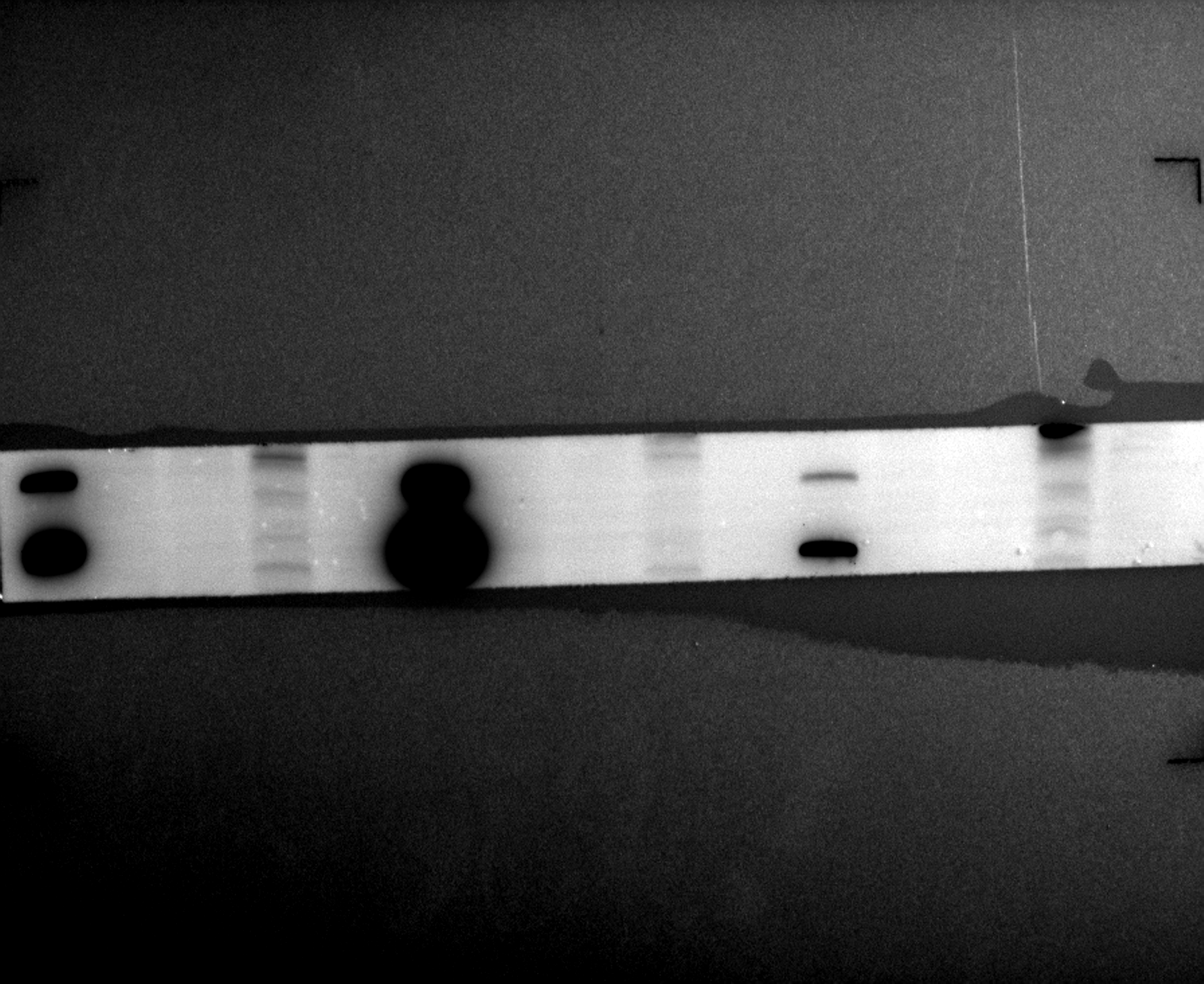

Supplement: Supplementary file 3 — Additional file 3. [file 13287_2026_4964_MOESM3_ESM.zip › Raw WB data 0809/ASC+NLRP3+PARKIN SIRNA0428/parkin全膜.Tif]

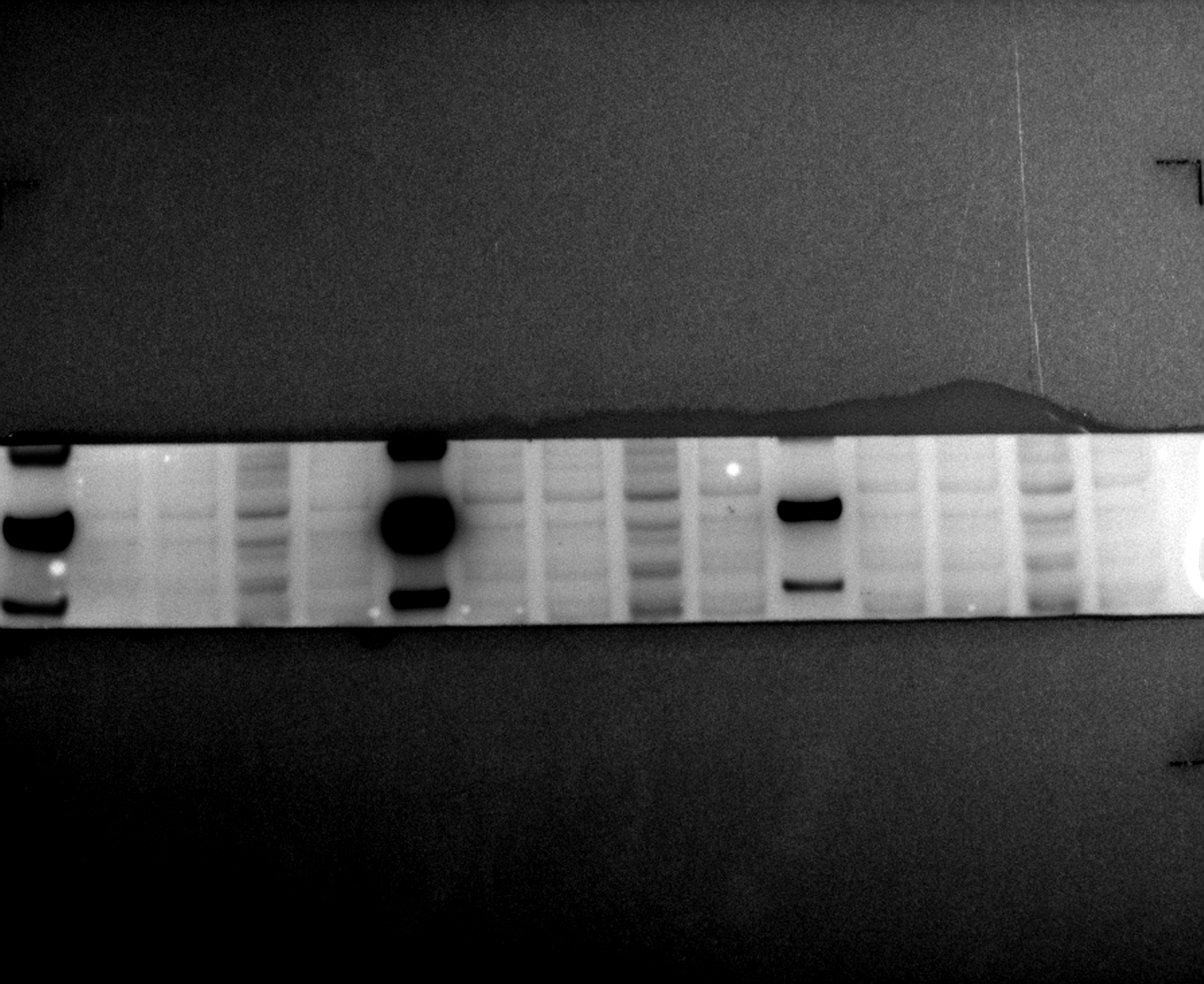

Supplement: Supplementary file 3 — Additional file 3. [file 13287_2026_4964_MOESM3_ESM.zip › Raw WB data 0809/ASC+NLRP3+PARKIN SIRNA0428/parkin全膜.Tif0429.Tif]

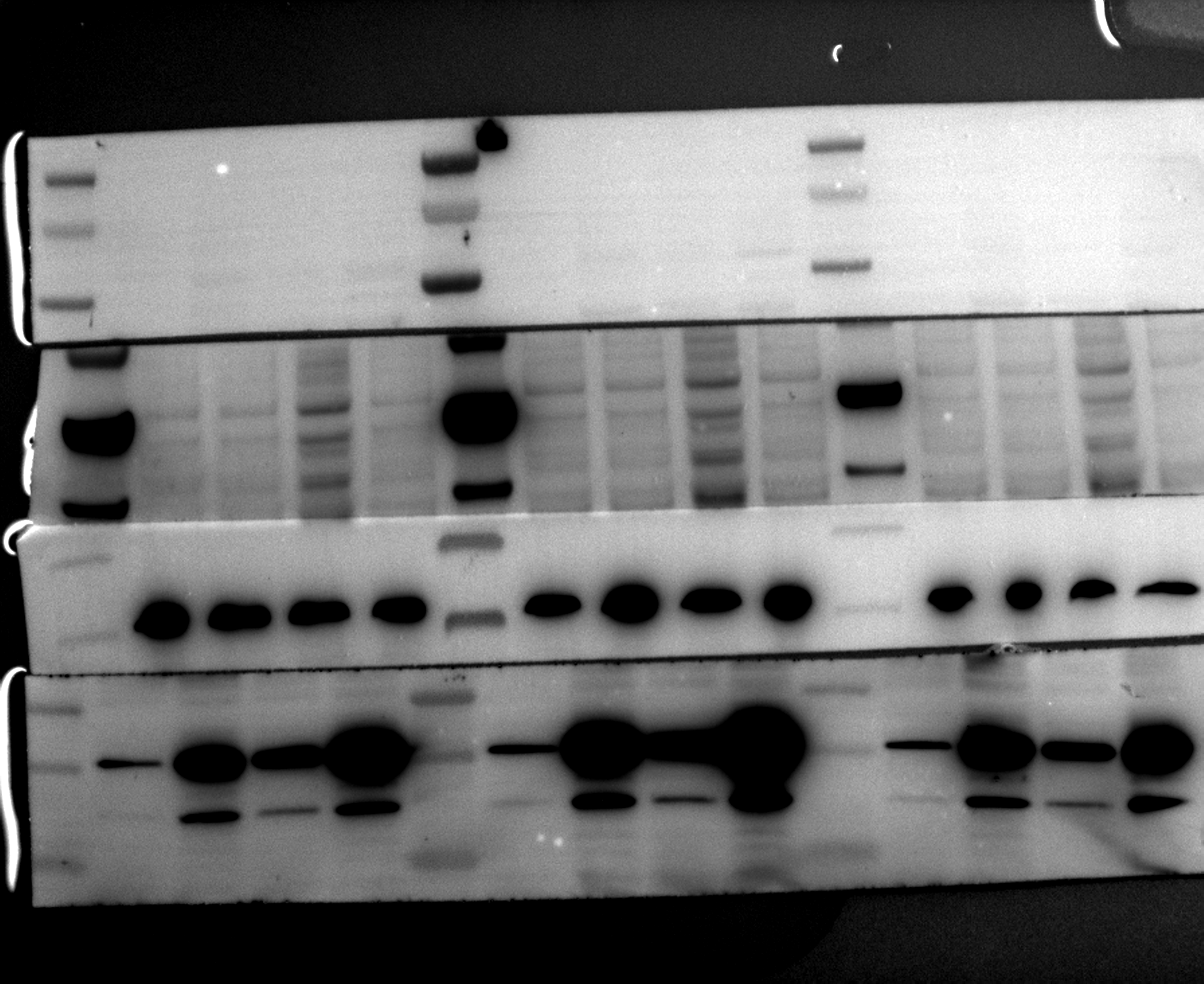

Supplement: Supplementary file 3 — Additional file 3. [file 13287_2026_4964_MOESM3_ESM.zip › Raw WB data 0809/ASC+NLRP3+PARKIN SIRNA0428/全膜拼接.Tif]

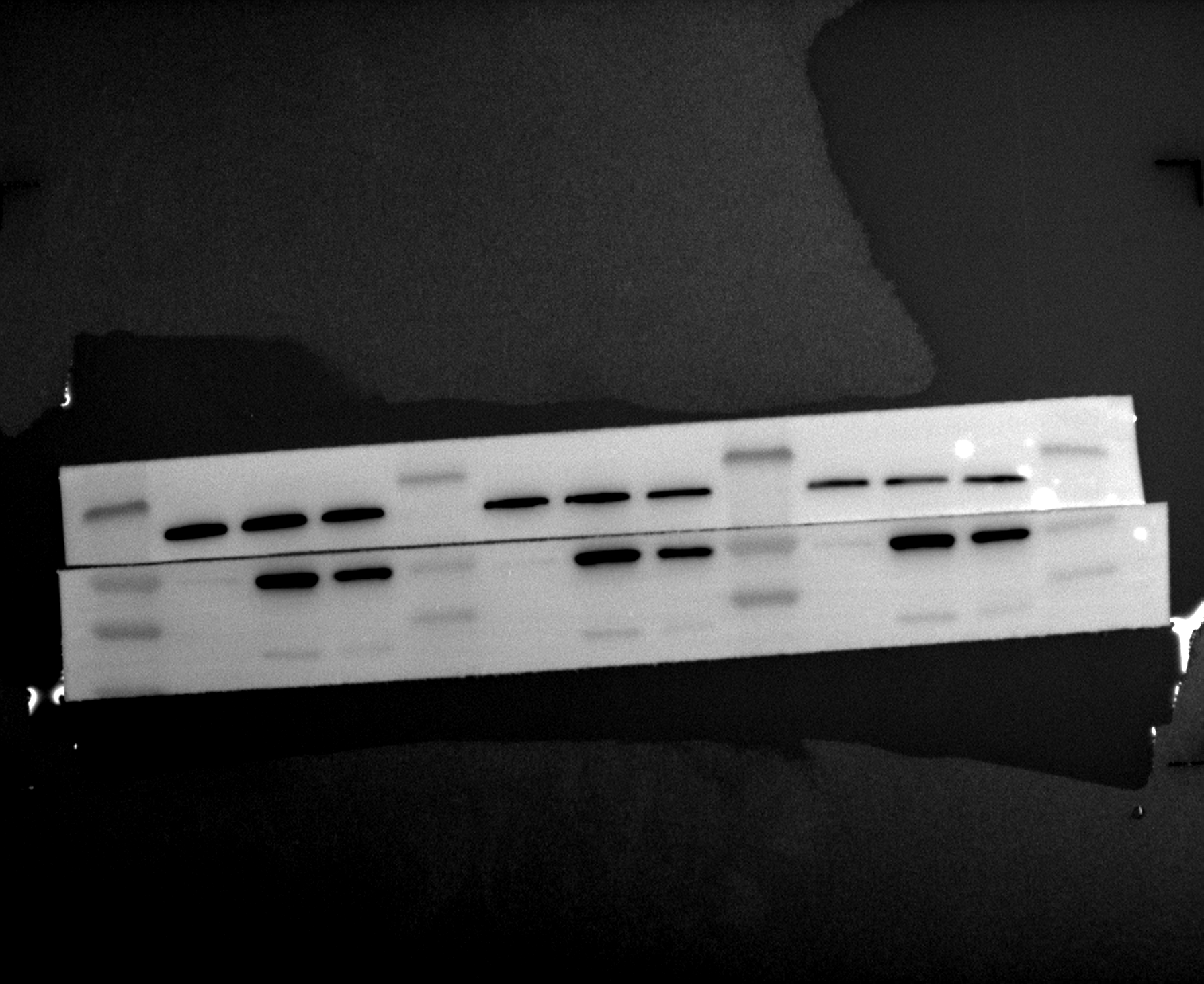

Supplement: Supplementary file 3 — Additional file 3. [file 13287_2026_4964_MOESM3_ESM.zip › Raw WB data 0809/ASC0306小鼠GAPDH+ASC/ASC+GAPDH全膜.Tif]

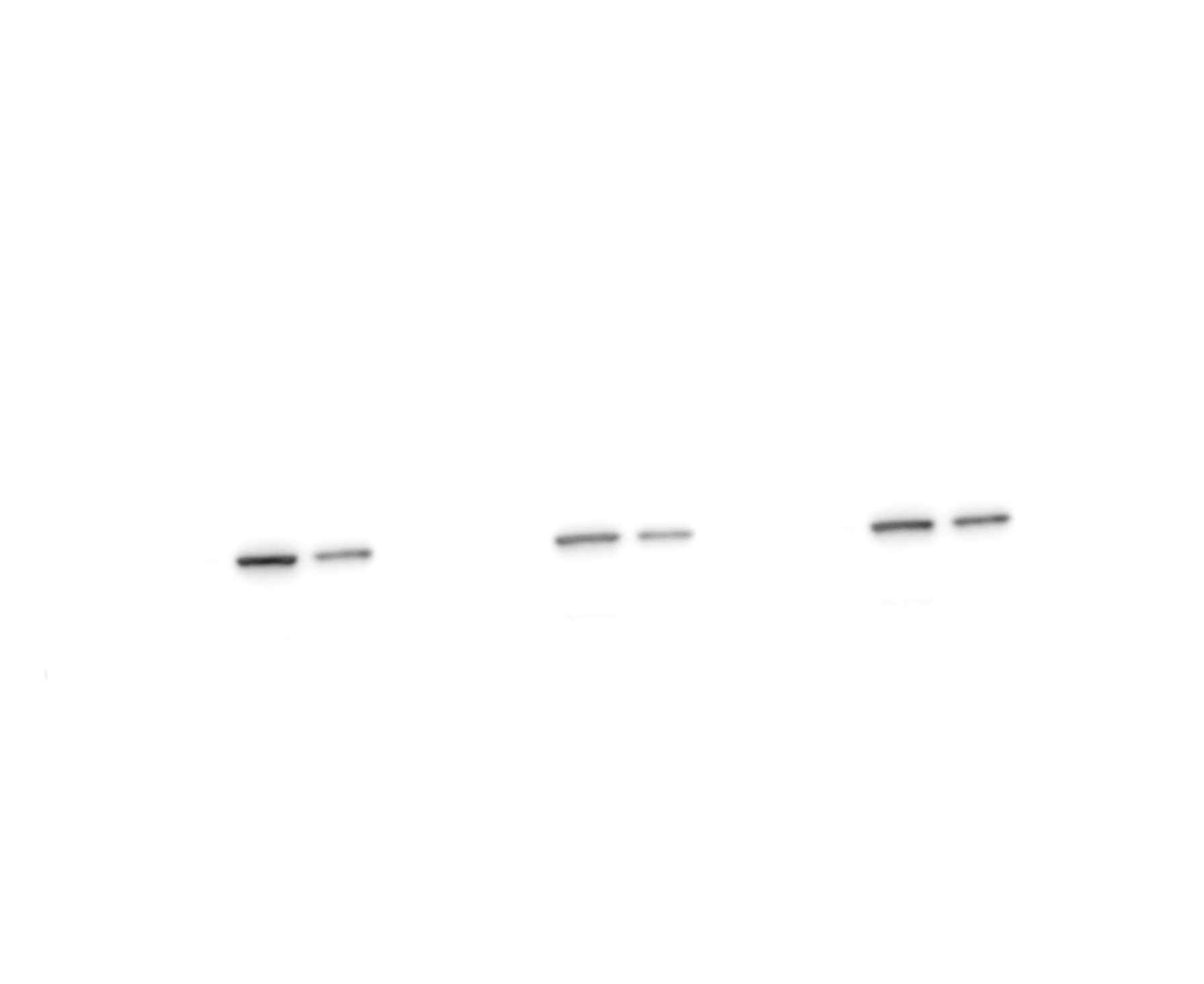

Supplement: Supplementary file 3 — Additional file 3. [file 13287_2026_4964_MOESM3_ESM.zip › Raw WB data 0809/ASC0306小鼠GAPDH+ASC/ASC-800ms.Tif]

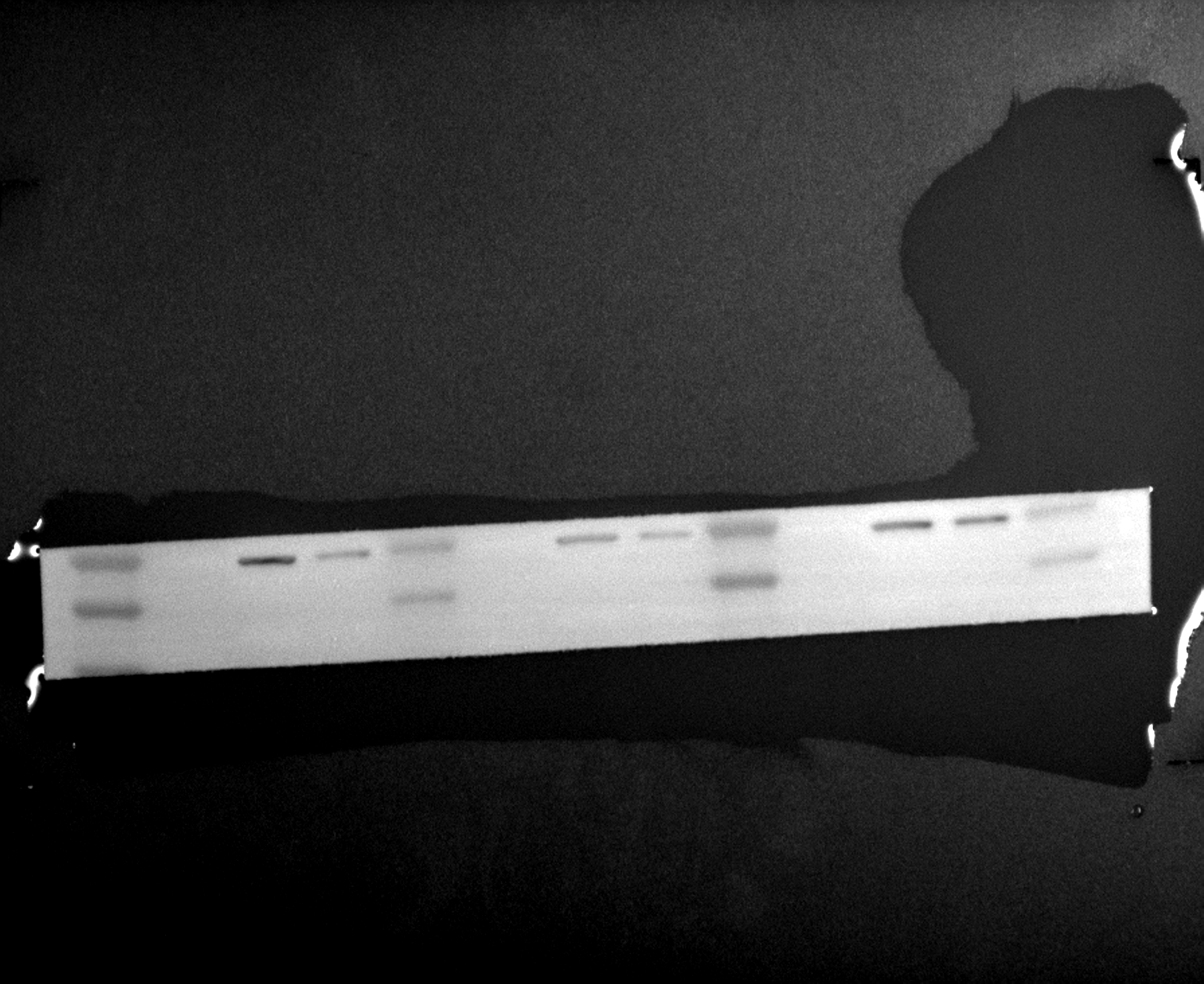

Supplement: Supplementary file 3 — Additional file 3. [file 13287_2026_4964_MOESM3_ESM.zip › Raw WB data 0809/ASC0306小鼠GAPDH+ASC/ASC全膜.Tif]

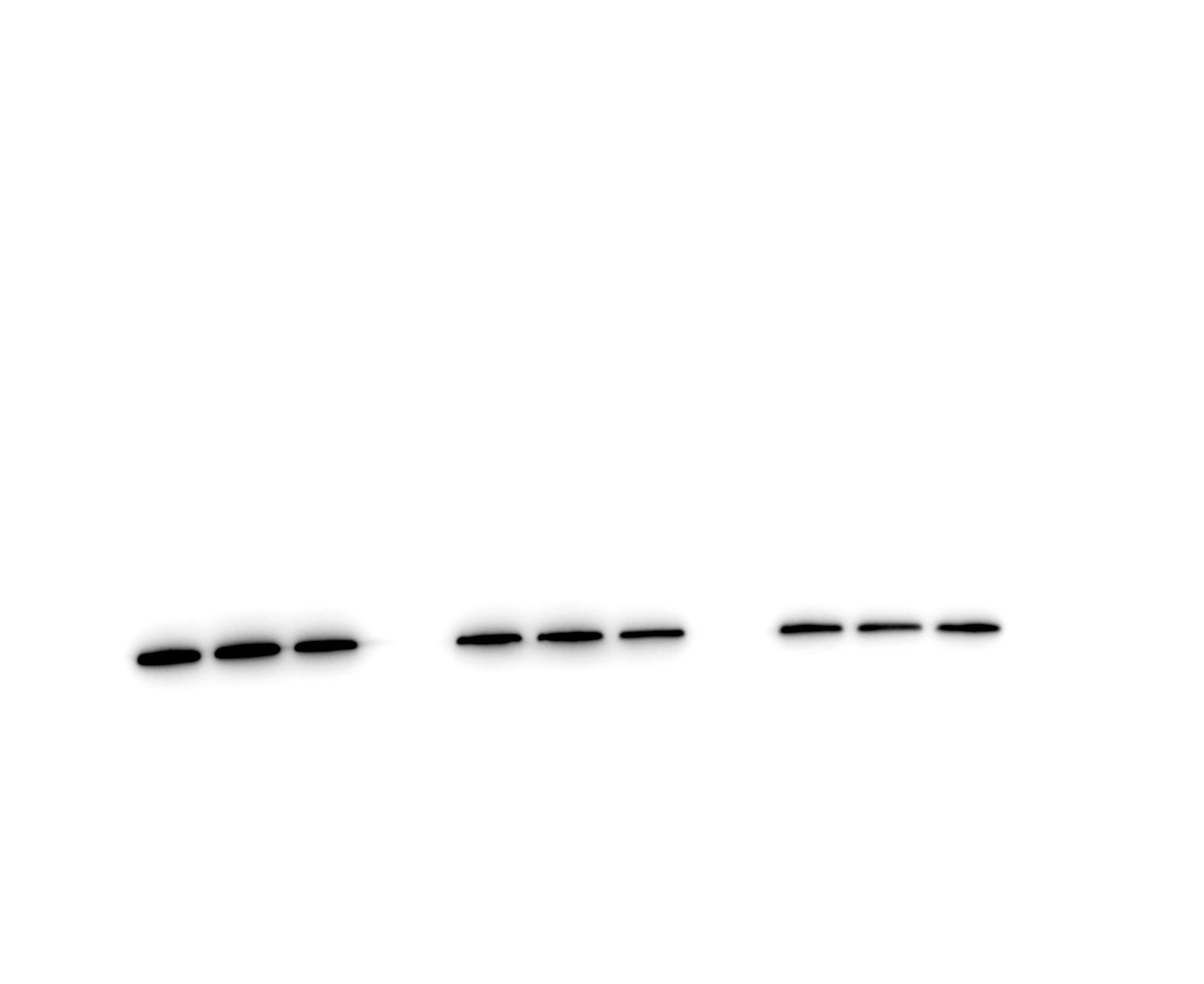

Supplement: Supplementary file 3 — Additional file 3. [file 13287_2026_4964_MOESM3_ESM.zip › Raw WB data 0809/ASC0306小鼠GAPDH+ASC/GAPDH.Tif]

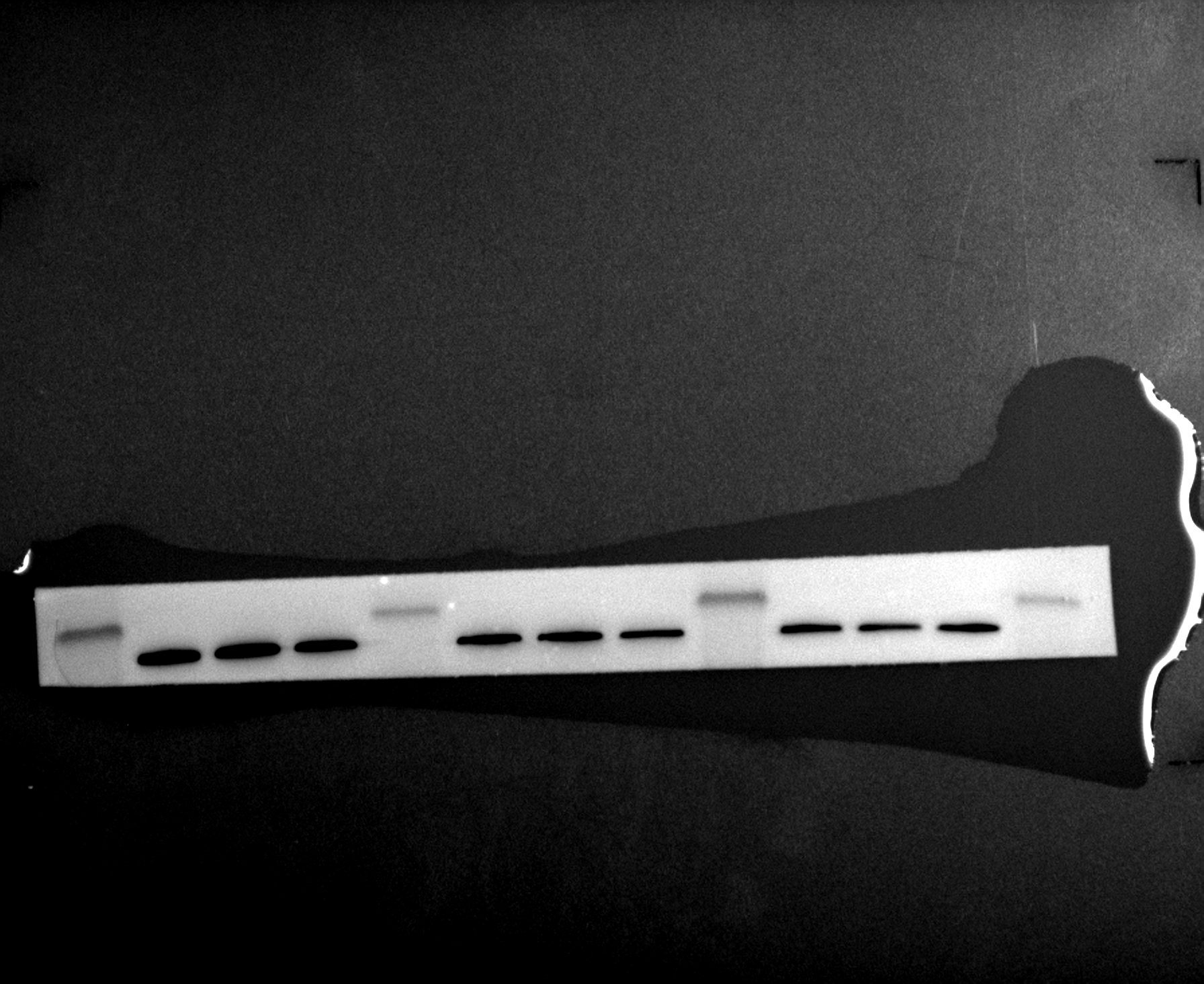

Supplement: Supplementary file 3 — Additional file 3. [file 13287_2026_4964_MOESM3_ESM.zip › Raw WB data 0809/ASC0306小鼠GAPDH+ASC/GAPDH全膜.Tif]

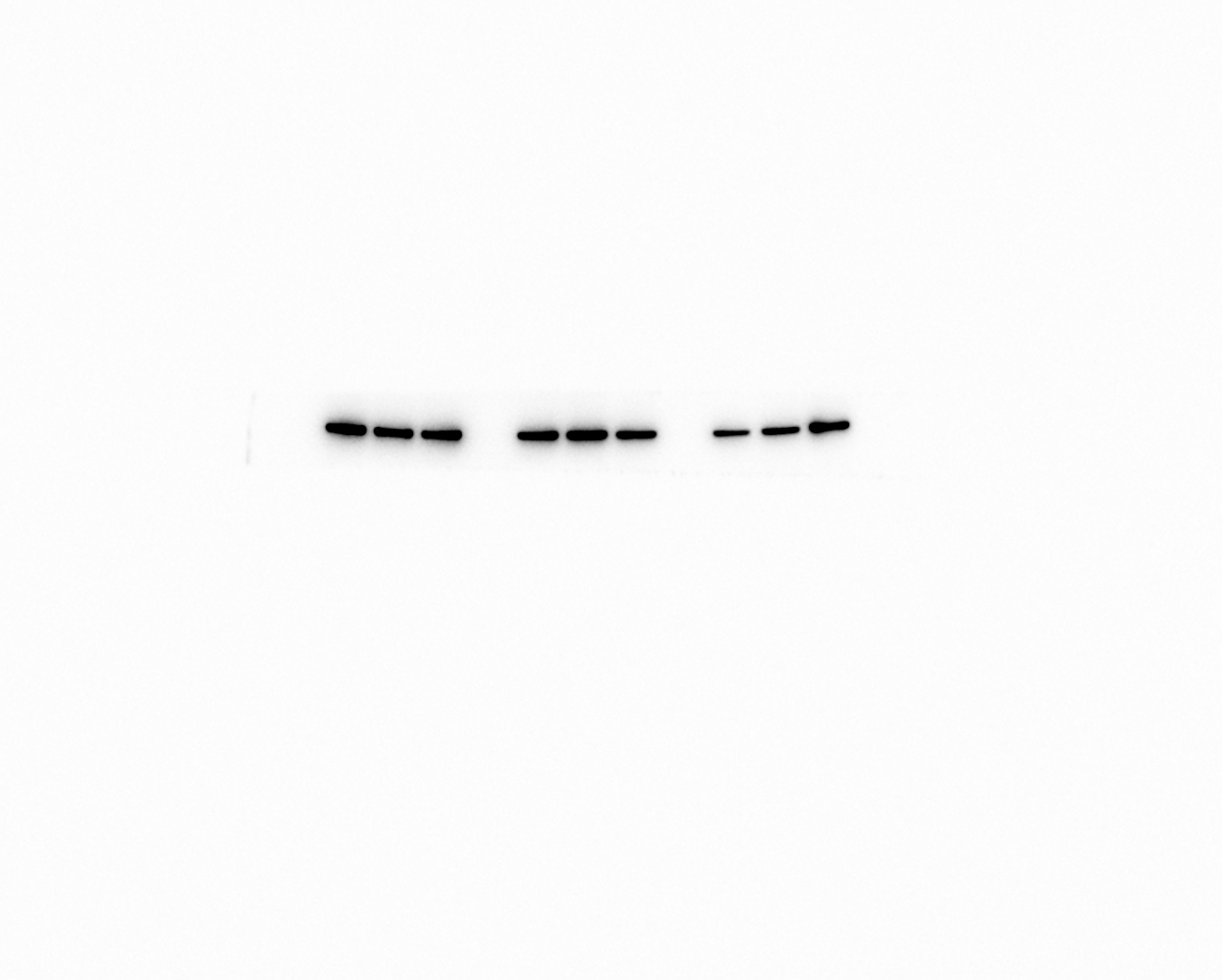

Supplement: Supplementary file 3 — Additional file 3. [file 13287_2026_4964_MOESM3_ESM.zip › Raw WB data 0809/HaCaTASC+NLRP3/GAPDH-200ms.tif]

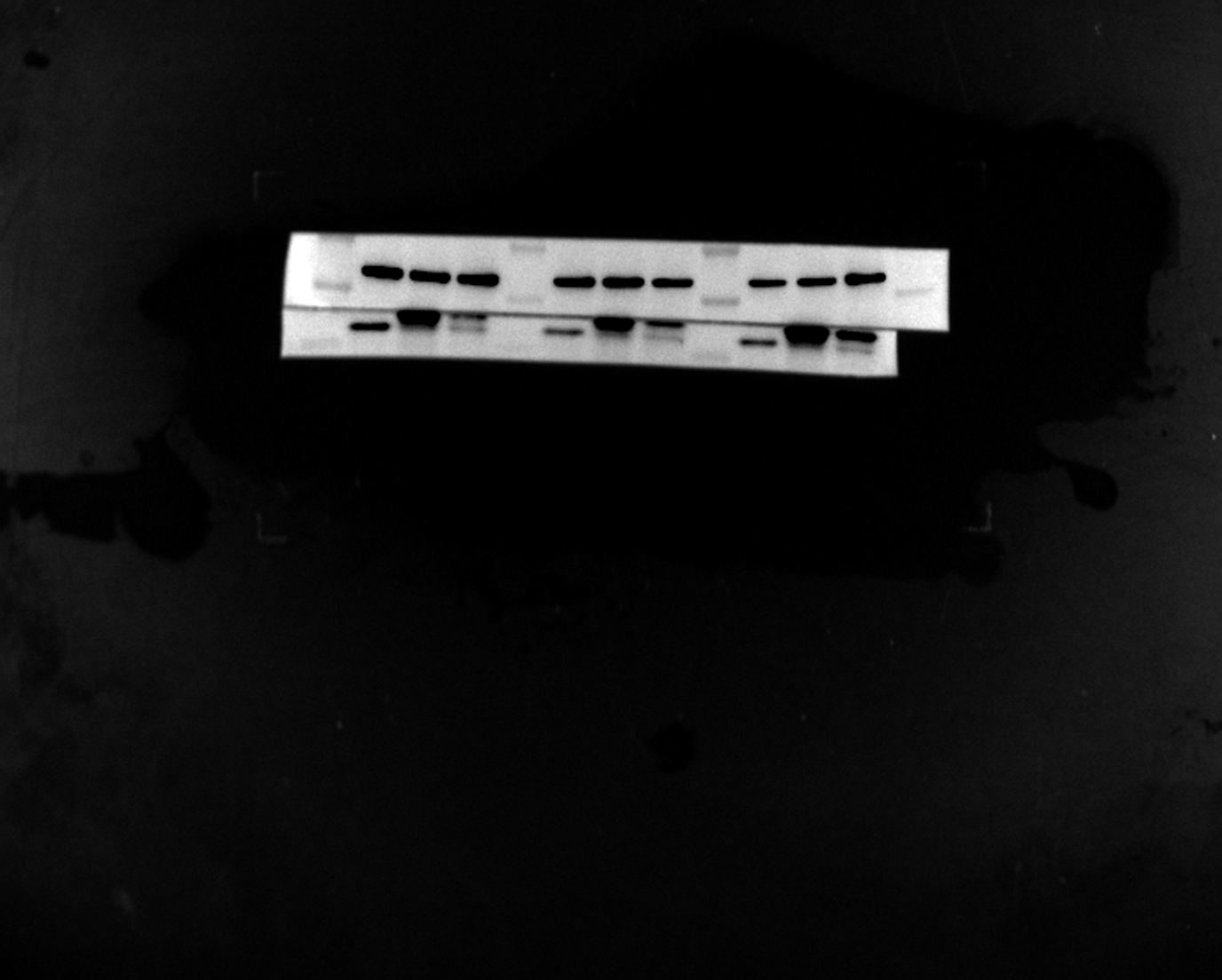

Supplement: Supplementary file 3 — Additional file 3. [file 13287_2026_4964_MOESM3_ESM.zip › Raw WB data 0809/HaCaTASC+NLRP3/gapdh+人ASCquanmo.tif]

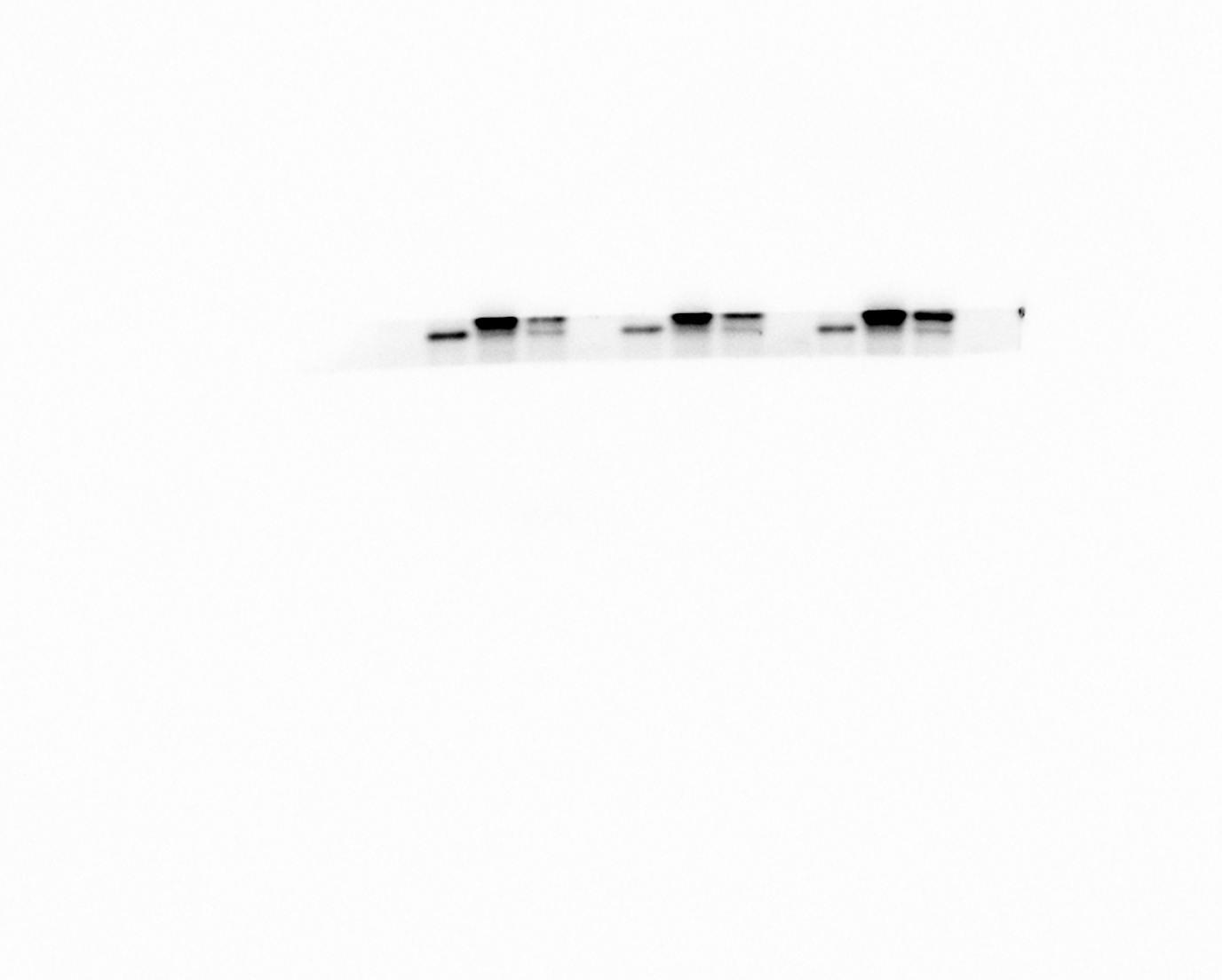

Supplement: Supplementary file 3 — Additional file 3. [file 13287_2026_4964_MOESM3_ESM.zip › Raw WB data 0809/HaCaTASC+NLRP3/人ASC-900ms.tif]

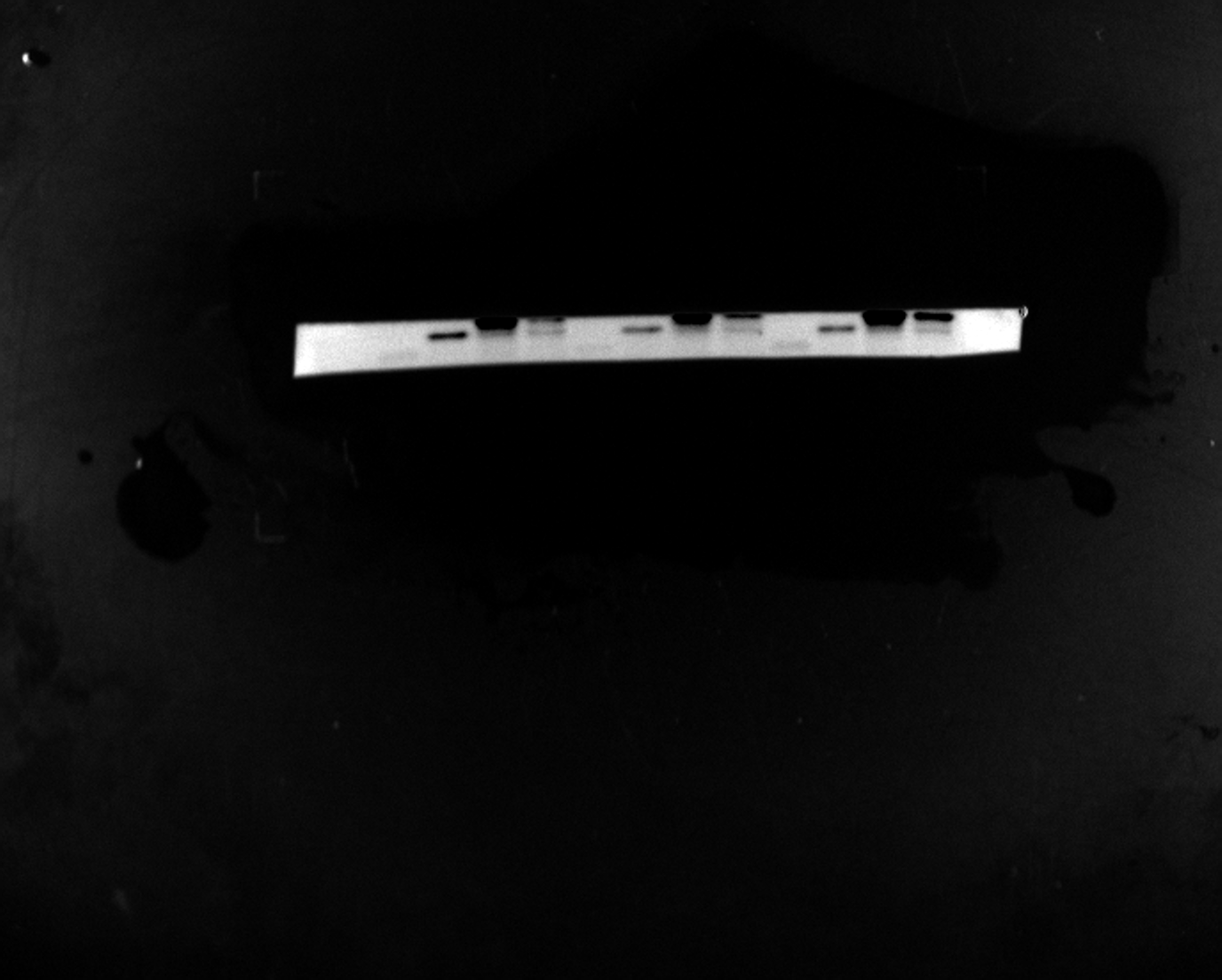

Supplement: Supplementary file 3 — Additional file 3. [file 13287_2026_4964_MOESM3_ESM.zip › Raw WB data 0809/HaCaTASC+NLRP3/人ASC-900ms全膜.tif]

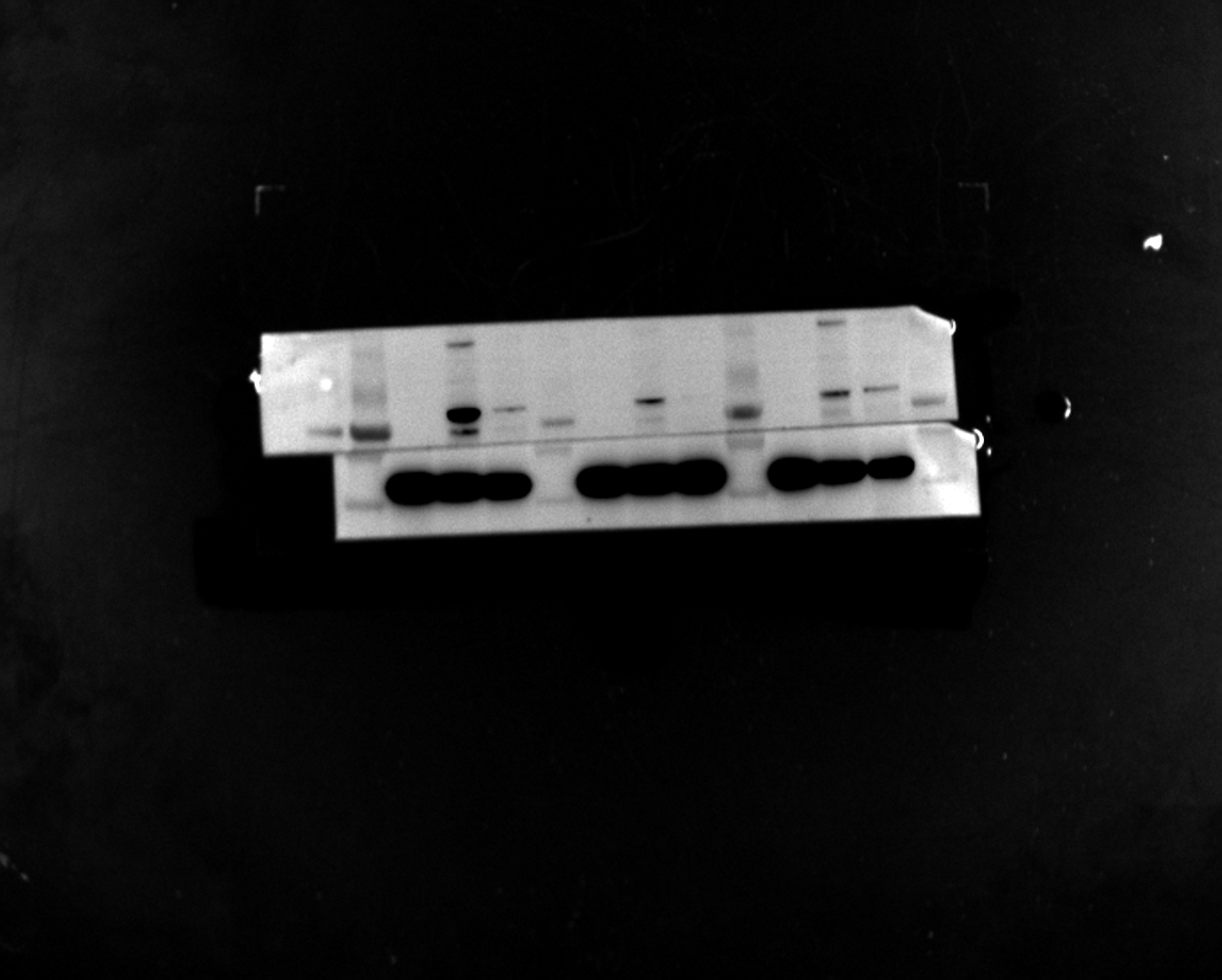

Supplement: Supplementary file 3 — Additional file 3. [file 13287_2026_4964_MOESM3_ESM.zip › Raw WB data 0809/HaCaTASC+NLRP3/全膜人NLRP3+GAPDH.tif]

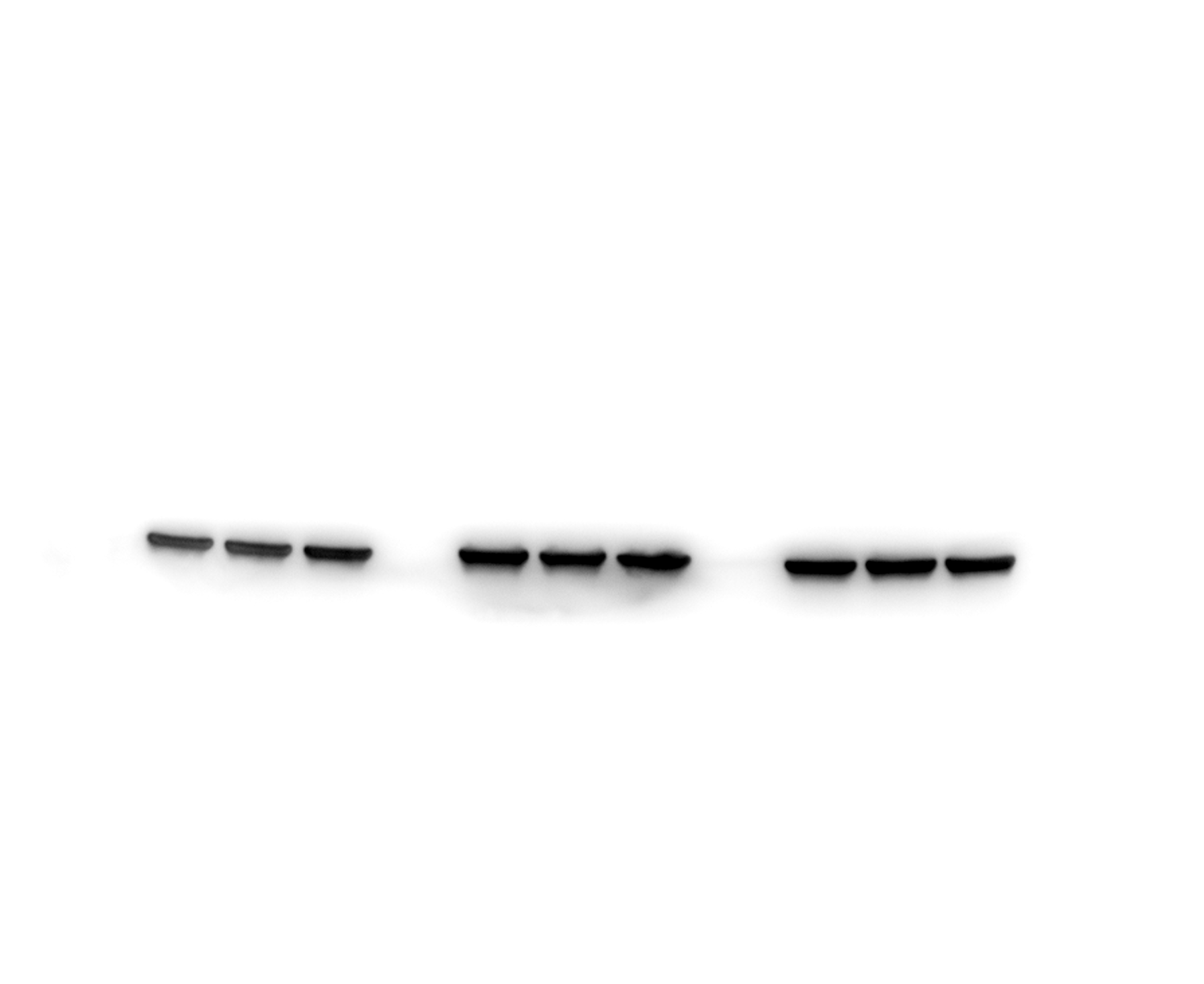

Supplement: Supplementary file 3 — Additional file 3. [file 13287_2026_4964_MOESM3_ESM.zip › Raw WB data 0809/HaCat KRT1+KRT6+MNLRP3+HaCaTNLRP3/GAPDH+ 人KRT60405/GAPDH+100ms.Tif]

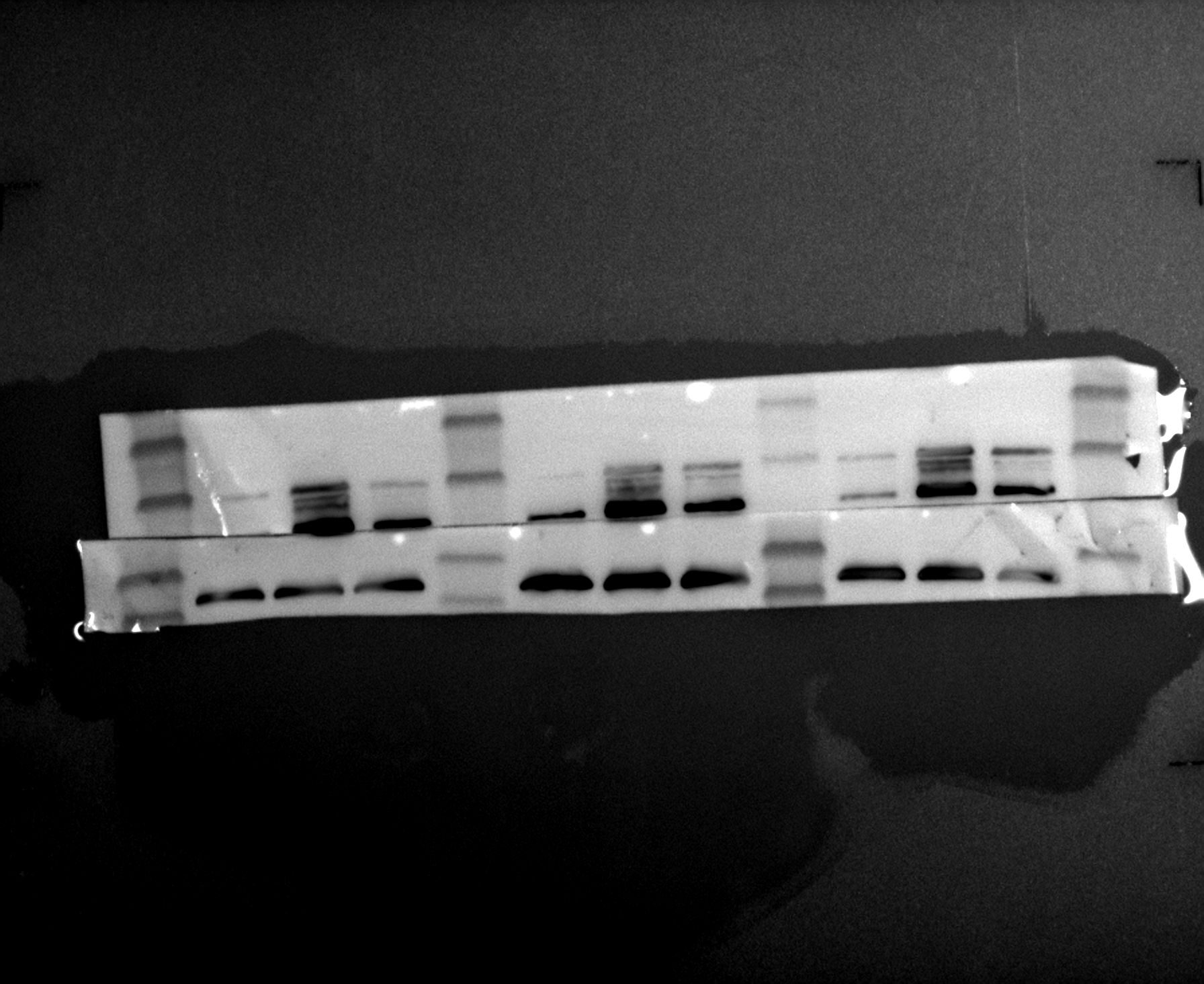

Supplement: Supplementary file 3 — Additional file 3. [file 13287_2026_4964_MOESM3_ESM.zip › Raw WB data 0809/HaCat KRT1+KRT6+MNLRP3+HaCaTNLRP3/GAPDH+ 人KRT60405/GAPDH+KRT6全膜.Tif]

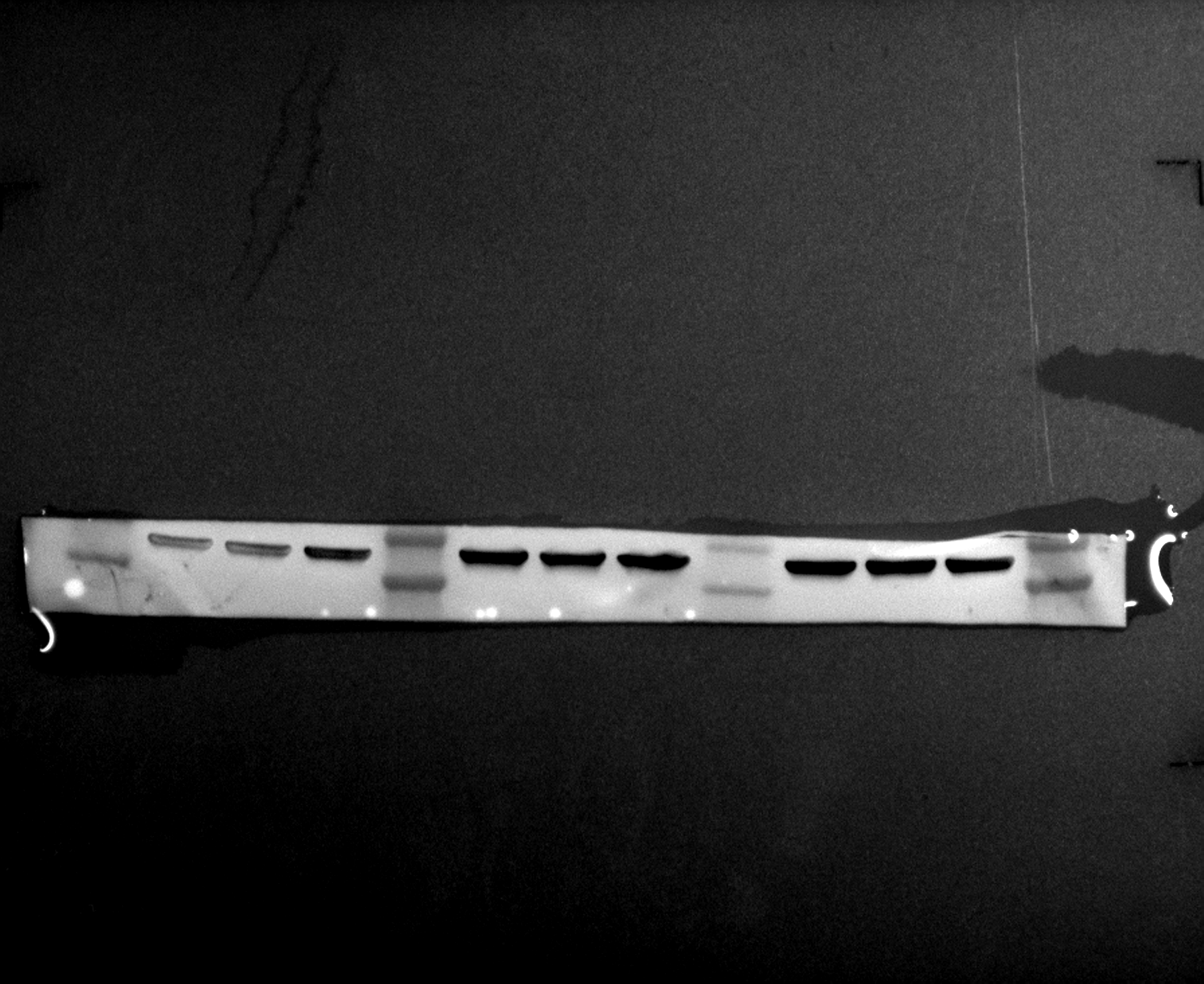

Supplement: Supplementary file 3 — Additional file 3. [file 13287_2026_4964_MOESM3_ESM.zip › Raw WB data 0809/HaCat KRT1+KRT6+MNLRP3+HaCaTNLRP3/GAPDH+ 人KRT60405/GAPDH全膜.Tif]

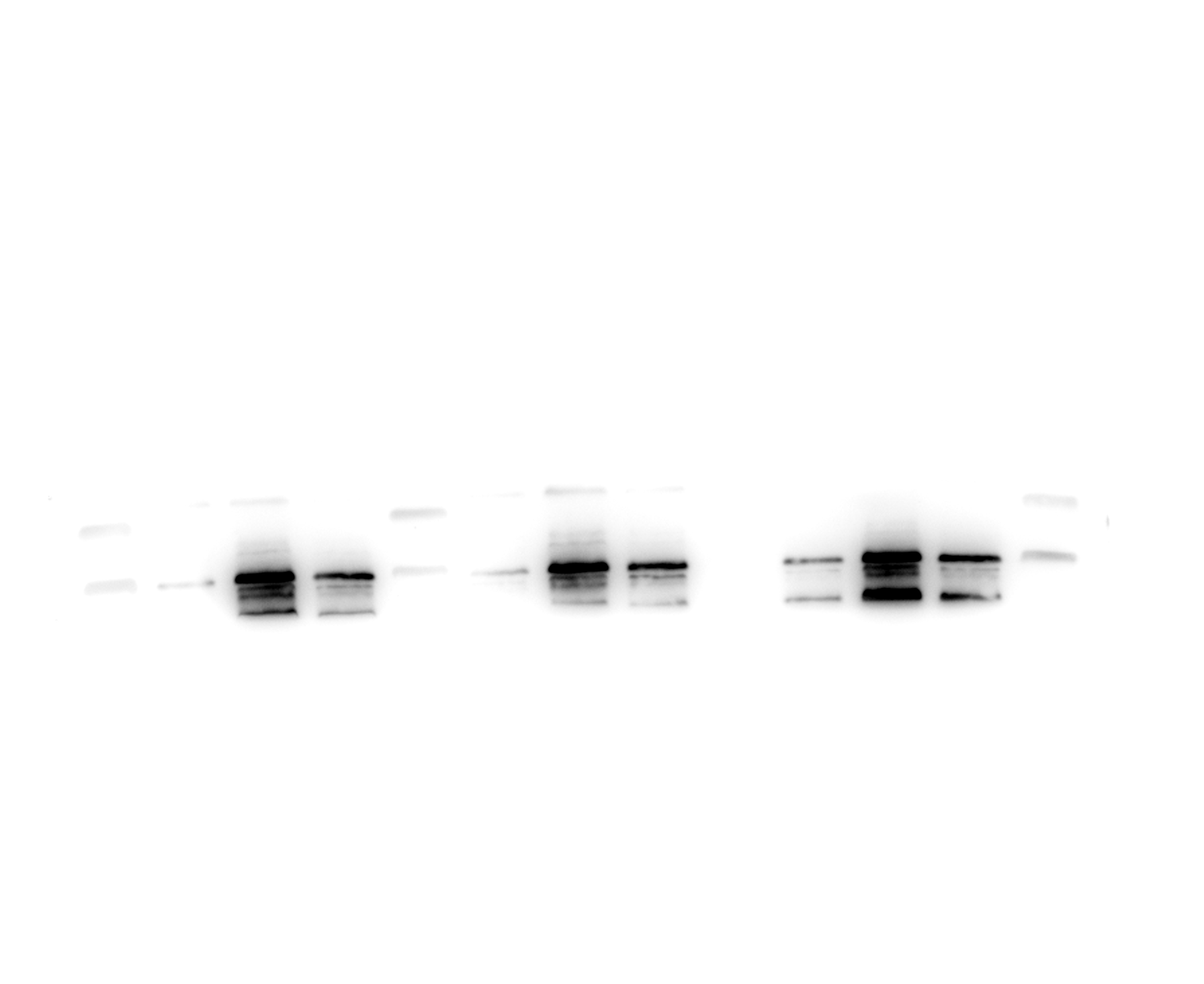

Supplement: Supplementary file 3 — Additional file 3. [file 13287_2026_4964_MOESM3_ESM.zip › Raw WB data 0809/HaCat KRT1+KRT6+MNLRP3+HaCaTNLRP3/GAPDH+ 人KRT60405/KRT6+300MS.Tif]

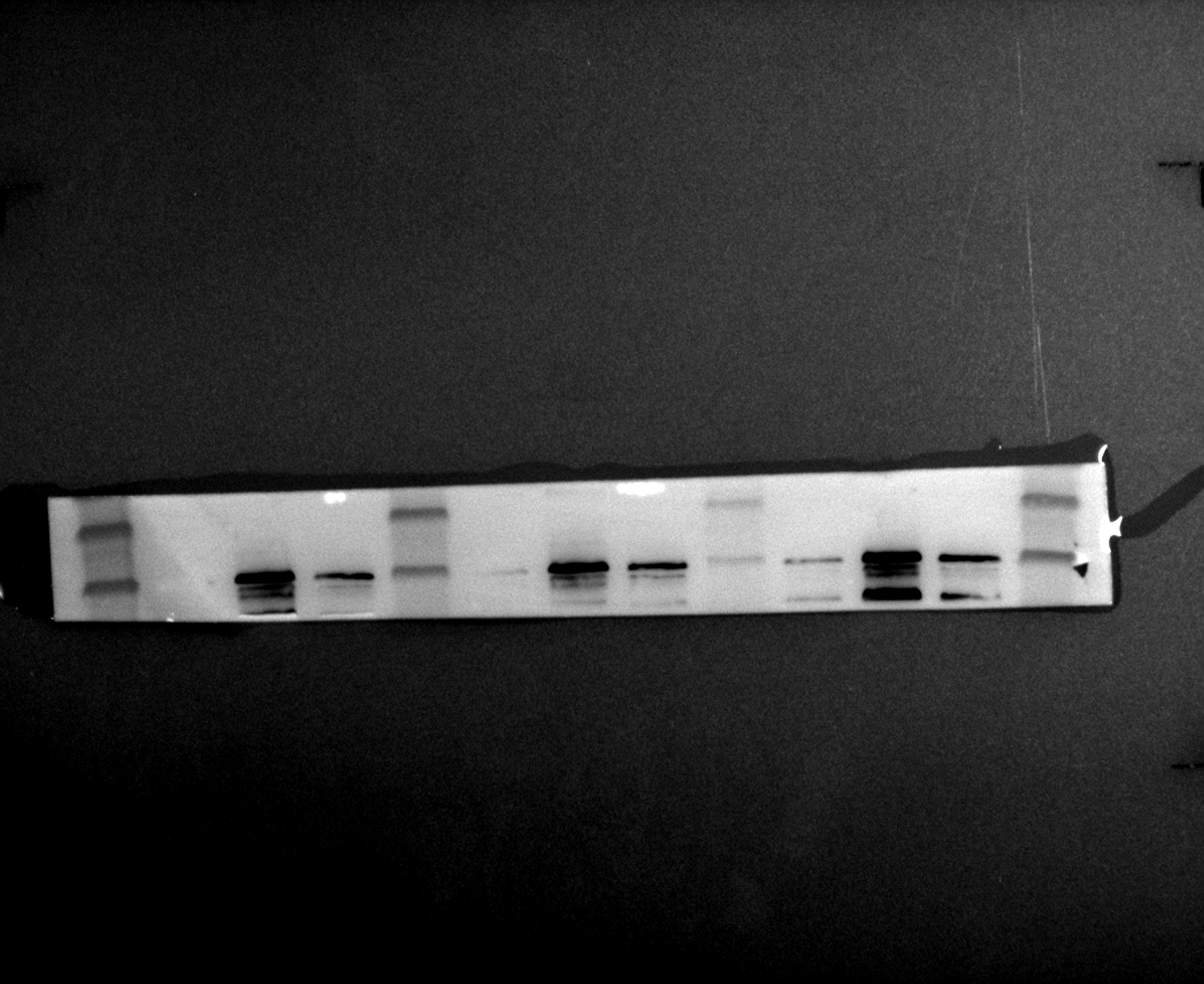

Supplement: Supplementary file 3 — Additional file 3. [file 13287_2026_4964_MOESM3_ESM.zip › Raw WB data 0809/HaCat KRT1+KRT6+MNLRP3+HaCaTNLRP3/GAPDH+ 人KRT60405/KRT6全膜.Tif]

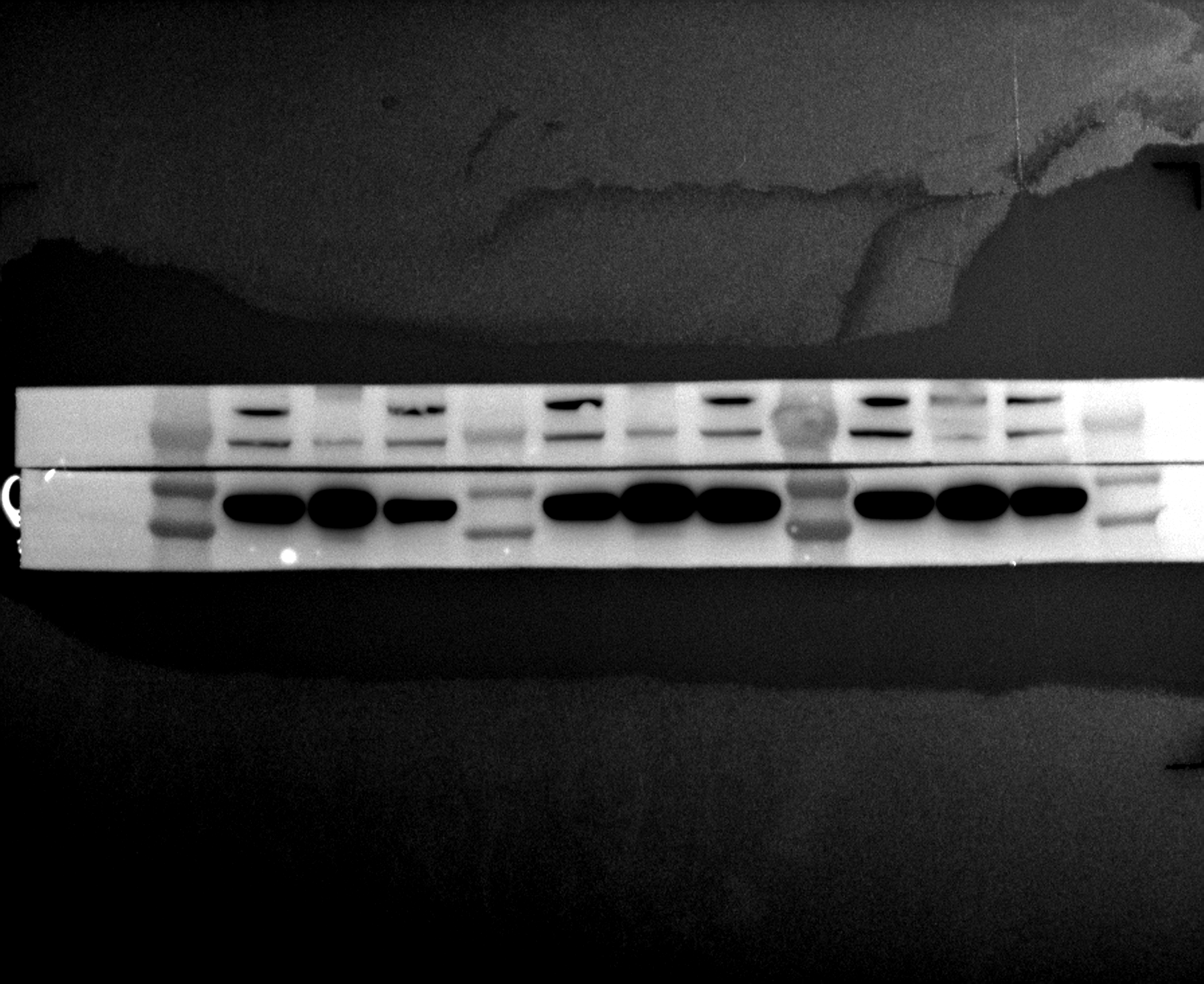

Supplement: Supplementary file 3 — Additional file 3. [file 13287_2026_4964_MOESM3_ESM.zip › Raw WB data 0809/HaCat KRT1+KRT6+MNLRP3+HaCaTNLRP3/GAPDH+KRT1REN/GAPDH+KRT1.Tif]

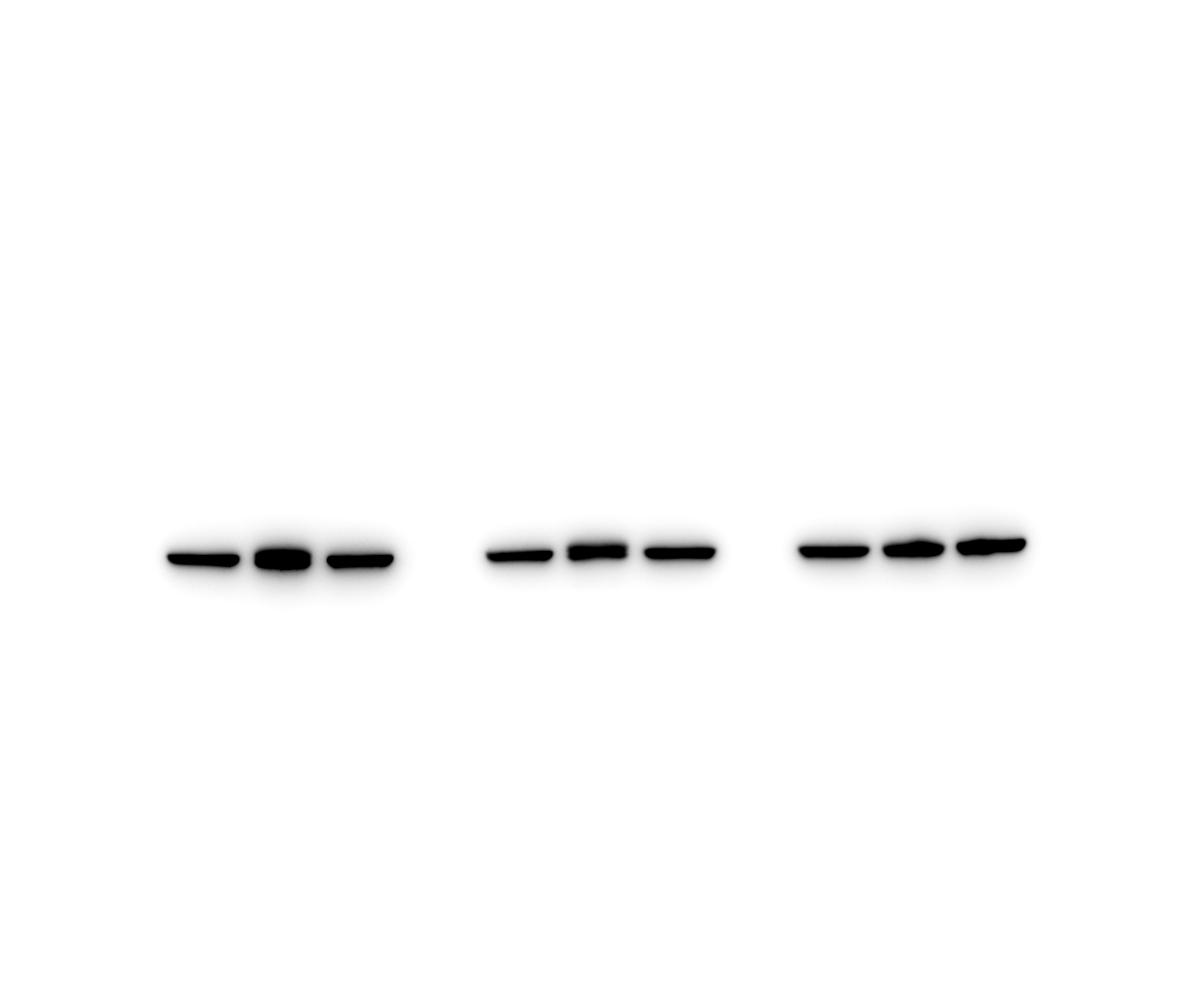

Supplement: Supplementary file 3 — Additional file 3. [file 13287_2026_4964_MOESM3_ESM.zip › Raw WB data 0809/HaCat KRT1+KRT6+MNLRP3+HaCaTNLRP3/GAPDH+KRT1REN/GAPDH.Tif]

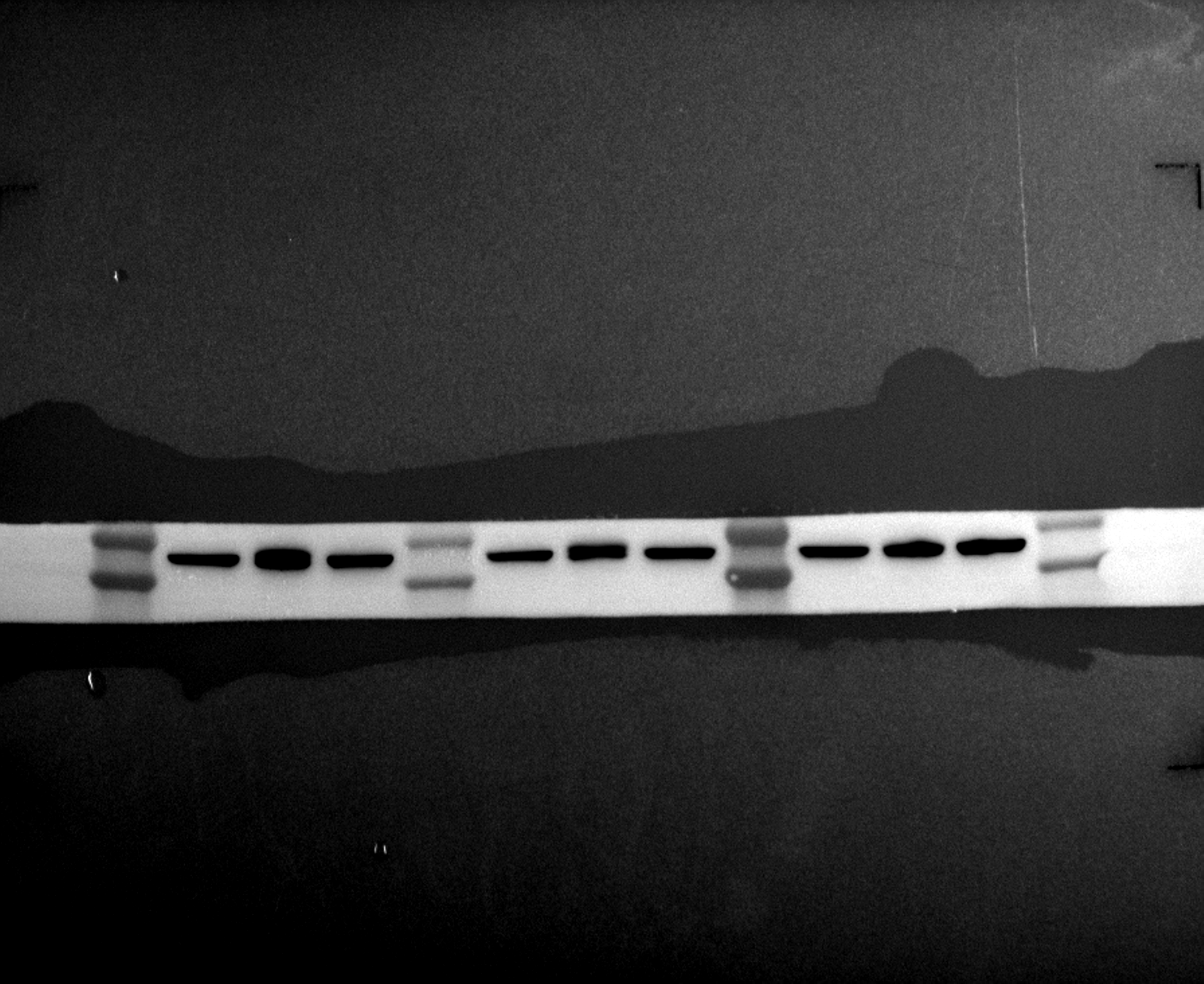

Supplement: Supplementary file 3 — Additional file 3. [file 13287_2026_4964_MOESM3_ESM.zip › Raw WB data 0809/HaCat KRT1+KRT6+MNLRP3+HaCaTNLRP3/GAPDH+KRT1REN/GAPDH全膜.Tif]

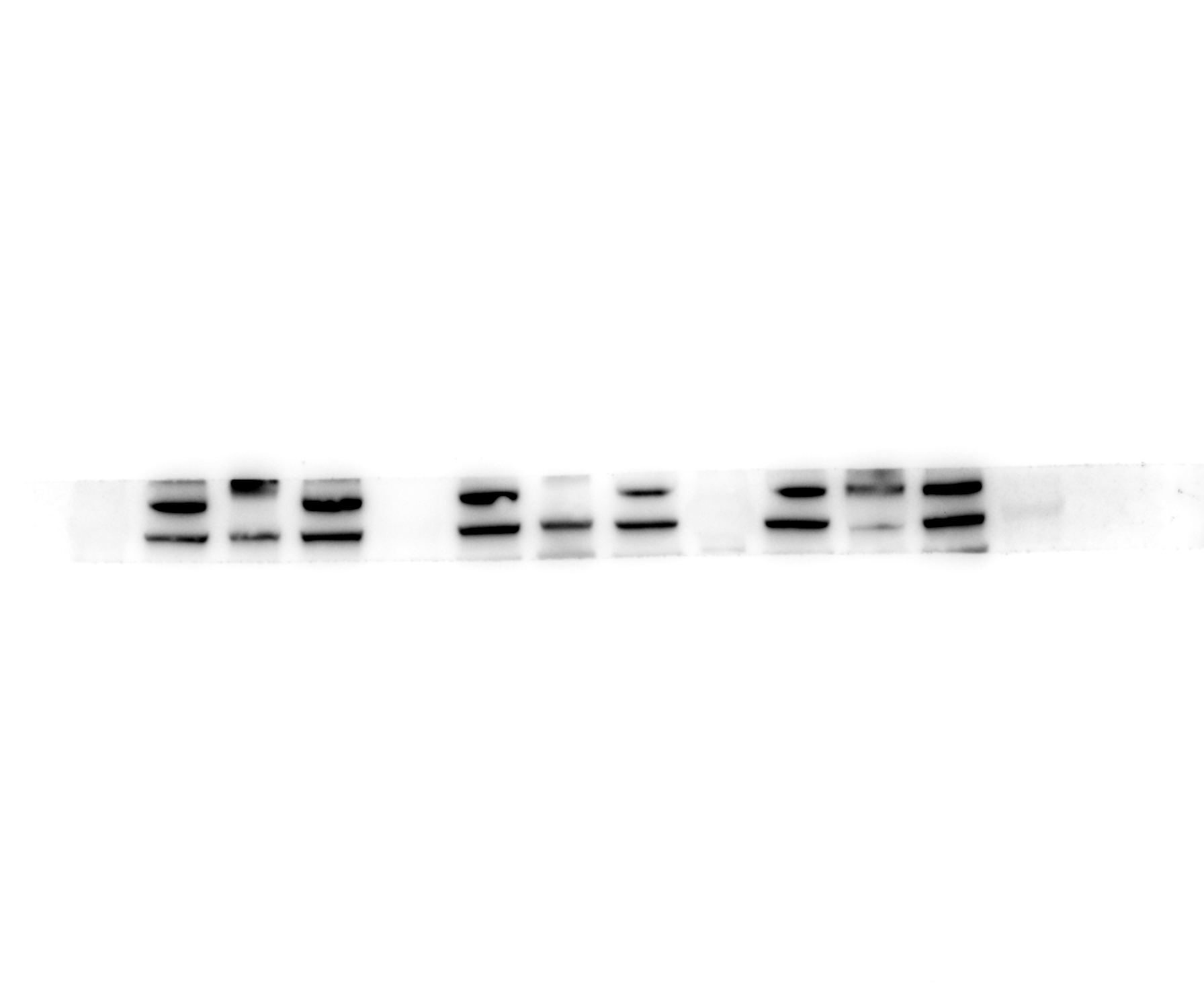

Supplement: Supplementary file 3 — Additional file 3. [file 13287_2026_4964_MOESM3_ESM.zip › Raw WB data 0809/HaCat KRT1+KRT6+MNLRP3+HaCaTNLRP3/GAPDH+KRT1REN/KRT1-300ms.Tif]

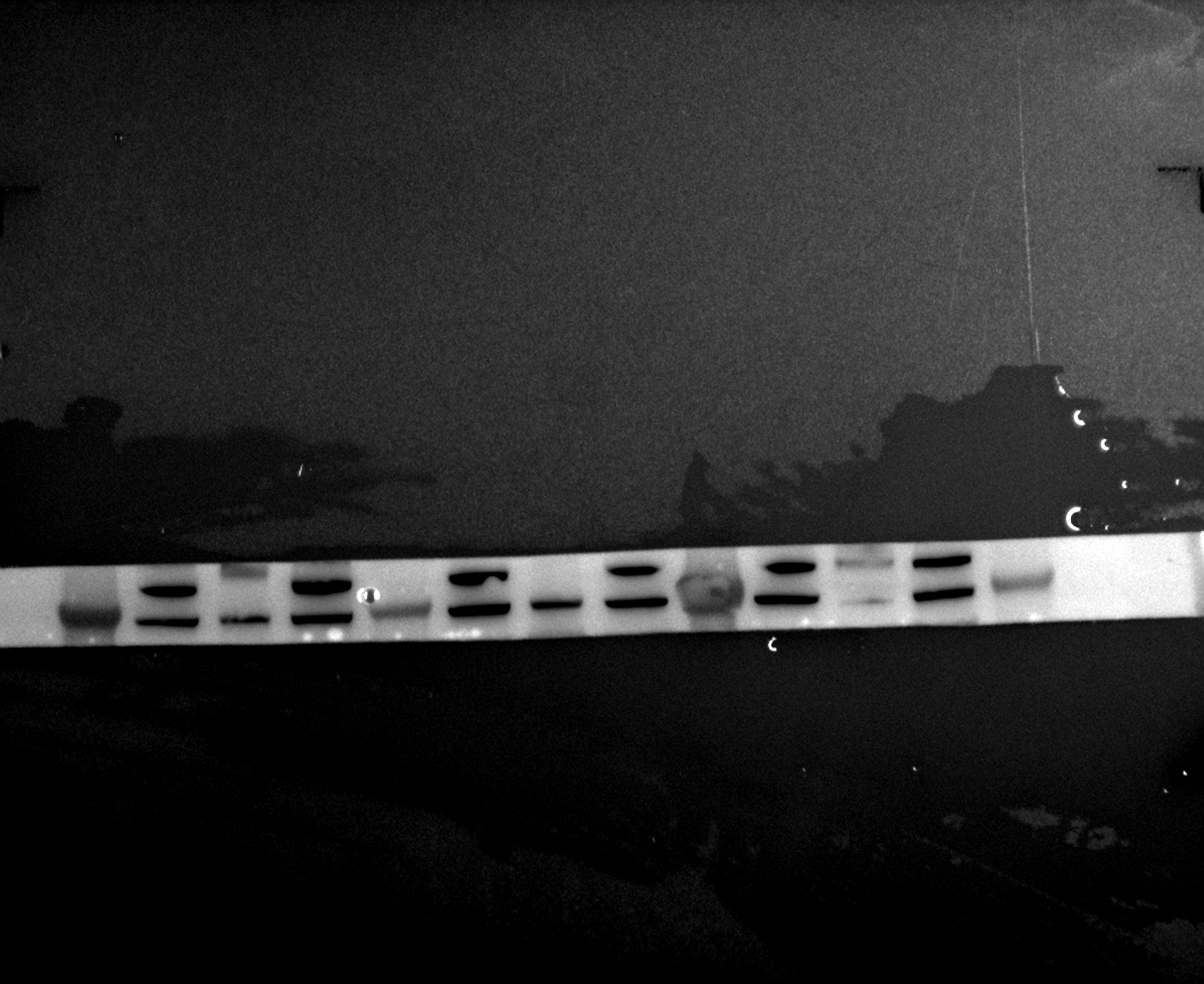

Supplement: Supplementary file 3 — Additional file 3. [file 13287_2026_4964_MOESM3_ESM.zip › Raw WB data 0809/HaCat KRT1+KRT6+MNLRP3+HaCaTNLRP3/GAPDH+KRT1REN/KRT1全膜.Tif]

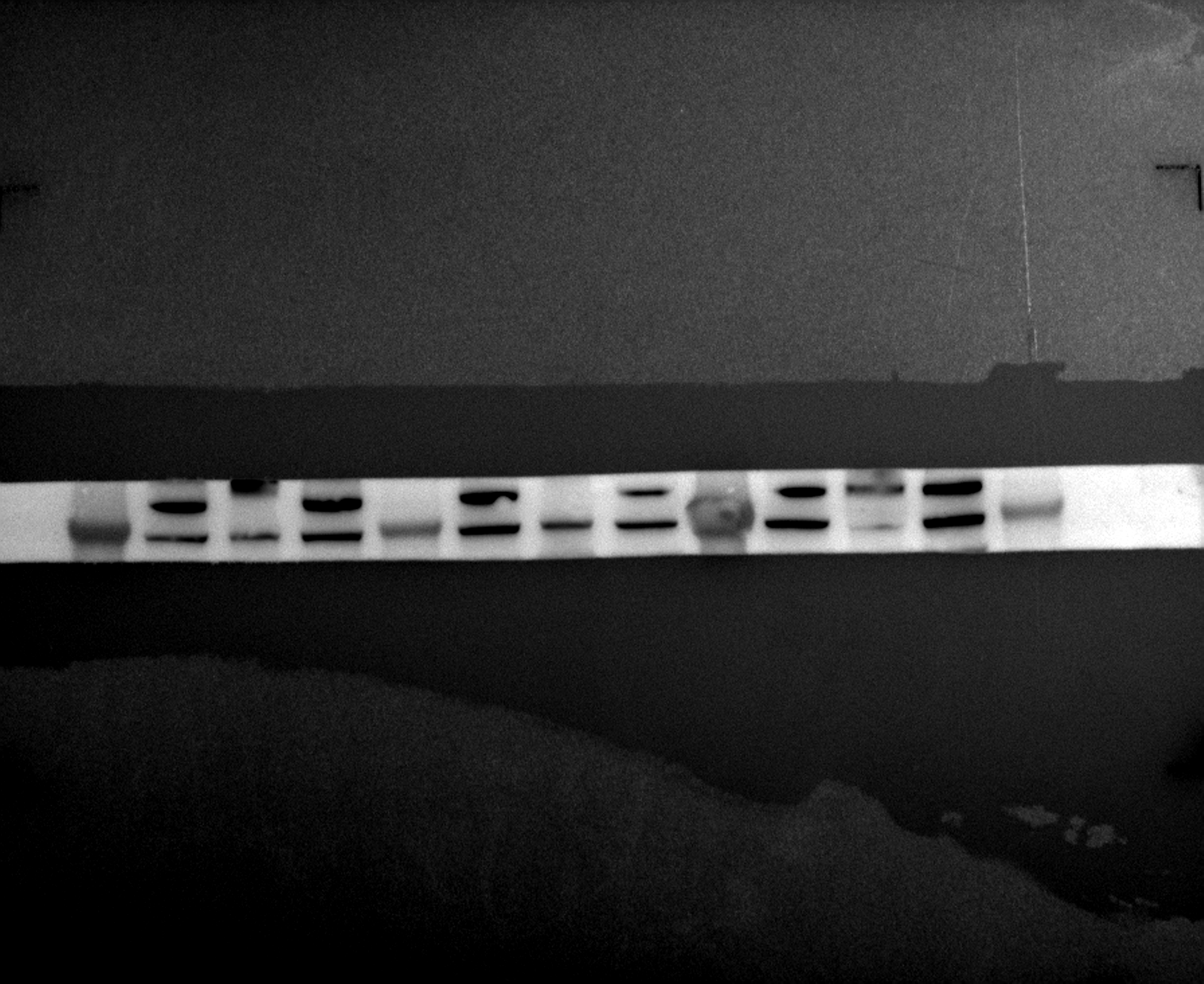

Supplement: Supplementary file 3 — Additional file 3. [file 13287_2026_4964_MOESM3_ESM.zip › Raw WB data 0809/HaCat KRT1+KRT6+MNLRP3+HaCaTNLRP3/GAPDH+KRT1REN/KRT1全膜2.Tif]

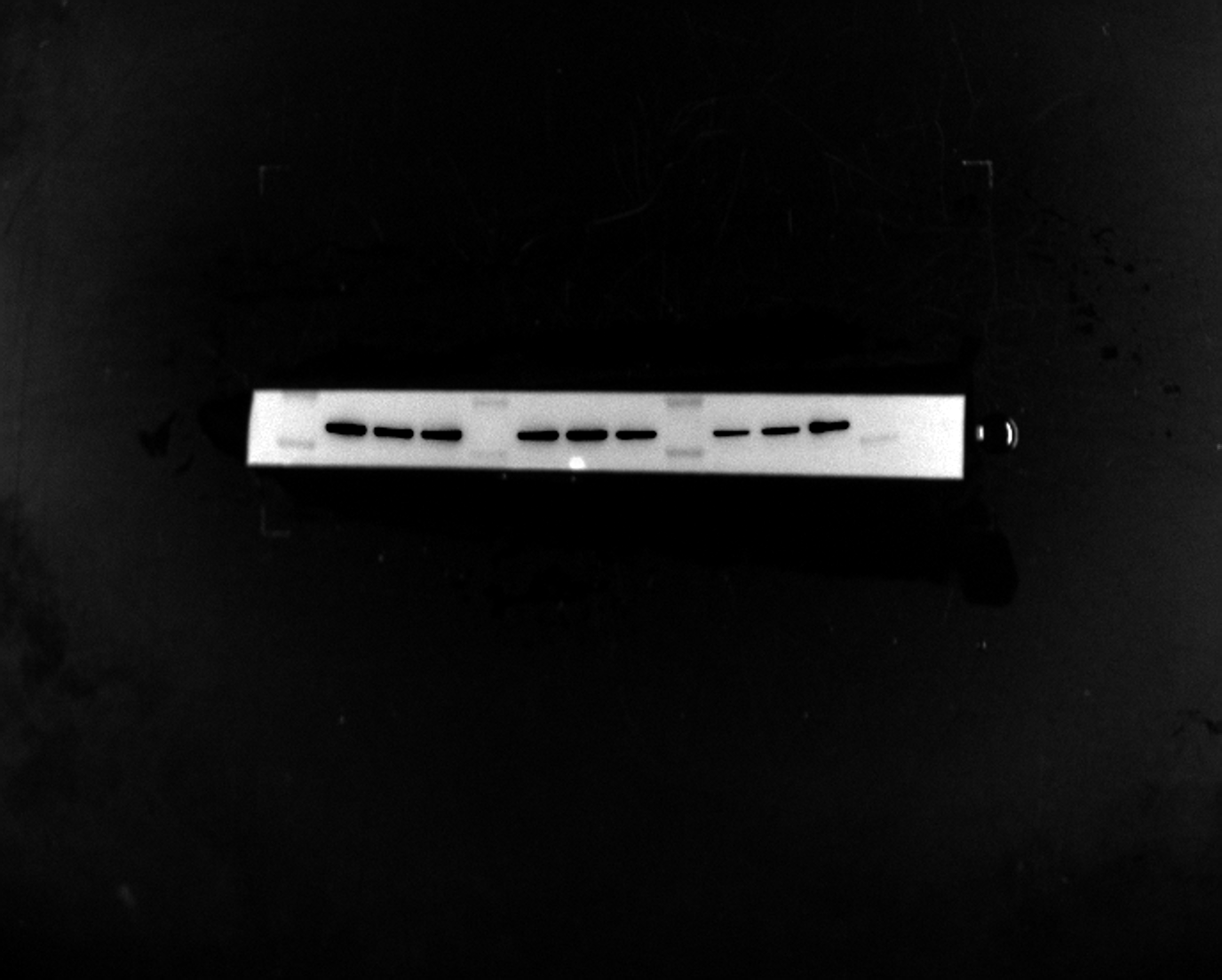

Supplement: Supplementary file 3 — Additional file 3. [file 13287_2026_4964_MOESM3_ESM.zip › Raw WB data 0809/HaCat KRT1+KRT6+MNLRP3+HaCaTNLRP3/人ASC0307/GAPDHquanmo.tif]

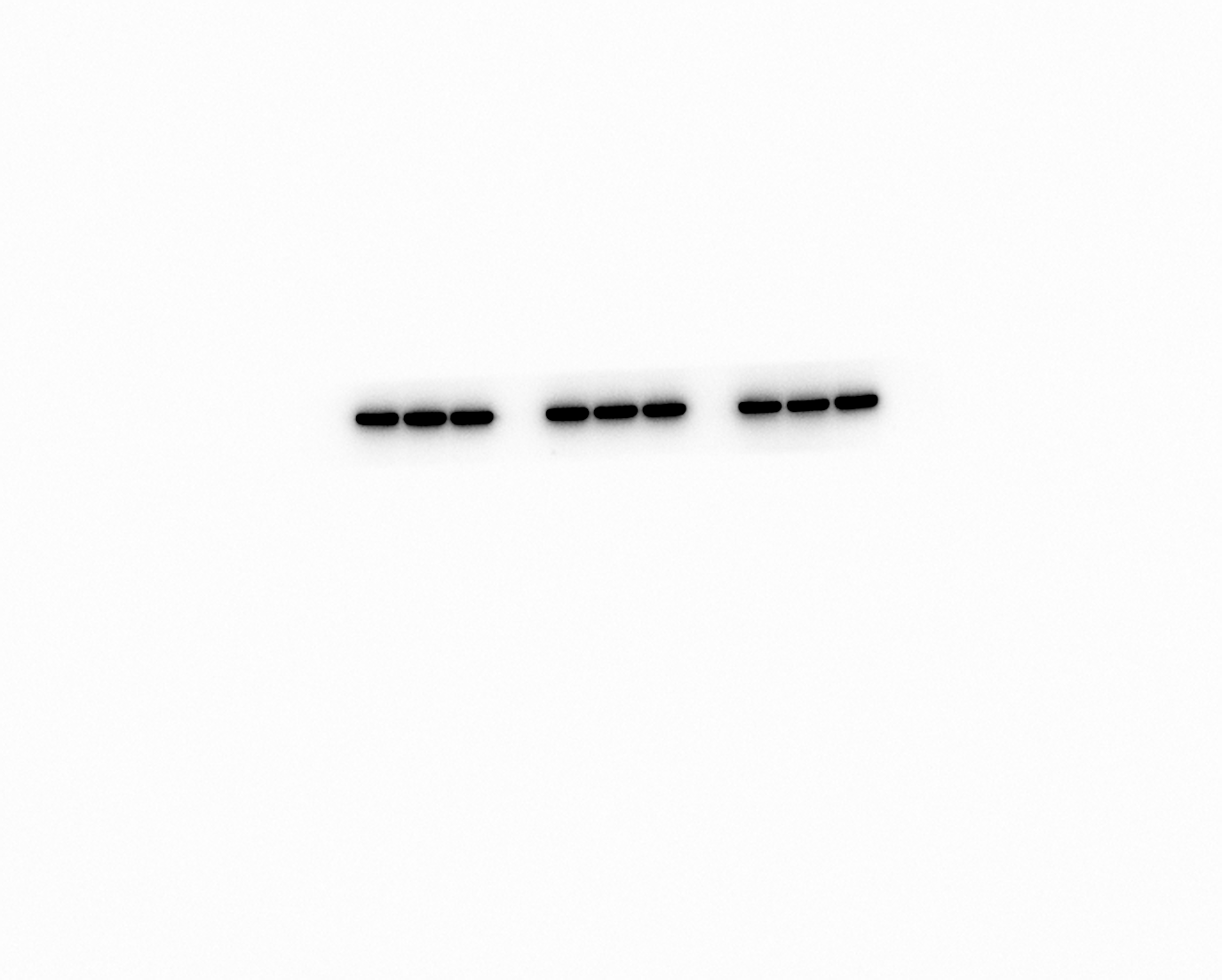

Supplement: Supplementary file 3 — Additional file 3. [file 13287_2026_4964_MOESM3_ESM.zip › Raw WB data 0809/HaCat KRT1+KRT6+MNLRP3+HaCaTNLRP3/小鼠NLRP3/GAPDH+100MS.tif]

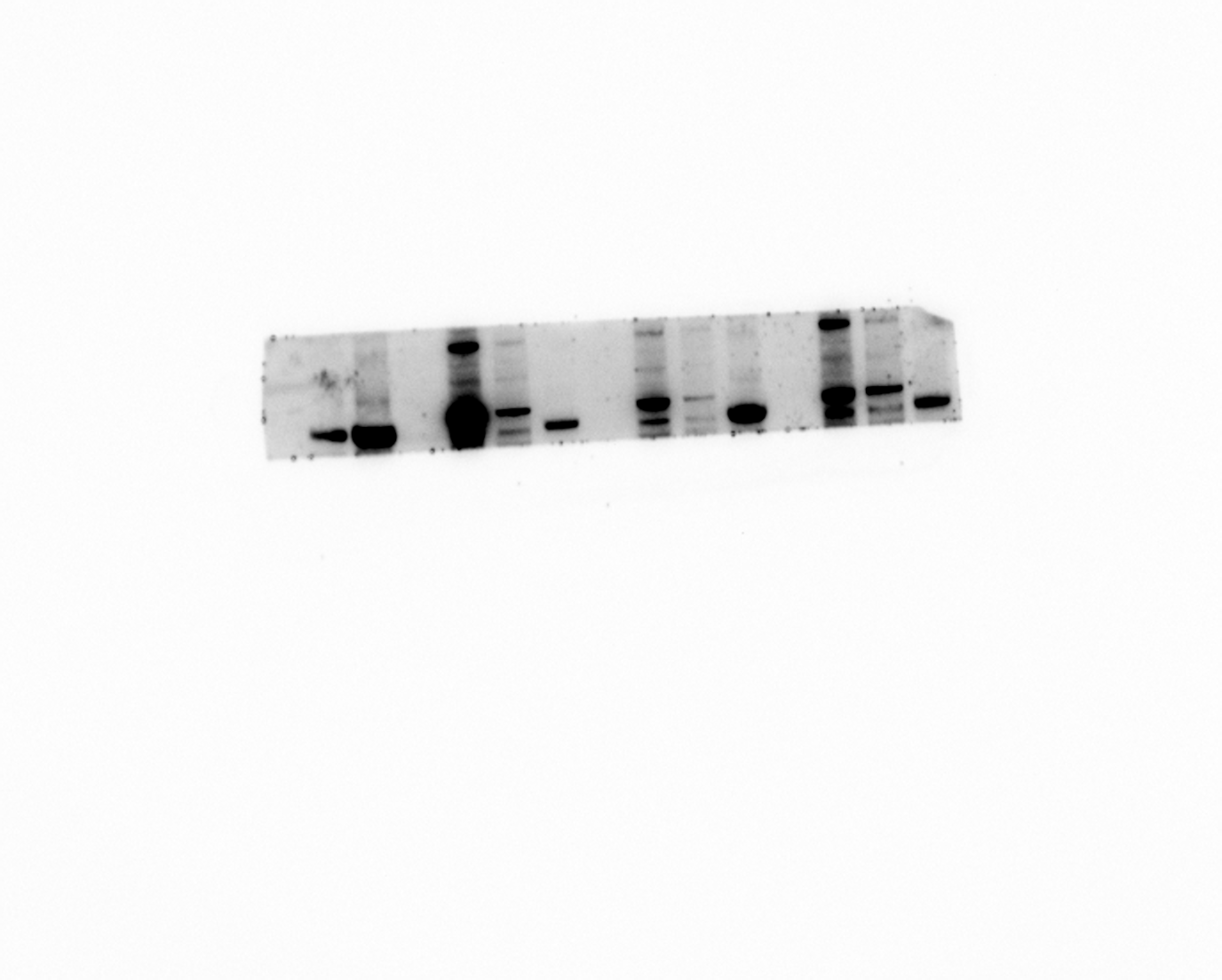

Supplement: Supplementary file 3 — Additional file 3. [file 13287_2026_4964_MOESM3_ESM.zip › Raw WB data 0809/HaCat KRT1+KRT6+MNLRP3+HaCaTNLRP3/小鼠NLRP3/NLRP3+500ms.tif]

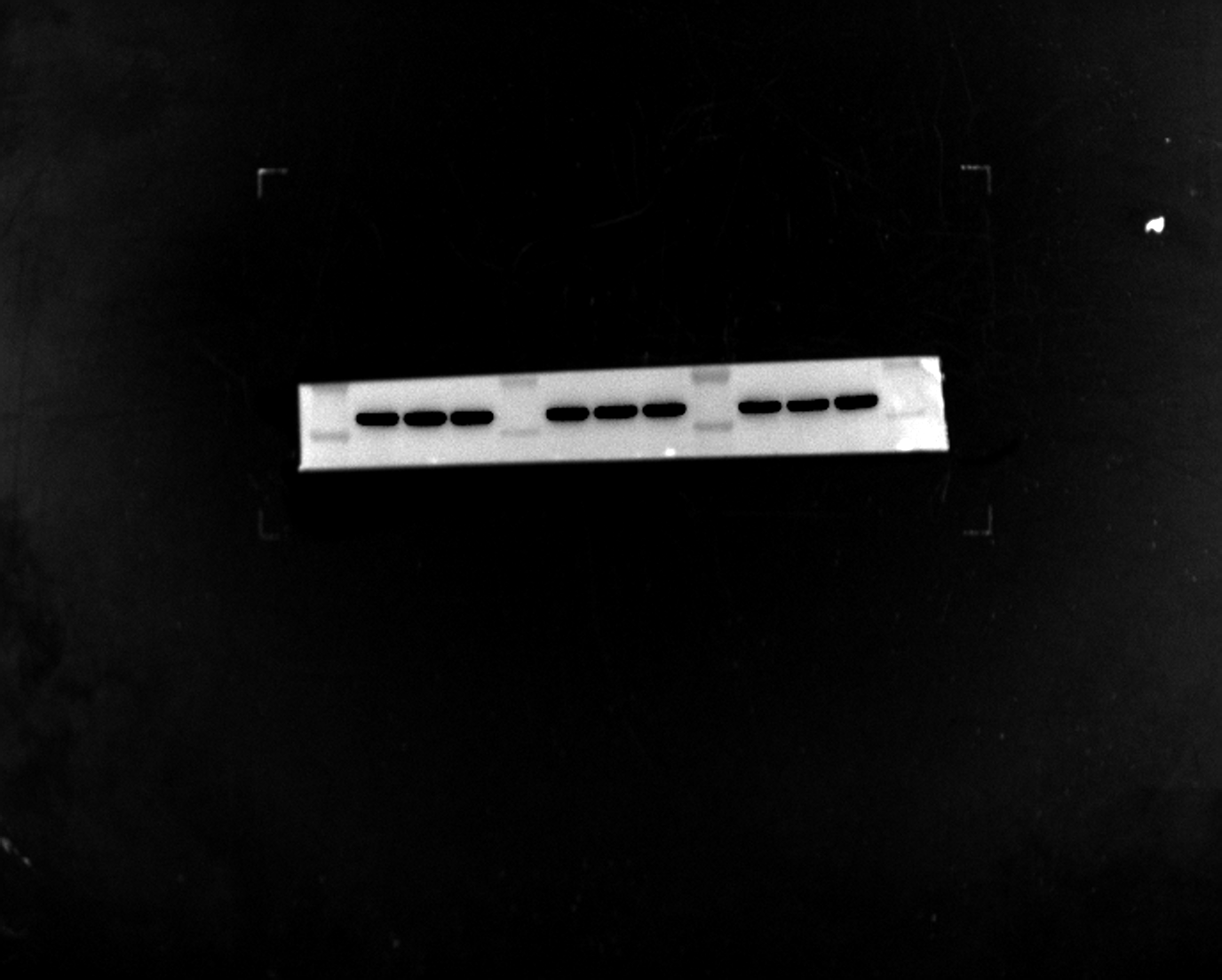

Supplement: Supplementary file 3 — Additional file 3. [file 13287_2026_4964_MOESM3_ESM.zip › Raw WB data 0809/HaCat KRT1+KRT6+MNLRP3+HaCaTNLRP3/小鼠NLRP3/全膜GAPDH.tif]

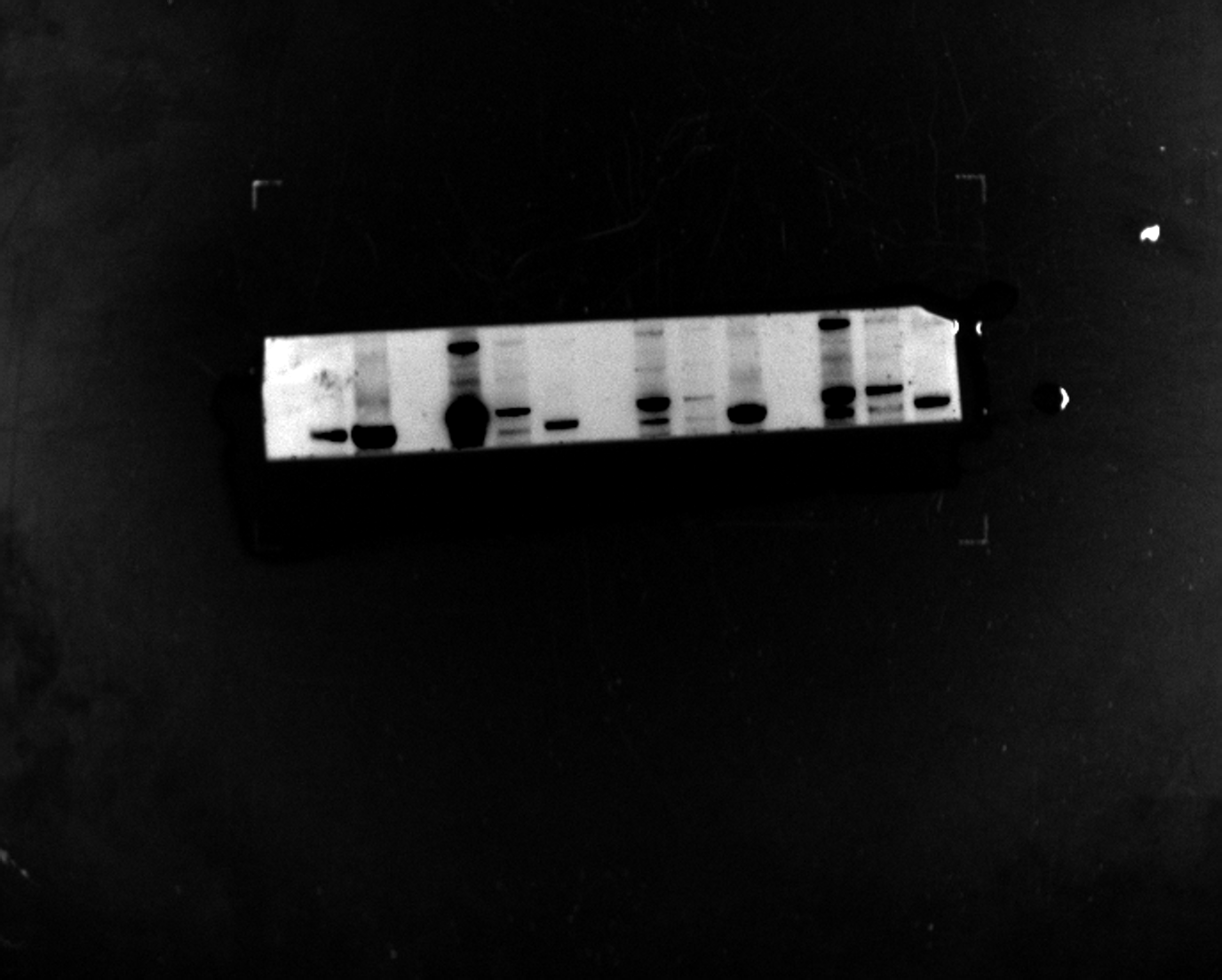

Supplement: Supplementary file 3 — Additional file 3. [file 13287_2026_4964_MOESM3_ESM.zip › Raw WB data 0809/HaCat KRT1+KRT6+MNLRP3+HaCaTNLRP3/小鼠NLRP3/全膜NLRP3.tif]

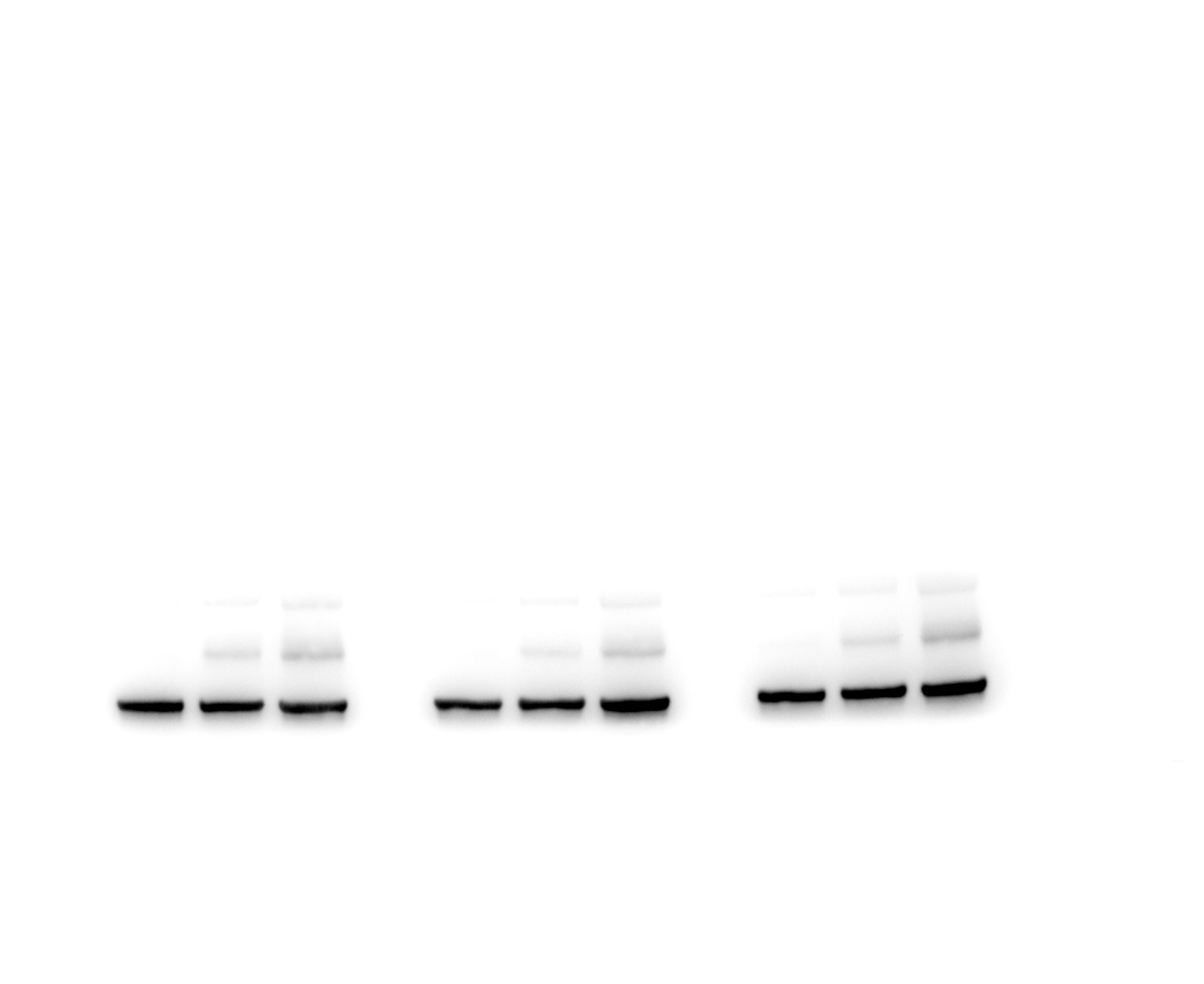

Supplement: Supplementary file 3 — Additional file 3. [file 13287_2026_4964_MOESM3_ESM.zip › Raw WB data 0809/M GAPDH+LC3B+P62X3+PINK1X1/ACTIN.Tif]

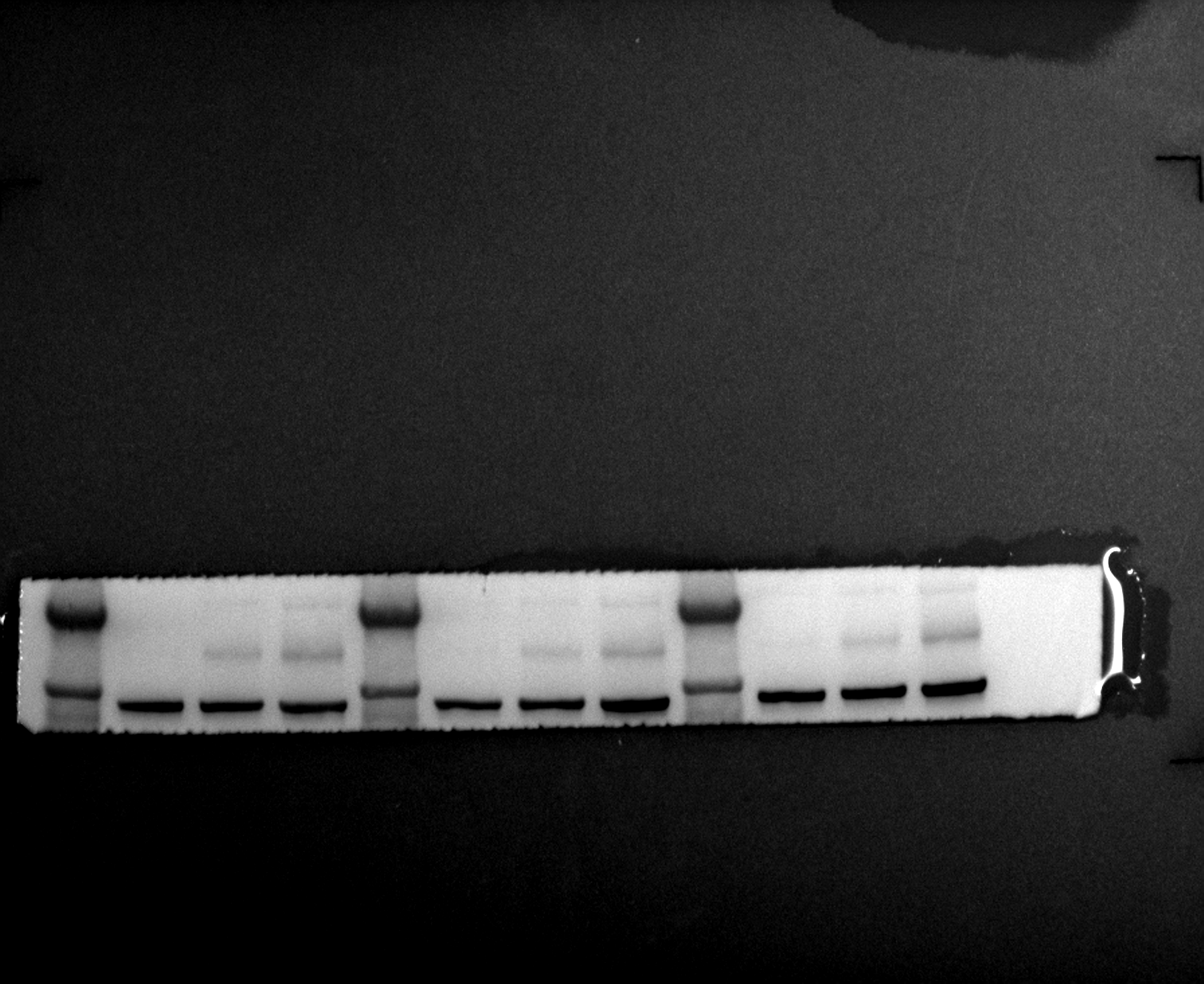

Supplement: Supplementary file 3 — Additional file 3. [file 13287_2026_4964_MOESM3_ESM.zip › Raw WB data 0809/M GAPDH+LC3B+P62X3+PINK1X1/ACTINQUANMO.Tif]

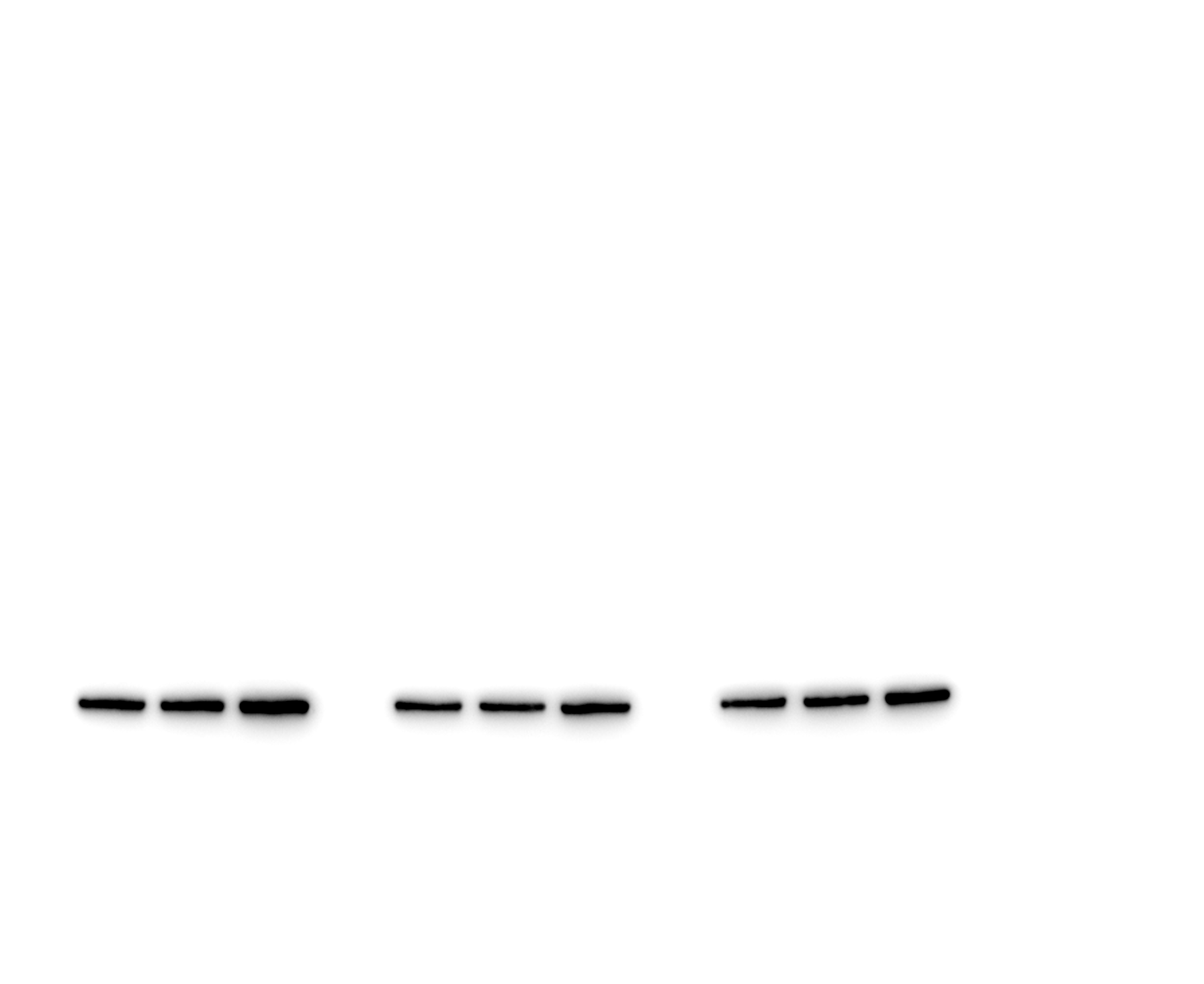

Supplement: Supplementary file 3 — Additional file 3. [file 13287_2026_4964_MOESM3_ESM.zip › Raw WB data 0809/M GAPDH+LC3B+P62X3+PINK1X1/GAPDH.Tif小鼠x3用]

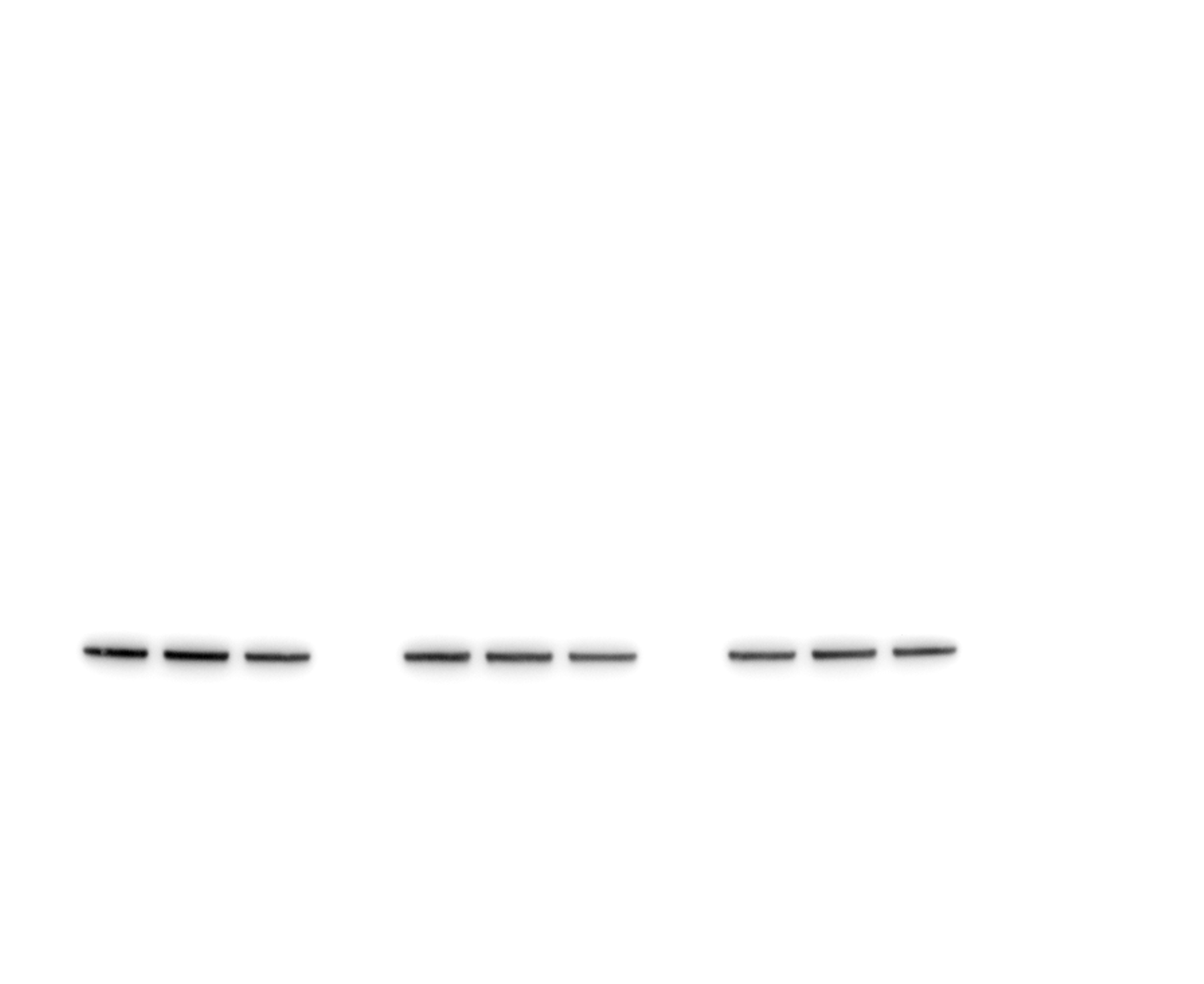

Supplement: Supplementary file 3 — Additional file 3. [file 13287_2026_4964_MOESM3_ESM.zip › Raw WB data 0809/M GAPDH+LC3B+P62X3+PINK1X1/GAPDH1228.Tif]

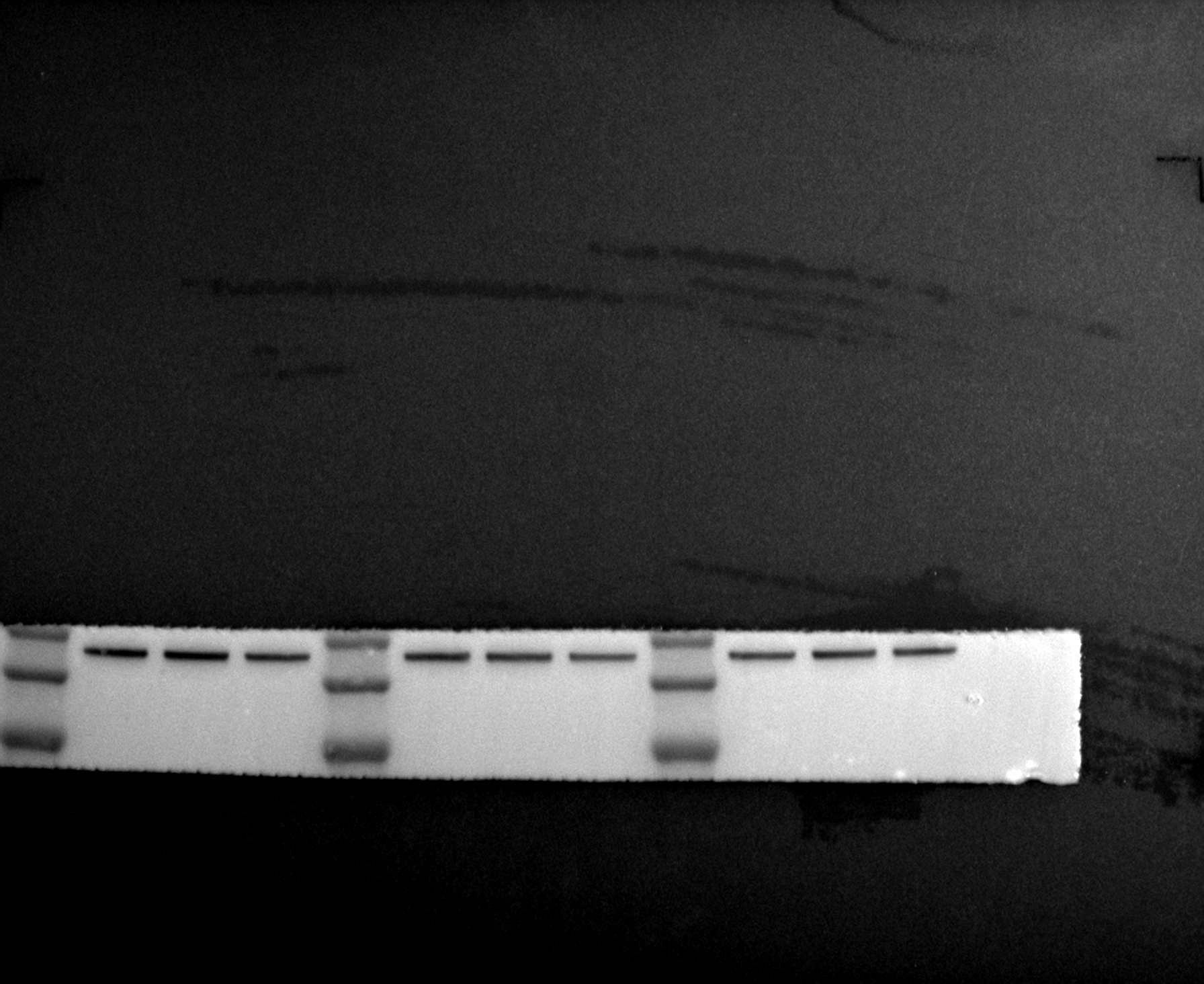

Supplement: Supplementary file 3 — Additional file 3. [file 13287_2026_4964_MOESM3_ESM.zip › Raw WB data 0809/M GAPDH+LC3B+P62X3+PINK1X1/GAPDH80S.Tif]

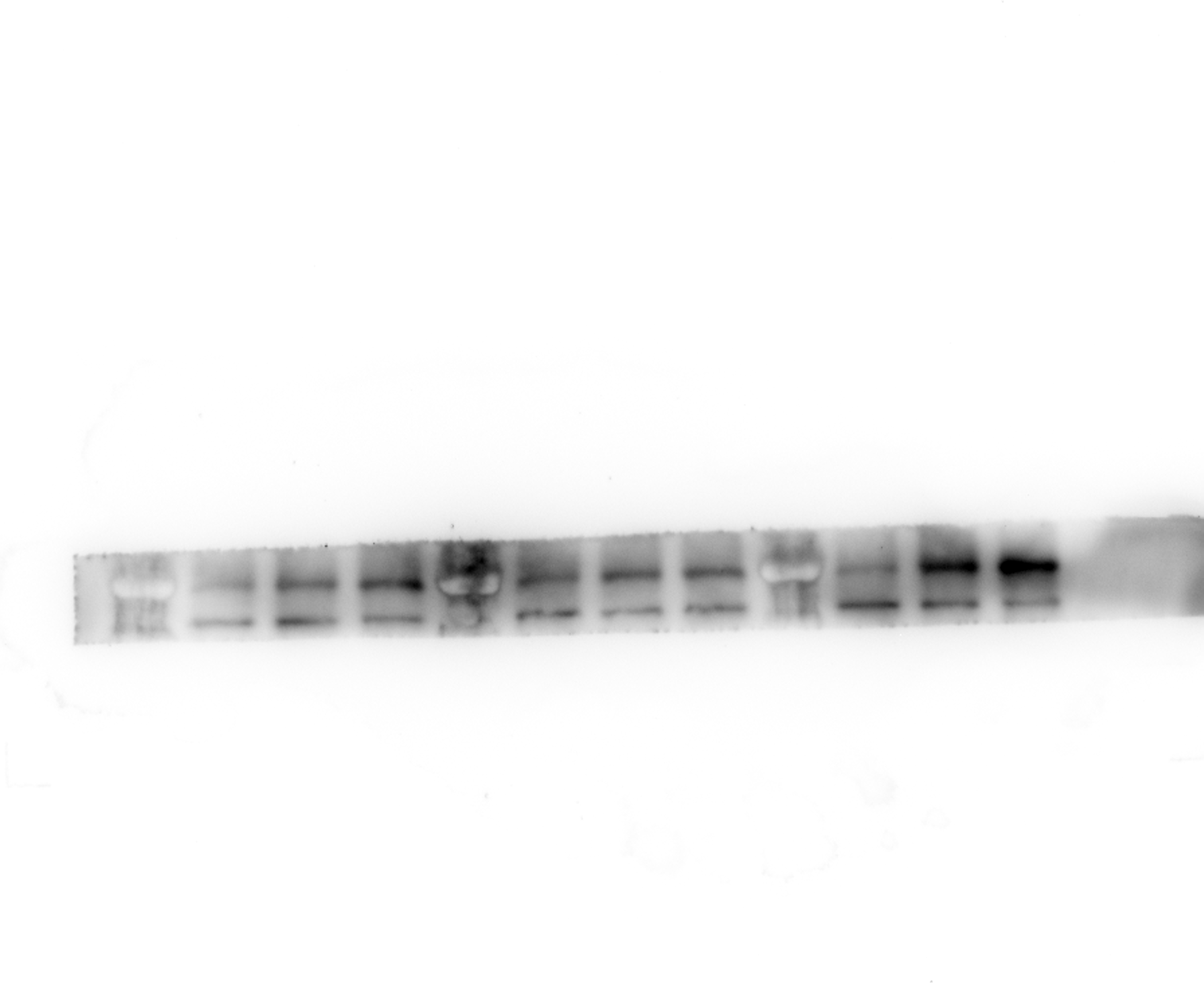

Supplement: Supplementary file 3 — Additional file 3. [file 13287_2026_4964_MOESM3_ESM.zip › Raw WB data 0809/M GAPDH+LC3B+P62X3+PINK1X1/LC3B.Tif小鼠x3]

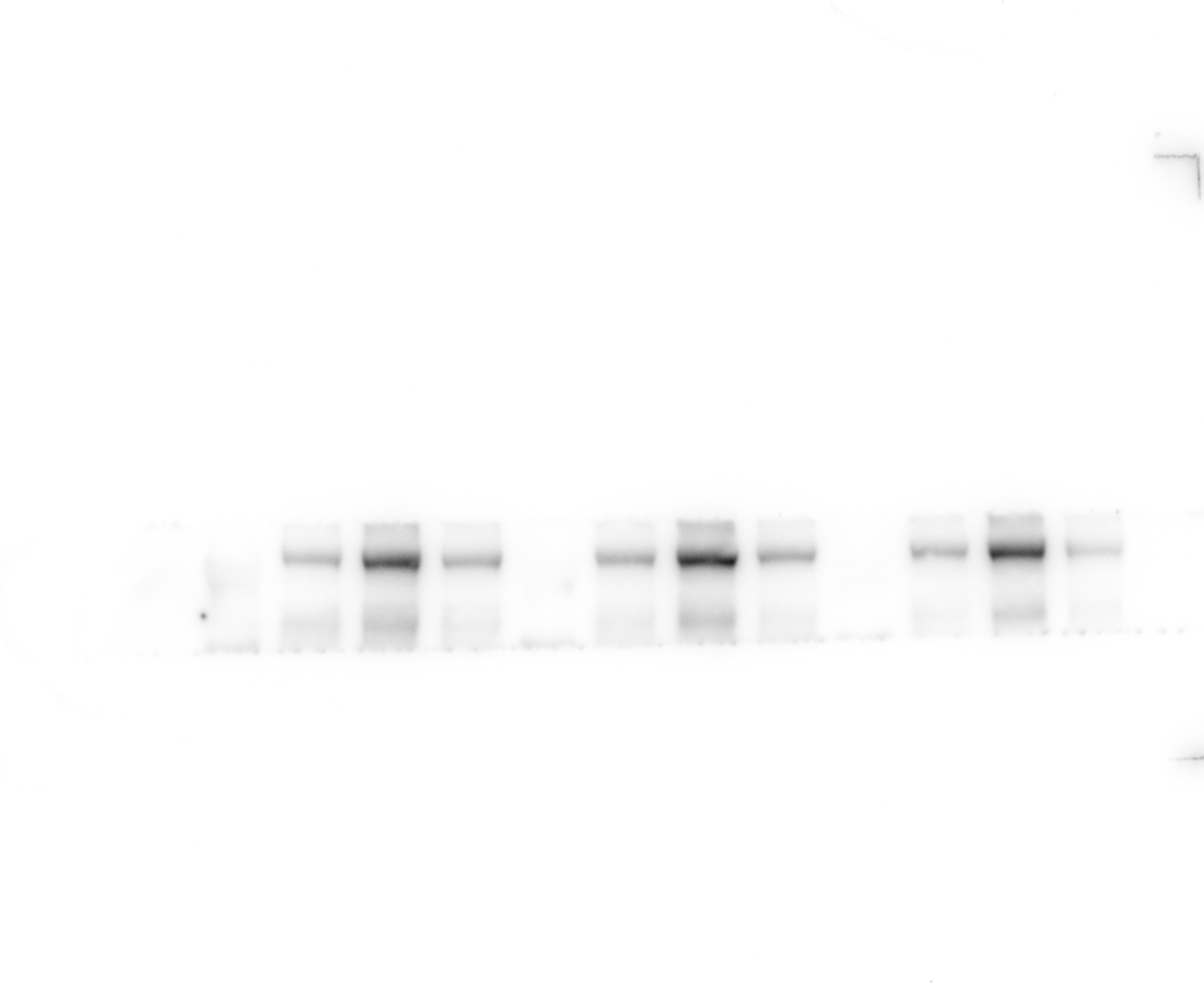

Supplement: Supplementary file 3 — Additional file 3. [file 13287_2026_4964_MOESM3_ESM.zip › Raw WB data 0809/M GAPDH+LC3B+P62X3+PINK1X1/P62 30S.Ti小鼠x3用]

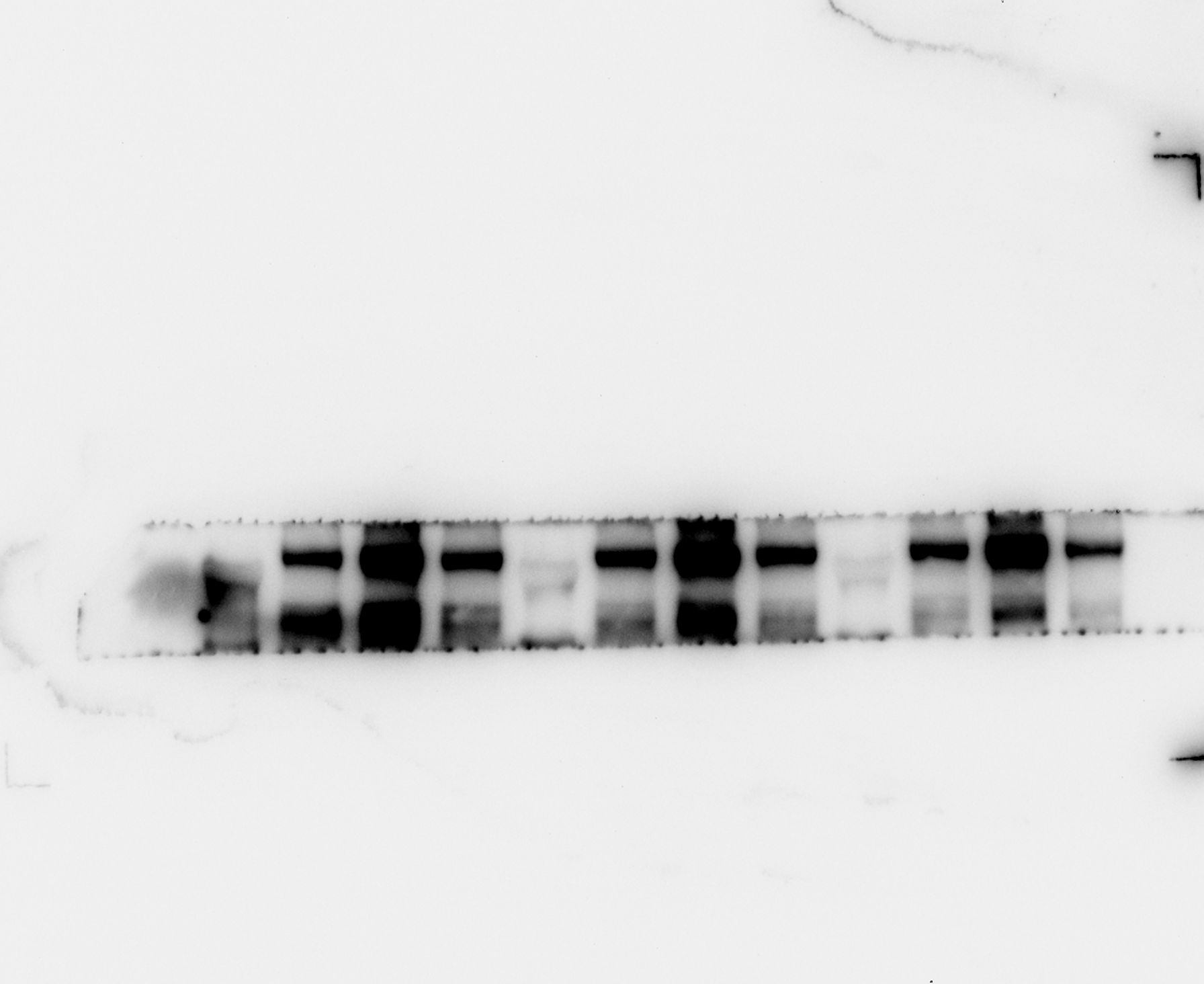

Supplement: Supplementary file 3 — Additional file 3. [file 13287_2026_4964_MOESM3_ESM.zip › Raw WB data 0809/M GAPDH+LC3B+P62X3+PINK1X1/P62 60s.小鼠用x3]

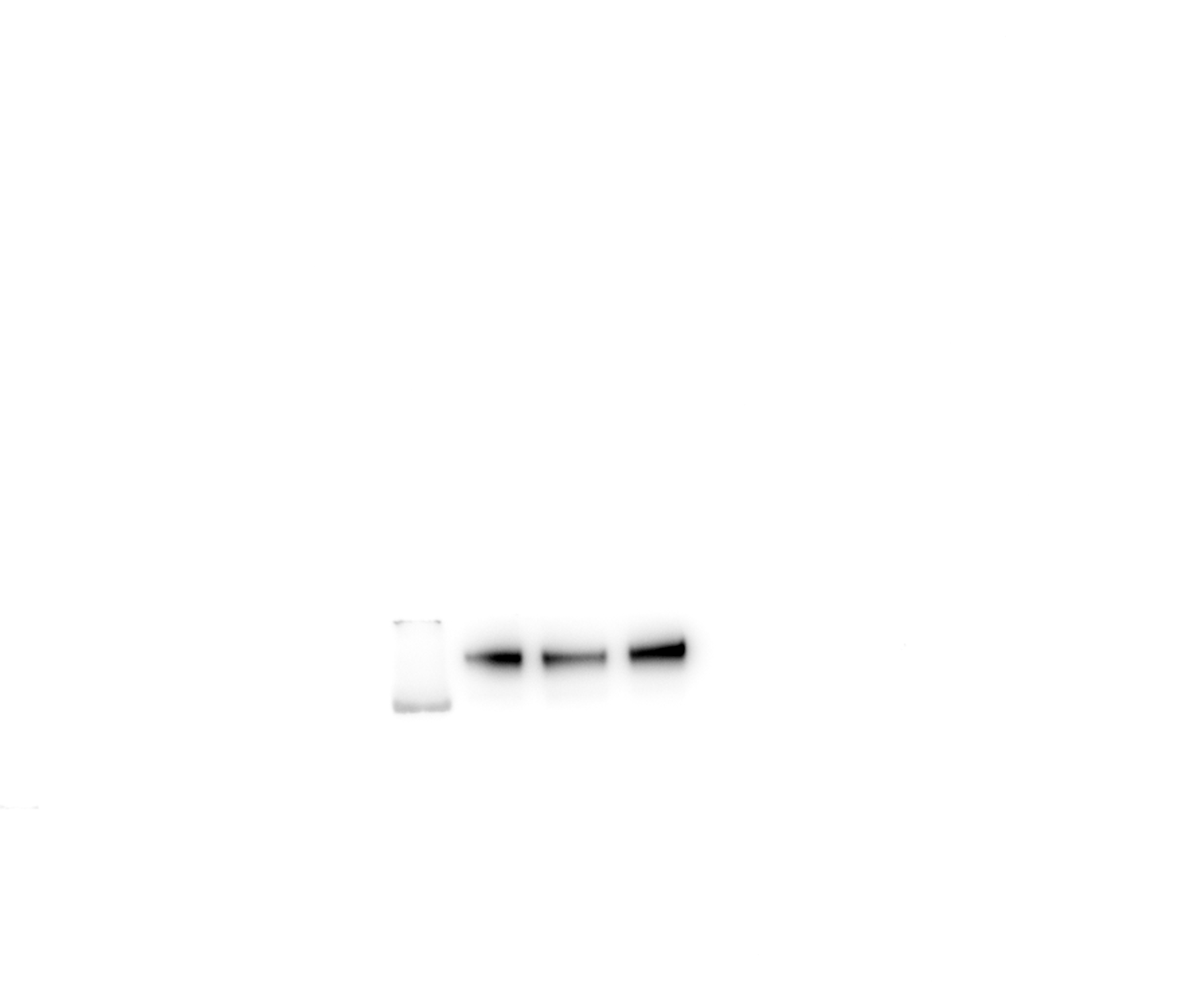

Supplement: Supplementary file 3 — Additional file 3. [file 13287_2026_4964_MOESM3_ESM.zip › Raw WB data 0809/M GAPDH+LC3B+P62X3+PINK1X1/PINK1-1.小鼠x1]

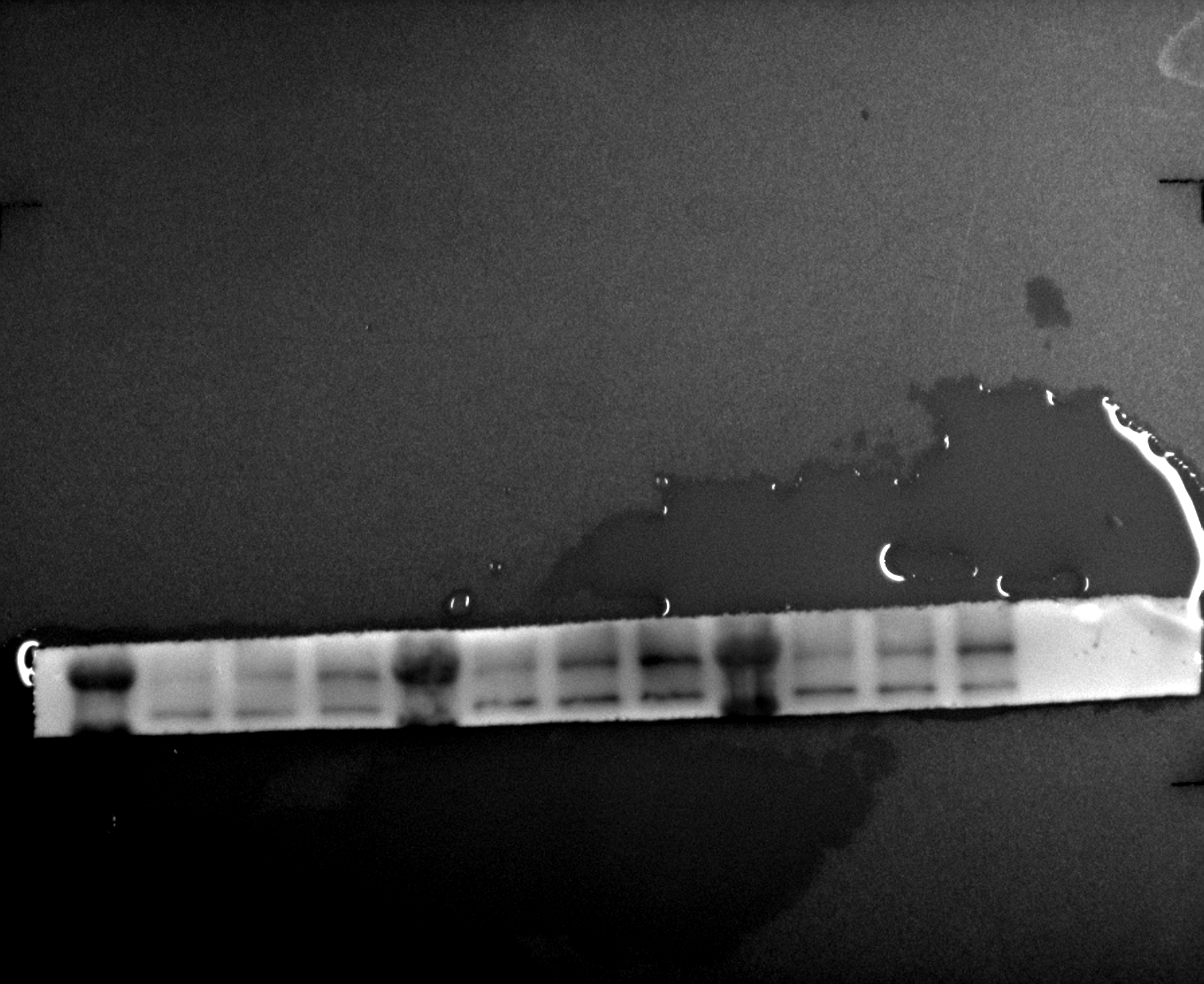

Supplement: Supplementary file 3 — Additional file 3. [file 13287_2026_4964_MOESM3_ESM.zip › Raw WB data 0809/M GAPDH+LC3B+P62X3+PINK1X1/parkin]

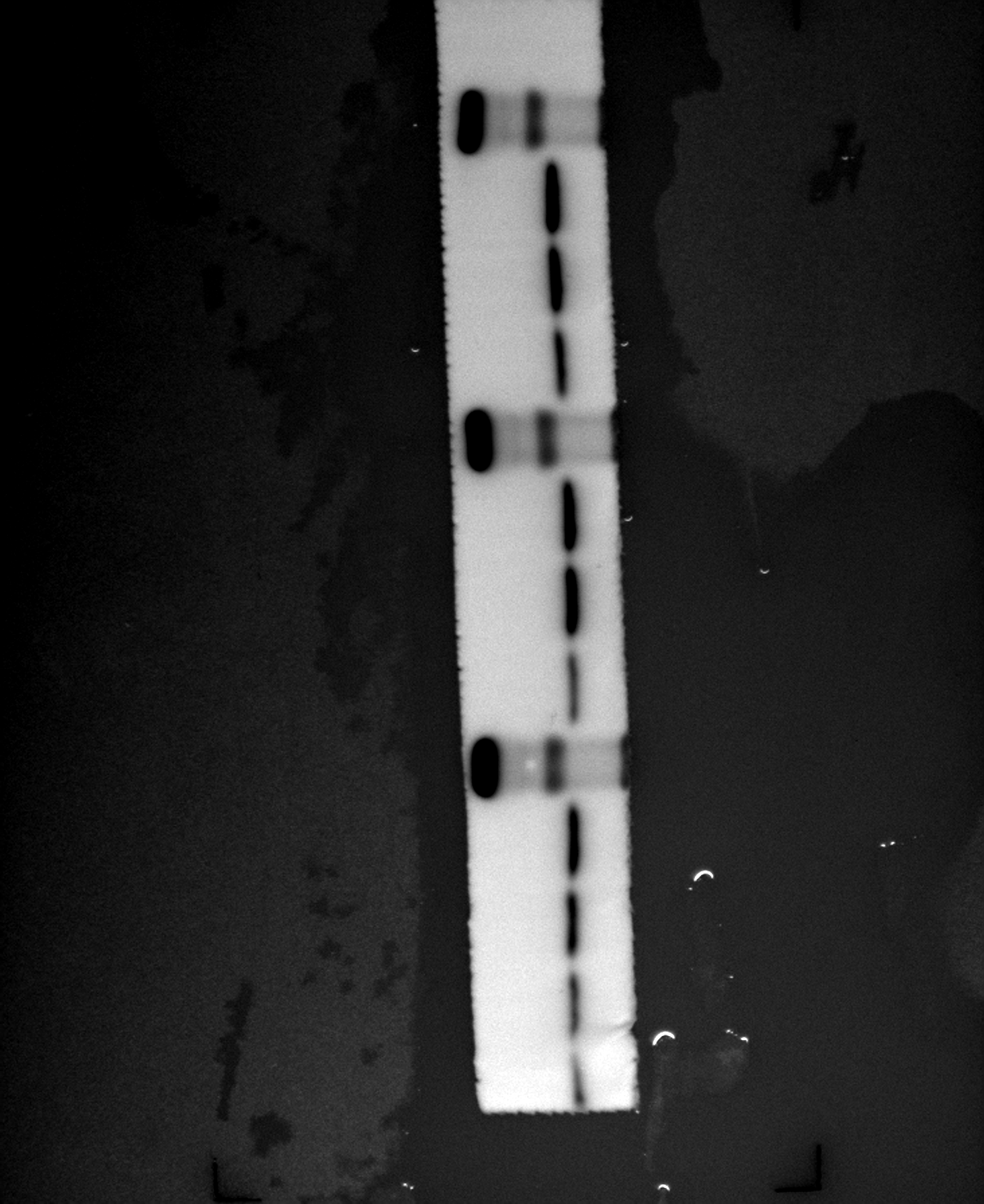

Supplement: Supplementary file 3 — Additional file 3. [file 13287_2026_4964_MOESM3_ESM.zip › Raw WB data 0809/M GAPDH+LC3B+P62X3+PINK1X1/tom20-2.Tif]

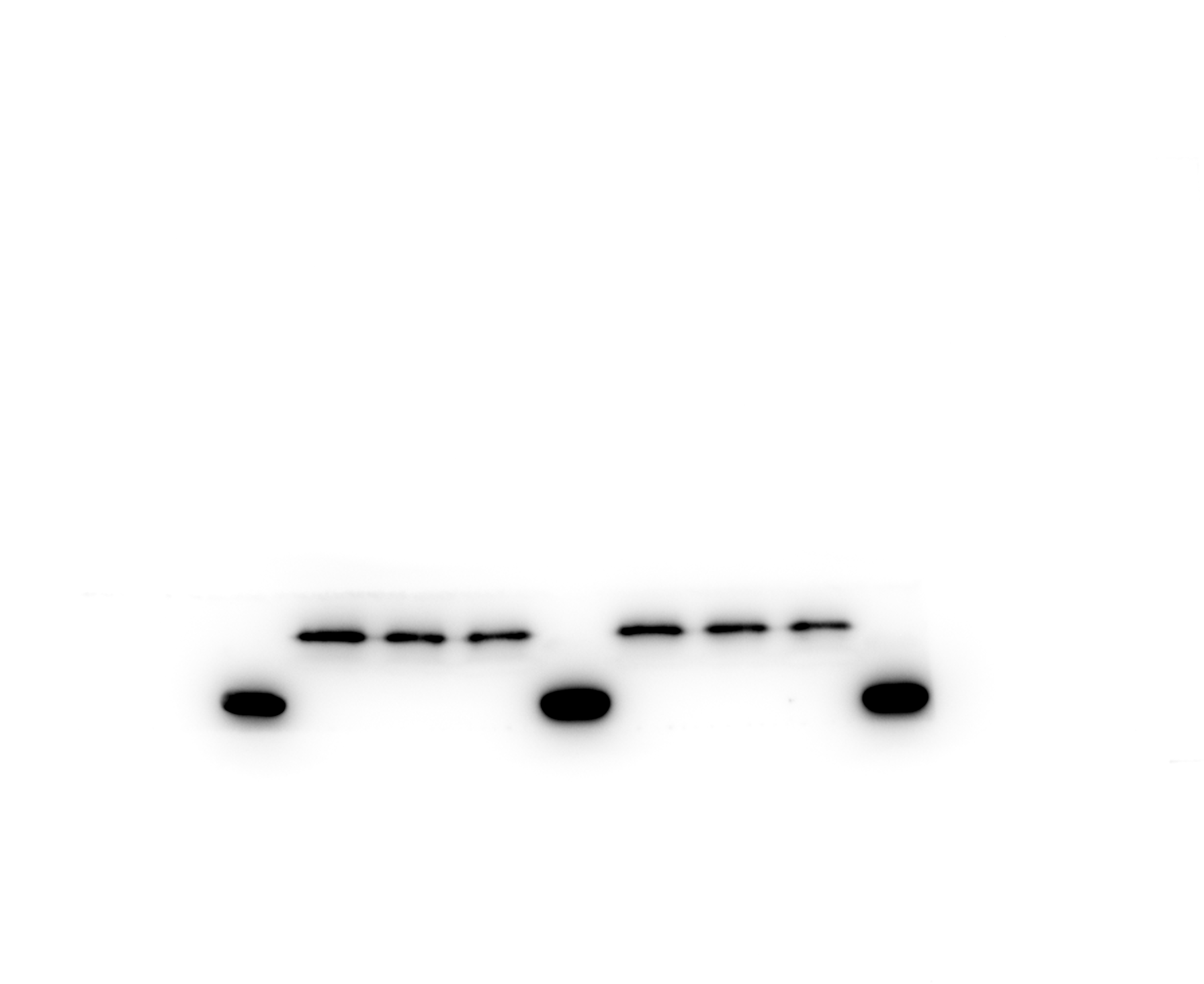

Supplement: Supplementary file 3 — Additional file 3. [file 13287_2026_4964_MOESM3_ESM.zip › Raw WB data 0809/M GAPDH+LC3B+P62X3+PINK1X1/tomo20.Tif]

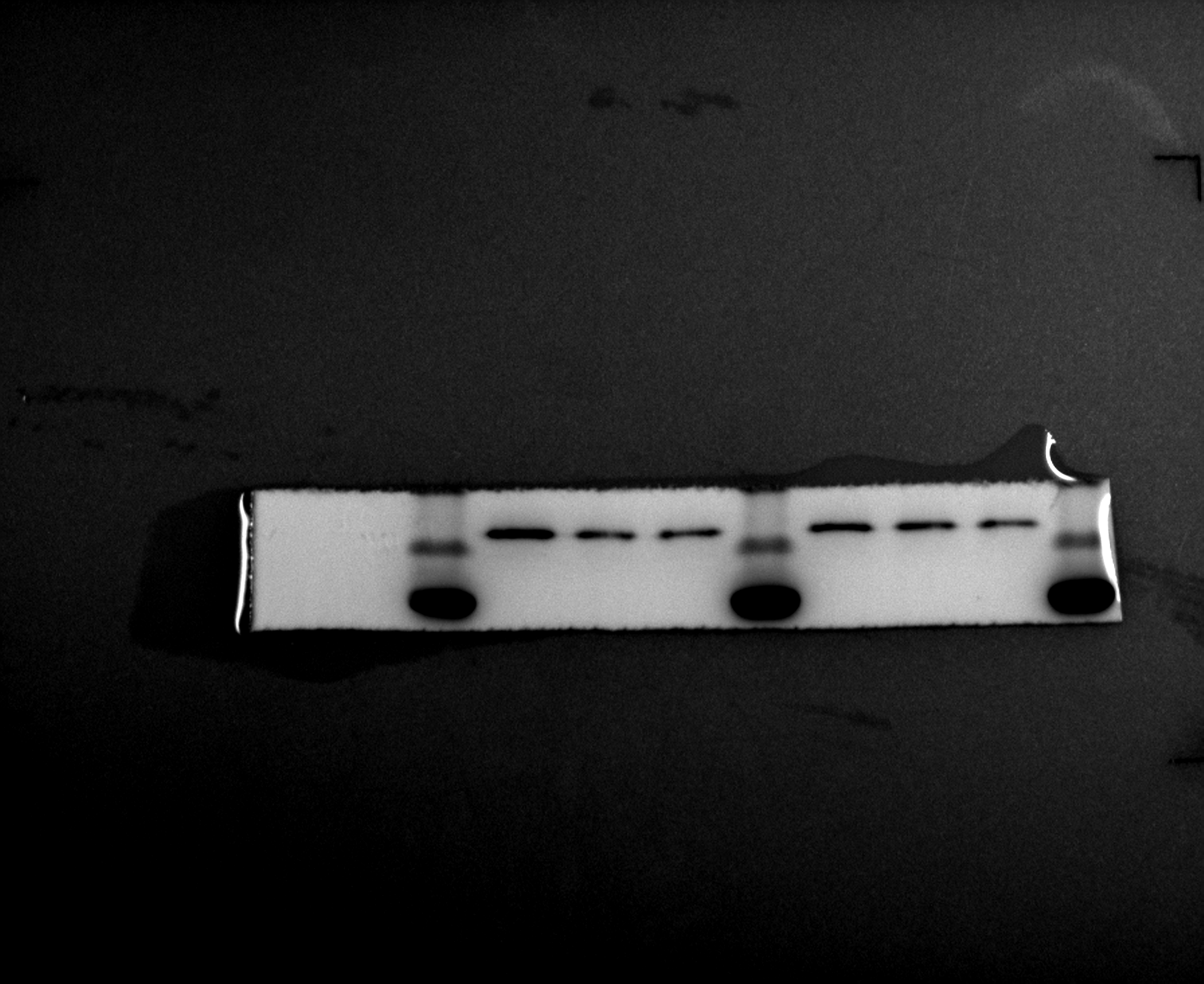

Supplement: Supplementary file 3 — Additional file 3. [file 13287_2026_4964_MOESM3_ESM.zip › Raw WB data 0809/M GAPDH+LC3B+P62X3+PINK1X1/tomo20QUANMO.Tif]

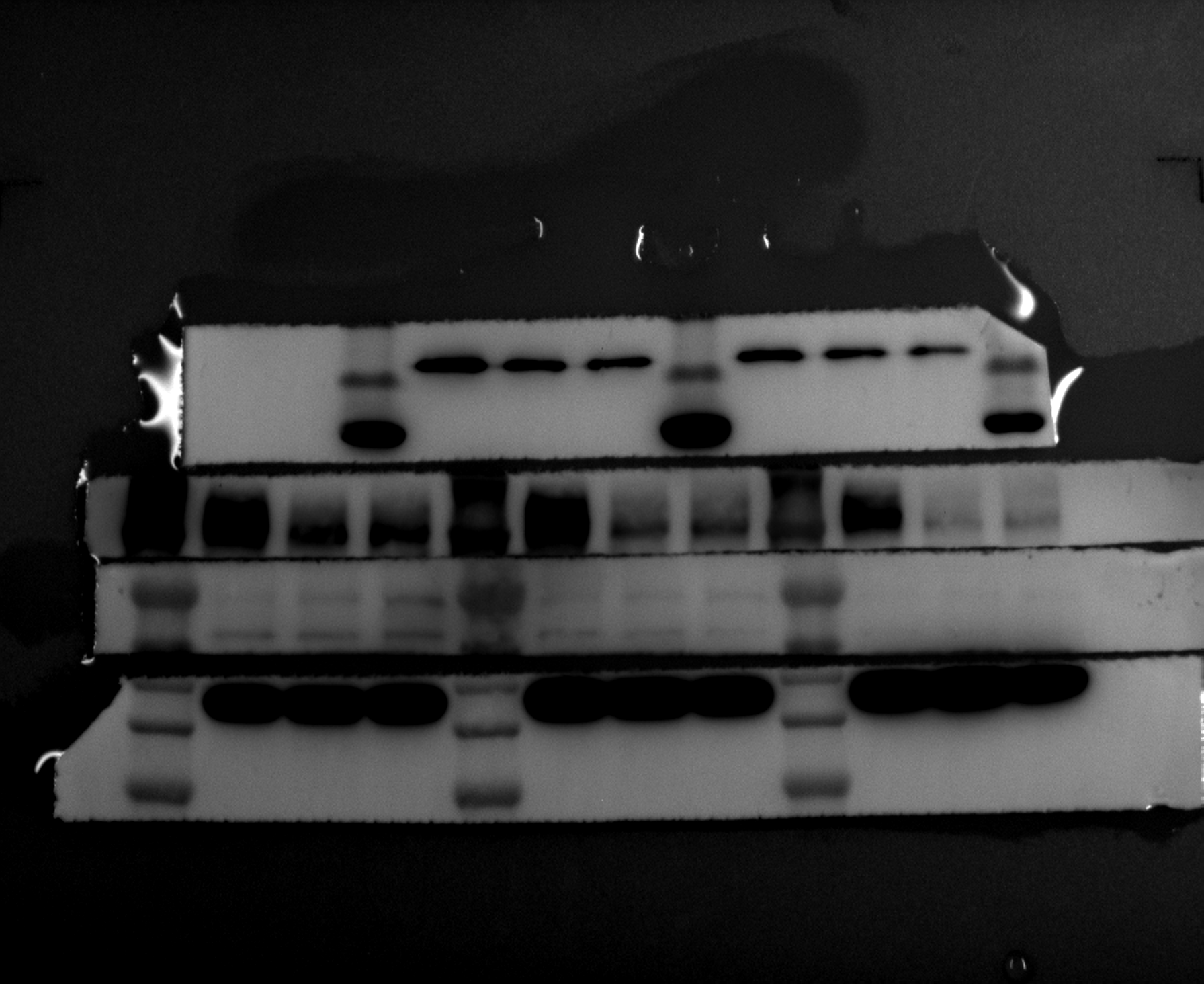

Supplement: Supplementary file 3 — Additional file 3. [file 13287_2026_4964_MOESM3_ESM.zip › Raw WB data 0809/M GAPDH+LC3B+P62X3+PINK1X1/全膜合成.Tif]

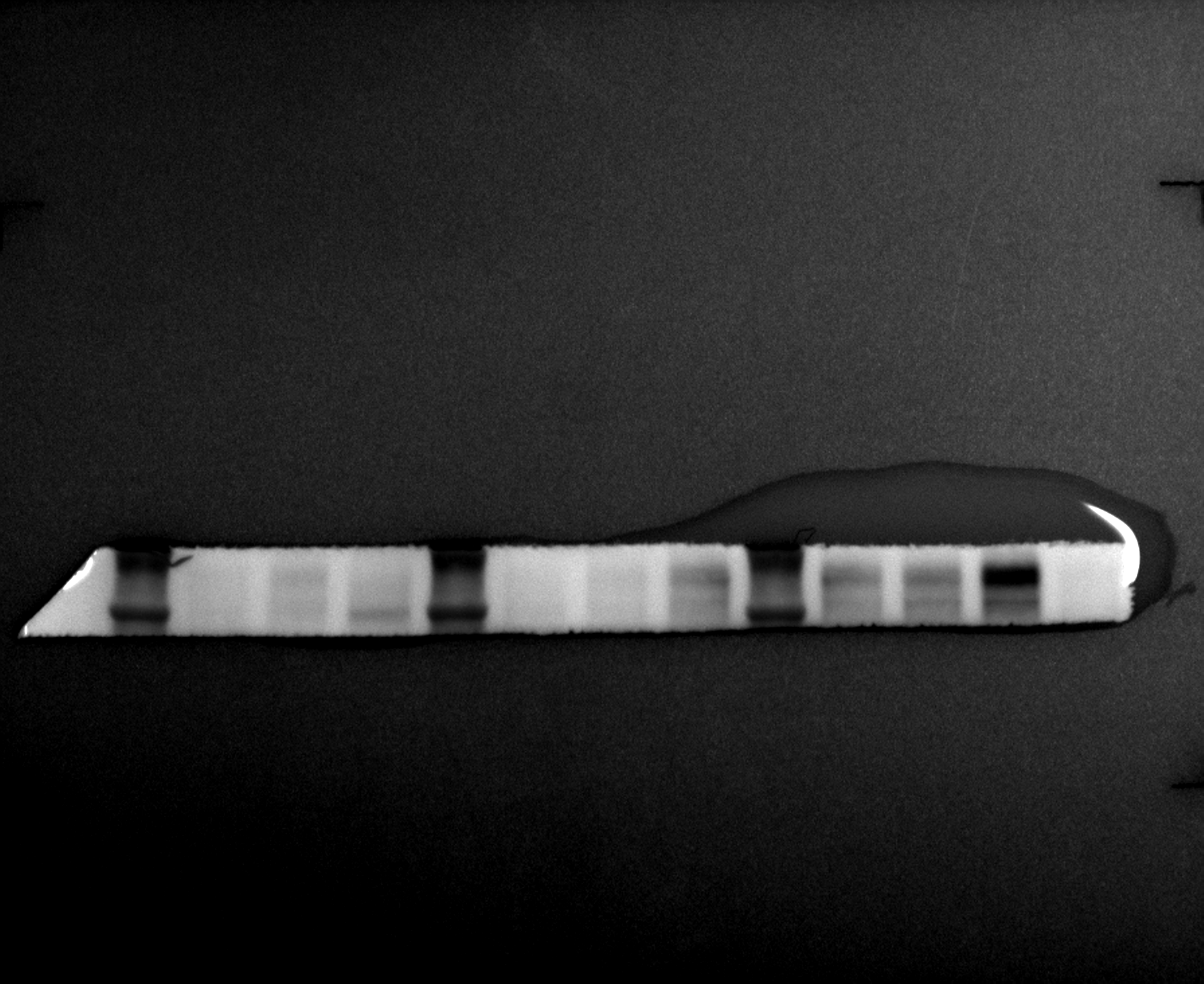

Supplement: Supplementary file 3 — Additional file 3. [file 13287_2026_4964_MOESM3_ESM.zip › Raw WB data 0809/M GAPDH+LC3B+P62X3+PINK1X1/小鼠parkinX3]

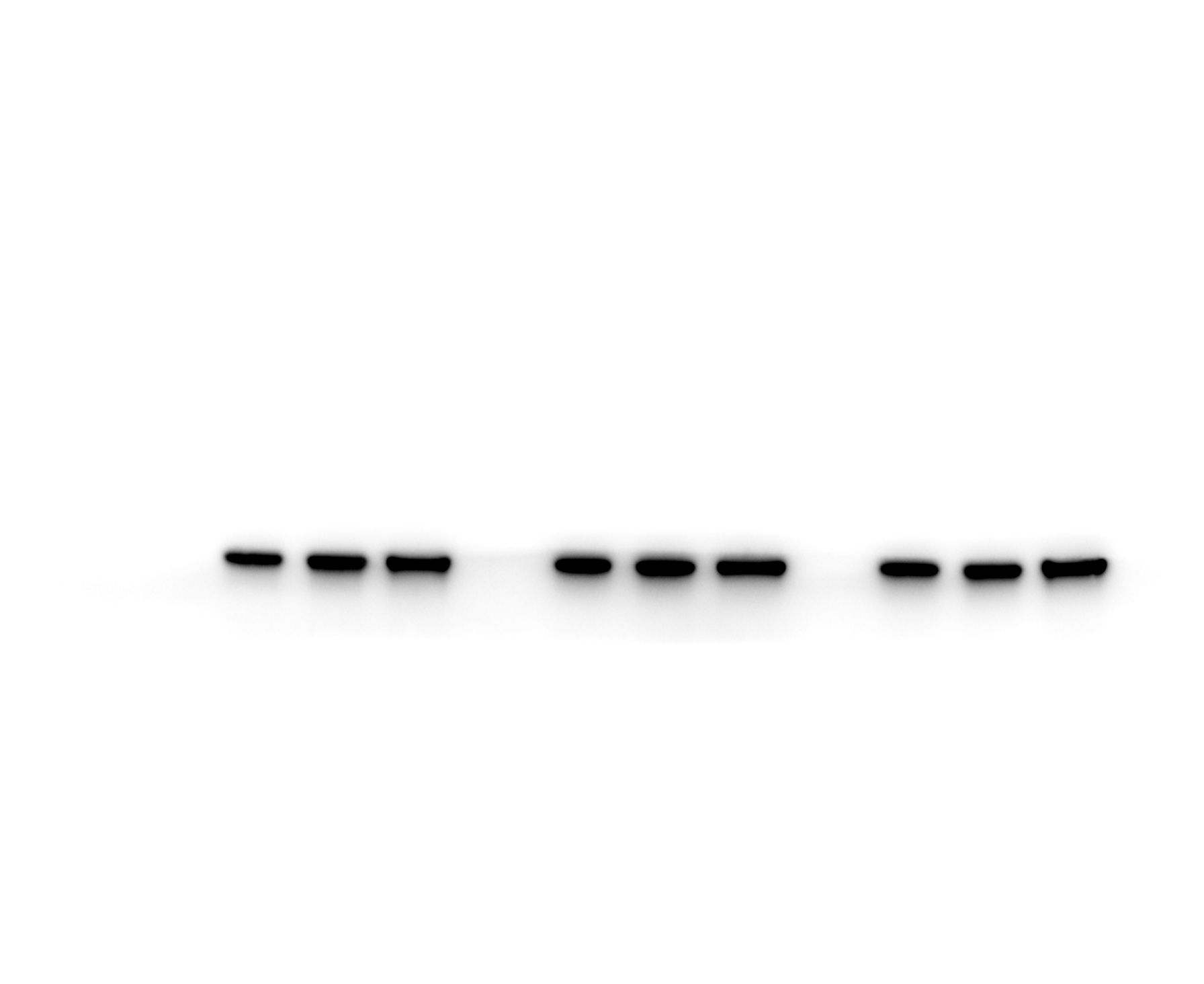

Supplement: Supplementary file 3 — Additional file 3. [file 13287_2026_4964_MOESM3_ESM.zip › Raw WB data 0809/M WBPINK1+Parkin/GAPDH %.Tif]

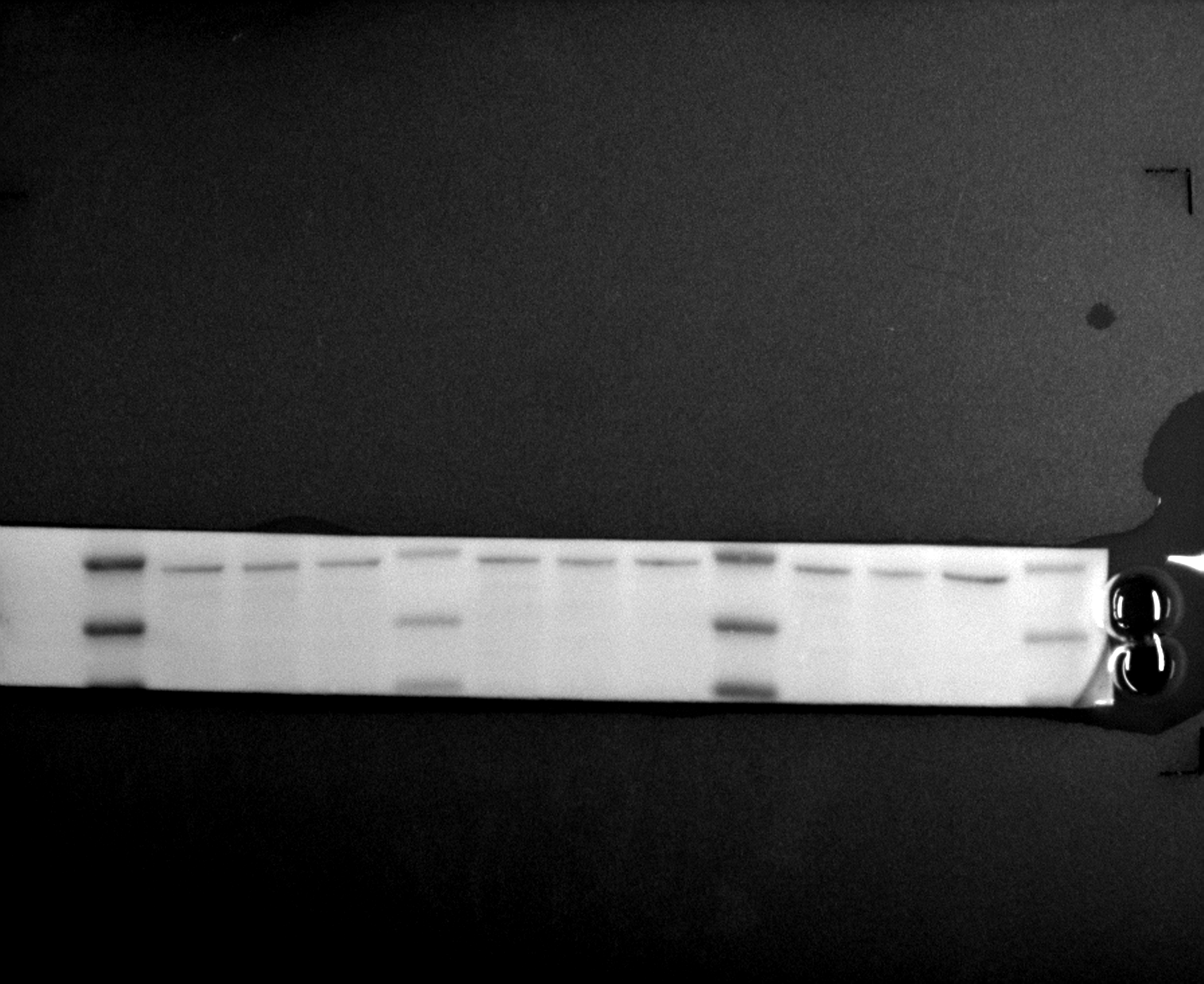

Supplement: Supplementary file 3 — Additional file 3. [file 13287_2026_4964_MOESM3_ESM.zip › Raw WB data 0809/M WBPINK1+Parkin/GAPDH全膜2.Tif]

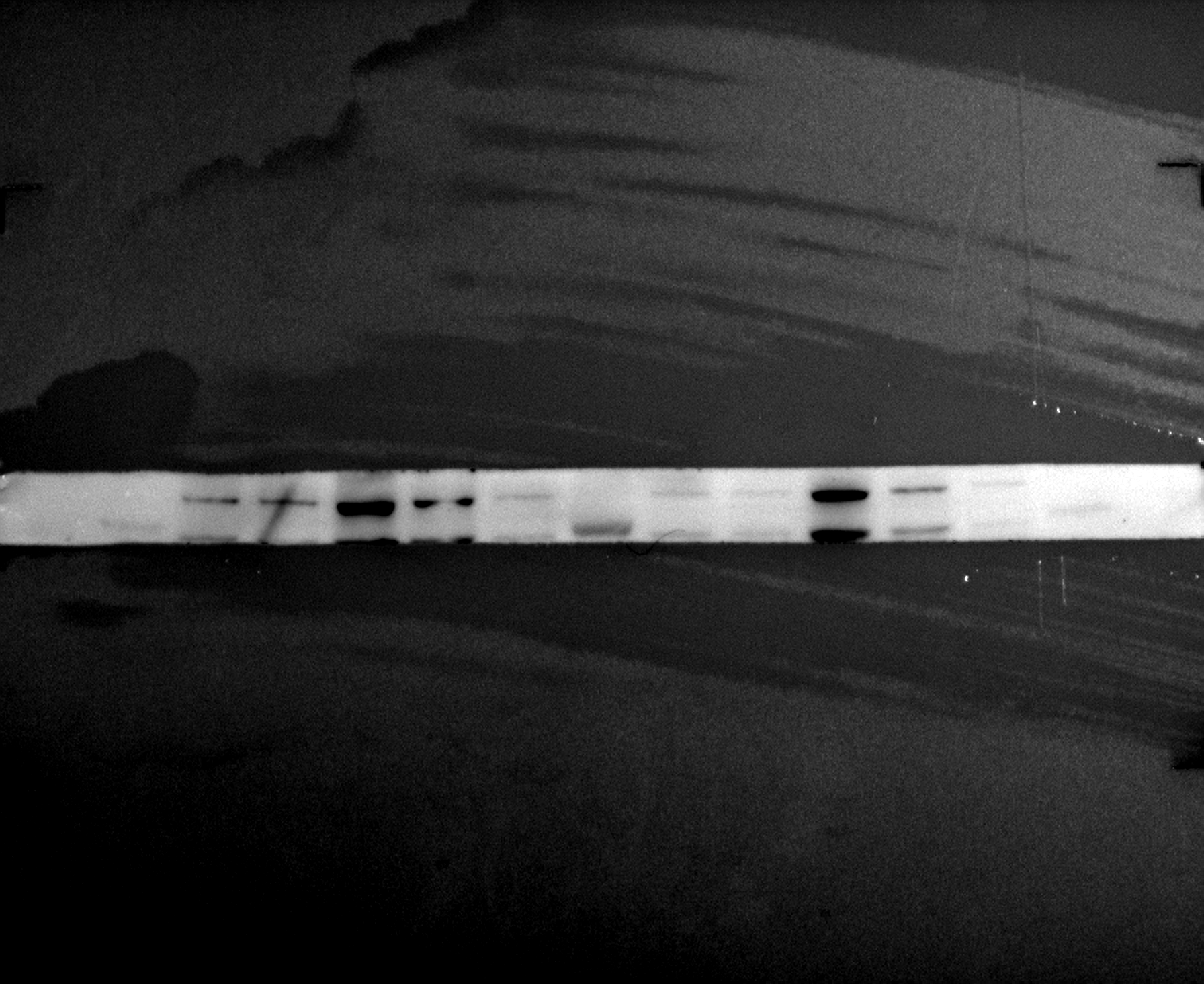

Supplement: Supplementary file 3 — Additional file 3. [file 13287_2026_4964_MOESM3_ESM.zip › Raw WB data 0809/M WBPINK1+Parkin/PINK1 rescuex20418.Tif]

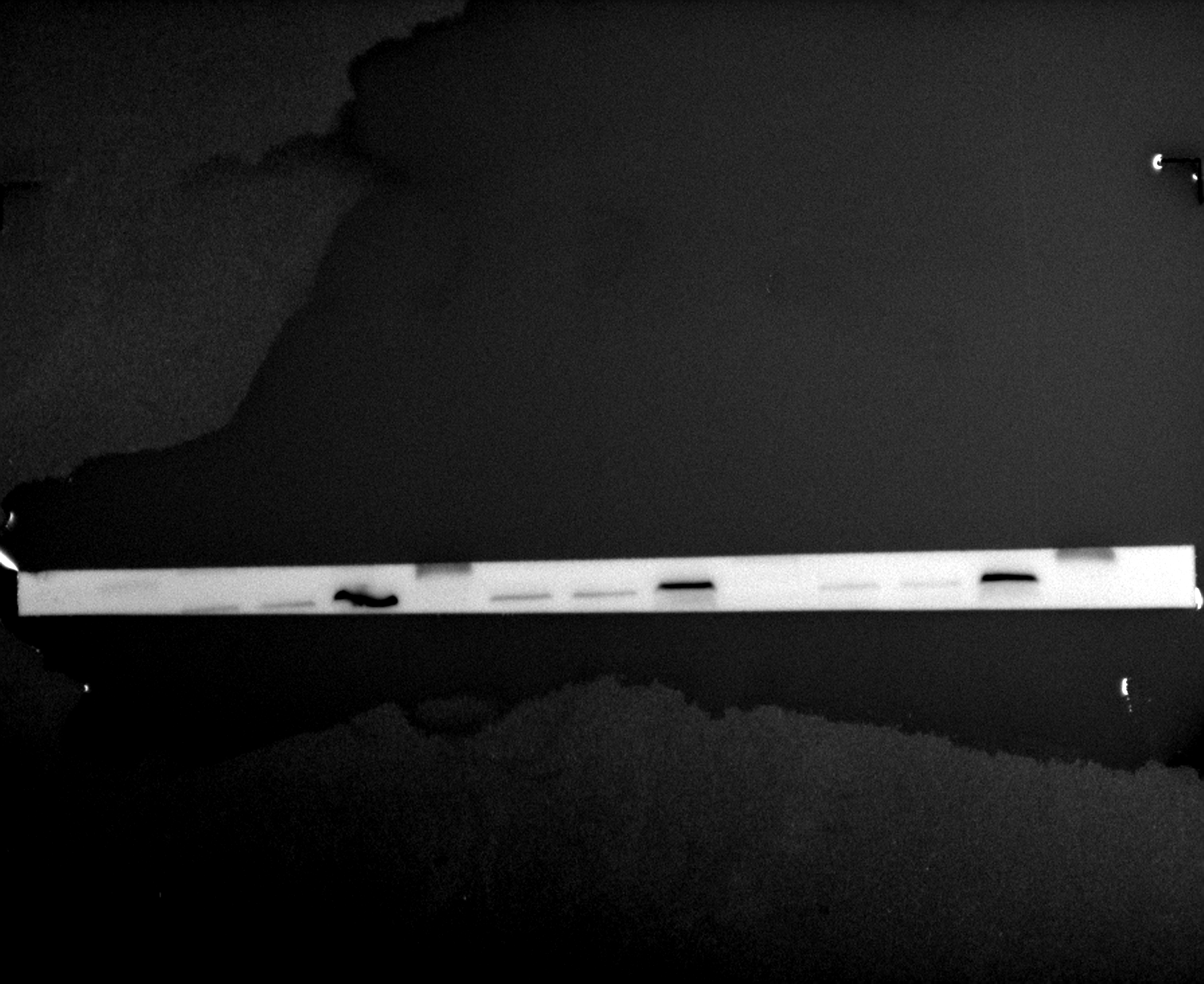

Supplement: Supplementary file 3 — Additional file 3. [file 13287_2026_4964_MOESM3_ESM.zip › Raw WB data 0809/M WBPINK1+Parkin/PINK1全膜.Tif]

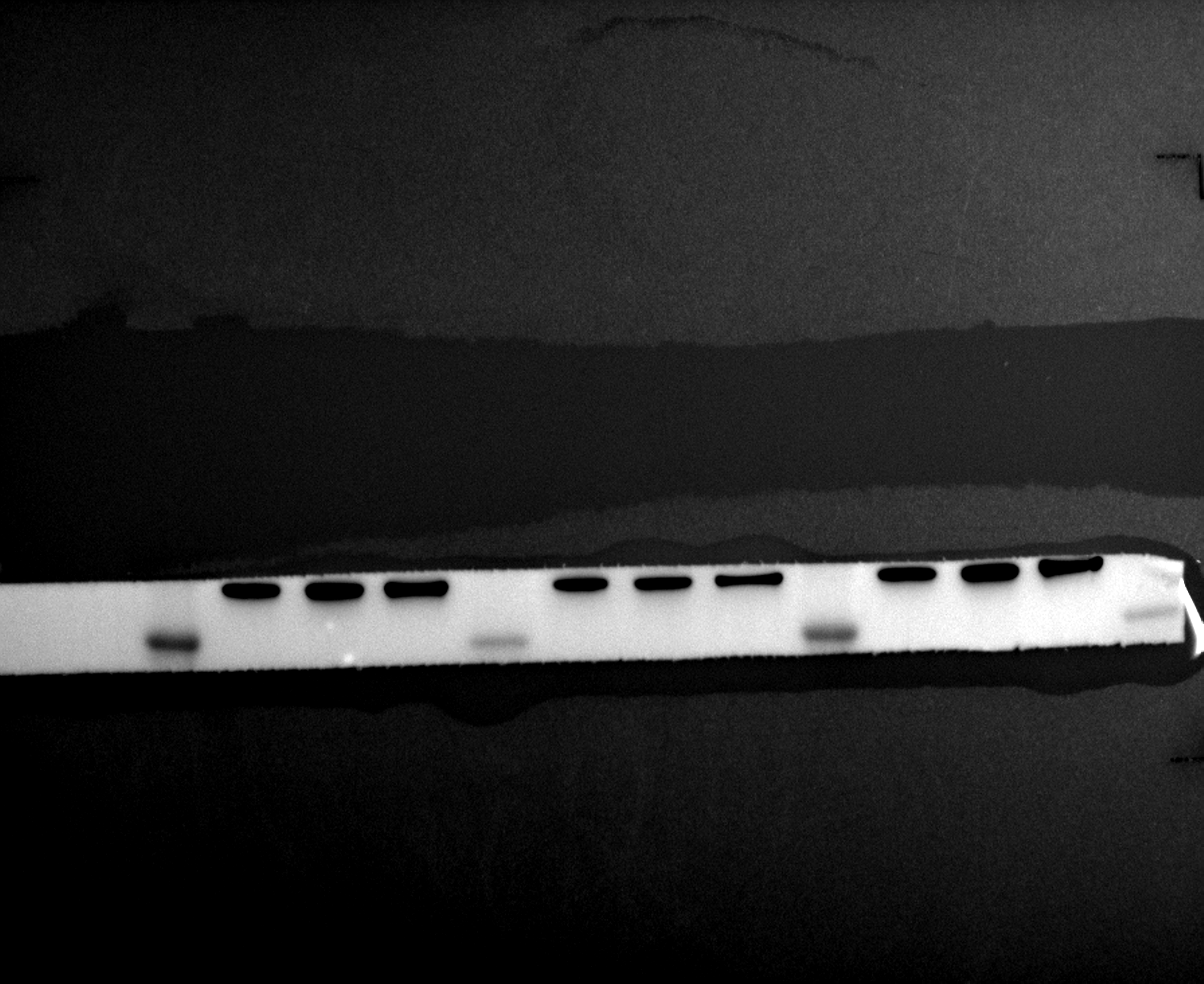

Supplement: Supplementary file 3 — Additional file 3. [file 13287_2026_4964_MOESM3_ESM.zip › Raw WB data 0809/M WBPINK1+Parkin/gapdh全膜.Tif2.Tif]

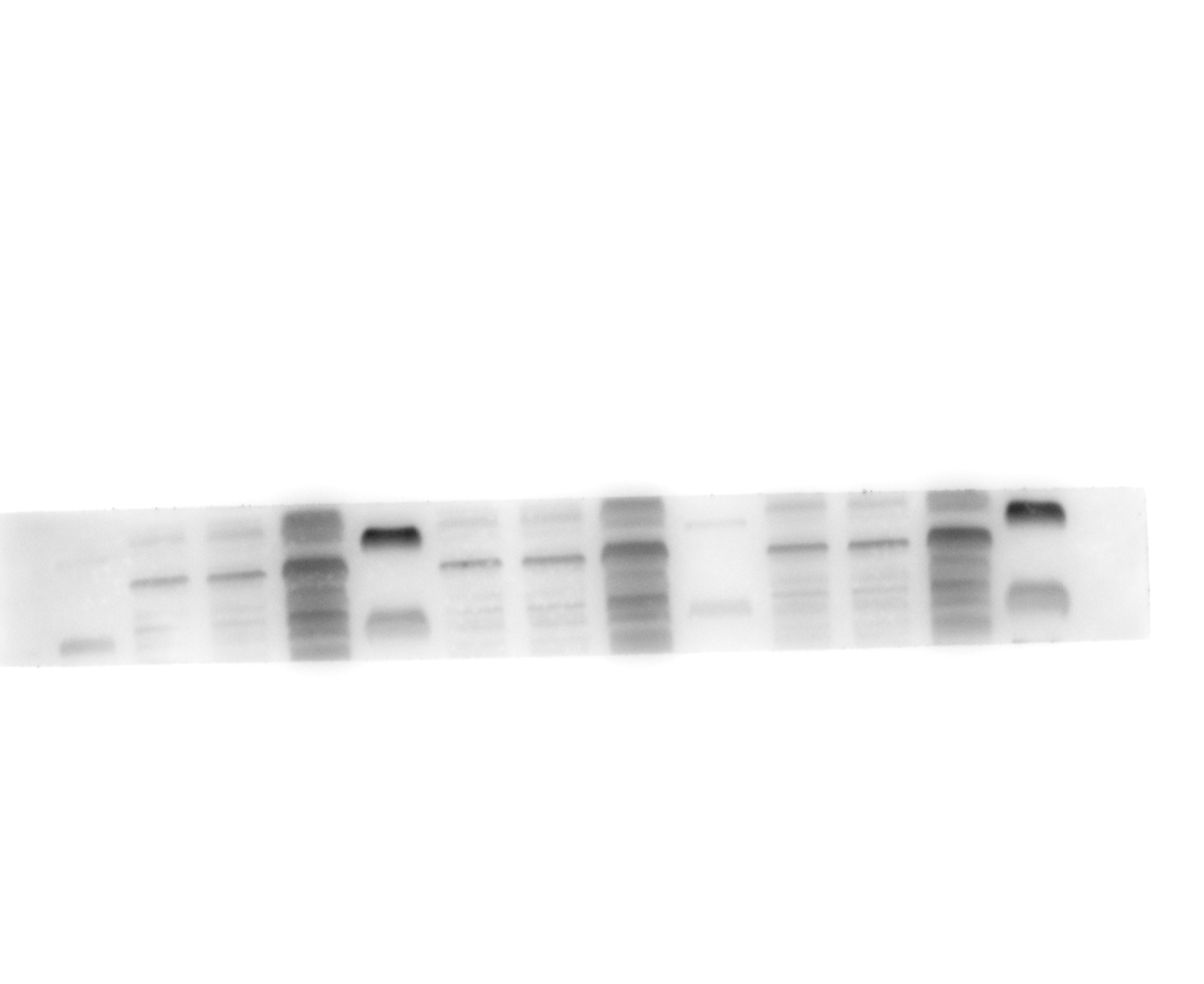

Supplement: Supplementary file 3 — Additional file 3. [file 13287_2026_4964_MOESM3_ESM.zip › Raw WB data 0809/M WBPINK1+Parkin/parkin-400ms.Tif]

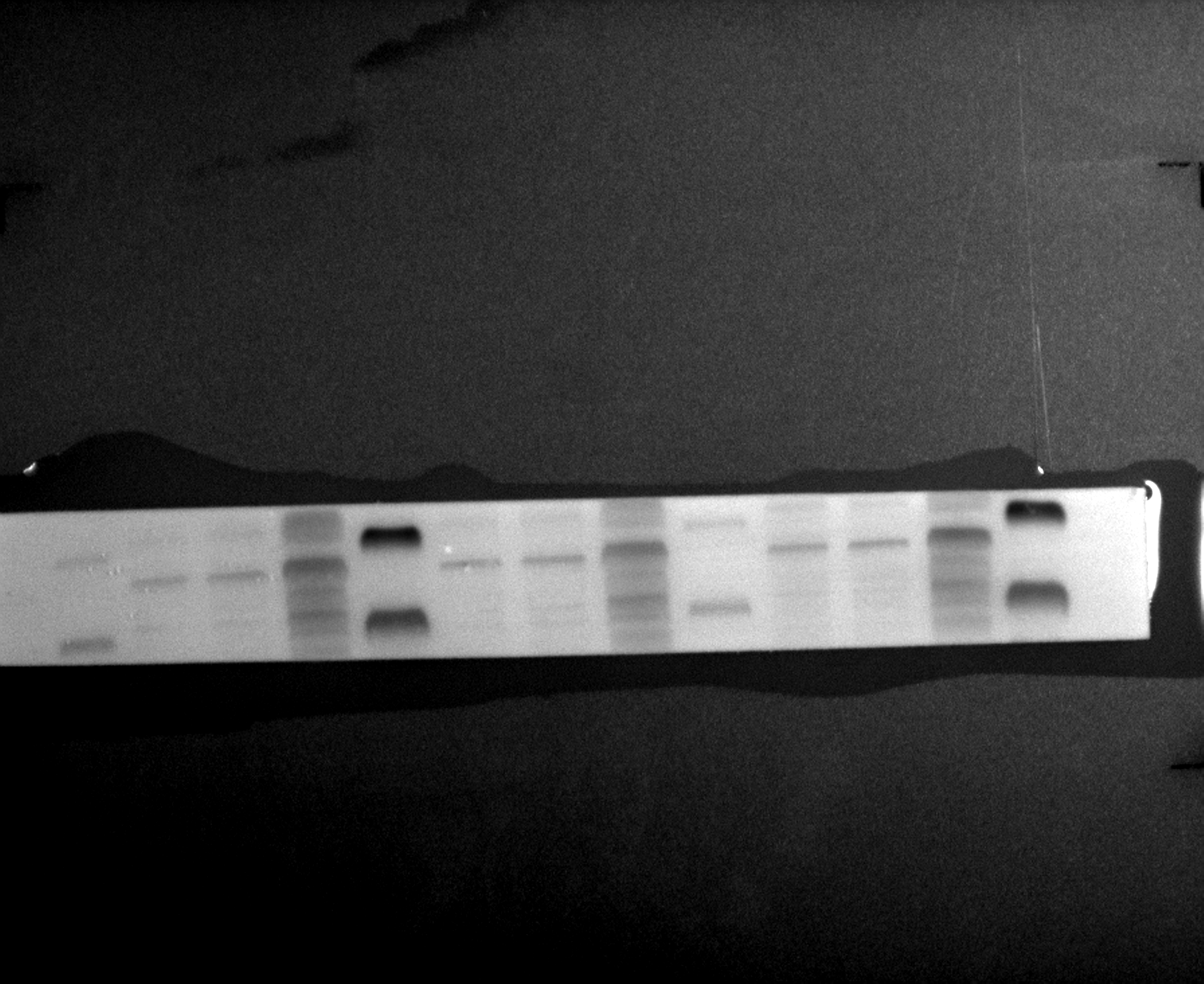

Supplement: Supplementary file 3 — Additional file 3. [file 13287_2026_4964_MOESM3_ESM.zip › Raw WB data 0809/M WBPINK1+Parkin/parkin全膜.Tif]

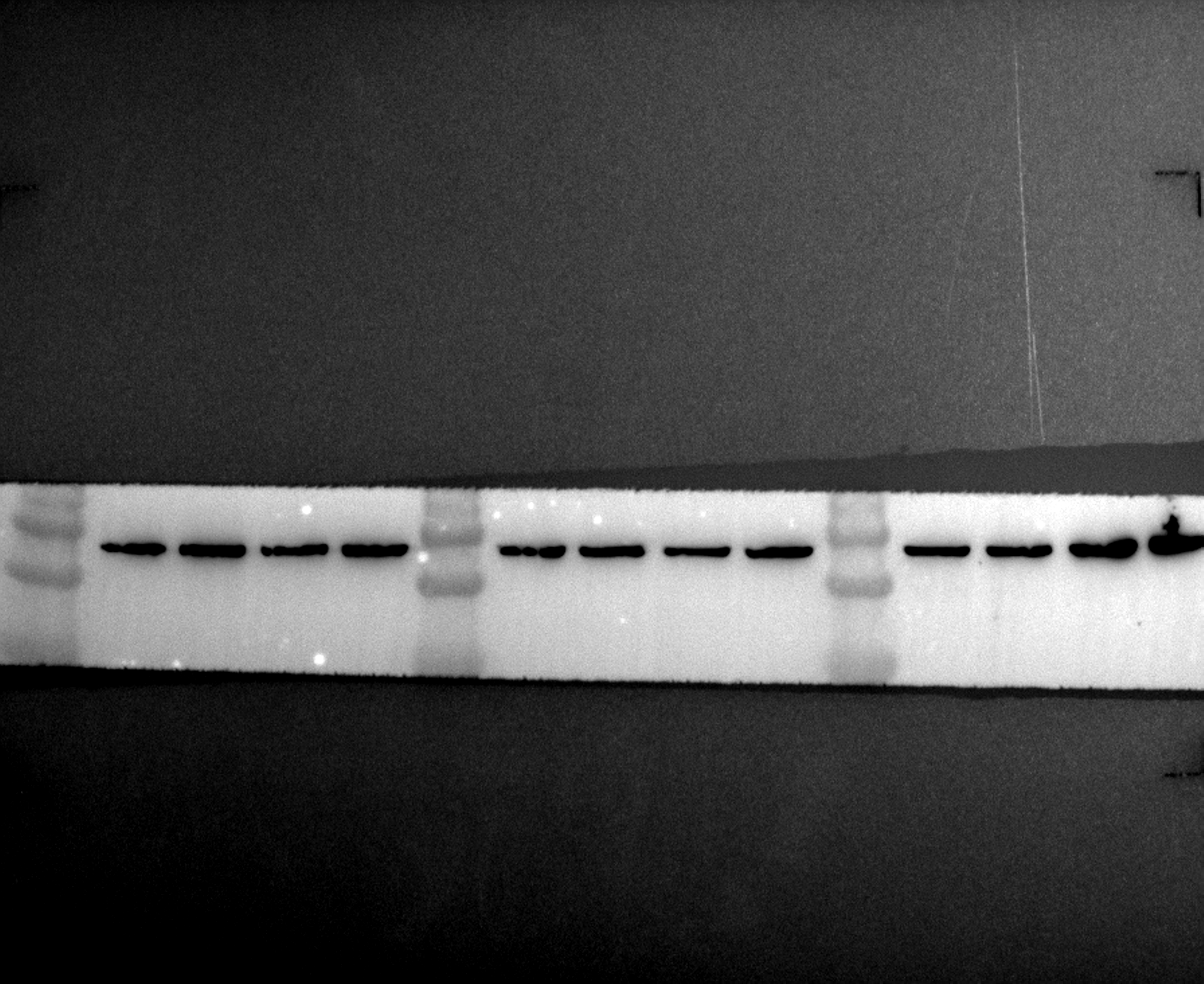

Supplement: Supplementary file 3 — Additional file 3. [file 13287_2026_4964_MOESM3_ESM.zip › Raw WB data 0809/PINK1P62LC3Bbeclin-1PARKINGAPDHsiRNA20250520/GAPDH-1S-R.Tif]

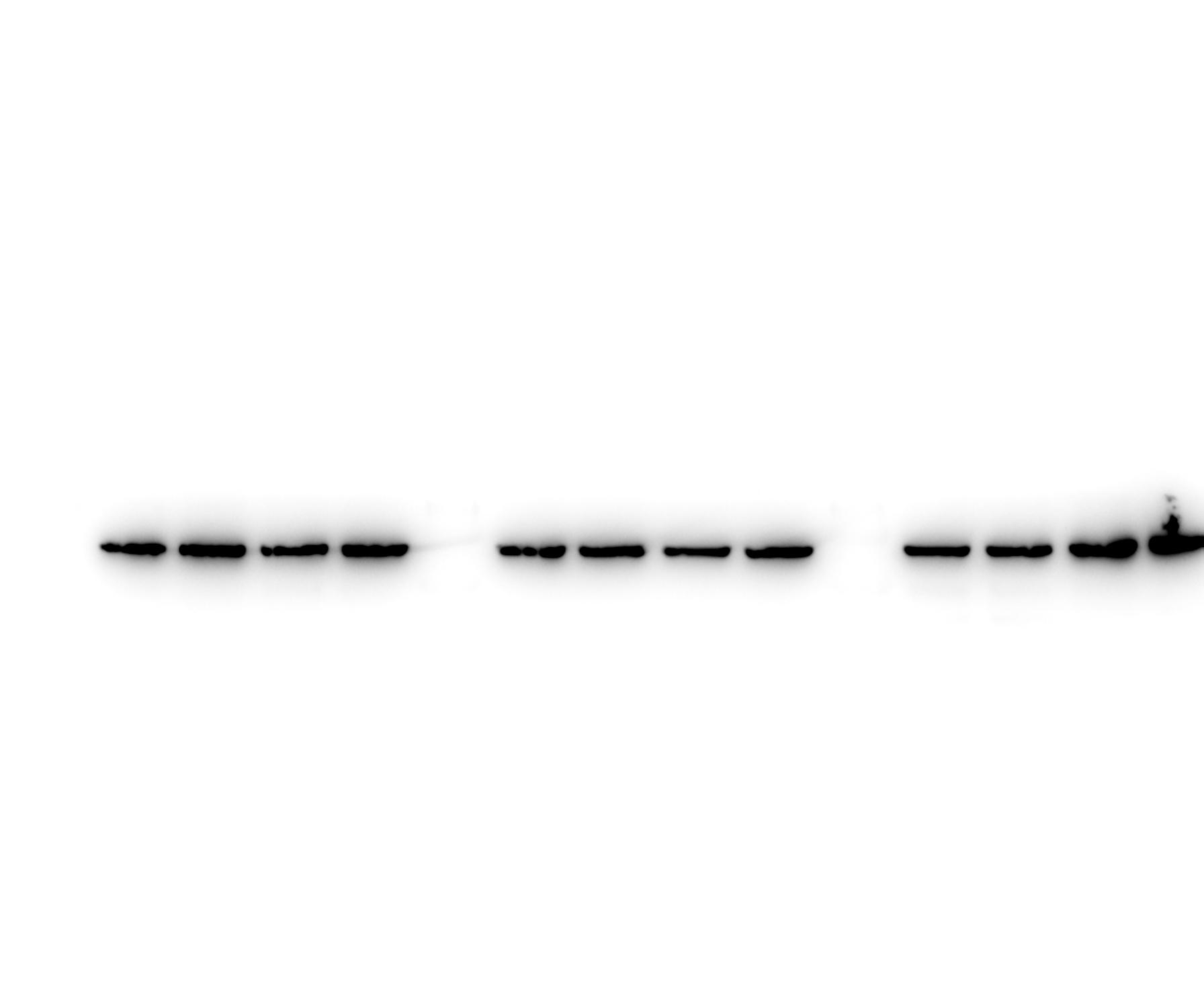

Supplement: Supplementary file 3 — Additional file 3. [file 13287_2026_4964_MOESM3_ESM.zip › Raw WB data 0809/PINK1P62LC3Bbeclin-1PARKINGAPDHsiRNA20250520/GAPDH-1S.Tif]

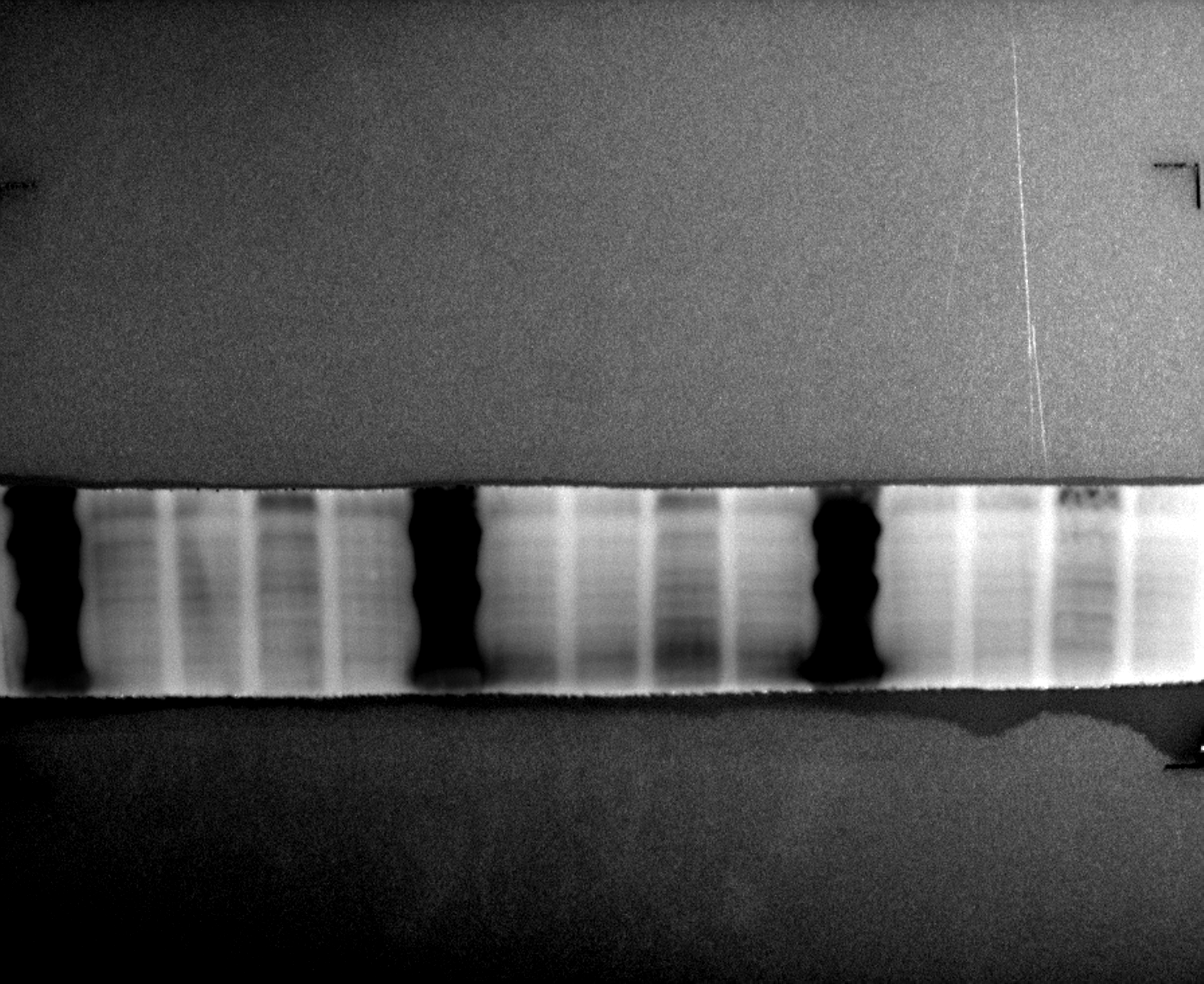

Supplement: Supplementary file 3 — Additional file 3. [file 13287_2026_4964_MOESM3_ESM.zip › Raw WB data 0809/PINK1P62LC3Bbeclin-1PARKINGAPDHsiRNA20250520/PARKIN-2.5S-R.Tif]

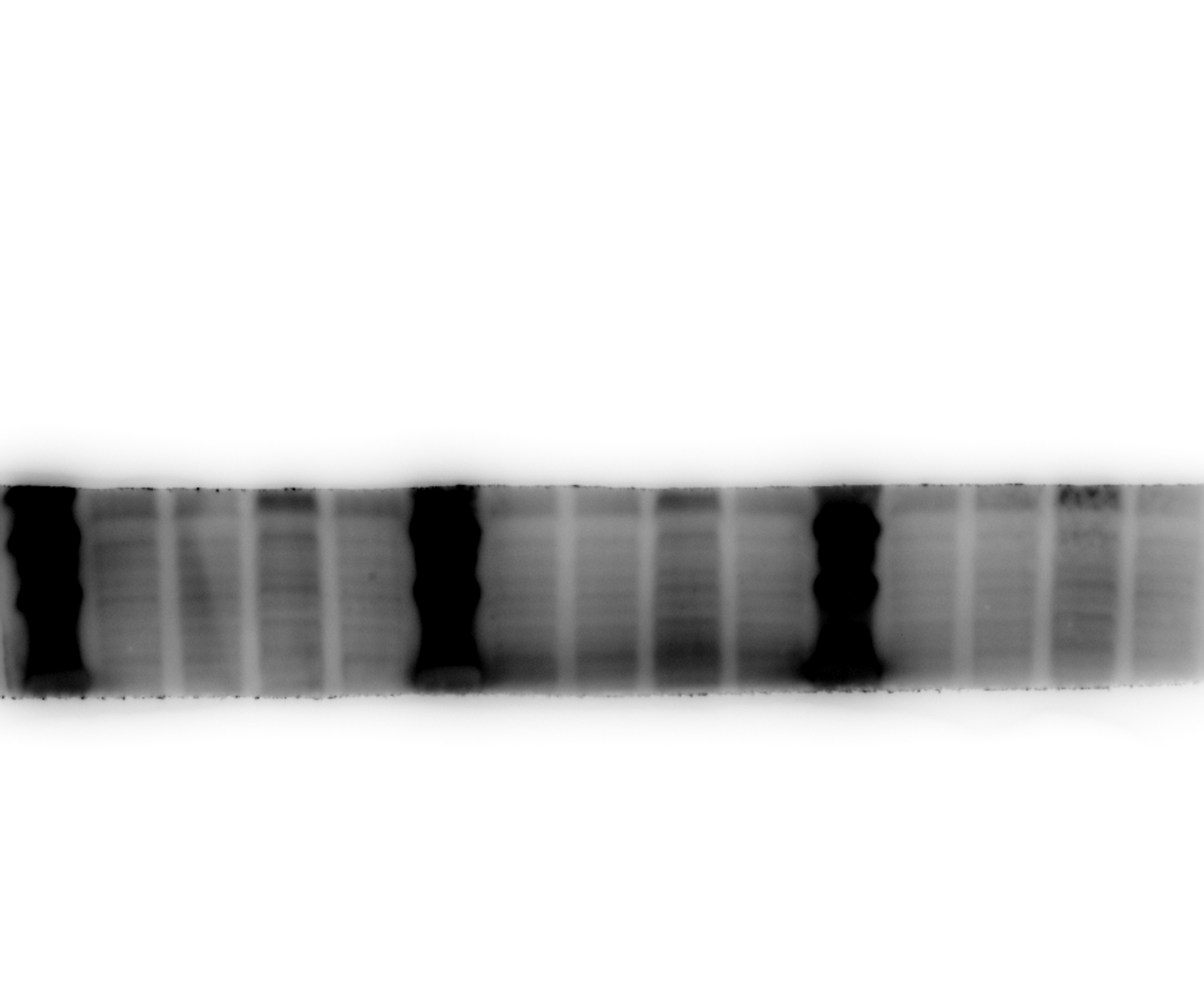

Supplement: Supplementary file 3 — Additional file 3. [file 13287_2026_4964_MOESM3_ESM.zip › Raw WB data 0809/PINK1P62LC3Bbeclin-1PARKINGAPDHsiRNA20250520/PARKIN-2.5S.Tif]

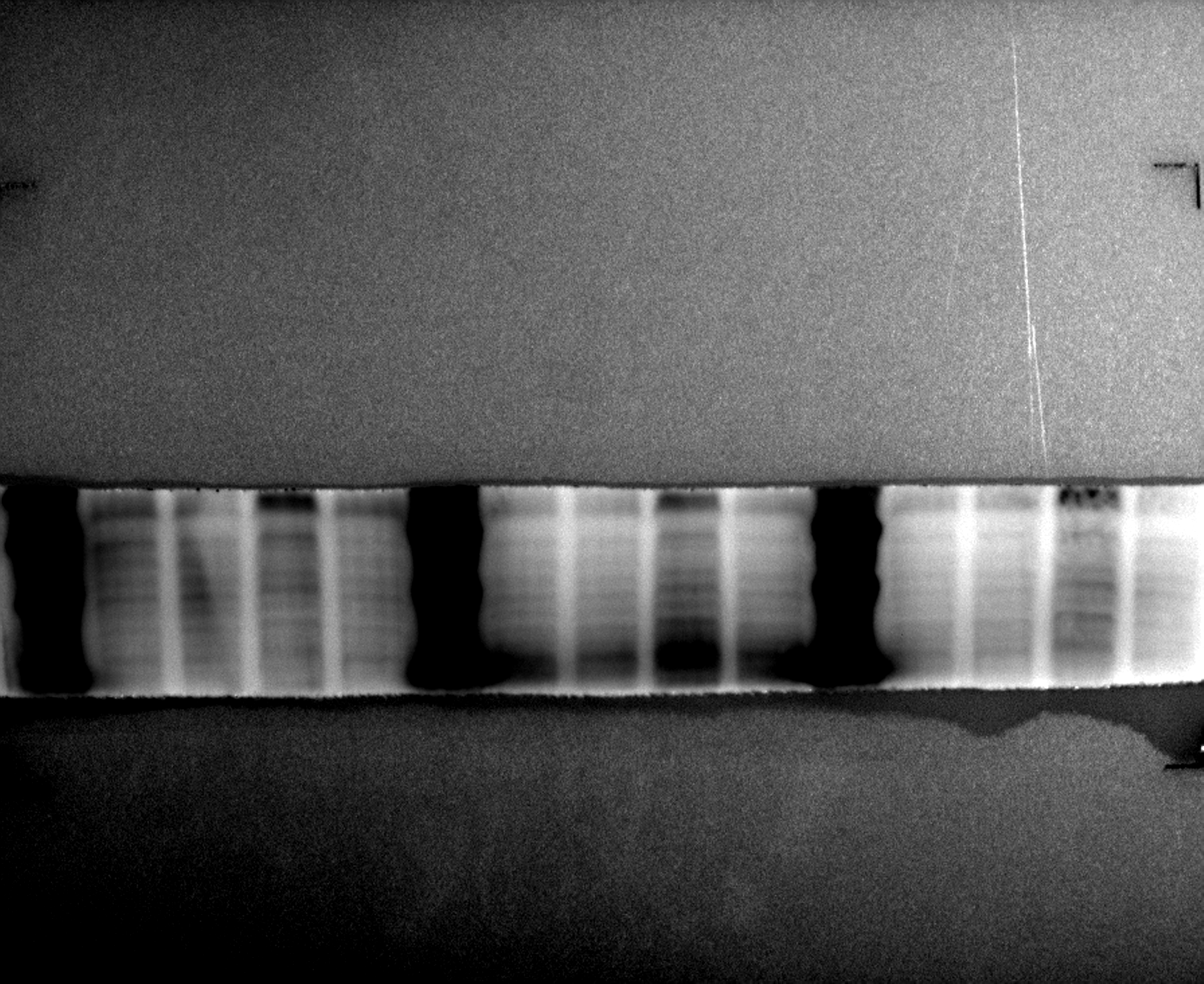

Supplement: Supplementary file 3 — Additional file 3. [file 13287_2026_4964_MOESM3_ESM.zip › Raw WB data 0809/PINK1P62LC3Bbeclin-1PARKINGAPDHsiRNA20250520/PARKIN-3S-R.Tif]

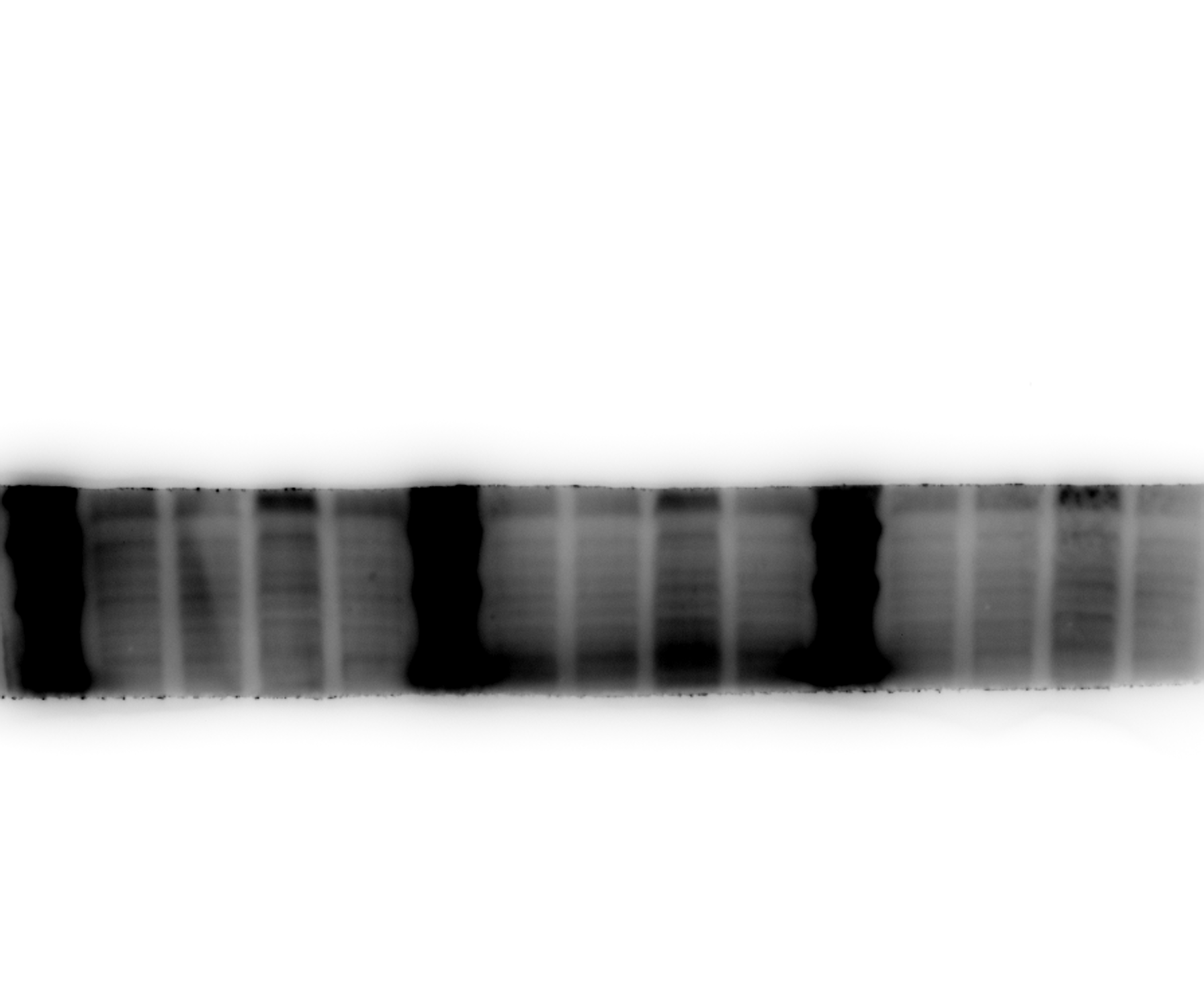

Supplement: Supplementary file 3 — Additional file 3. [file 13287_2026_4964_MOESM3_ESM.zip › Raw WB data 0809/PINK1P62LC3Bbeclin-1PARKINGAPDHsiRNA20250520/PARKIN-3S.Tif]

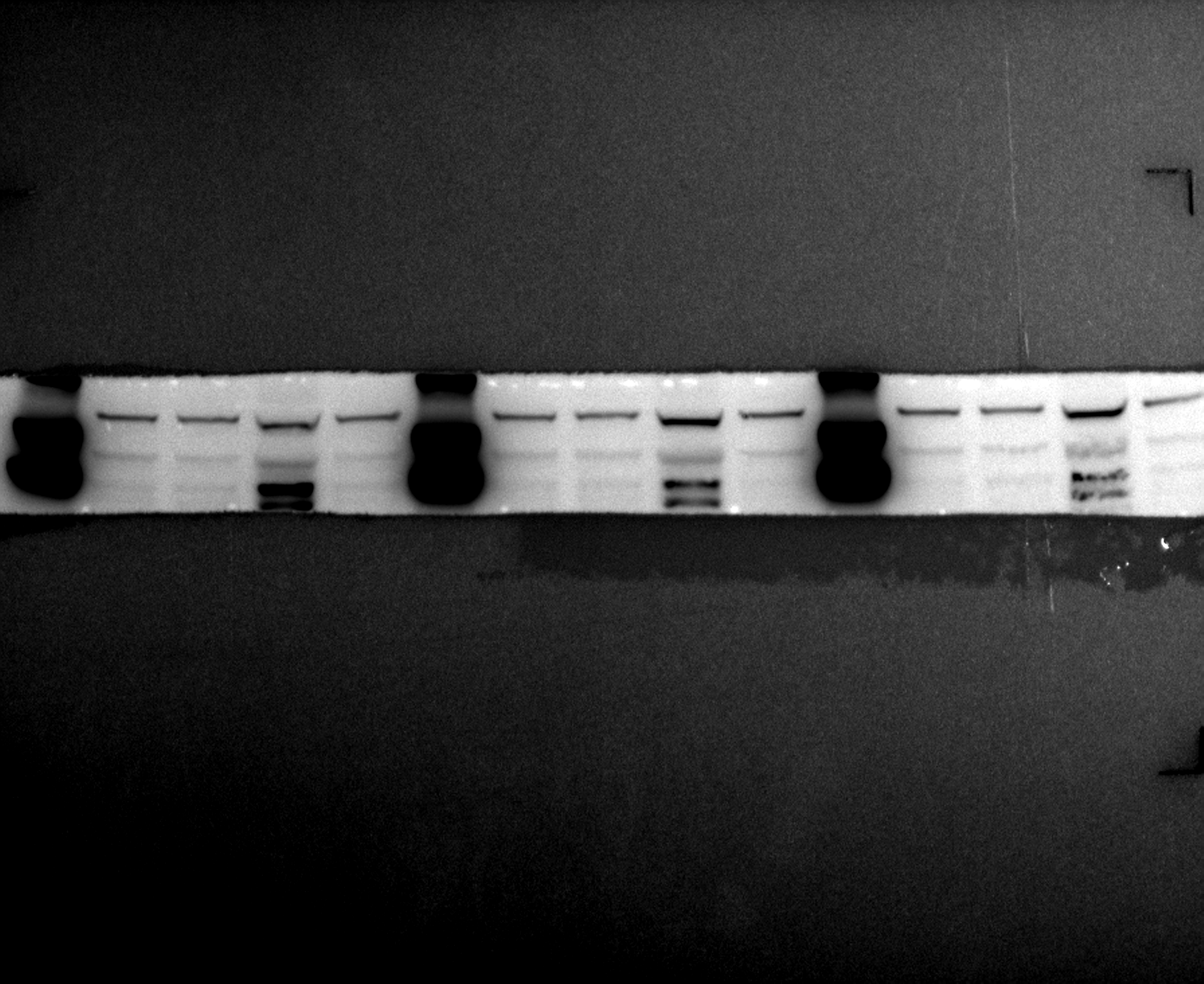

Supplement: Supplementary file 3 — Additional file 3. [file 13287_2026_4964_MOESM3_ESM.zip › Raw WB data 0809/PINK1P62LC3Bbeclin-1PARKINGAPDHsiRNA20250520/PINK-1-2s-r.Tif]

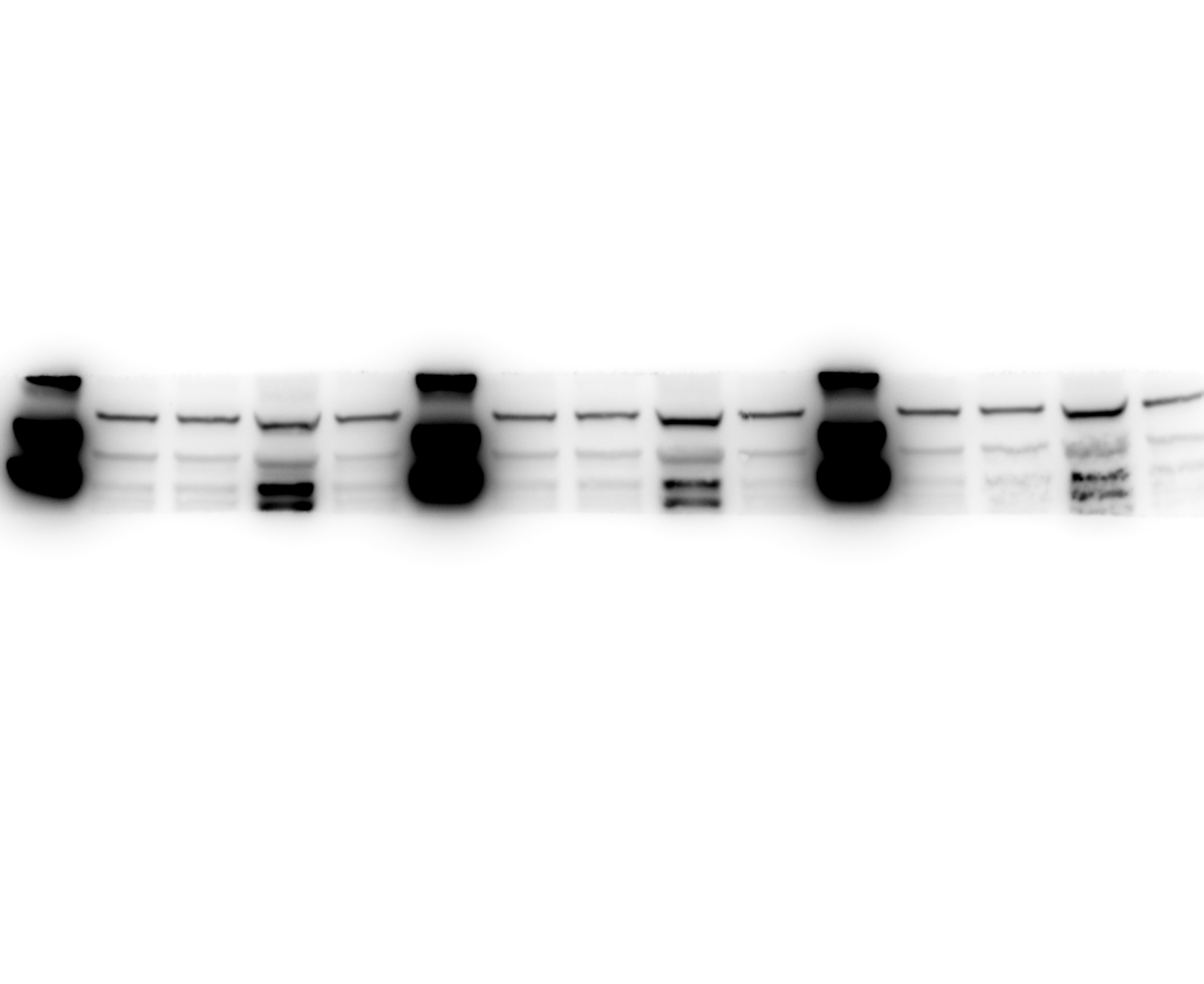

Supplement: Supplementary file 3 — Additional file 3. [file 13287_2026_4964_MOESM3_ESM.zip › Raw WB data 0809/PINK1P62LC3Bbeclin-1PARKINGAPDHsiRNA20250520/PINK1-2s.Tif]

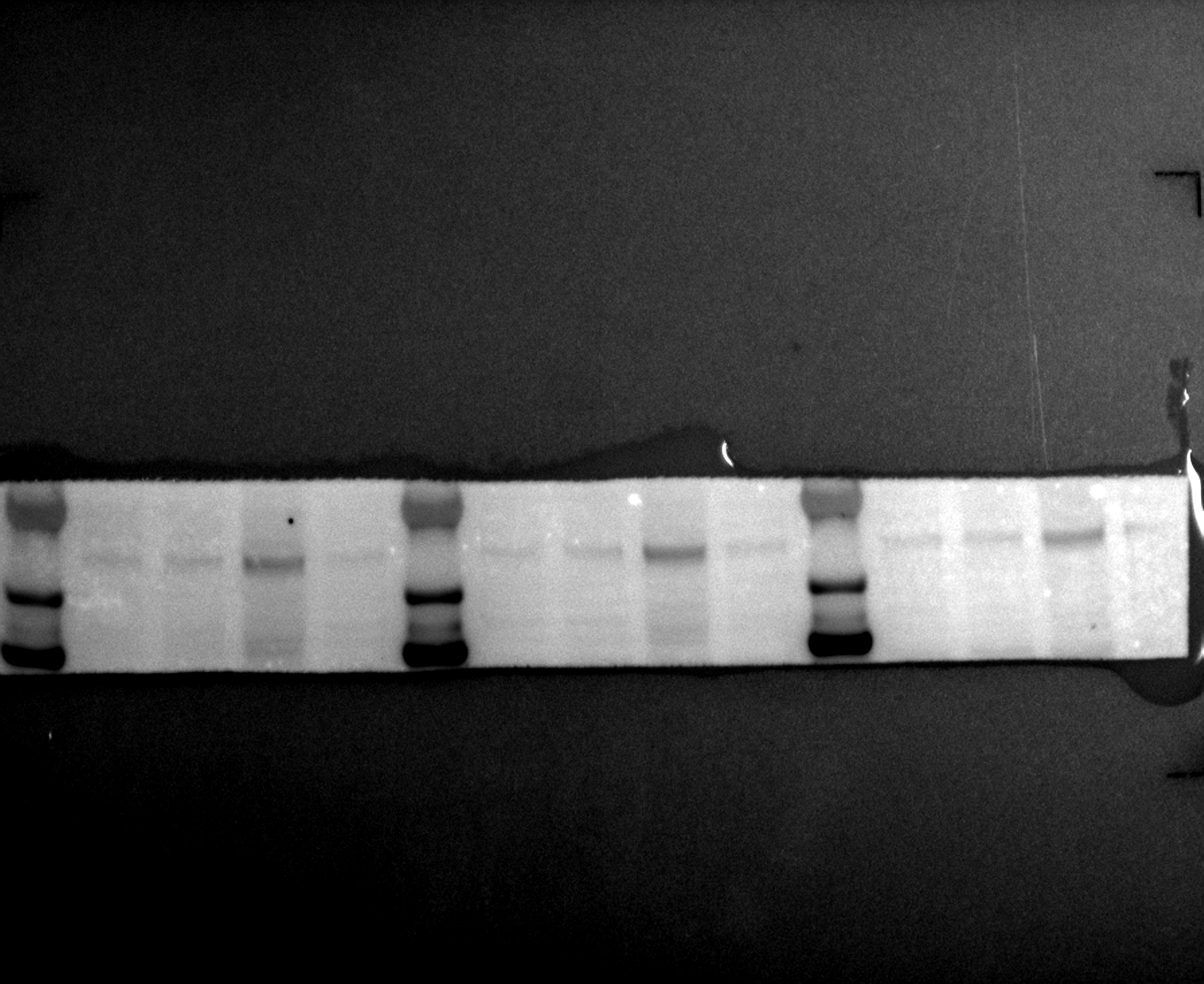

Supplement: Supplementary file 3 — Additional file 3. [file 13287_2026_4964_MOESM3_ESM.zip › Raw WB data 0809/PINK1P62LC3Bbeclin-1PARKINGAPDHsiRNA20250520/beclin1-5s-r.Tif]

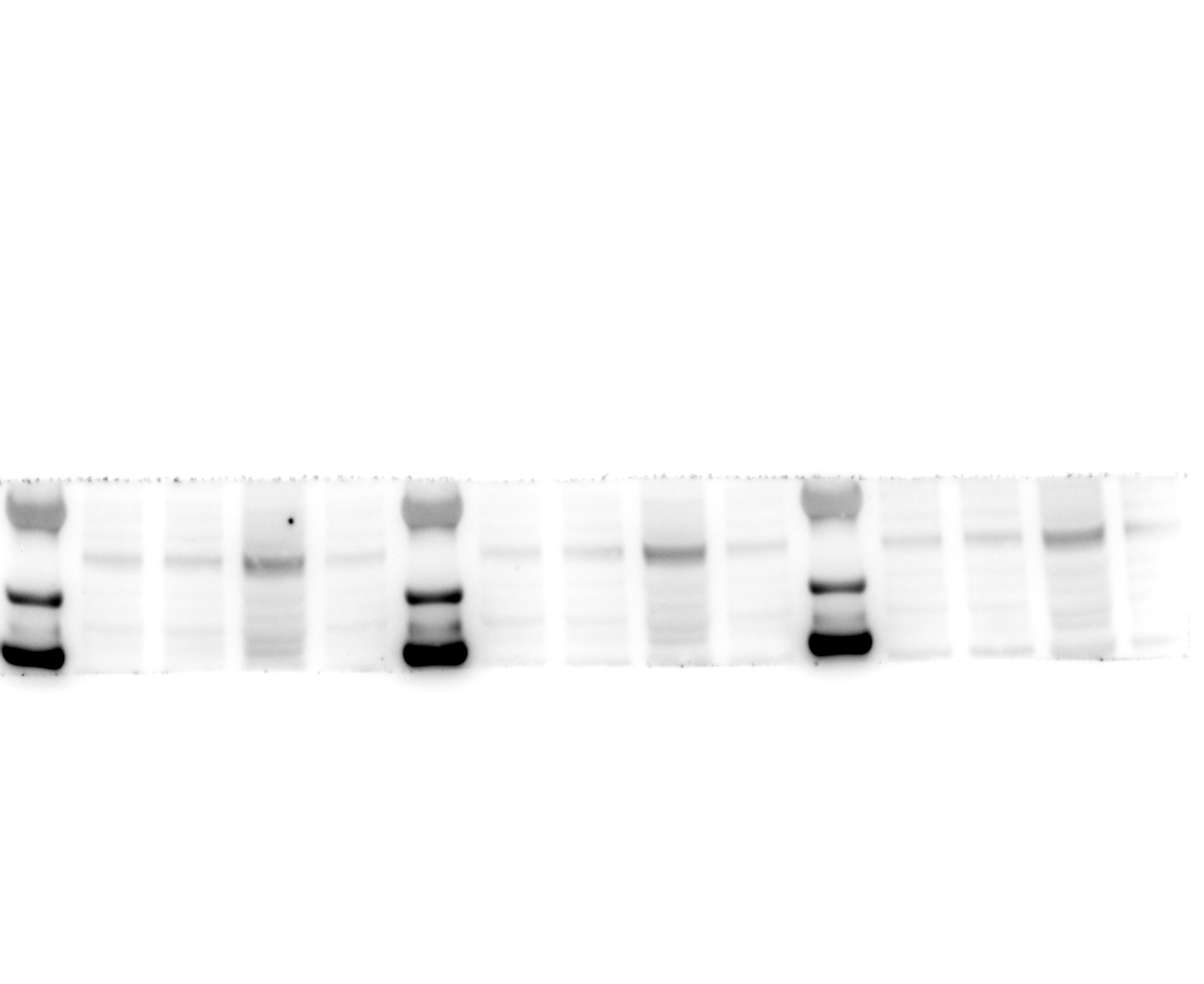

Supplement: Supplementary file 3 — Additional file 3. [file 13287_2026_4964_MOESM3_ESM.zip › Raw WB data 0809/PINK1P62LC3Bbeclin-1PARKINGAPDHsiRNA20250520/beclin1-5s.Tif]

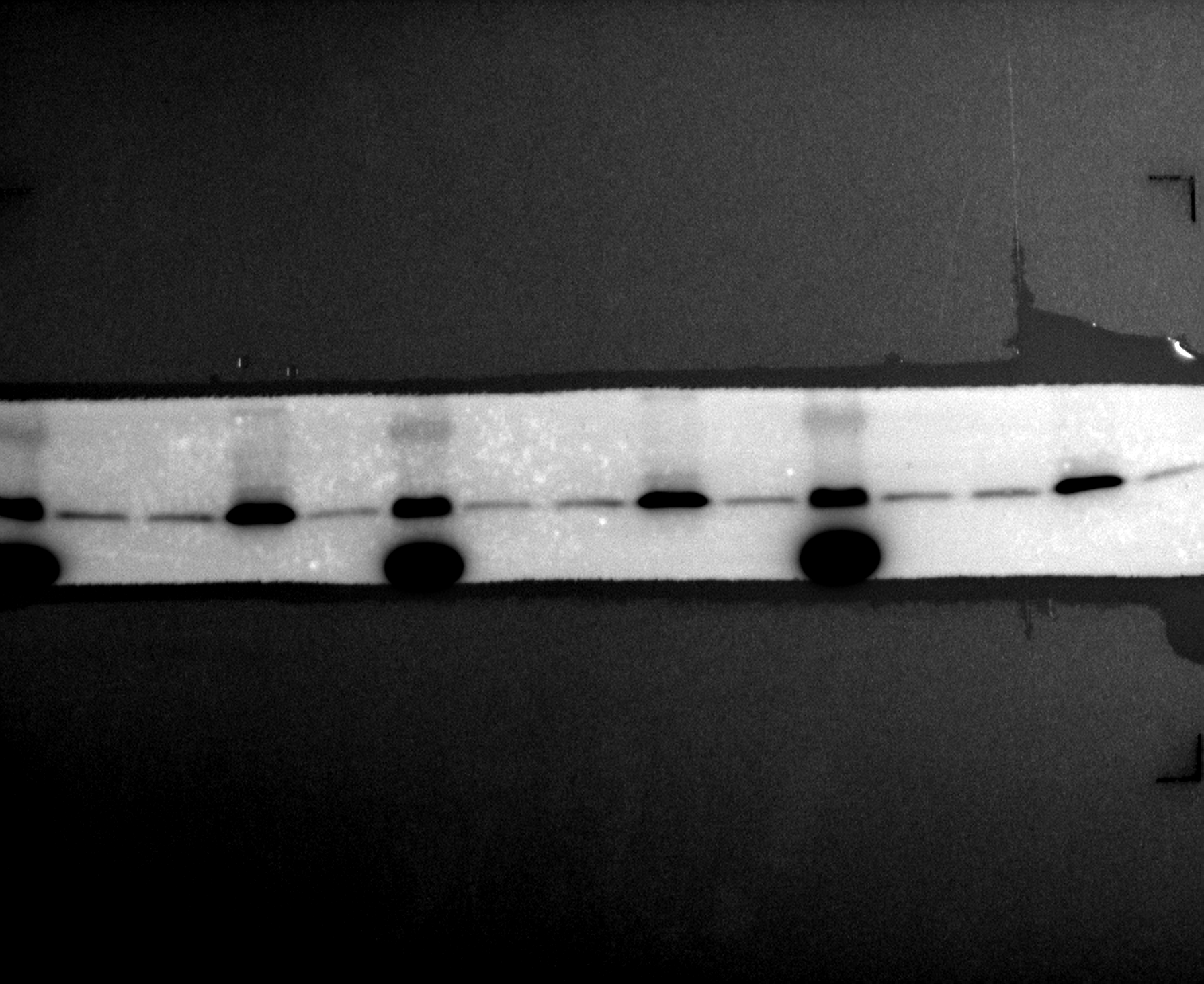

Supplement: Supplementary file 3 — Additional file 3. [file 13287_2026_4964_MOESM3_ESM.zip › Raw WB data 0809/PINK1P62LC3Bbeclin-1PARKINGAPDHsiRNA20250520/lc3-1s-r.Tif]

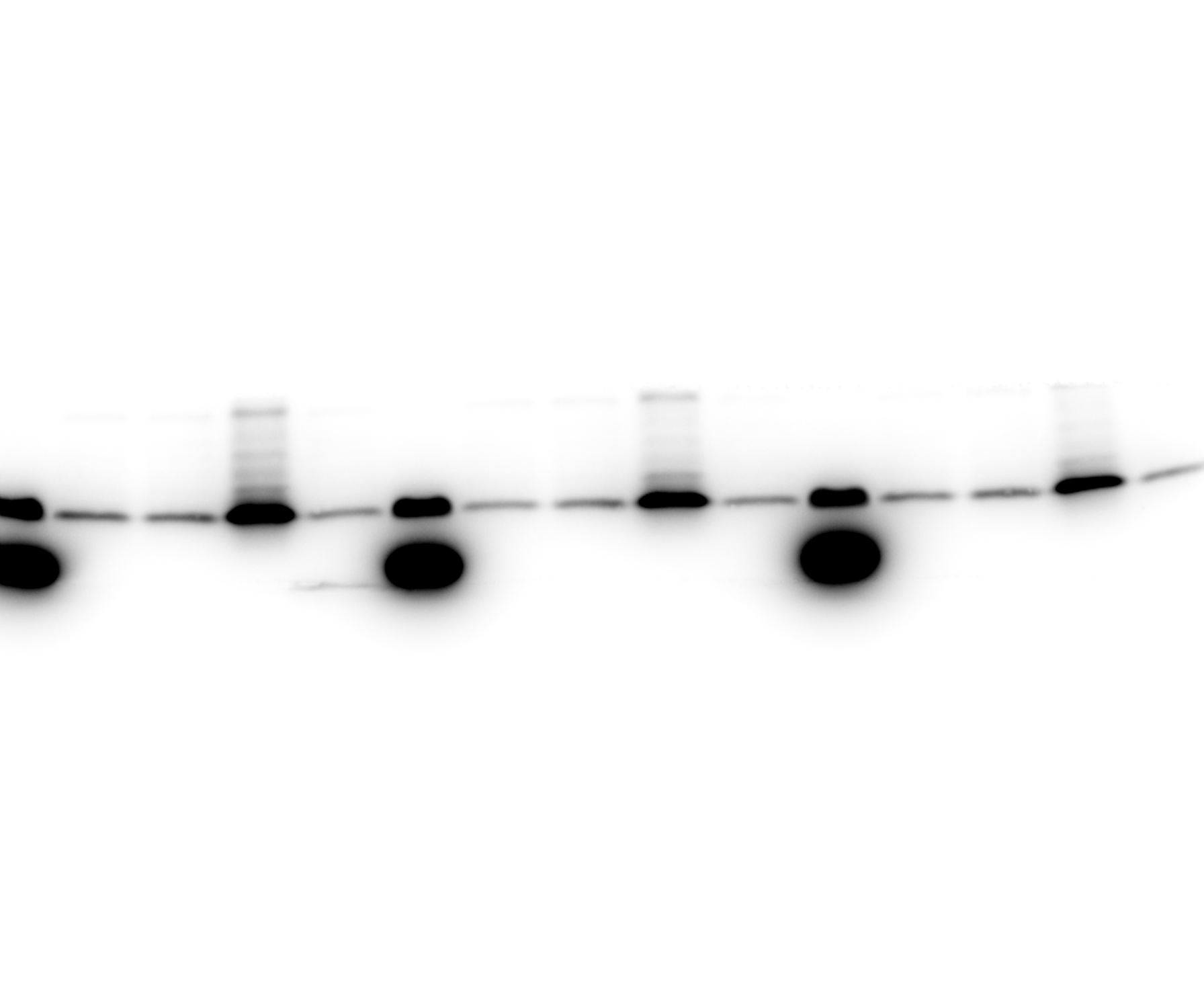

Supplement: Supplementary file 3 — Additional file 3. [file 13287_2026_4964_MOESM3_ESM.zip › Raw WB data 0809/PINK1P62LC3Bbeclin-1PARKINGAPDHsiRNA20250520/lc3-1s.Tif]

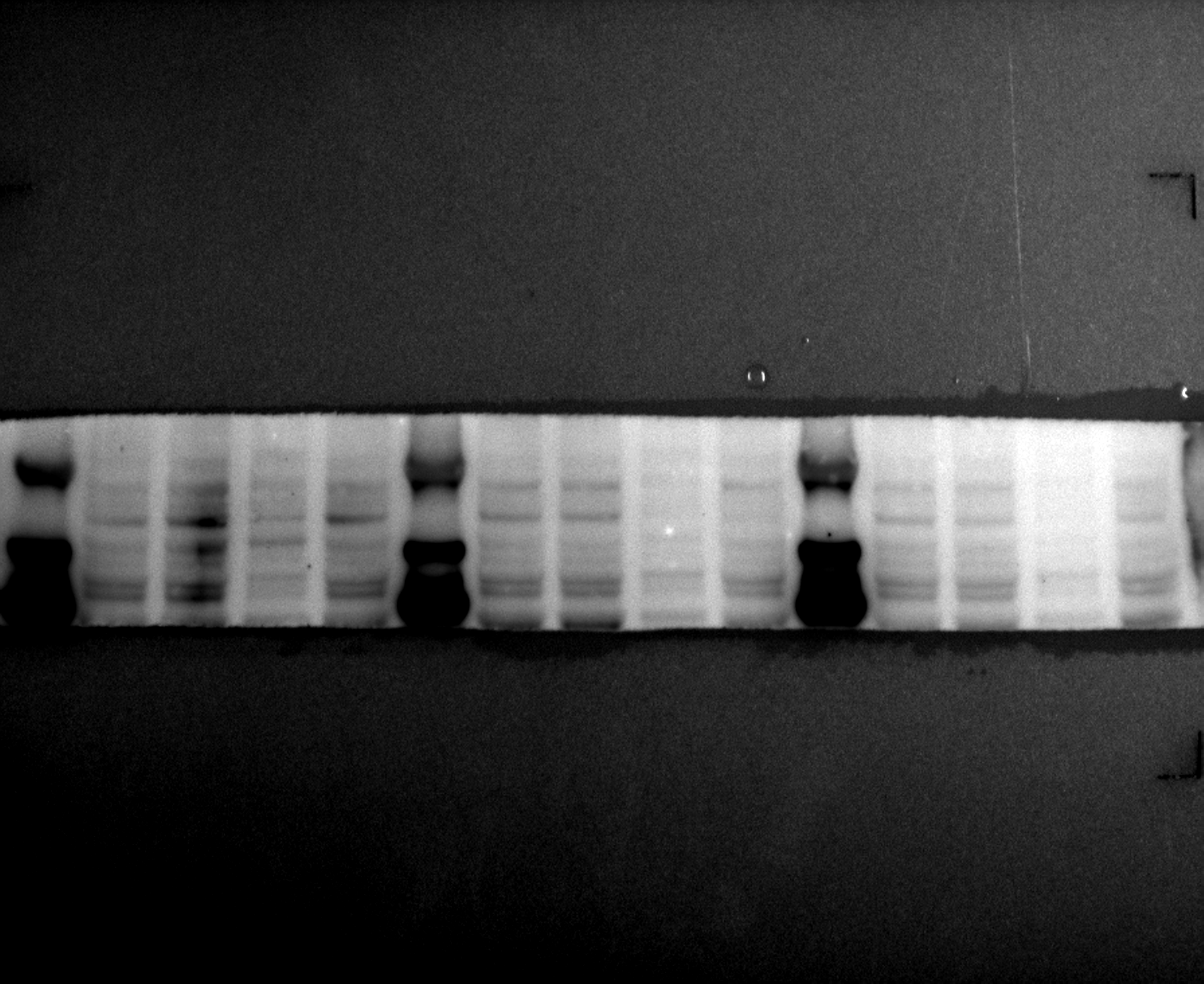

Supplement: Supplementary file 3 — Additional file 3. [file 13287_2026_4964_MOESM3_ESM.zip › Raw WB data 0809/PINK1P62LC3Bbeclin-1PARKINGAPDHsiRNA20250520/p62-30s-r.Tif]

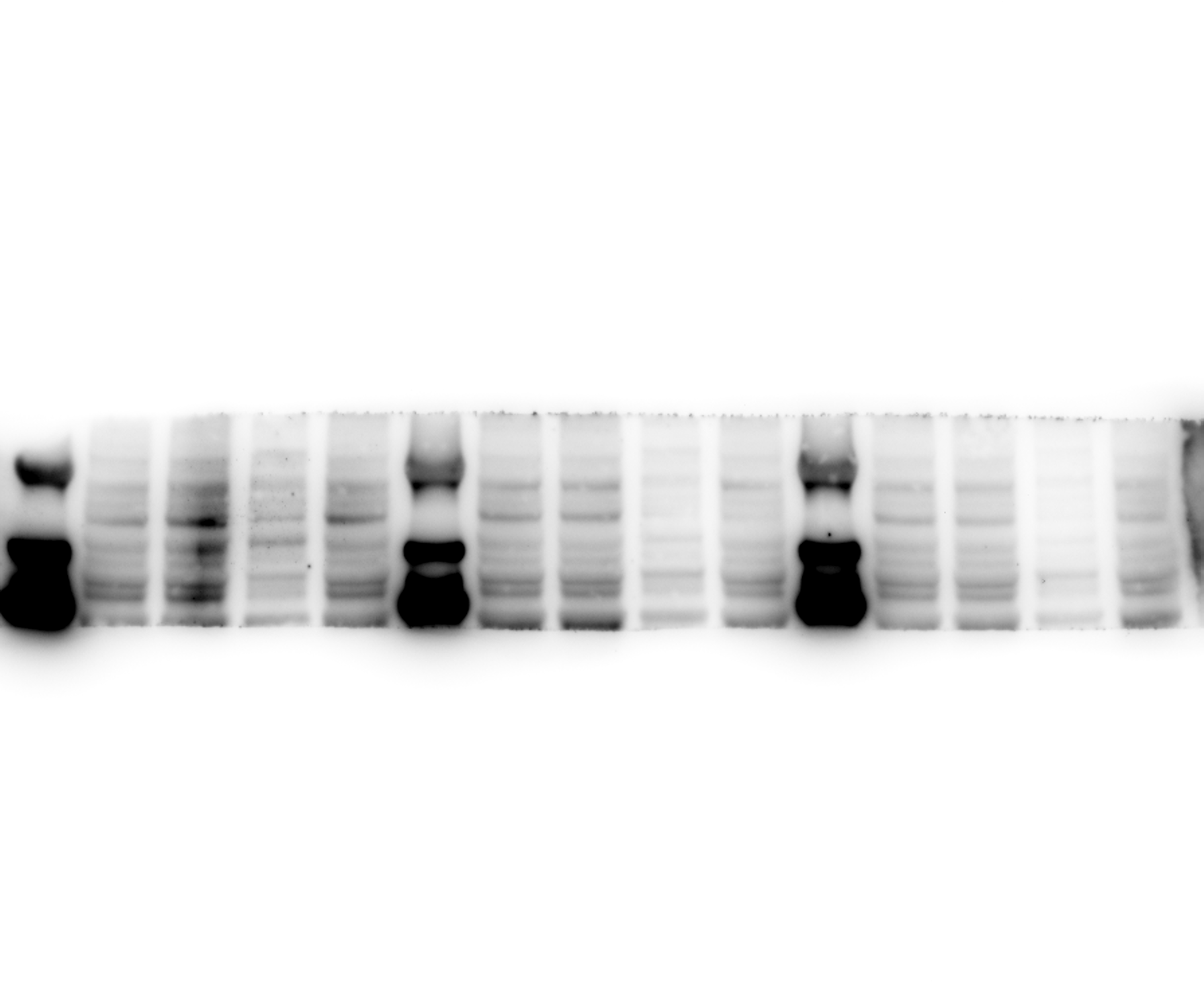

Supplement: Supplementary file 3 — Additional file 3. [file 13287_2026_4964_MOESM3_ESM.zip › Raw WB data 0809/PINK1P62LC3Bbeclin-1PARKINGAPDHsiRNA20250520/p62-30s.Tif]

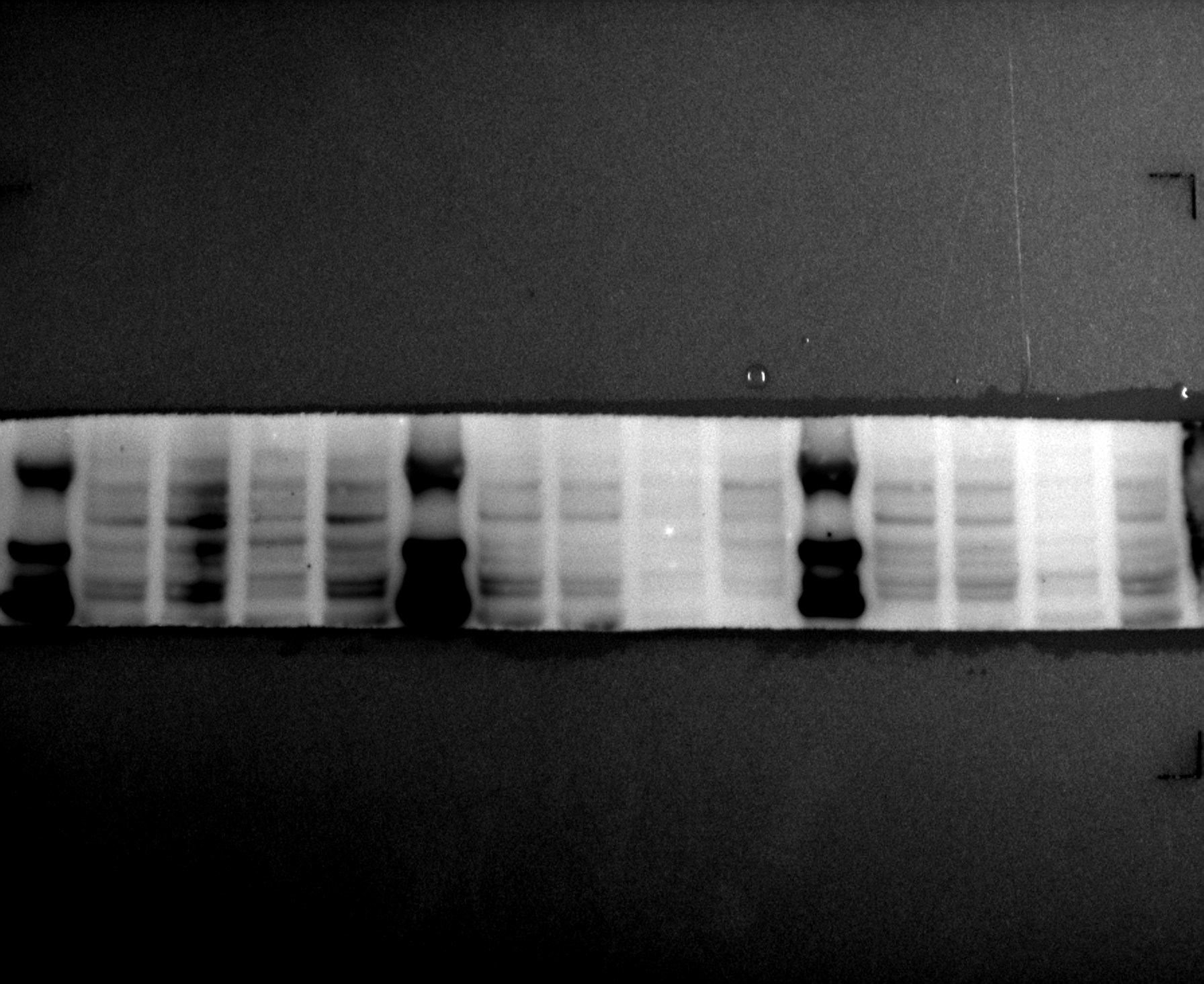

Supplement: Supplementary file 3 — Additional file 3. [file 13287_2026_4964_MOESM3_ESM.zip › Raw WB data 0809/PINK1P62LC3Bbeclin-1PARKINGAPDHsiRNA20250520/p62-40s-r.Tif]

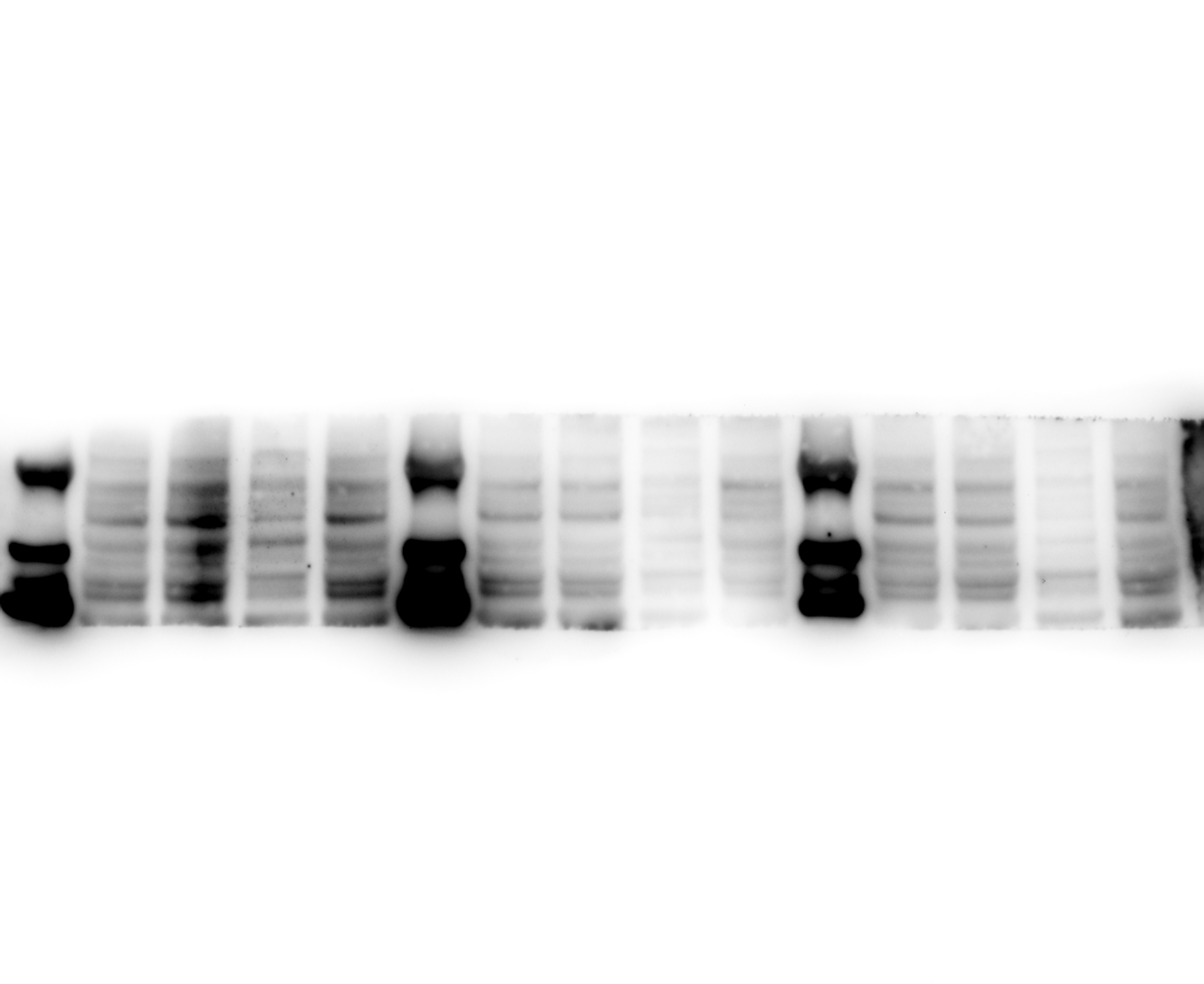

Supplement: Supplementary file 3 — Additional file 3. [file 13287_2026_4964_MOESM3_ESM.zip › Raw WB data 0809/PINK1P62LC3Bbeclin-1PARKINGAPDHsiRNA20250520/p62-40s.Tif]

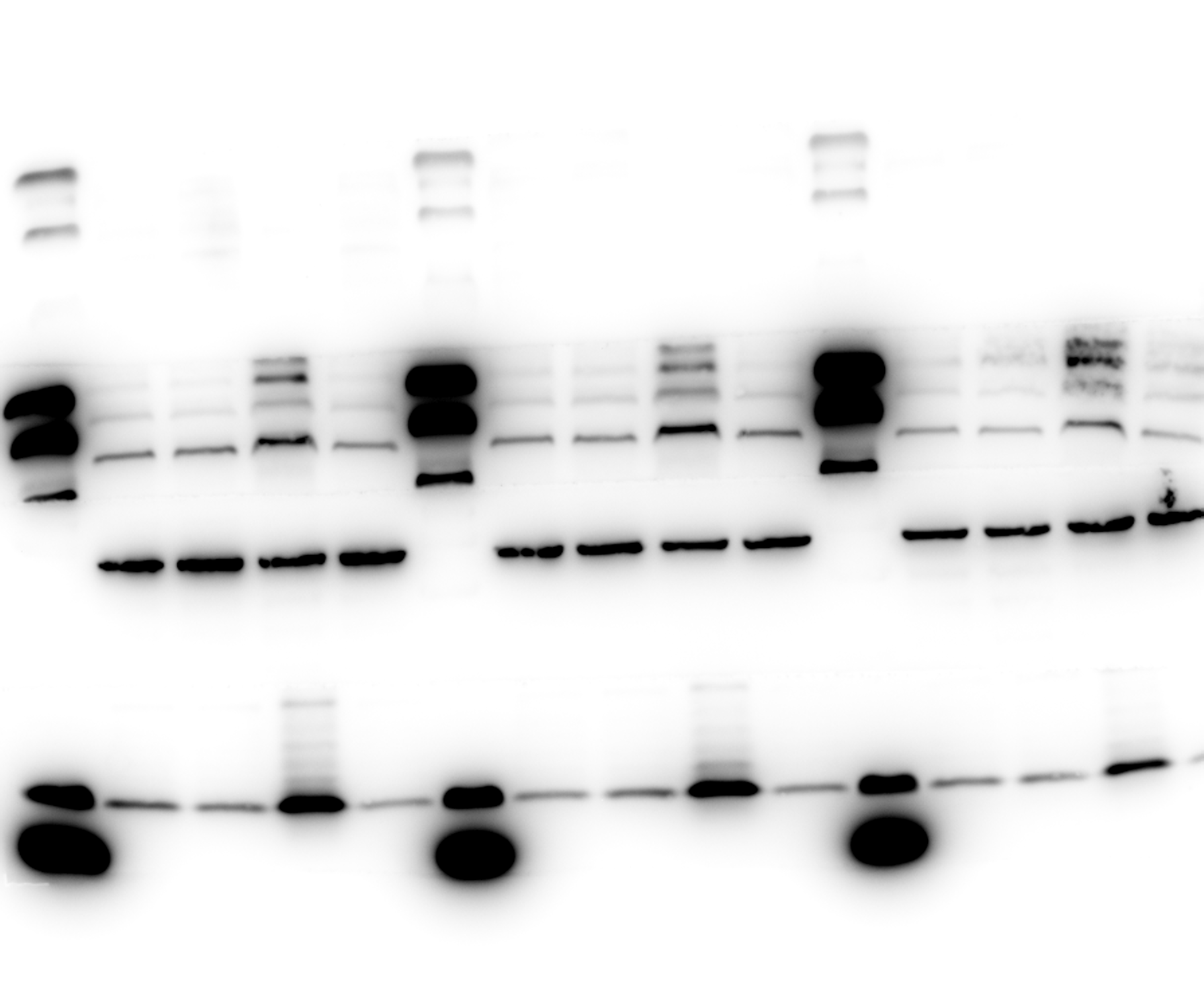

Supplement: Supplementary file 3 — Additional file 3. [file 13287_2026_4964_MOESM3_ESM.zip › Raw WB data 0809/PINK1P62LC3Bbeclin-1PARKINGAPDHsiRNA20250520/全膜-p62.Tif]

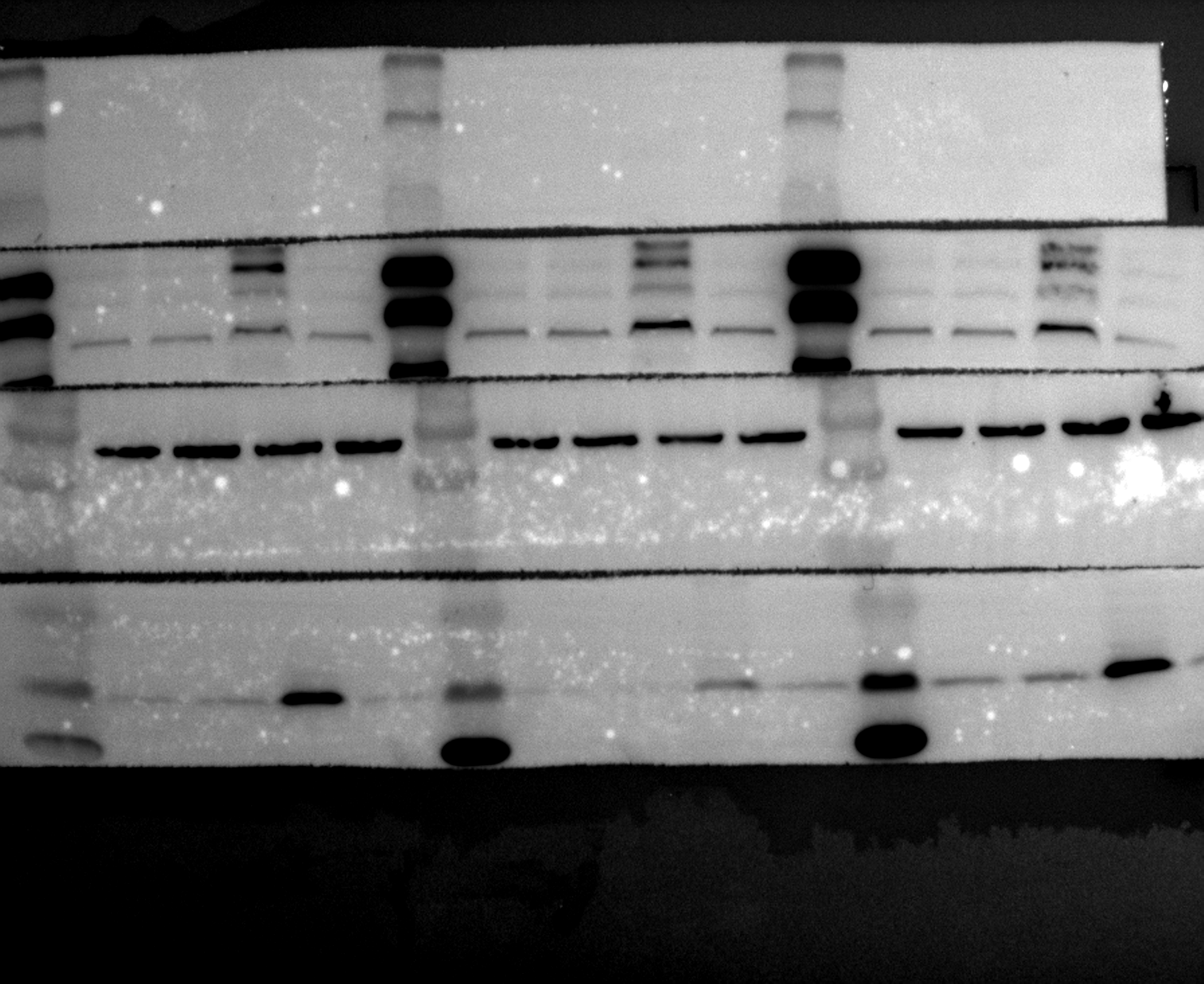

Supplement: Supplementary file 3 — Additional file 3. [file 13287_2026_4964_MOESM3_ESM.zip › Raw WB data 0809/PINK1P62LC3Bbeclin-1PARKINGAPDHsiRNA20250520/全膜1-beclin1-r.Tif]

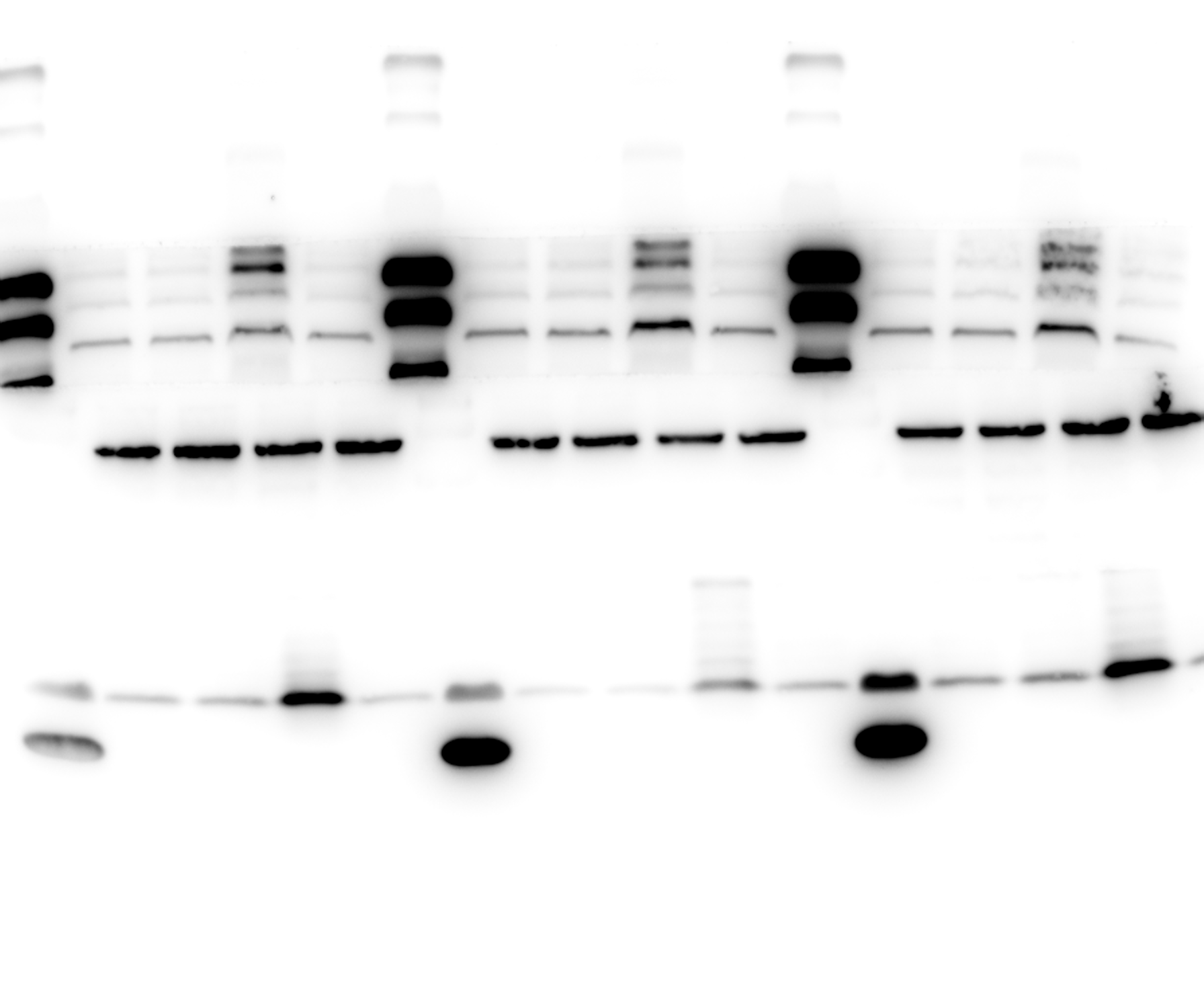

Supplement: Supplementary file 3 — Additional file 3. [file 13287_2026_4964_MOESM3_ESM.zip › Raw WB data 0809/PINK1P62LC3Bbeclin-1PARKINGAPDHsiRNA20250520/全膜1-beclin1.Tif]

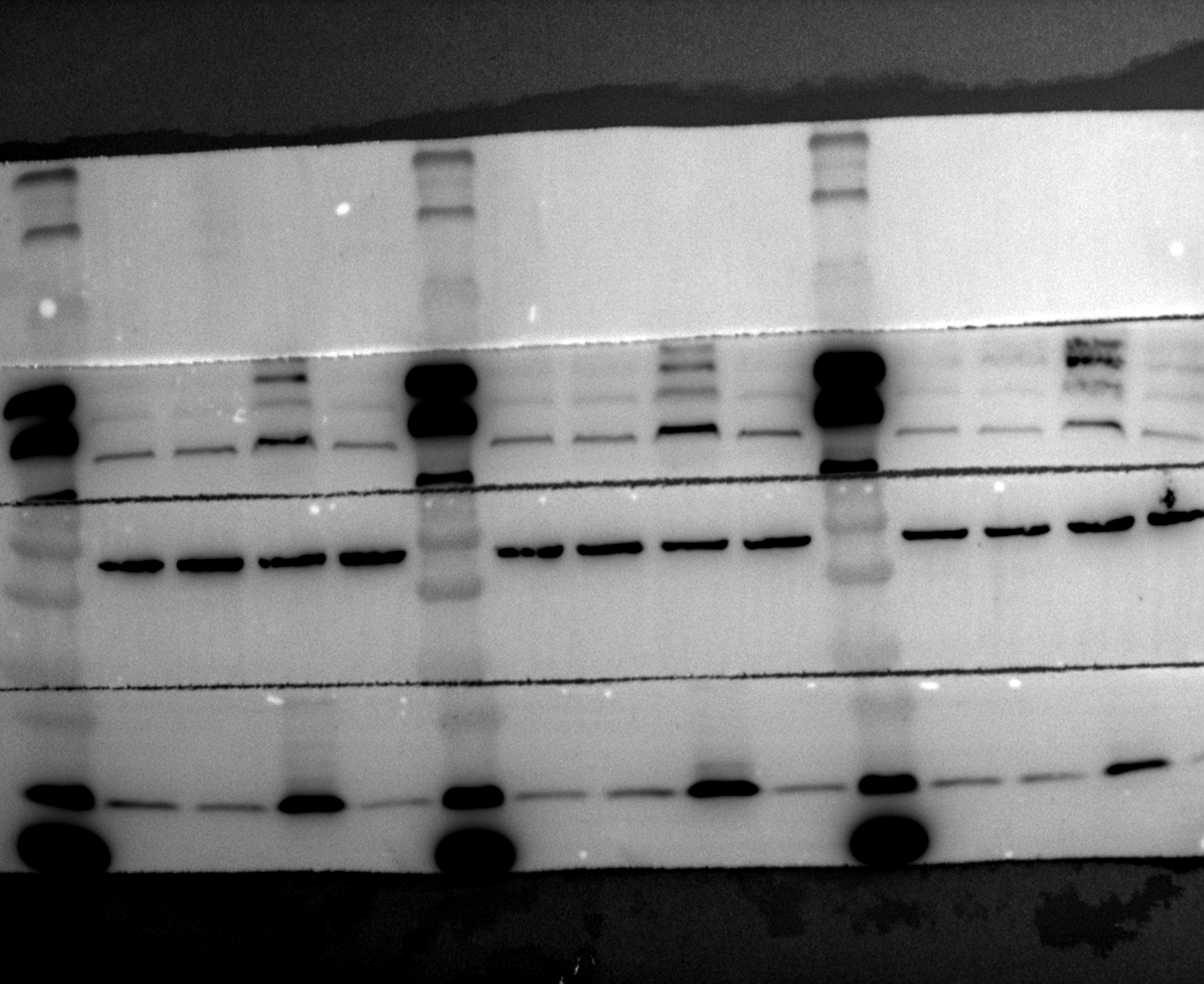

Supplement: Supplementary file 3 — Additional file 3. [file 13287_2026_4964_MOESM3_ESM.zip › Raw WB data 0809/PINK1P62LC3Bbeclin-1PARKINGAPDHsiRNA20250520/全膜1-r-p62.Tif]

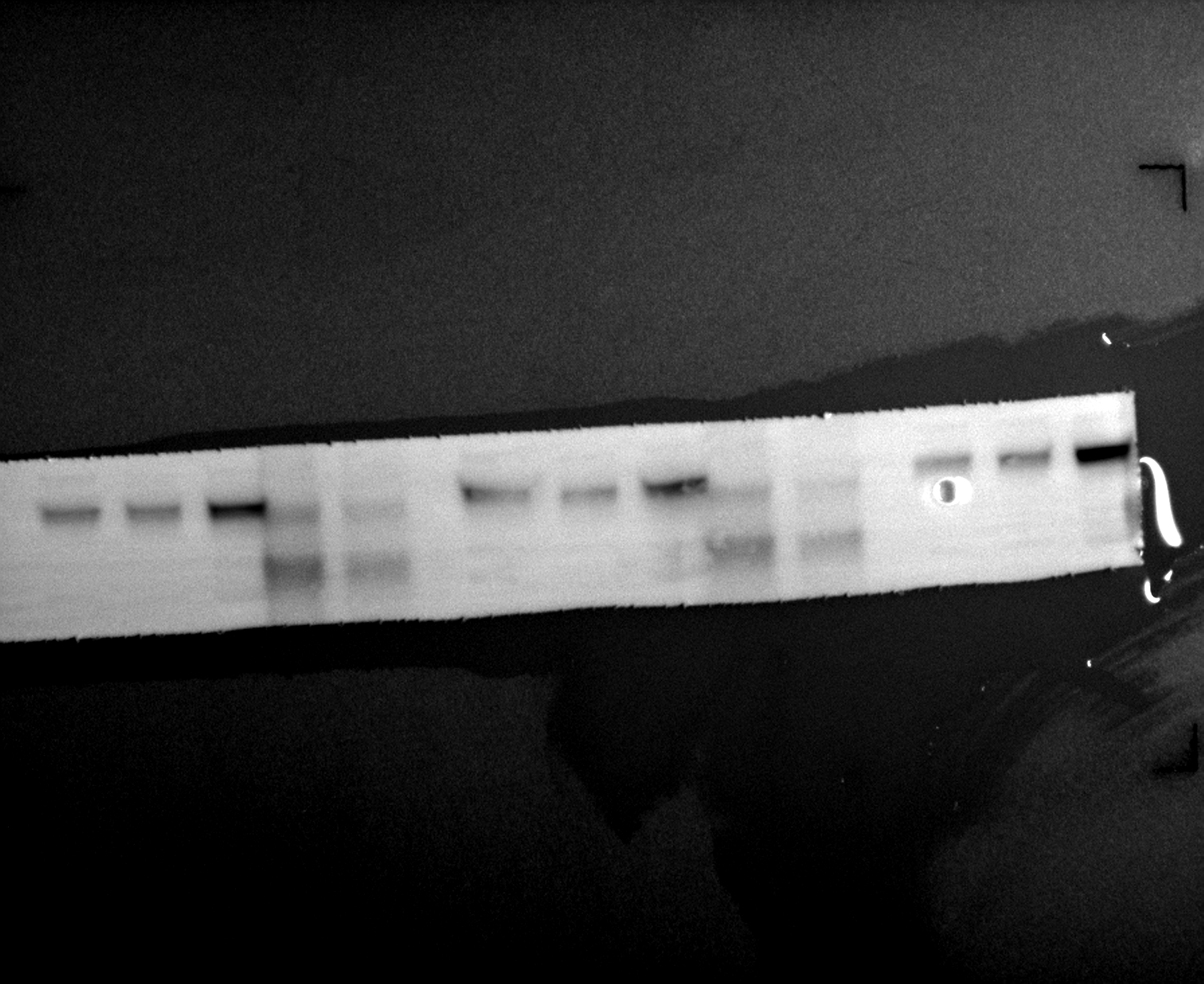

Supplement: Supplementary file 3 — Additional file 3. [file 13287_2026_4964_MOESM3_ESM.zip › Raw WB data 0809/PINK1ParkinLC3BBeclin-1P62 HaCaT/BECLIN1全膜24.10.06.x3.tif]

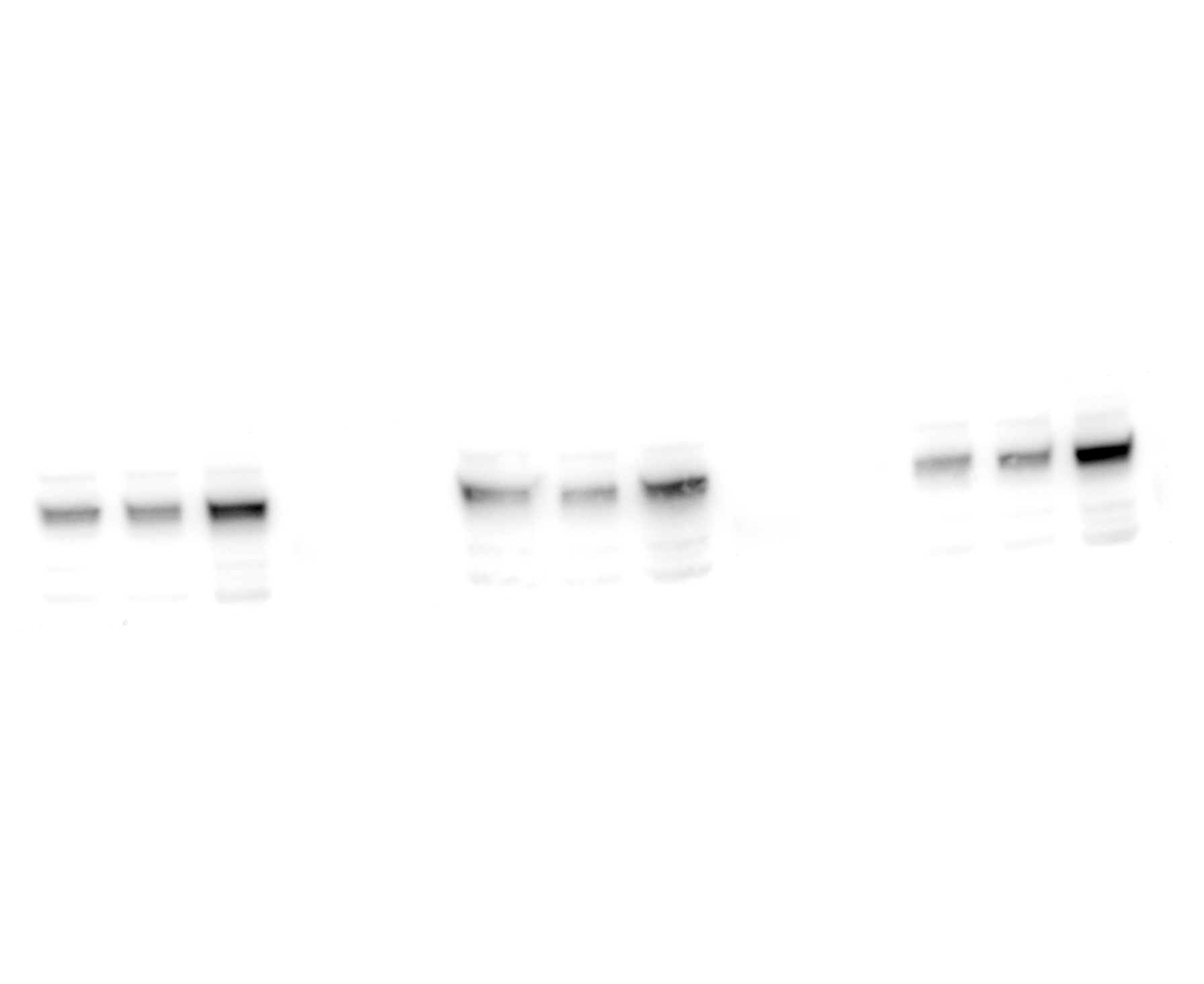

Supplement: Supplementary file 3 — Additional file 3. [file 13287_2026_4964_MOESM3_ESM.zip › Raw WB data 0809/PINK1ParkinLC3BBeclin-1P62 HaCaT/Beclin-1-2s人x.Tif]

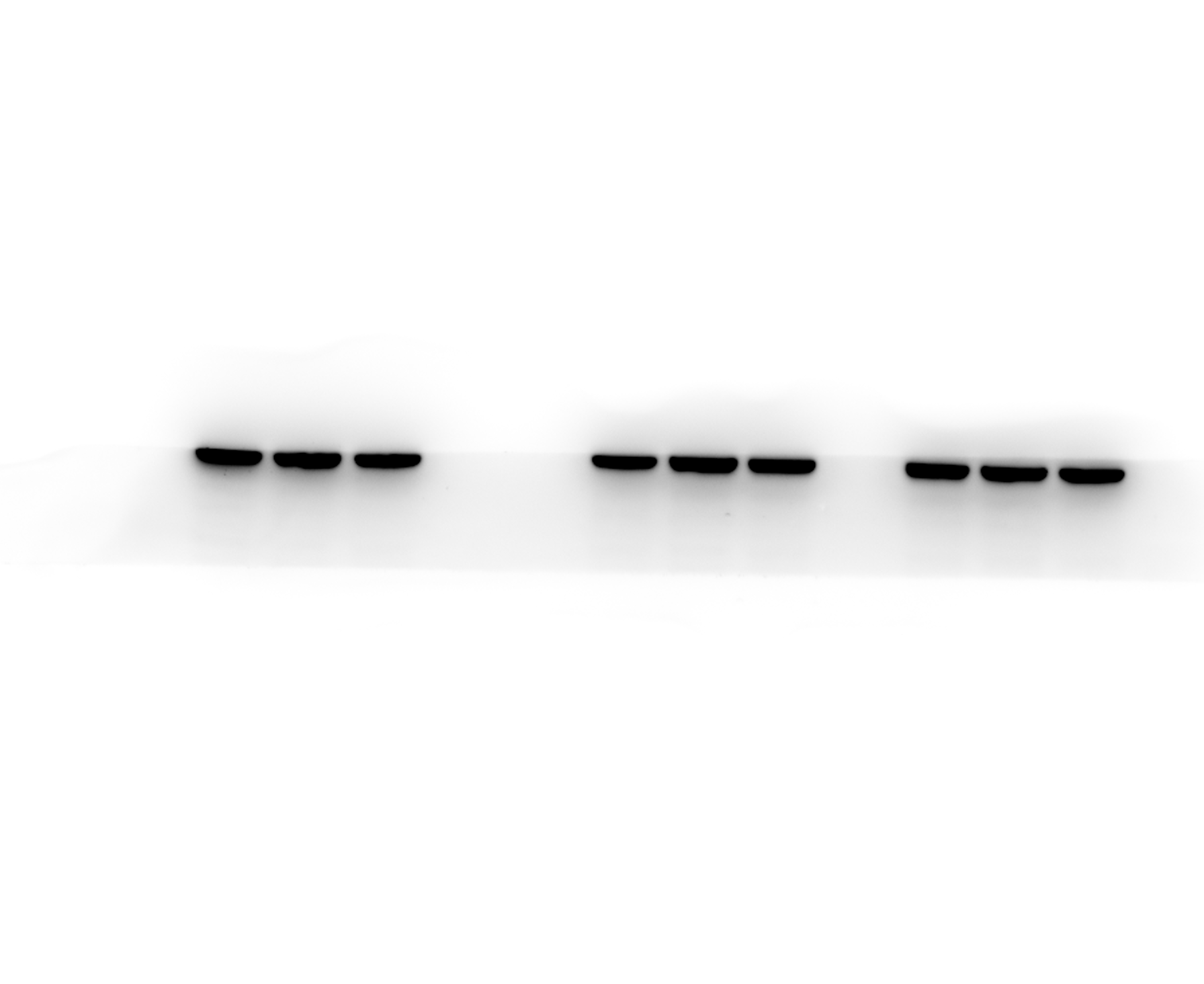

Supplement: Supplementary file 3 — Additional file 3. [file 13287_2026_4964_MOESM3_ESM.zip › Raw WB data 0809/PINK1ParkinLC3BBeclin-1P62 HaCaT/GAPDH-800ms.Tif]

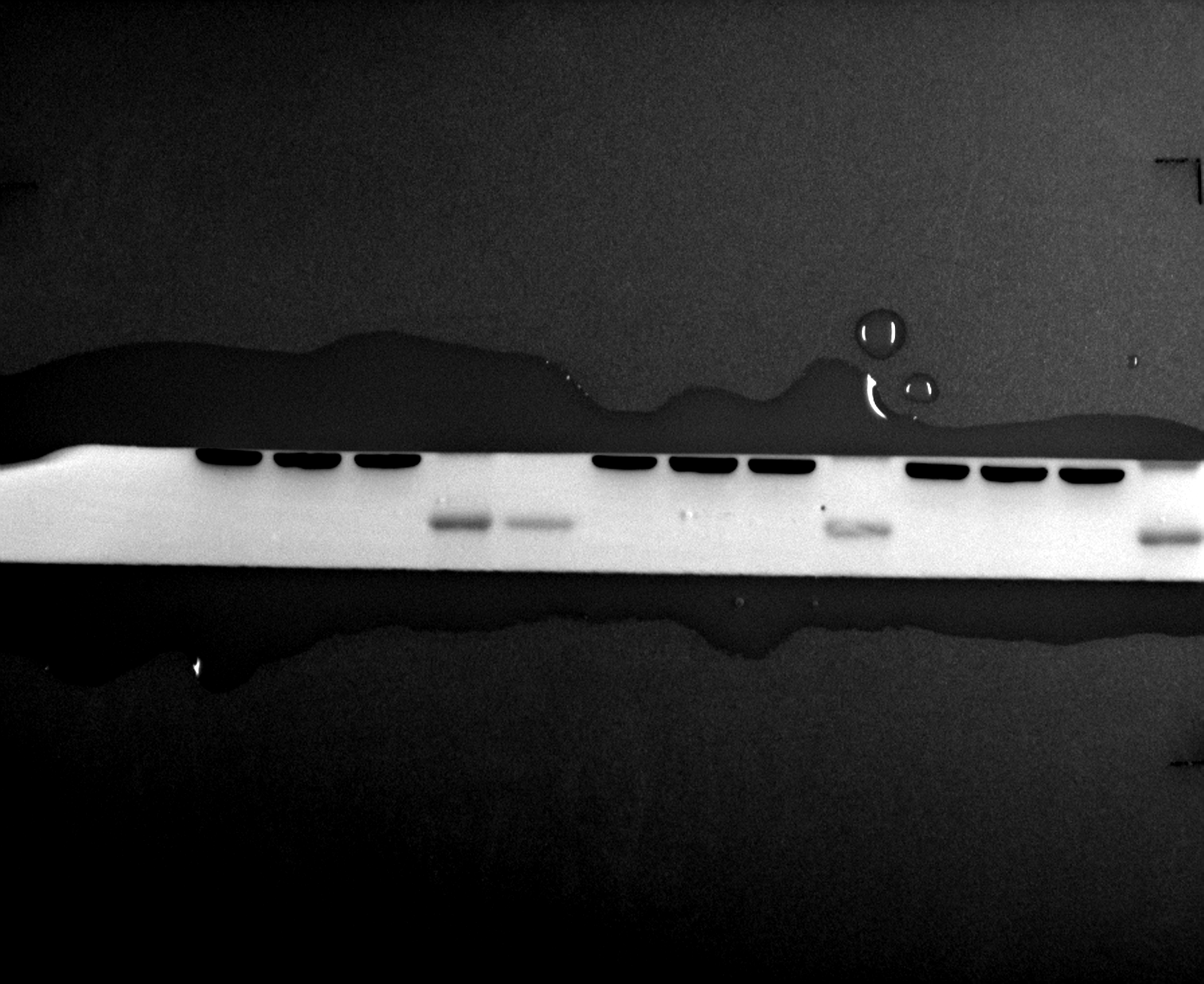

Supplement: Supplementary file 3 — Additional file 3. [file 13287_2026_4964_MOESM3_ESM.zip › Raw WB data 0809/PINK1ParkinLC3BBeclin-1P62 HaCaT/GAPDH全膜.Tif]

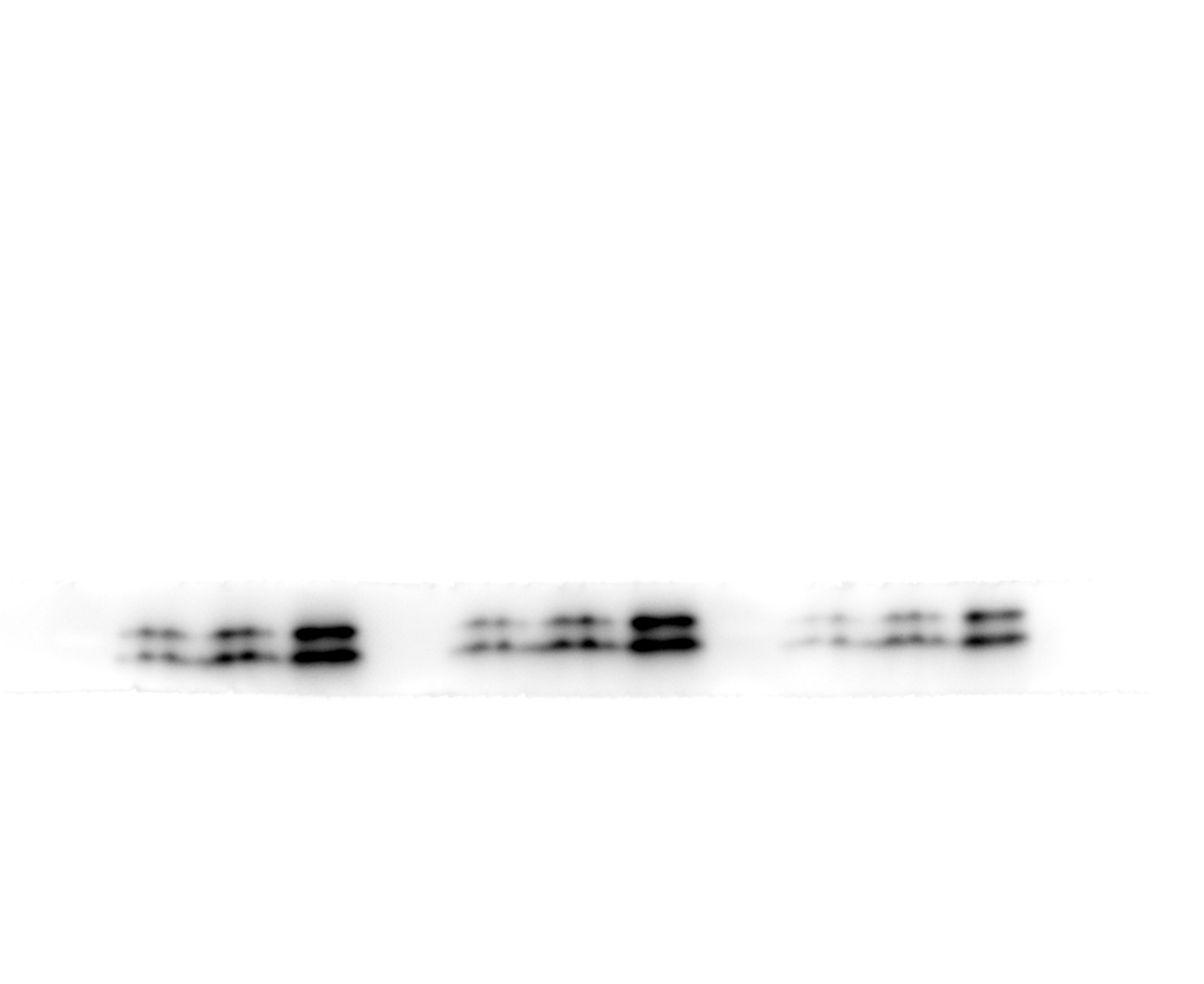

Supplement: Supplementary file 3 — Additional file 3. [file 13287_2026_4964_MOESM3_ESM.zip › Raw WB data 0809/PINK1ParkinLC3BBeclin-1P62 HaCaT/LC3B 2s.Tif]

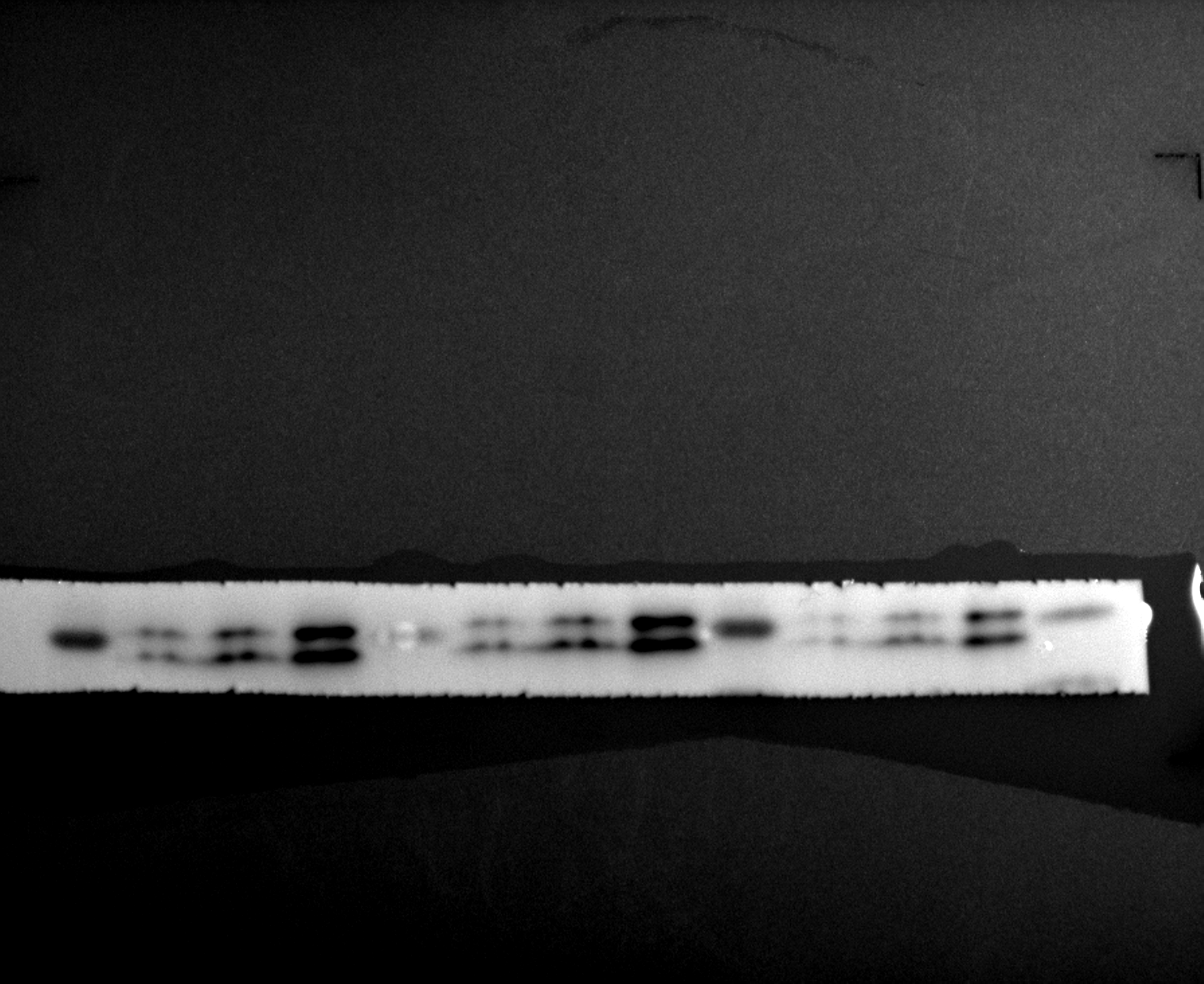

Supplement: Supplementary file 3 — Additional file 3. [file 13287_2026_4964_MOESM3_ESM.zip › Raw WB data 0809/PINK1ParkinLC3BBeclin-1P62 HaCaT/LC3B全膜2.Tif]

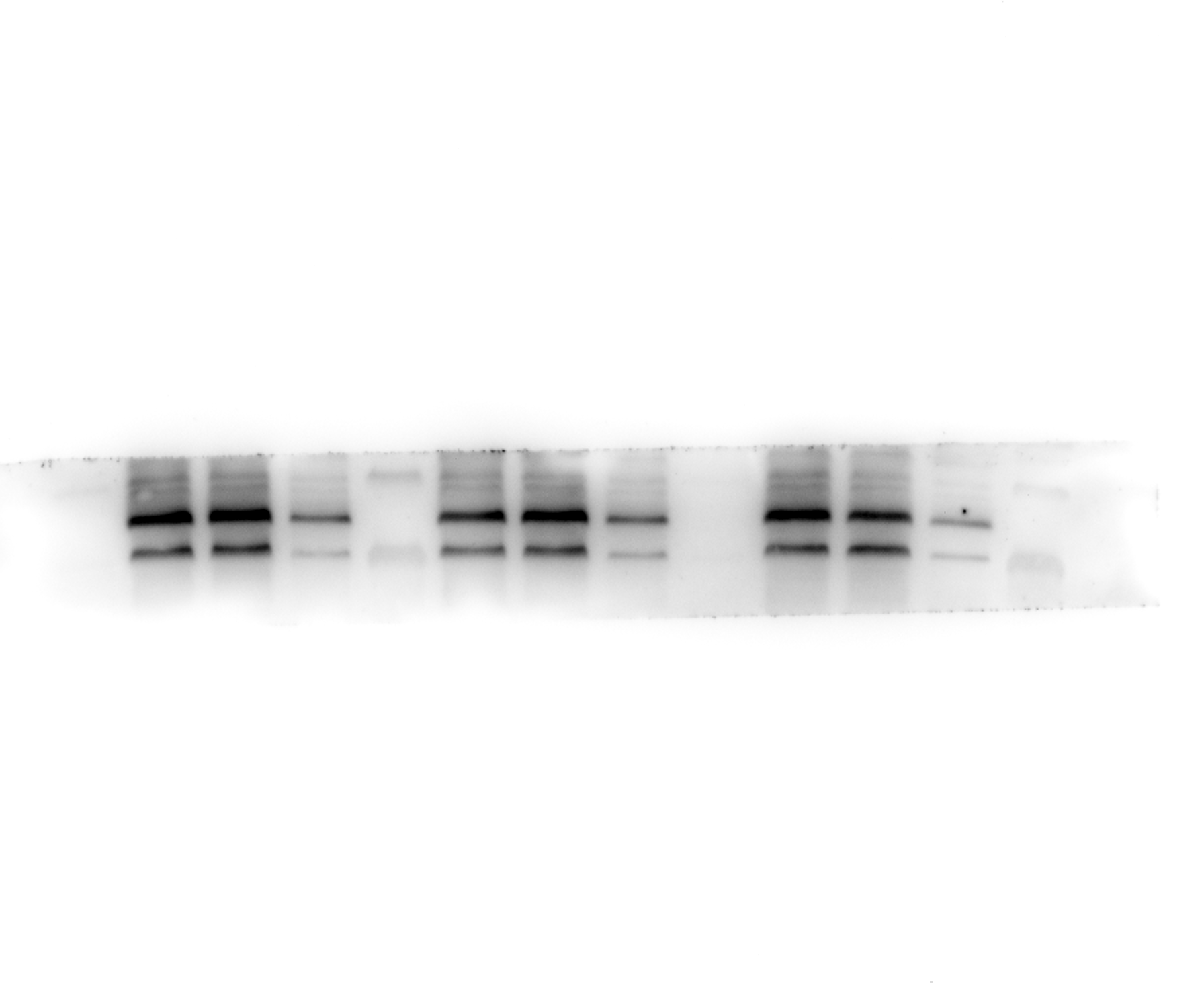

Supplement: Supplementary file 3 — Additional file 3. [file 13287_2026_4964_MOESM3_ESM.zip › Raw WB data 0809/PINK1ParkinLC3BBeclin-1P62 HaCaT/P62+30s.Tif]

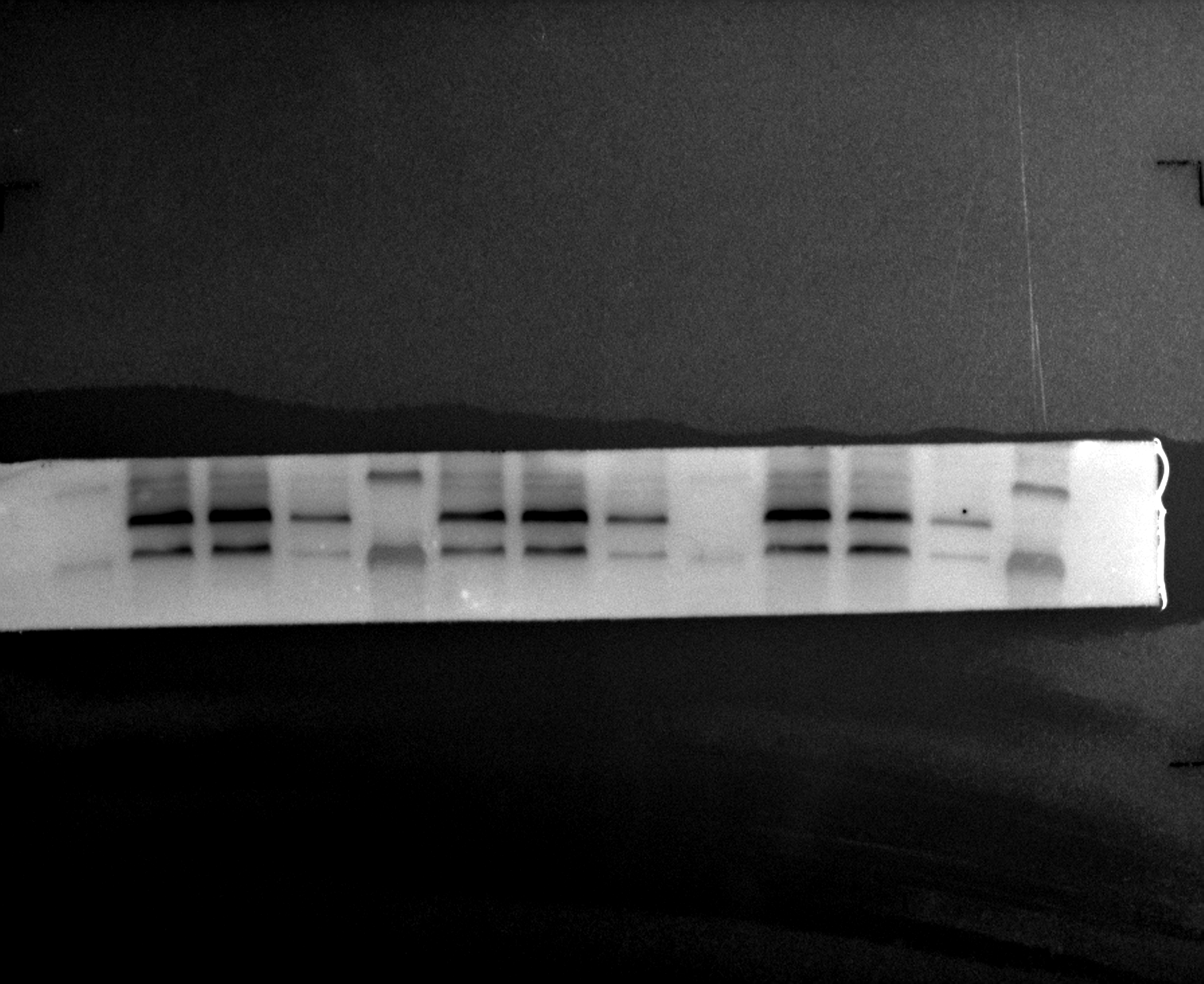

Supplement: Supplementary file 3 — Additional file 3. [file 13287_2026_4964_MOESM3_ESM.zip › Raw WB data 0809/PINK1ParkinLC3BBeclin-1P62 HaCaT/P62全膜.Tif]

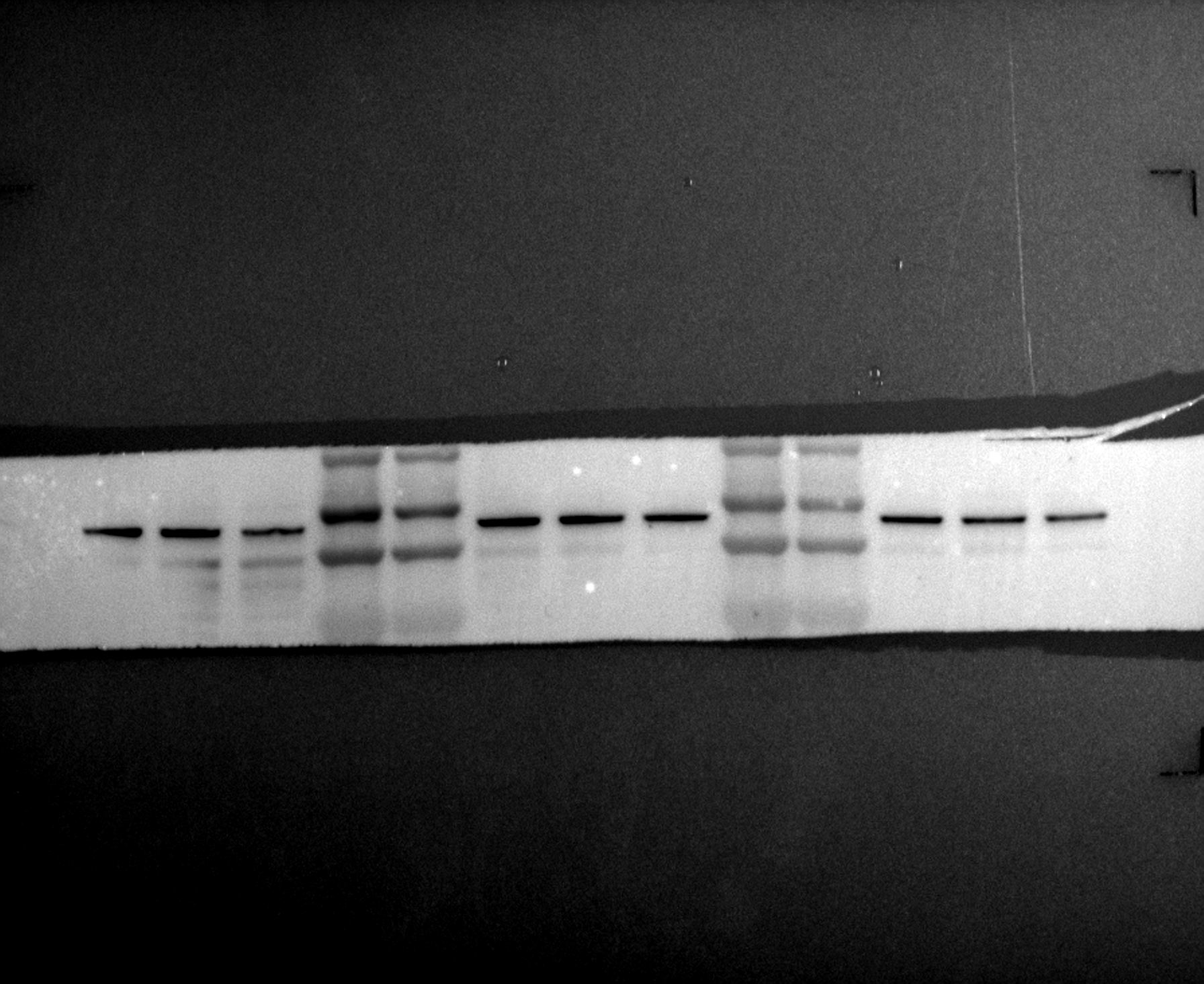

Supplement: Supplementary file 3 — Additional file 3. [file 13287_2026_4964_MOESM3_ESM.zip › Raw WB data 0809/PINK1ParkinLC3BBeclin-1P62 HaCaT/gapdh-1-2s-r..配beclin-1.Tif]

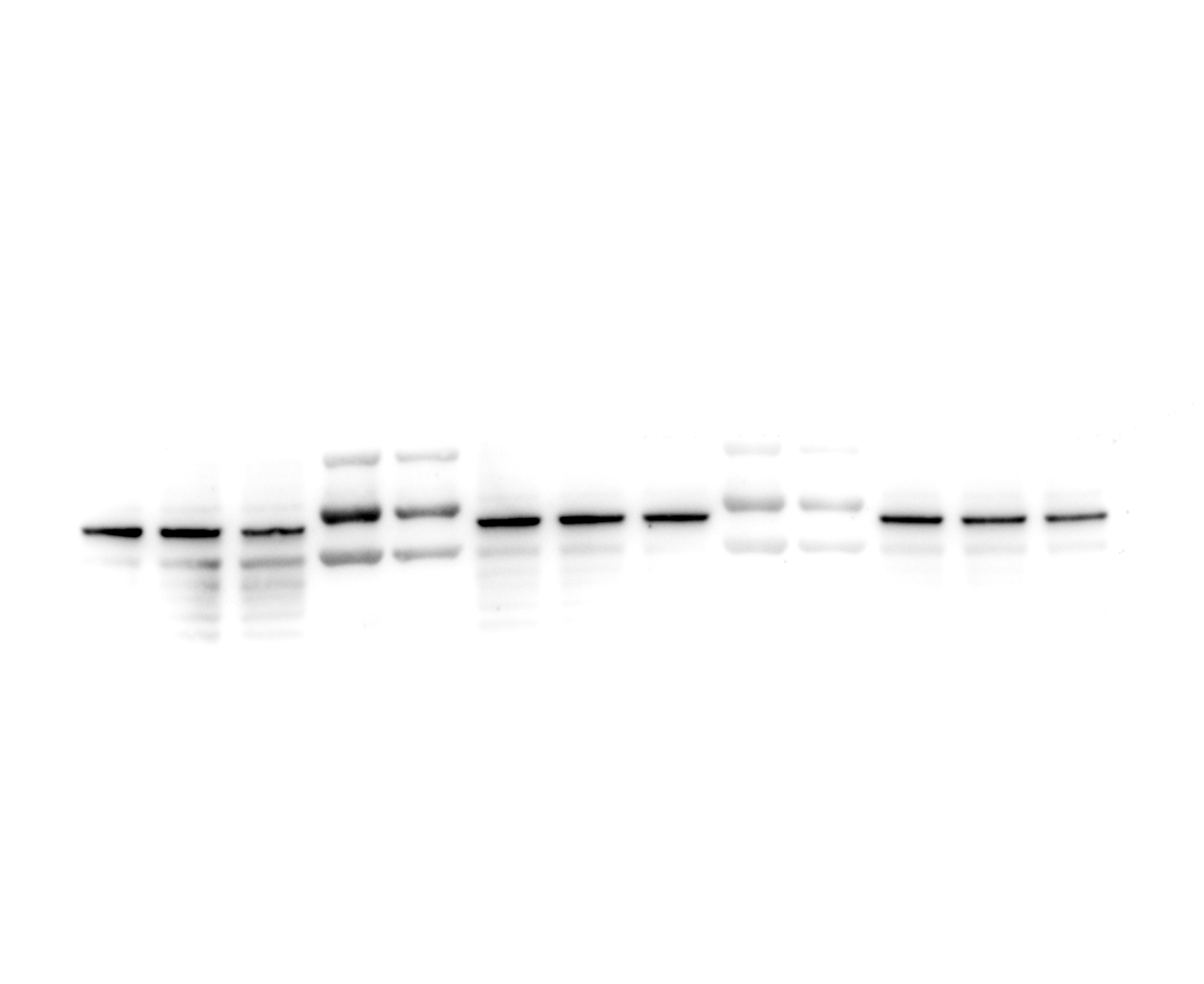

Supplement: Supplementary file 3 — Additional file 3. [file 13287_2026_4964_MOESM3_ESM.zip › Raw WB data 0809/PINK1ParkinLC3BBeclin-1P62 HaCaT/gapdh-1-2s.配beclin-1.Tif]

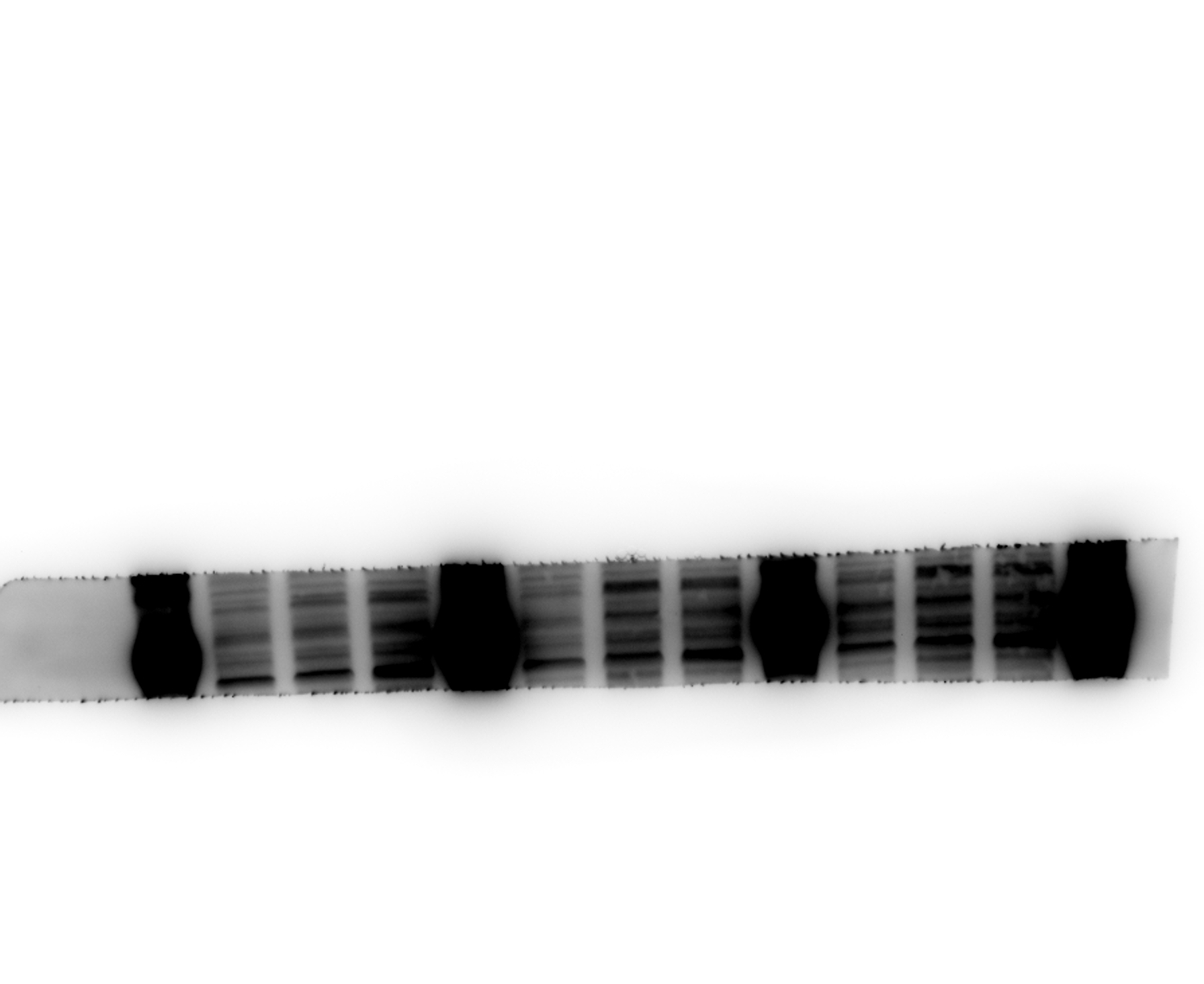

Supplement: Supplementary file 3 — Additional file 3. [file 13287_2026_4964_MOESM3_ESM.zip › Raw WB data 0809/PINK1ParkinLC3BBeclin-1P62 HaCaT/pink1-2s全膜.tif.tif]
