# Supplementary material for: Research based on serine metabolism indicates mesenchymal stem cells alleviate psoriasis by regulating the PSPH-PINK1-Parkin-NLRP3 pathway in HaCaT
Source: Stem Cell Res Ther. 2026 Mar 28;17:176. doi: 10.1186/s13287-026-04964-z (PMC13151198; doi:10.1186/s13287-026-04964-z)
Supplement: Supplementary file 4 — Additional file 4. [file 13287_2026_4964_MOESM4_ESM.zip › supplementary materials 0806/Supplementary Material 2.docx]

Primers sequences targeting genes in HaCaT KCs were as follows:

IL-1β,5′-ATGATGGCTTATTACAGTGGCAA3′(forward)and5′-GTCGGAGATTCGTAGCTG GA-3′ (reverse),

IL-6,5′-ACTCACCTCTTCAGAACGAATTG-3′(forward)and5′-CCATCTTTGGAAGGTTCA GGTTG-3′ (reverse),

IL-8,5′-ACTGAGAGTGATTGAGAGTGGAC3′(forward)and5′-AACCCTCTGCACCCAGTT TTC-3′ (reverse),

TNF-α,5′-CCTCTCTCTAATCAGCCCTCTG-3′(forward)and5′-GAGGACCTGGGAGTAGAT GAG-3′ (reverse),

MCP-1,5′-CAGCCAGATGCAATCAATGCC3′(forward)and5′-TGGAATCCTGAACCCACT TCT-3′ (reverse),

GAPDH,5′-CACATGGCCTCCAAGGAGTAA-3′(forward)and5′-TGAGGGTCTCTCTCTTCCTCT TGT-3′ (reverse),

KRT6,5′-GGGTTTCAGTGCCAACTCAG-3′(forward)and5′-CCAGGCCATACAGACTGCGG-3′(reverse),

KRT1,5′-CTTTTCTGCTGTTTCCCAATGAA-3′(forward)and5′-GGAAAGAACAAAGCAGGG TCATAG-3′ (reverse),

CCL20,5′-GGCGAATCAGAAGCAAGCAA-3′ (forward) and 5′-

GGATTTGCGCACACAGACAA-3′ (reverse),

CCL27, 5′-CCAGACCCTACAGCAGCATT-3′ (forward) 5′-

TGCAGTTCCACCTGGATGAC-3′ (reverse),

CCL7, 5′-GAAGGACCACCAGTAGCCAC-3′ (forward) and 5′-

GTCCTGGACCCACTTCTGTG-3 (reverse),

PSPH , 5′-TGTCAGAAATGACACGGCGA-3′ (forward) and 5′-

GTAGGCGACTTACCAGCTCC -3′ (reverse),

Primers sequences targeting genes in mice were as follows:

IL-6,5′-CCTCTCTGCAAGAGACTTCCAT-3′(forward)and5′-AGTCTCCTCTCCGGACTTGT-3′ (reverse),

TNF-α,5′-ATCCGCGACGTGGAACTG-3′(forward)and5′-ACCGCCTGGAGTTCTGGAA-3′ (reverse),

GAPDH,5′-AGCTTGTCATCAACGGGAAG-3′(forward)and5′-TTTGATGTTAGTGGGGTC TCG-3′ (reverse),

CCL20,5′-AGCCAGGCAGAAGCAAGCAAC-3′(forward)and5′-ATCGGCCATCTGTCTTGTGAAACC′-3′(reverse),

MCP-1,5′-CACTCACCTGCTGCTACTCATTCAC-3′(forward)and5′-CTTCTTTGGGACACCTGCT GCTG-3′(reverse),

CCL7,5′-GTGGTTAGAACGCCAAGGGA-3′(forward)and5′-TTGCTGTTGGGGGTTTGAGT-3′ (reverse),

IL-1β, 5′-GGGCTGCTTCCAAACCTTTG-3′(forward) and 5′-

AAGACACAGGTAGCTGCCAC-3′ (reverse),

IL8, 5′-TCTGCAGCTCTGTGTGAAGG (forward) and 5′-

TGGGGTGGAAAGGTTTGGAG-3′(reverse),

CCL27, 5′-GAAGGACCACCAGTAGCCAC-3′(forward) and 5′-

GTCCTGGACCCACTTCTGTG-3′(reverse).6
