# Supplementary material for: Research based on serine metabolism indicates mesenchymal stem cells alleviate psoriasis by regulating the PSPH-PINK1-Parkin-NLRP3 pathway in HaCaT
Source: Stem Cell Res Ther. 2026 Mar 28;17:176. doi: 10.1186/s13287-026-04964-z (PMC13151198; doi:10.1186/s13287-026-04964-z)

A S C - 6 0 0 M S

A S C 全 膜

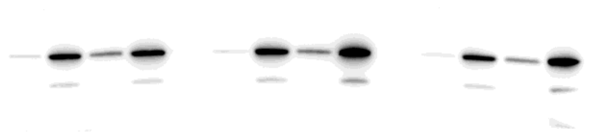

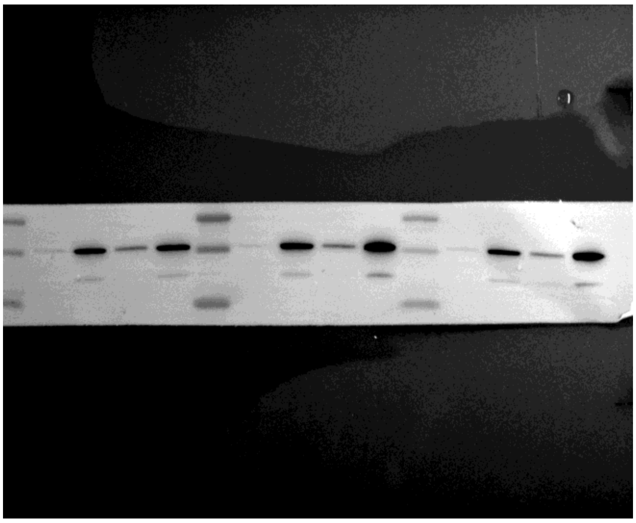

G A P D H - 8 0 0 M S

G A P D H 全 膜

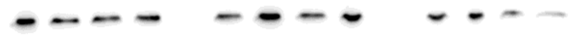

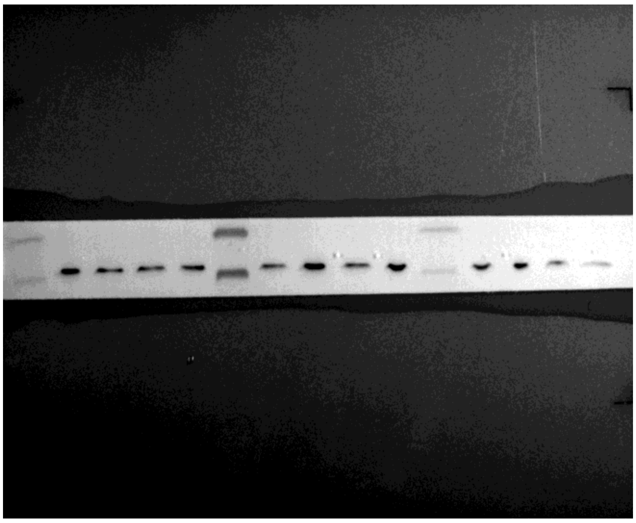

p a r k i n - 5 s

N L R P 3 全 膜

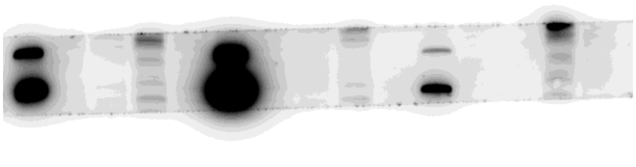

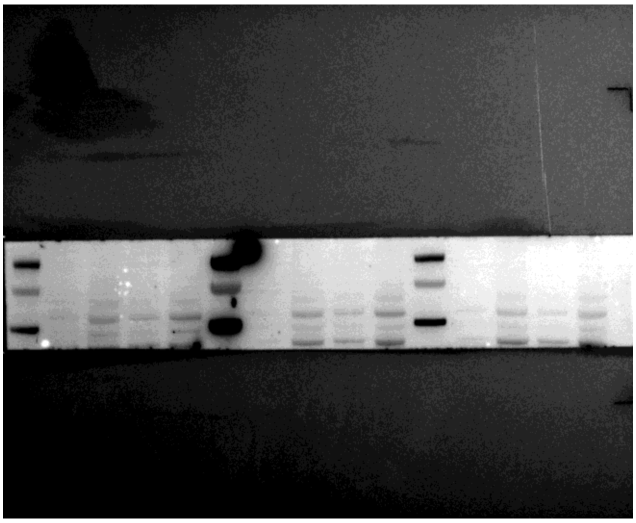

NLRP3-30S parkin-10s

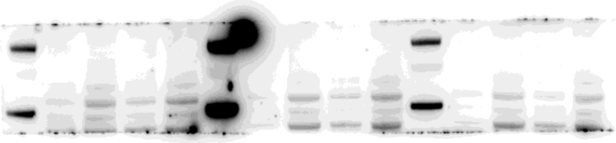

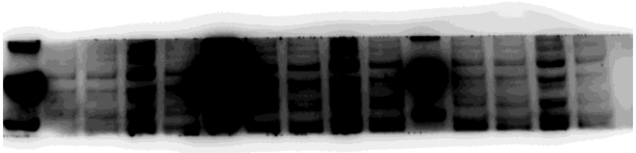

parkin 全膜.Tif0429 parkin 全膜

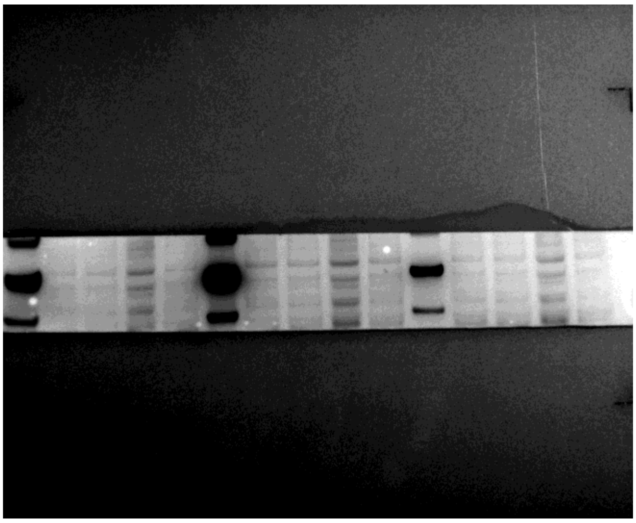

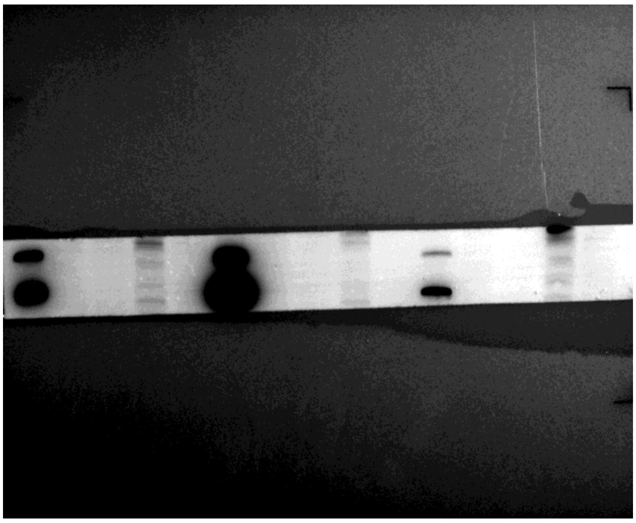

全膜拼接

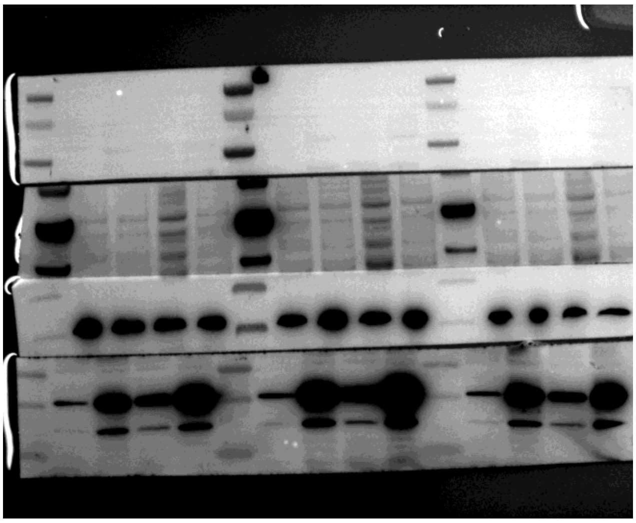

GAPDH 全膜

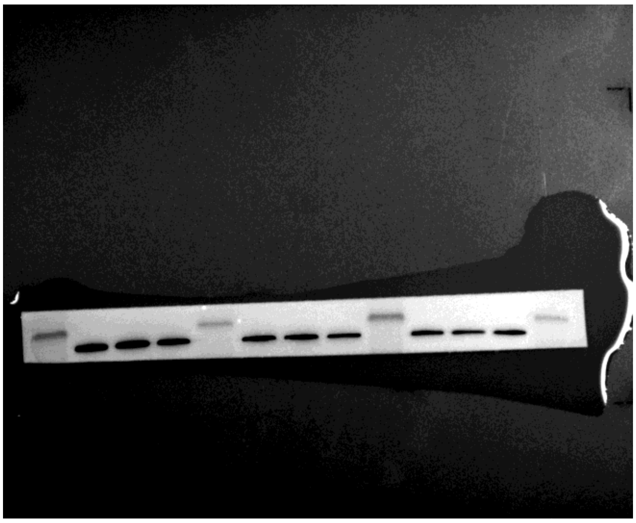

GAPDH

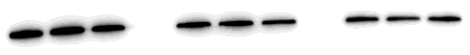

ASC 全膜

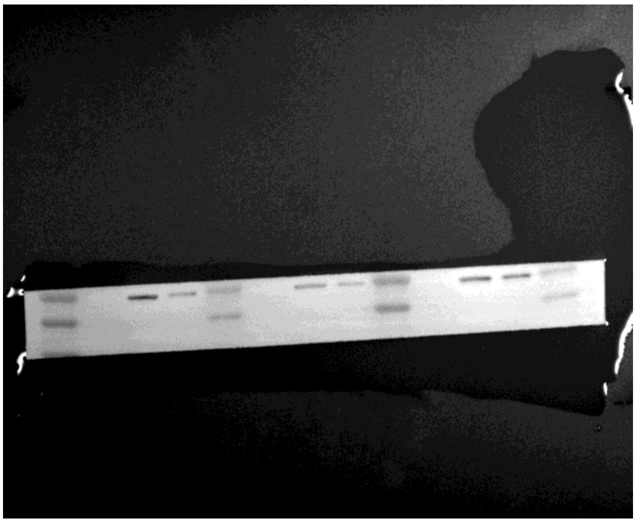

ASC-800ms

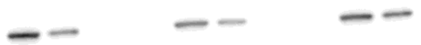

ASC+GAPDH 全膜

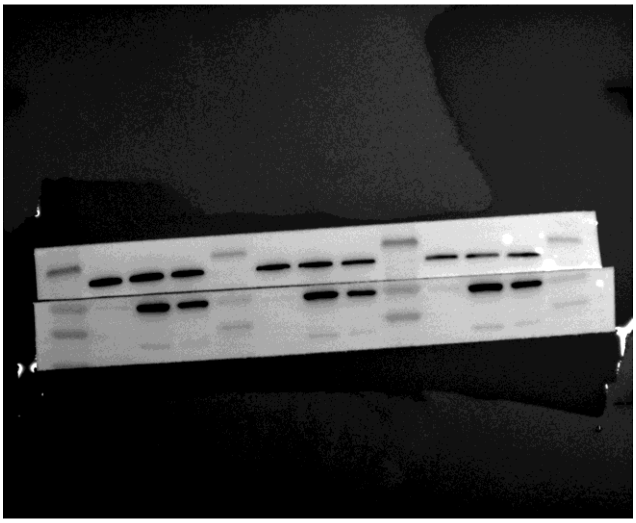

KRT6+300MS

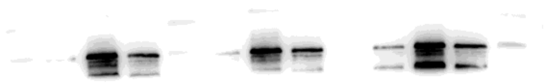

GAPDH 全膜

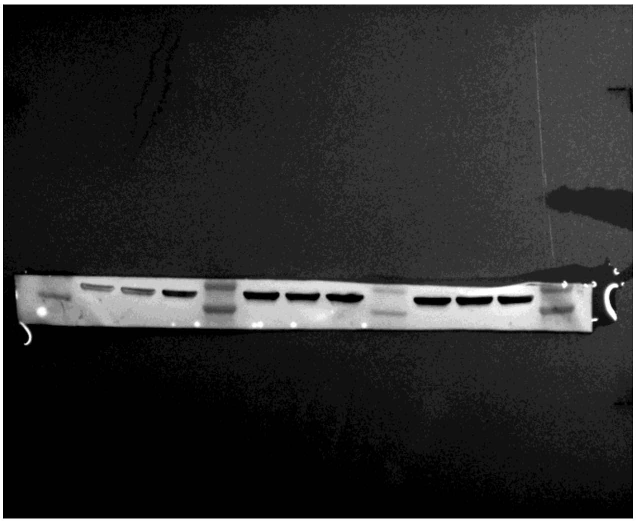

GAPDH+KRT6 全膜

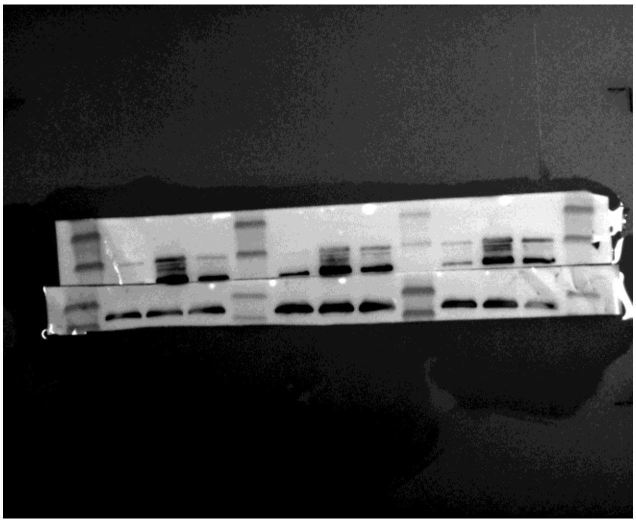

GAPDH+100ms

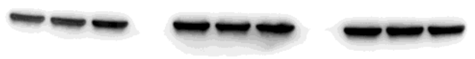

KRT6 全膜

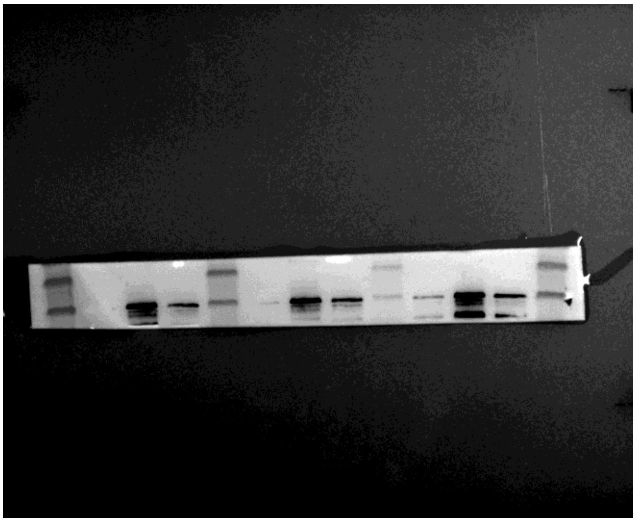

KRT1 全膜 2

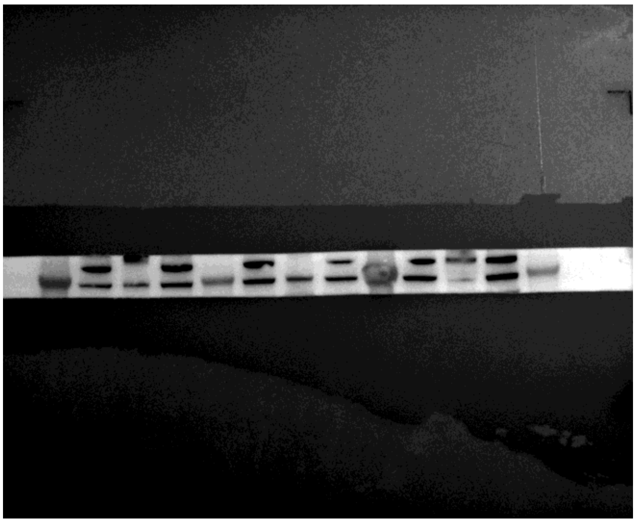

KRT1 全膜

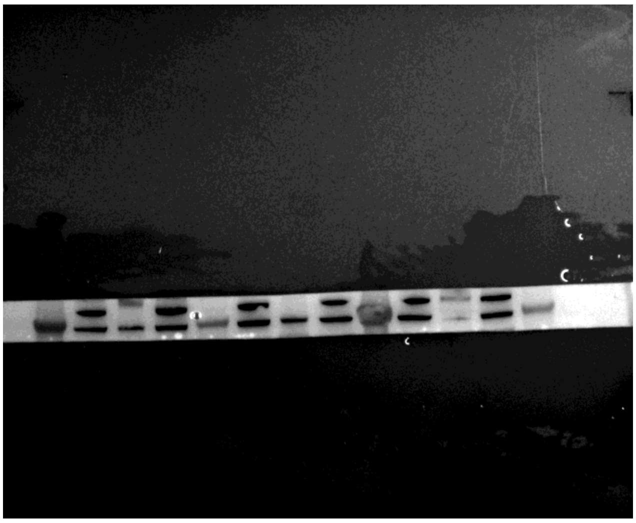

KRT1-300ms

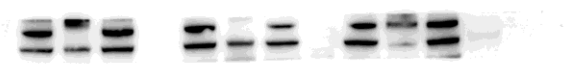

GAPDH 全膜

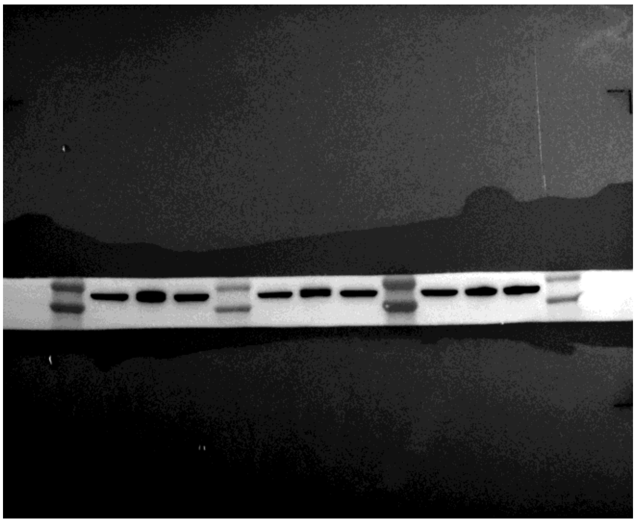

GAPDH+KRT1

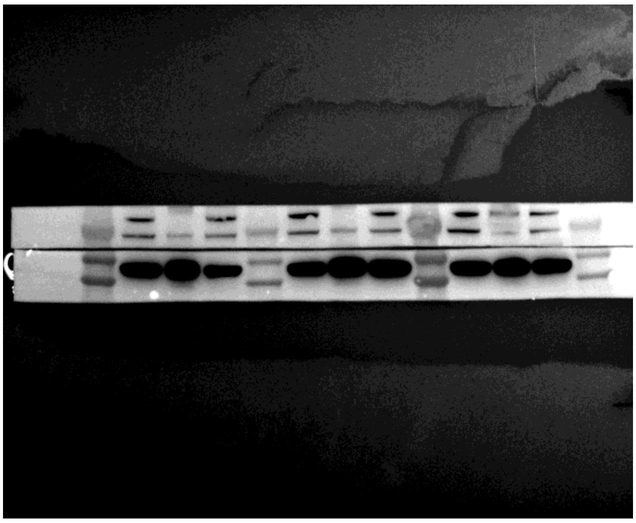

GAPDH

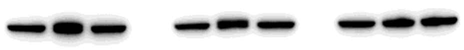

GAPDHquanmo

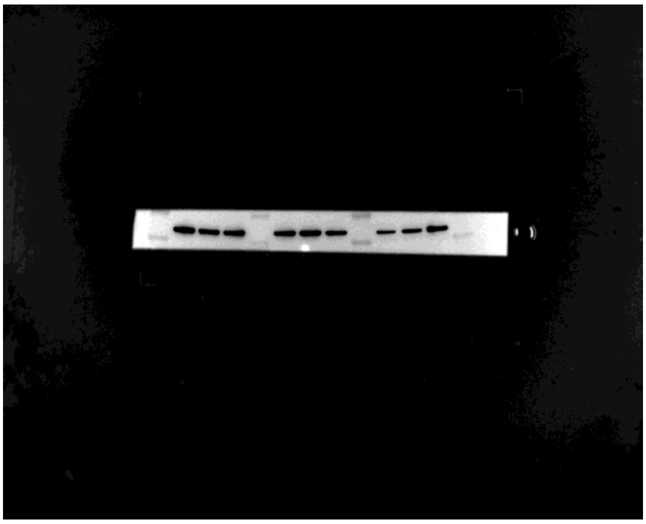

GAPDH-200ms

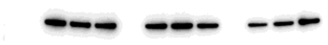

gapdh+ASCquanmo

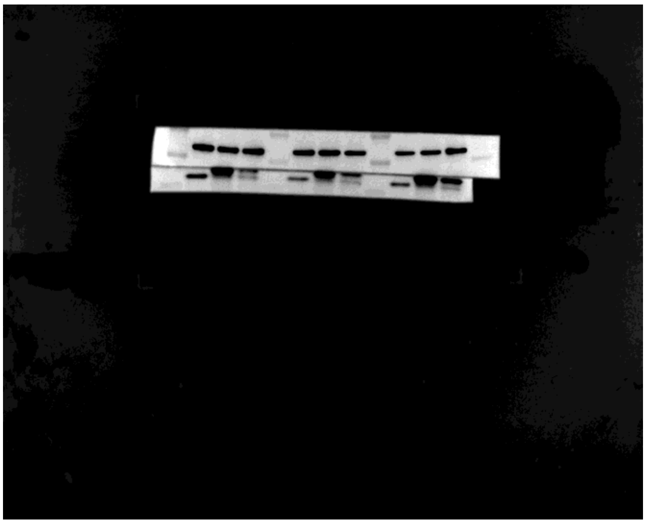

ascQUANMO

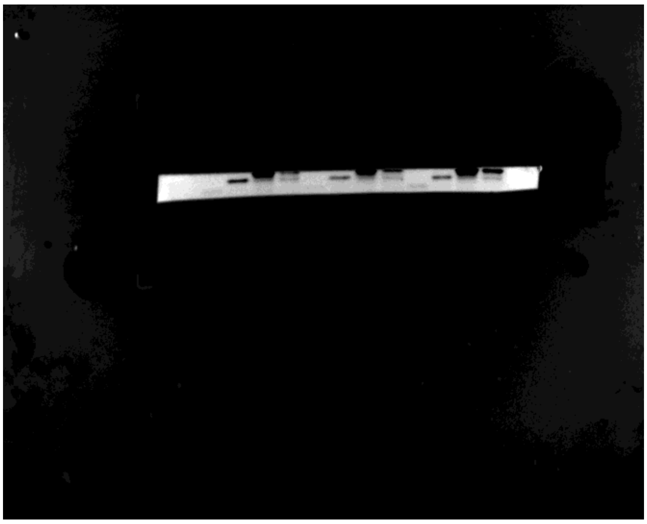

ASC-900ms 人的 3 组

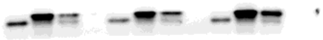

全膜 NLRP3+GAPDH

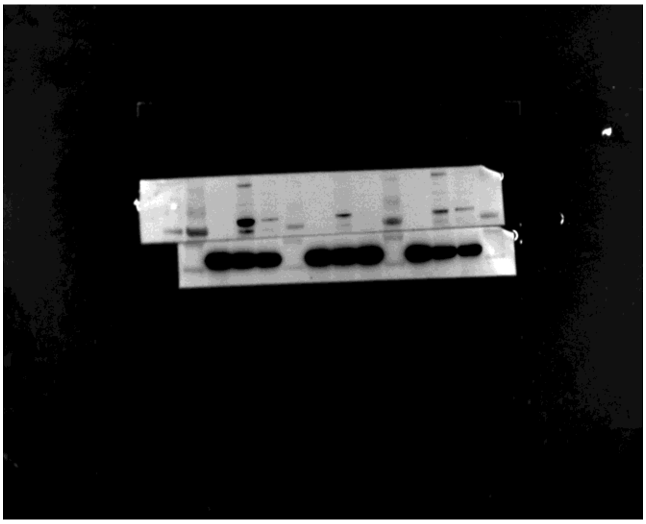

全膜 NLRP3

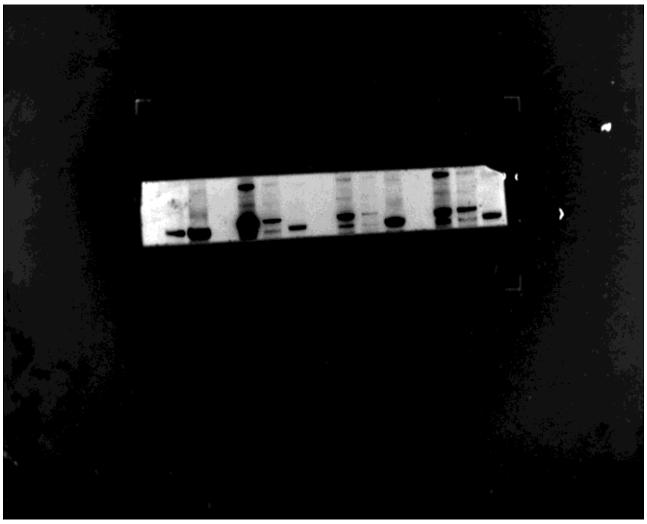

全膜 GAPDH

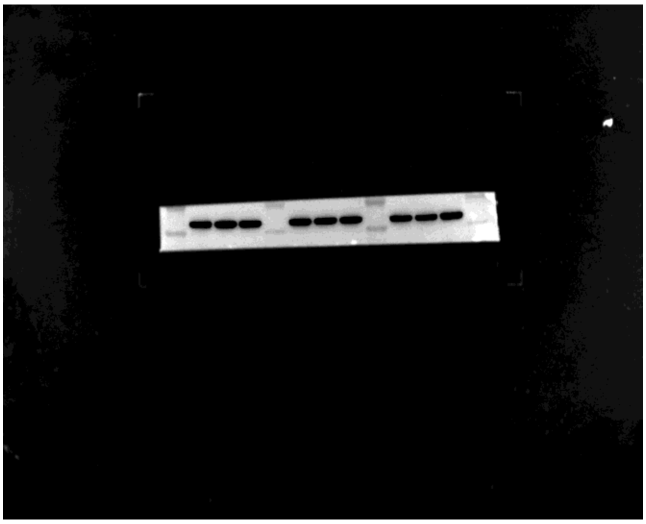

NLRP3+500ms

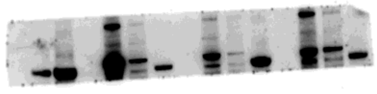

GAPDH+100MS

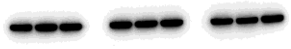

人 ASC-900ms 全膜

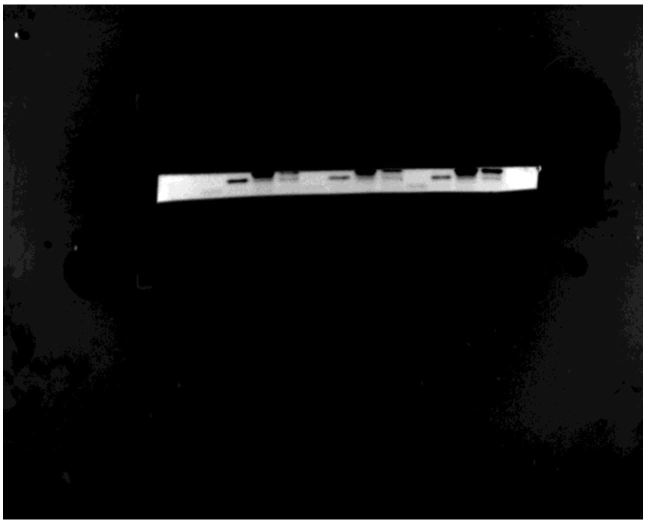

人 ASC-900ms

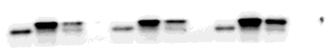

全膜人 NLRP3+GAPDH

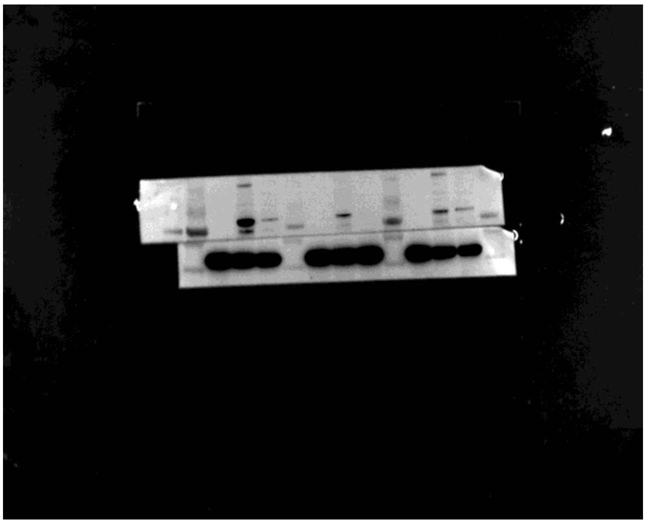

GAPDH-200ms

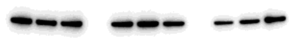

gapdh+人 ASCquanmo

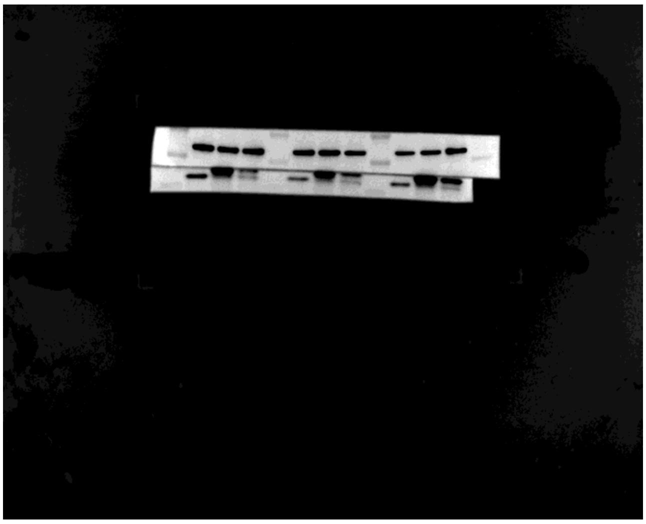

P62 全膜

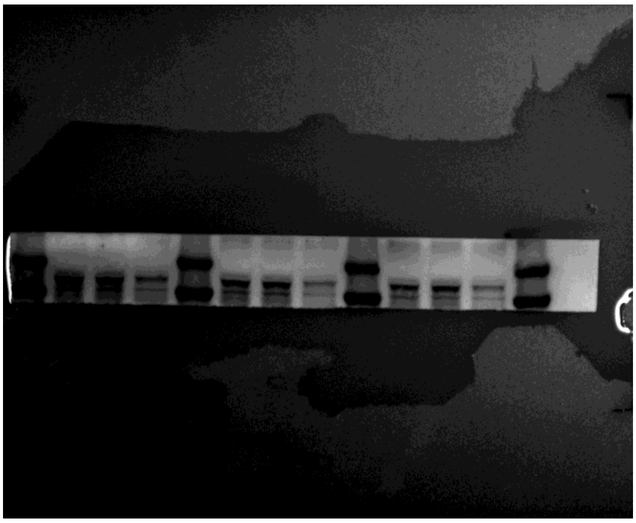

P62-3S

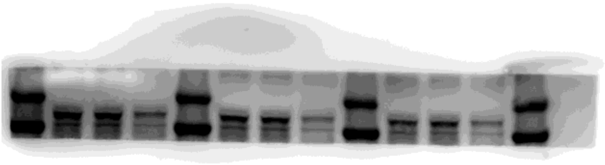

LC3B 全膜

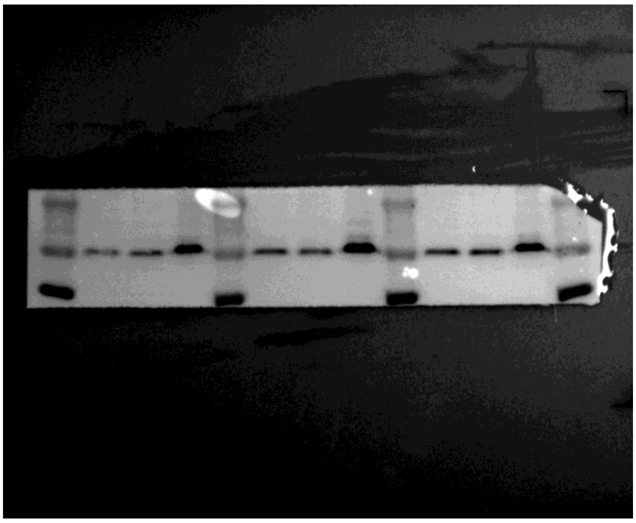

LC3B-1S

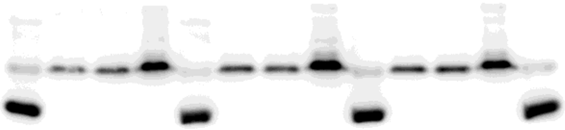

PINK1 全膜

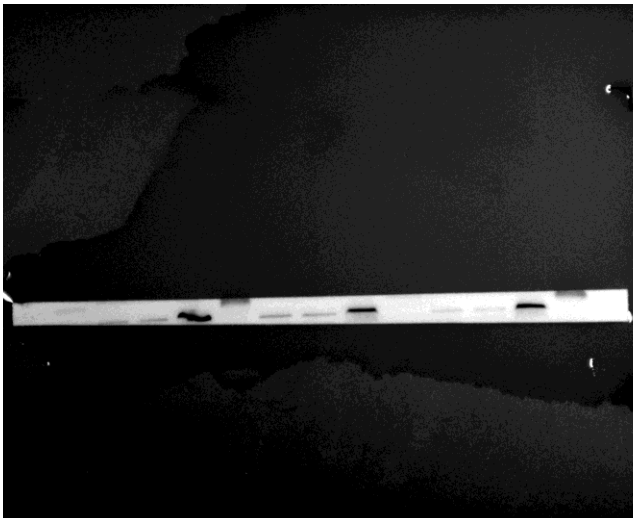

PINK1 rescuex20418

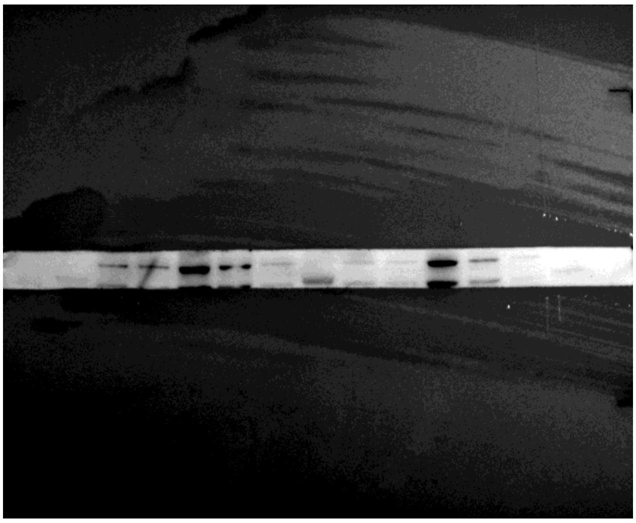

parkin 全膜

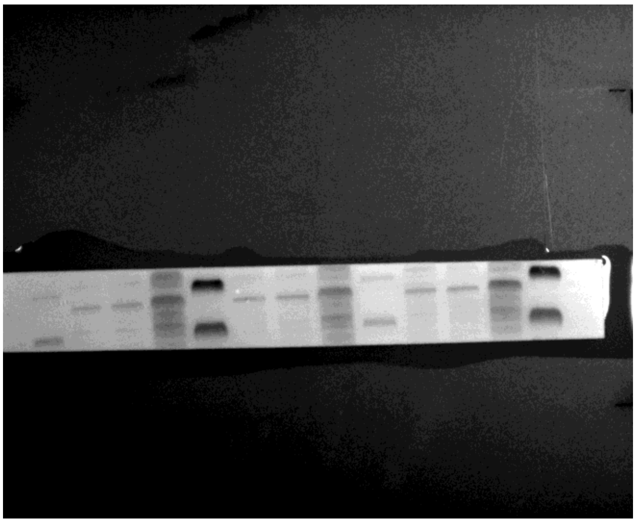

parkin-400ms

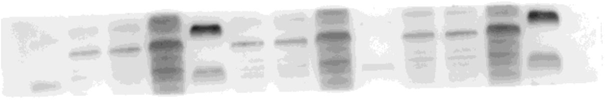

GAPDH 全膜 2

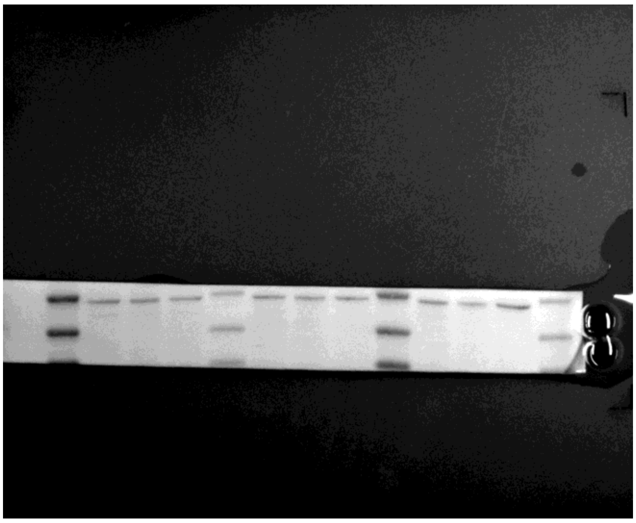

gapdh 全膜.Tif2

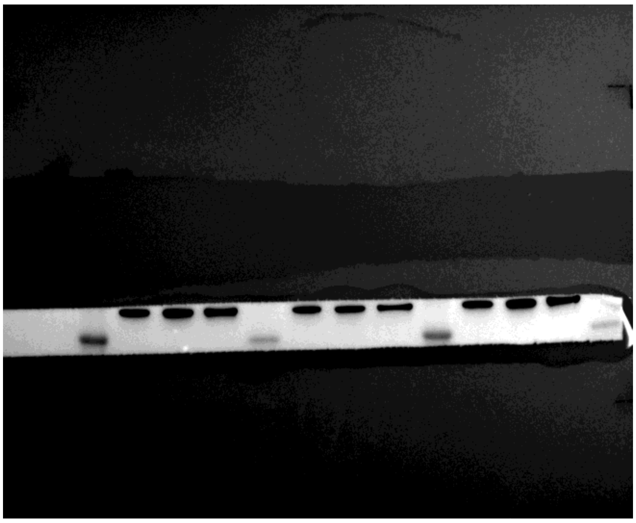

GAPDH %

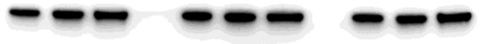

全膜-p62

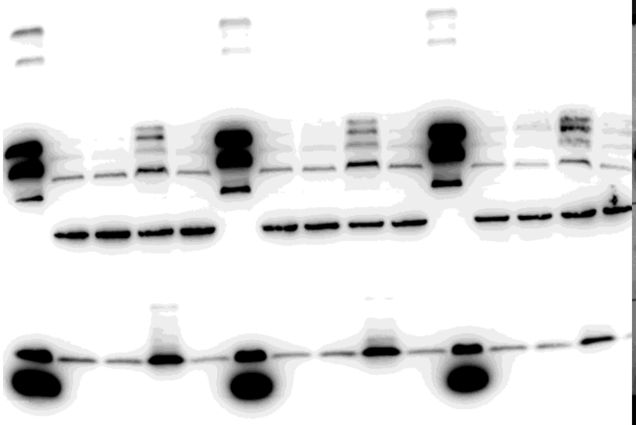

全膜 1-r-p62

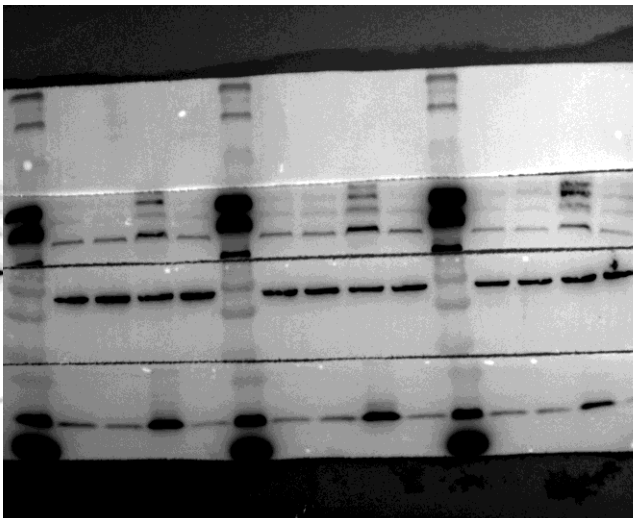

全膜 1-beclin1-r

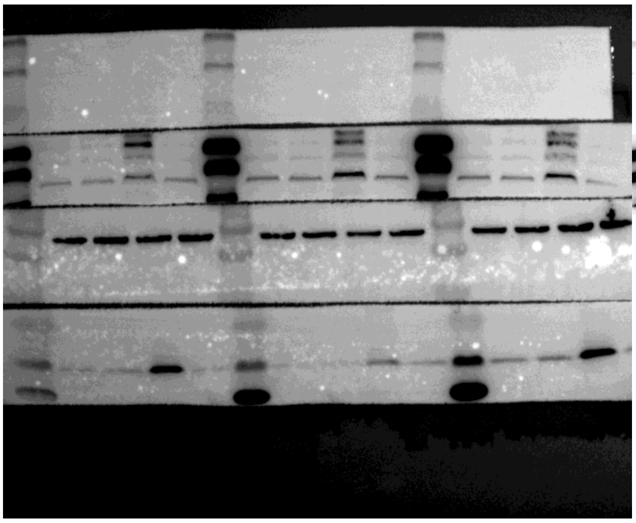

全膜 1-beclin1

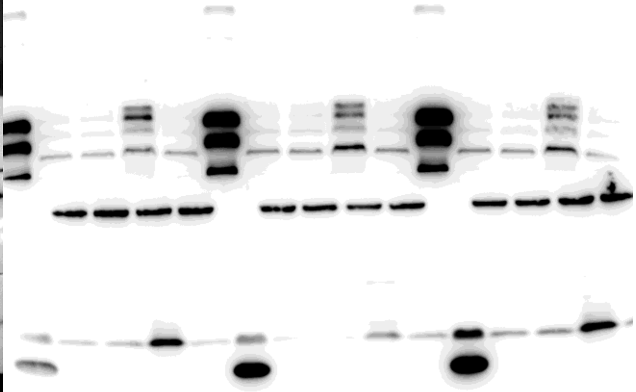

PINK-1-2s-r

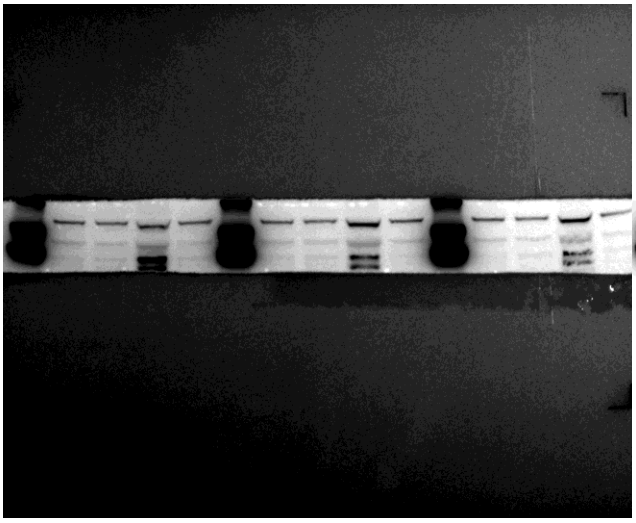

PINK1-2s

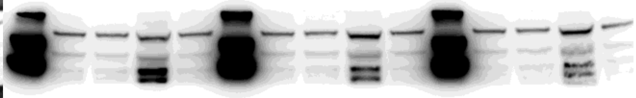

PARKIN-3S-R

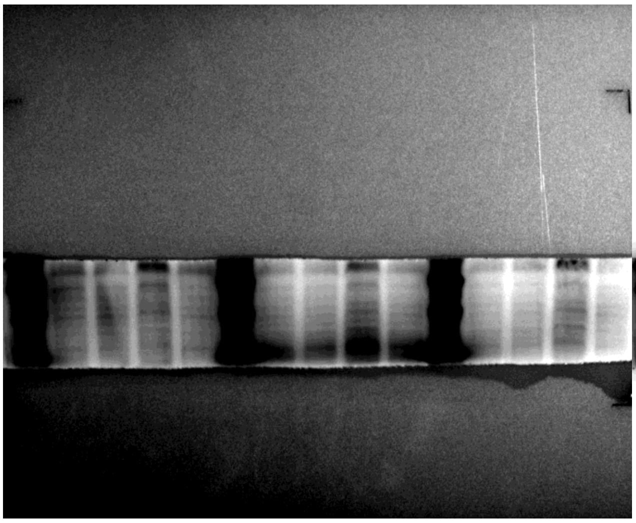

PARKIN-3S

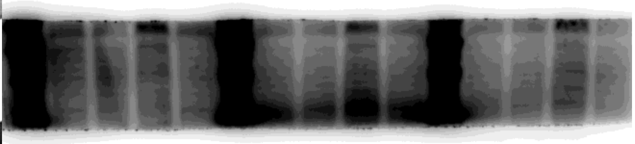

PARKIN-2.5S-R

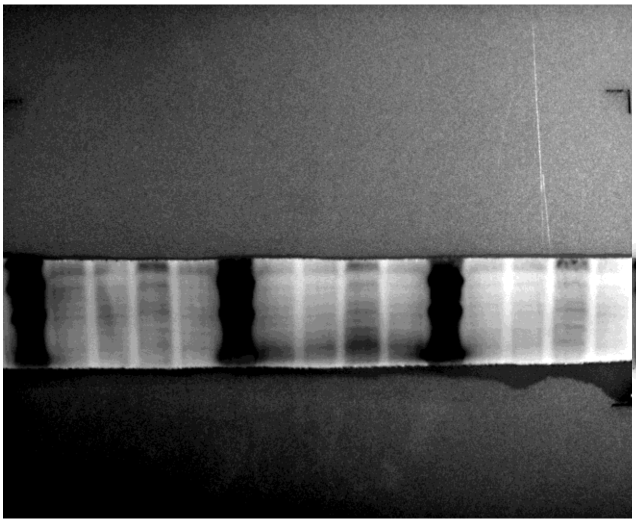

PARKIN-2.5S

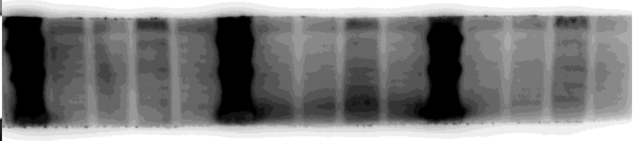

p62-40s-r

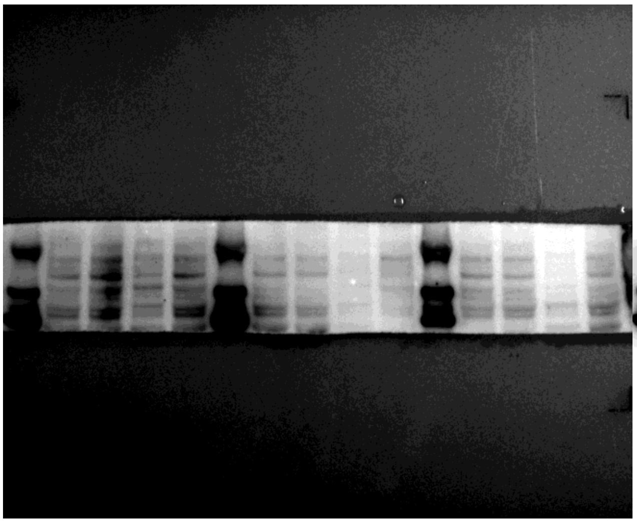

p62-40s

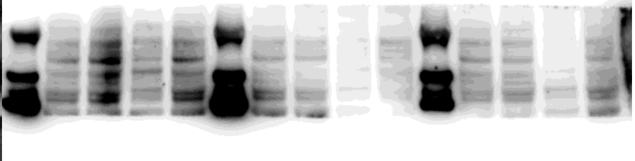

p62-30s-r

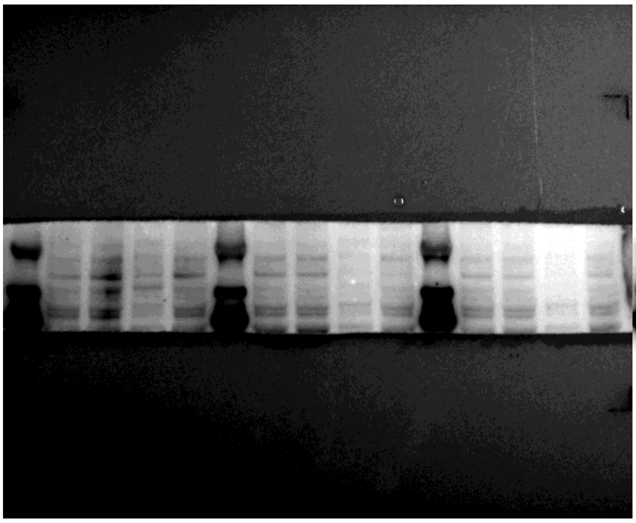

p62-30s

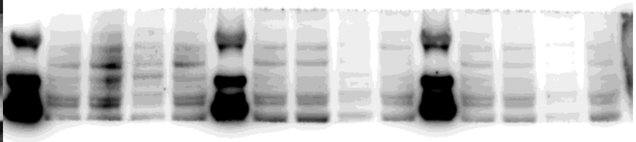

lc3-1s-r

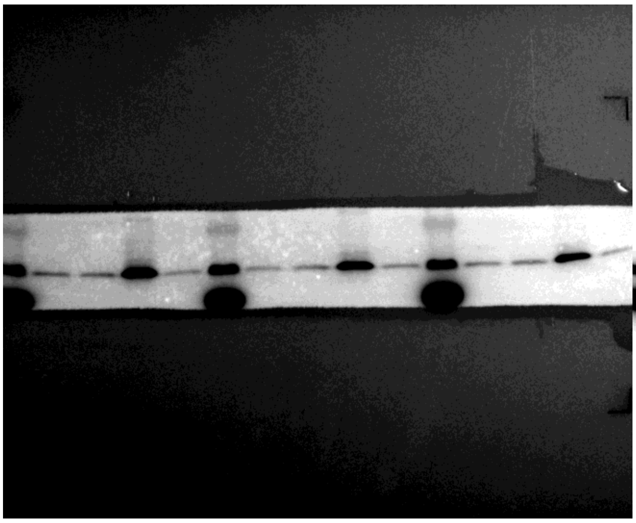

lc3-1s

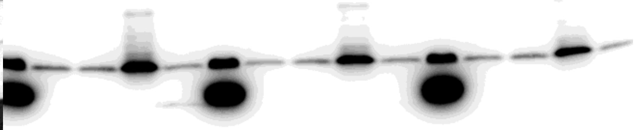

GAPDH-1S-R

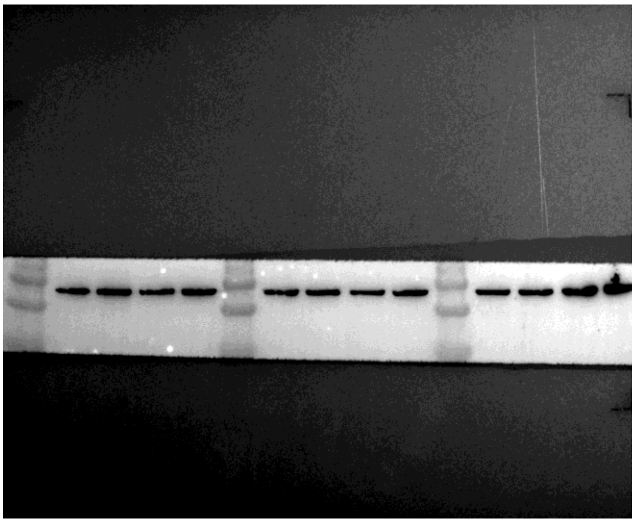

GAPDH-1S

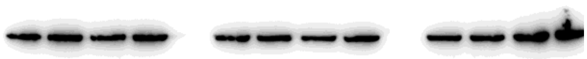

beclin1-5s-r

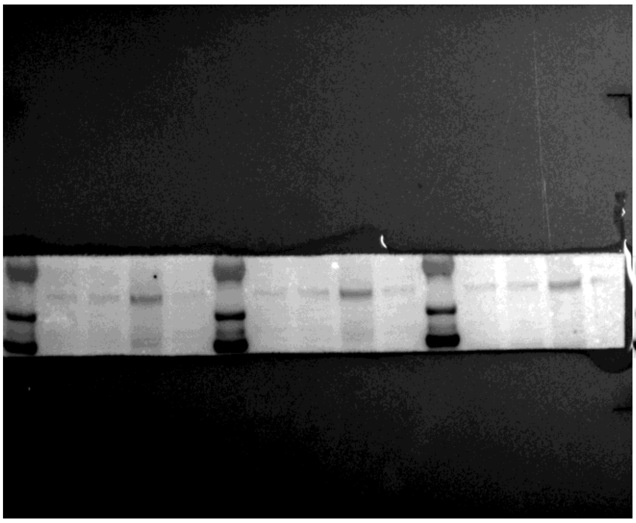

beclin1-5s

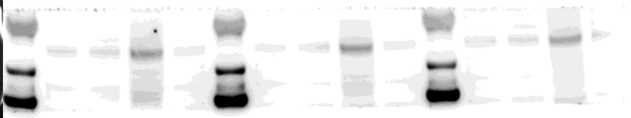

全膜 GAPDH

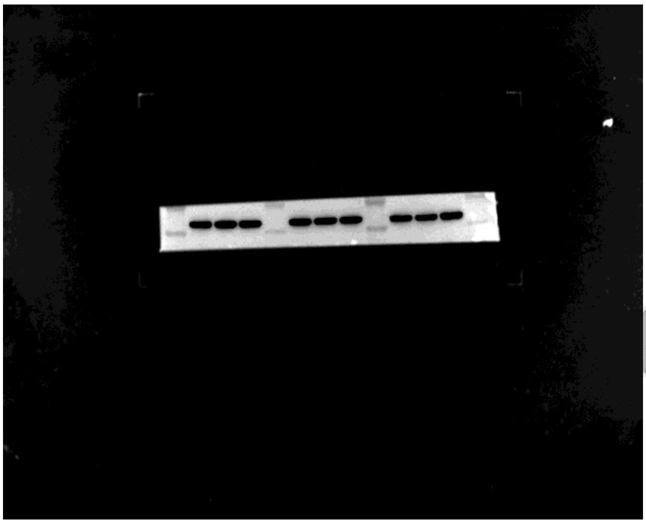

pink1-2s 全膜.tif

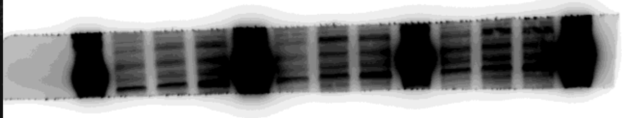

parkin-3s 全膜

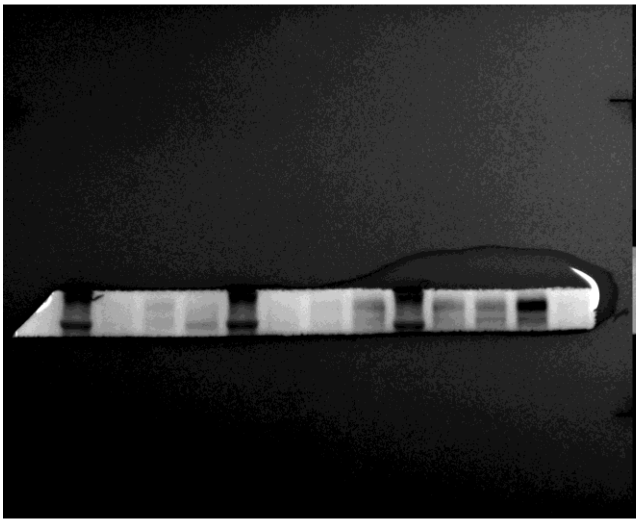

P62 全膜

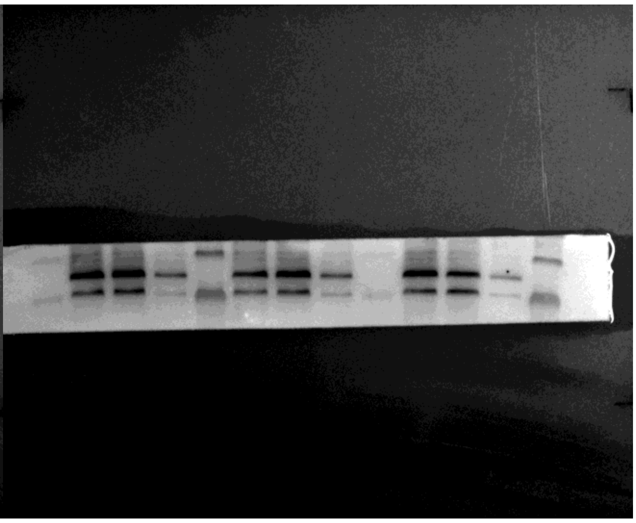

P62+30s

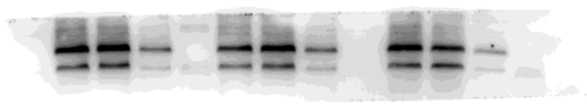

LC3B 全膜 2

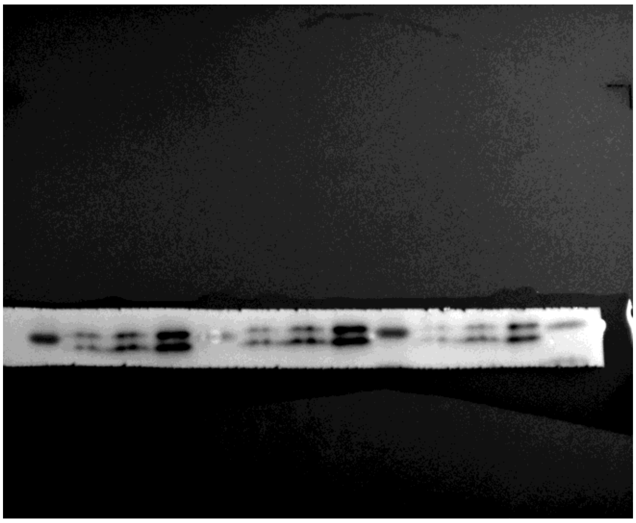

LC3B 2s

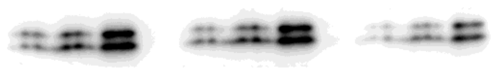

GAPDH 全膜

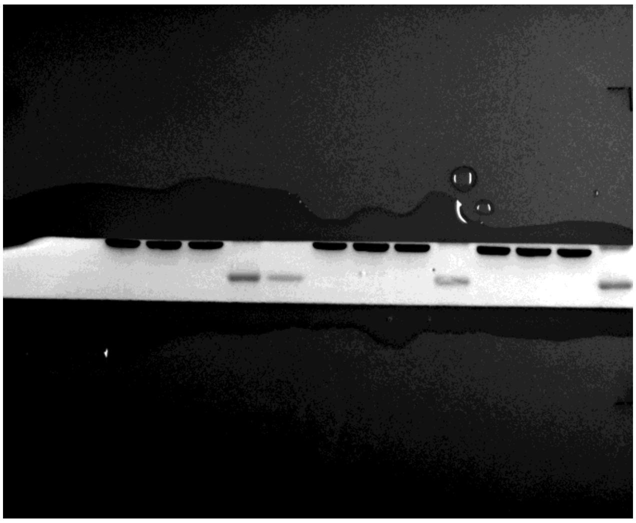

GAPDHquanmo

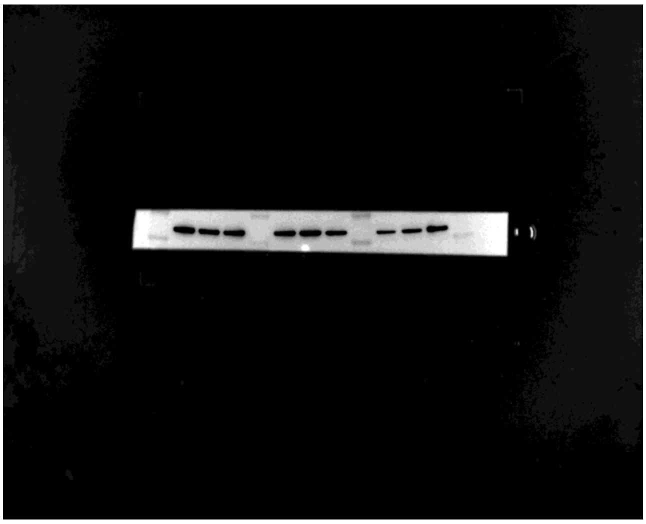

GAPDH-800ms

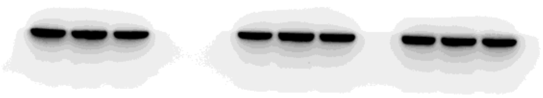

gapdh-1-2s-r..配 beclin-1

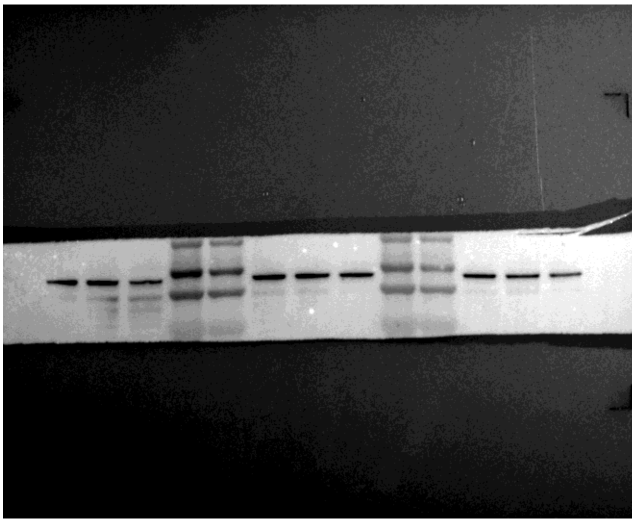

gapdh-1-2s.配 beclin-1

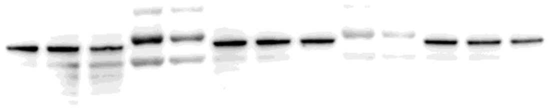

GAPDH+100MS

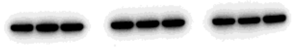

BECLIN1 全膜 24.10.06.x3

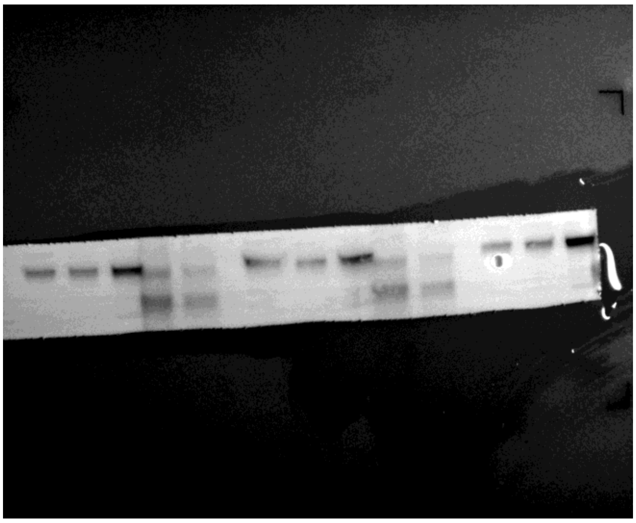

Beclin-1-2s 人 x

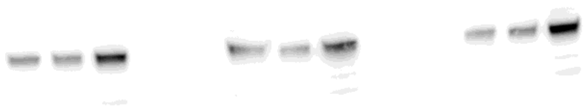

ACTIN

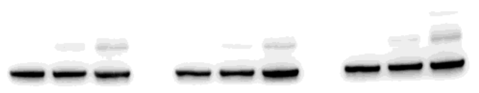

ACTINQUANMO

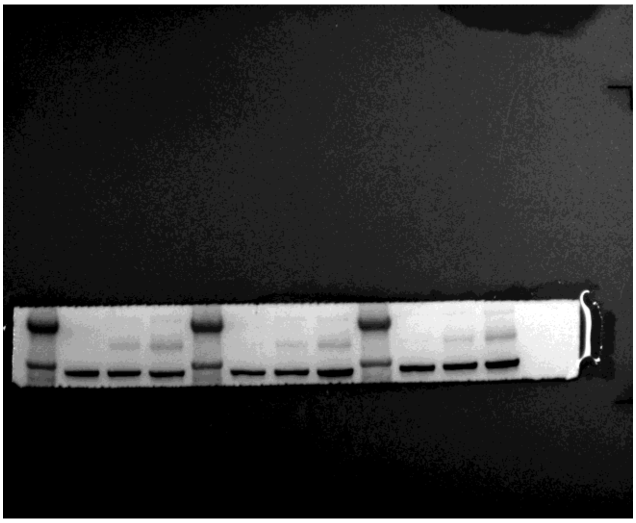

GAPDH80S

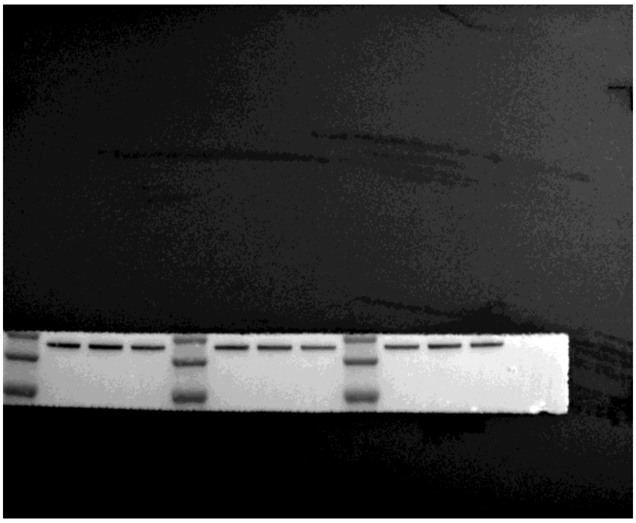

GAPDH1228

GAPDH80S

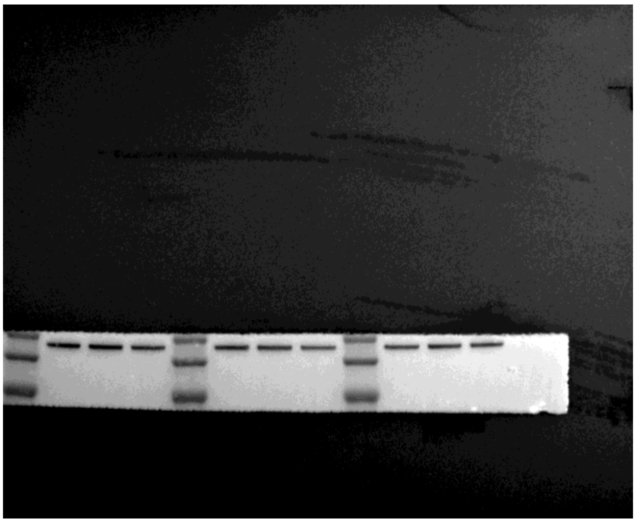

tom20-2

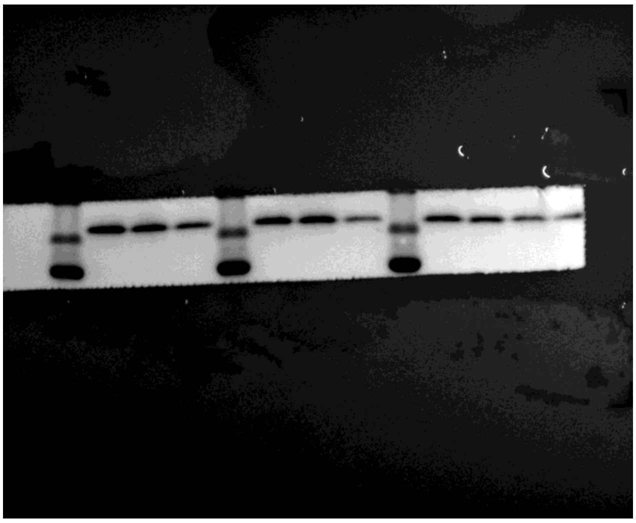

tomo20

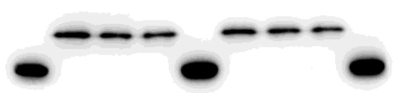

tomo20QUANMO

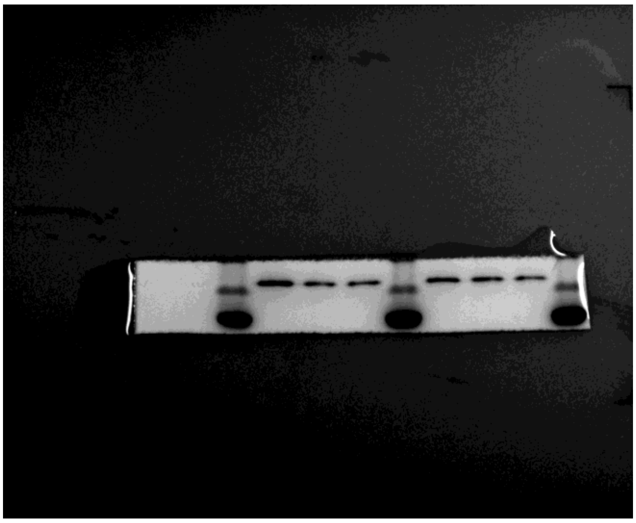

全膜合成

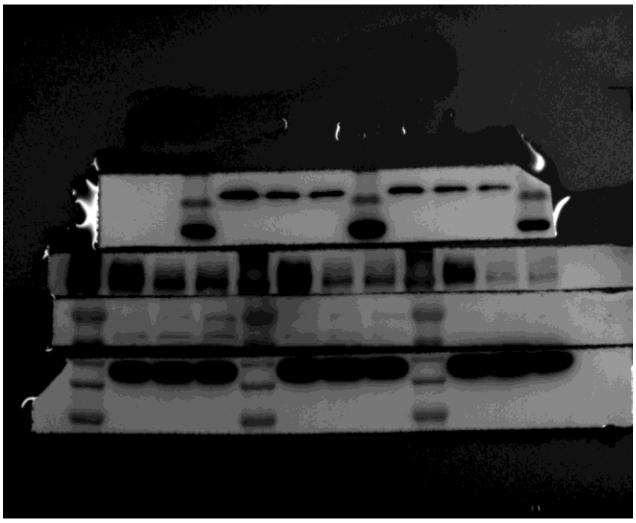

LC3B

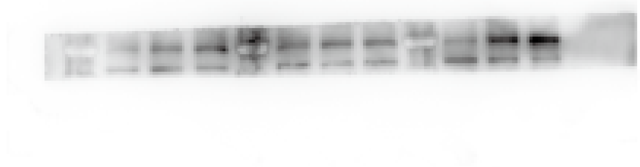

P62 30S.Ti 小鼠 x3 用

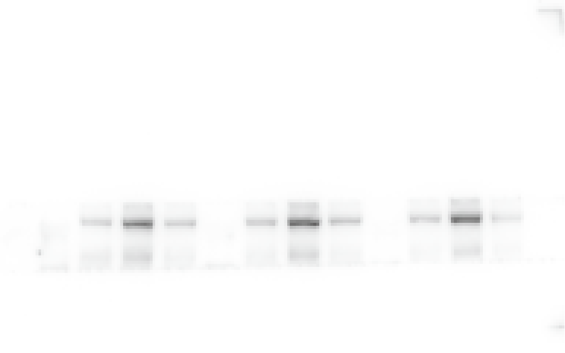

P62 60s

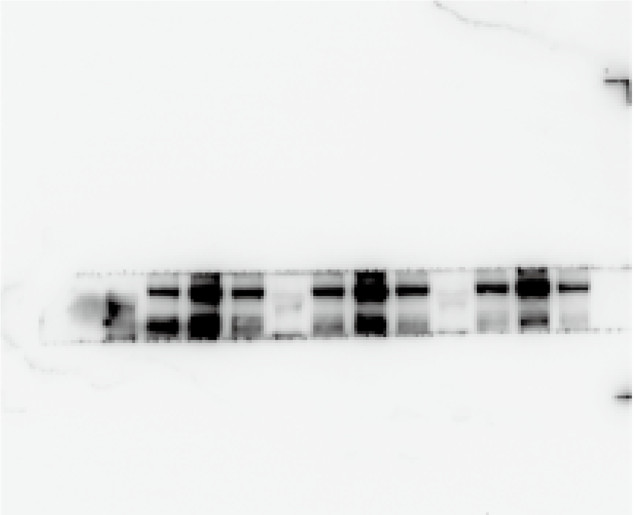

parkin

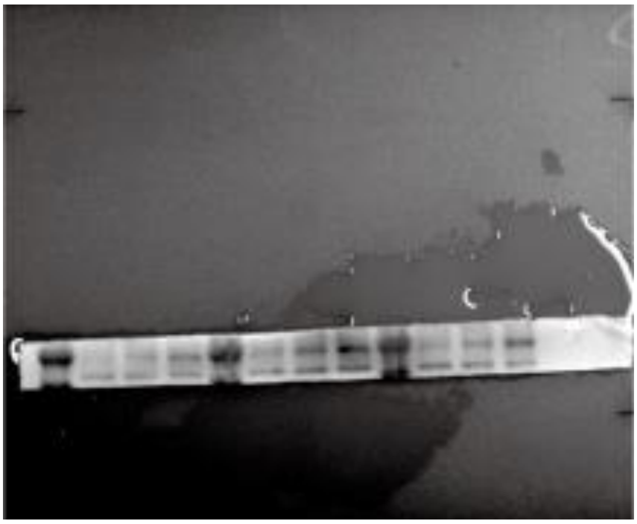

PINK1-1.小鼠 x1

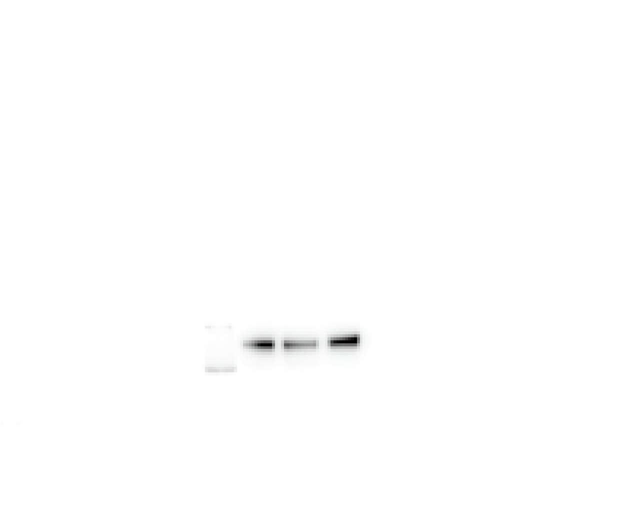

小鼠 parkinX3

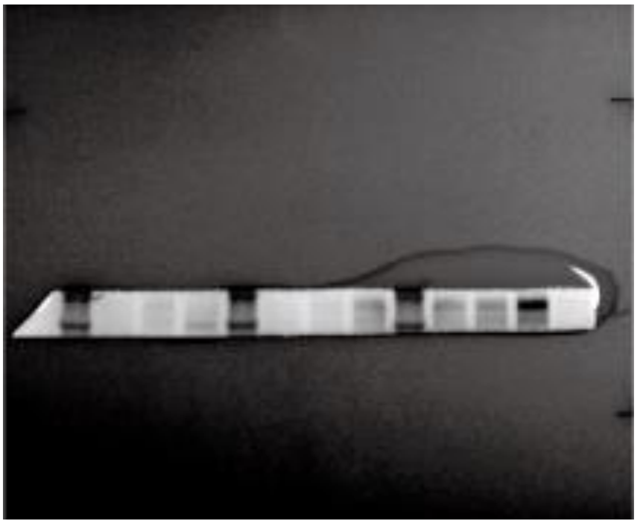

Supplement: Supplementary file 5 — Additional file 5. [file 13287_2026_4964_MOESM5_ESM.zip › supplementary materials 0829/WB raw data0829.pdf]
